# CBX4 deletion promotes tumorigenesis under Kras^G12D^ background by inducing genomic instability

**DOI:** 10.1038/s41392-023-01623-0

**Published:** 2023-09-12

**Authors:** Fangzhen Chen, Wulei Hou, Xiangtian Yu, Jing Wu, Zhengda Li, Jietian Xu, Zimu Deng, Gaobin Chen, Bo Liu, Xiaoxing Yin, Wei Yu, Lei Zhang, Guoliang Xu, Hongbin Ji, Chunmin Liang, Zuoyun Wang

**Affiliations:** 1 Department of Human Anatomy and Histoembryology, School of Basic Medical Sciences and Shanghai Xuhui Central Hospital, Shanghai Medical College, Fudan University, Shanghai, 200030 China; 2 Shanghai Xuhui Central Hospital, Zhongshan-Xuhui Hospital, Fudan University, Shanghai, 200031 China; 3 Clinical Research Center, Shanghai Jiao Tong University Affiliated Sixth People’s Hospital, Shanghai, China; 4 State Key Laboratory of Cell Biology, CAS Center for Excellence in Molecular Cell Science, Shanghai Institute of Biochemistry and Cell Biology, Chinese Academy of Sciences, Shanghai, China; 5 CAS Key Laboratory of Molecular Virology and Immunology, Institute Pasteur of Shanghai, Chinese Academy of Sciences, Shanghai, China; 6 Department of General Surgery, Jing’an District Central Hospital of Shanghai, Fudan University, Shanghai, China; 7 Key Laboratory of Respiratory Disease, People’s Hospital of Yangjiang, Yangjiang, Guangdong China

**Keywords:** Oncogenes, Epigenetics, Genomic instability

## Abstract

Chromobox protein homolog 4 (CBX4) is a component of the Polycomb group (PcG) multiprotein Polycomb repressive complexes 1 (PRC1), which is participated in several processes including growth, senescence, immunity, and tissue repair. CBX4 has been shown to have diverse, even opposite functions in different types of tissue and malignancy in previous studies. In this study, we found that CBX4 deletion promoted lung adenocarcinoma (LUAD) proliferation and progression in Kras^G12D^ mutated background. In vitro, over 50% *Cbx4*^*L/L*^*, Kras*^*G12D*^ mouse embryonic fibroblasts (MEFs) underwent apoptosis in the initial period after Adeno-Cre virus treatment, while a small portion of survival cells got increased proliferation and transformation abilities, which we called selected *Cbx4*^*−/−*^*, Kras*^*G12D*^ cells. Karyotype analysis and RNA-seq data revealed chromosome instability and genome changes in selected *Cbx4*^*−/−*^*, Kras*^*G12D*^ cells compared with *Kras*^*G12D*^ cells. Further study showed that P15, P16 and other apoptosis-related genes were upregulated in the primary *Cbx4*^*−/−*^*, Kras*^*G12D*^ cells due to chromosome instability, which led to the large population of cell apoptosis. In addition, multiple pathways including Hippo pathway and basal cell cancer-related signatures were altered in selected *Cbx4*^*−/−*^*, Kras*^*G12D*^ cells, ultimately leading to cancer. We also found that low expression of CBX4 in LUAD was associated with poorer prognosis under Kras mutation background from the human clinical data. To sum up, CBX4 deletion causes genomic instability to induce tumorigenesis under Kras^G12D^ background. Our study demonstrates that CBX4 plays an emerging role in tumorigenesis, which is of great importance in guiding the clinical treatment of lung adenocarcinoma.

## Introduction

The origin of tumor initiation remains one of the most important questions in modern cancer biology. The disruption of epigenetic changes, including DNA methylation, chromatin modifications, nucleosome positioning, and alterations in noncoding RNA profiles, may cause the alteration of gene function and cellular neoplastic transformation.^1–3^ Polycomb group (PcG) proteins are known as master transcriptional repressors, containing PRC1 and PRC2 complexes, and modify chromatin to control multiple physiological processes including cell senescence, cell apoptosis, cell proliferation, and cell differentiation.^4–6^ Chromobox (CBX) family proteins are the core components of PRC1, which has been proved to be a transcription suppressor and play an important role in organ development and tumorigenesis.^7,8^ There are eight members of CBX proteins family which can be further divided according to their molecular structures into HP1 group (formed by N-terminal and C-terminal chromodomains, contains CBX1, 3, 5) and Pc group (only n-terminal chromodomain, contains CBX2, 4, 6, 7, 8).^9^ CBX proteins are involved in multiple types of tumor progression.^10–12^

CBX4 has been proved to participate in several processes including growth, senescence, immunity, and tissue repair.^7^ Through the combination of Polycomb- and SUMO E3 ligase-dependent functions via N-terminal chromodomain and two SUMO-interacting motifs (SIM), CBX4 can regulate some cellular processes.^13,14^ CBX4 maintains nucleolar homeostasis and regulates the expression of certain genes to resist cell senescence.^15,16^ During cell differentiation, CBX4 interacts with P63 transcription factors to play a regulatory role.^17^ Besides, CBX4 also contributes to HIV-1 latency through forming phase separation.^18^ Furthermore, CBX4 has been studied extensively in multiple types of cancer.^19–21^ Previous studies have proved that CBX4 serves as a vital regulator for thymic epithelial generation and maintenance as well as the development of thymocyte.^22^ CBX4 has been demonstrated to promote metastasize and progression in lung cancer through regulating BMI-1.^23^ Overexpression of CBX4 recruits GCN5 to sustain H3K27Ac of Runx2 promoter, up-regulates its transcription and promotes lung metastasis in osteosarcoma subsequently.^19^ CBX4 is also found to promote breast cancer via miR-137 via Notch1 signaling, and to be stimulative to tumorigenesis in lung adenocarcinoma (LUAD) possibly through Wnt/β-catenin pathway.^20,21^

RAS is a membrane protein with GTPase activity and can transmit signals from cell membrane to nucleus in activated state, which regulates cell growth and differentiation.^24^ Oncogenic mutations including Kras^G12D^ are frequently observed in ~20% of all types of human cancers including carcinomas of the lung, colon, and pancreas.^25^ Kras mutation is a common mutation type in variety kinds of tumors, therefore it is often used as a tumor model in the studies of lung cancer.^26–28^Previous researches of targeting Kras strategy were nonspecific and inefficient, but in recent years a variety of new strategies have been developed to target Kras^G12D^, suggesting a promising future.^29–31^

We used the LSL-Kras^G12D^ activation induced lung tumor mice model in this study and found that CBX4 deletion promoted lung tumor progression in Kras^G12D^ background. Further, our in vitro and RNA-Seq data showed that CBX4 loss caused instability of chromosome and most cells tended to apoptosis in the initial stage. Then minority of cells survived from genomic instability due to several changes of signaling pathway including Hippo pathway and gained stronger proliferation and invasion ability, thereby promoting tumorigenesis. It reminds that the function of genes may be related to the contexts, pressure, and identity of tumors, which should be considered when developing the target therapies. In summary, we tended to investigate the role of CBX4, a key component of PRC1 complex, in lung adenocarcinoma tumorigenesis under Kras^G12D^ background with Cre-induced *LSL-Kras*^*G12D*^ mouse model.

## Results

### CBX4 deletion promotes tumorigenesis in Kras^G12D^ mice model

CBX4 is a component of the Polycomb group multiprotein and has been proved to participate in cell growth, senescence, and other processes else. It has also been studied in a variety of cancer species and its effect on tumor production is tissue heterogeneous.^15–21^ As mere CBX4 deficient does not induce LUAD (Supplementary Fig. 1), we chose *Kras*^*G12D*^ mouse model for this study, widely used in lung cancer studies, and Adeno-Cre could be delivered via nasal inhalation to initiate Kras^G12D^-dependent lung cancer.^25^ We crossed *Cbx4*^*L/L*^ with *LSL-Kras*^*G12D*^ mice to get *Cbx4*^*L/L*^*, LSL-Kras*^*G12D*^ mice for subsequent experiments. Immunofluorescence with Lenti-Cre-GFP demonstrated more tumor-like *Kras*^*G12D*^ cell aggregations due to loss of CBX4 (Supplementary Fig. 2).

**Fig. 1:**
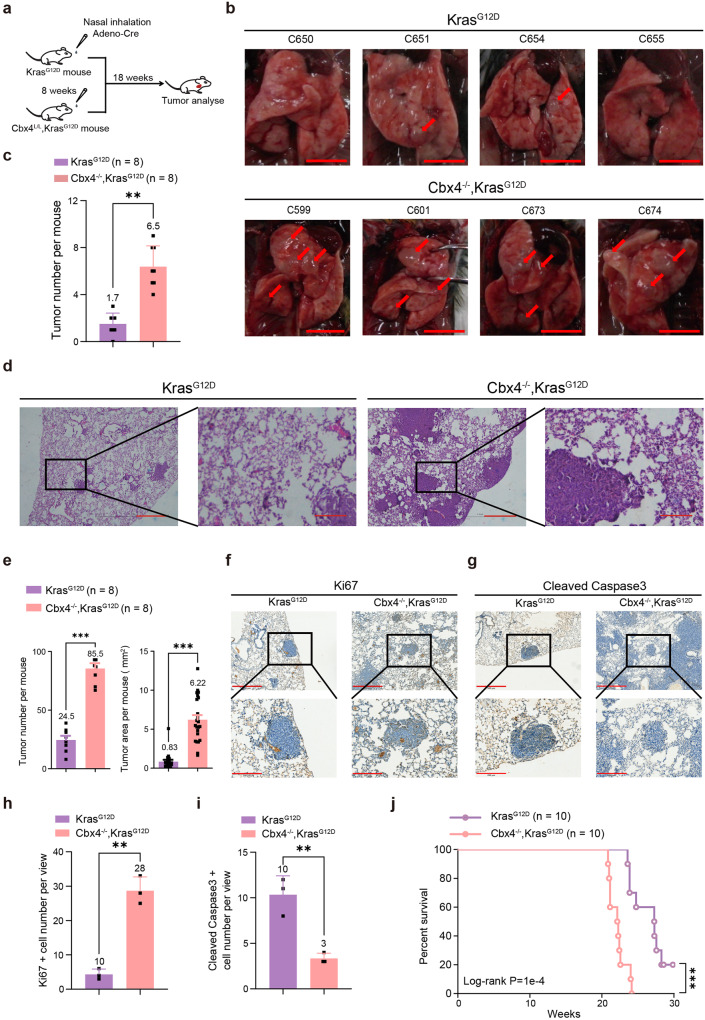
CBX4 deletion induces LUAD and causes a worse prognosis in *Kras*^*G12D*^ mice model. **a** A scheme for Adeno-Cre virus treatment for *Kras*^*G12D*^ and *Cbx4*^*−/−*^*, Kras*^*G12D*^ mouse model. **b** Representative photographs of lung tissue of *Kras*^*G12D*^ and *Cbx4*^*−/−*^*, Kras*^*G12D*^ mice after inhalation of Adeno-Cre for 18 weeks. Arrows indicate tumor lesions. Scale bar: 1 cm. **c** Tumor nodules’ number count of lung tissue of *Kras*^*G12D*^ (*n* = 8) and *Cbx4*^*−/−*^*, Kras*^*G12D*^ mice (*n* = 8) after inhalation of Adeno-Cre for 18 weeks. **d** Representative H&E staining in lung sections from *Kras*^*G12D*^ and *Cbx4*^*−/−*^*, Kras*^*G12D*^ mice. Scale bar: 100 μm (left), 10 μm (right). **e** Tumor nodules’ number count and tumor area data of lung tissue from *Kras*^*G12D*^ and *Cbx4*^*−/−*^*, Kras*^*G12D*^ mice after inhalation of Adeno-Cre for 18 weeks. **f** Representative immunohistochemical staining of Ki67 in lung sections from *Kras*^*G12D*^ and *Cbx4*^*−/−*^*, Kras*^*G12D*^ mice. Scale bar: 500 μm (up), 200 μm (down). **g** Representative immunohistochemical staining of cleaved-Caspase3 in lung sections from *Kras*^*G12D*^ and *Cbx4*^*−/−*^*, Kras*^*G12D*^ mice. Scale bar: 500 μm (up), 200 μm (down). **h** Quantitative analysis of Ki67 positive cell number in IHC staining of lung sections from *Kras*^*G12D*^ and *Cbx4*^*−/−*^*, Kras*^*G12D*^ mice. **i** Quantitative analysis of cleaved Caspase3 positive cell number in IHC staining of lung sections from *Kras*^*G12D*^ and *Cbx4*^*−/−*^*, Kras*^*G12D*^ mice. **j** Kaplan–Meier survival curves of *Kras*^*G12D*^ mice group (*n* = 10) and *Cbx4*^*−/−*^*, Kras*^*G12D*^ mice group (*n* = 10) after Adeno-Cre treatment. Data are shown as means ± SEM. **p < 0.01, ***p < 0.001

Next, *Kras*^*G12D*^ and *Cbx4*^*L/L*^*, LSL-Kras*^*G12D*^ mice were administrated with Adeno-Cre through nasal inhalation to induce LUAD (Fig. 1a and Supplementary Fig. 3). We observed more tumor nodules on the surface of lungs in *Cbx4*^*−/−*^*, Kras*^*G12D*^ mice and H&E staining showed significantly larger tumor size in *Cbx4*^*−/−*^*, Kras*^*G12D*^ mice (Fig. 1b–e). Immunohistochemical staining (IHC) of Ki67 indicated that *Cbx4*^*−/−*^*, Kras*^*G12D*^ tumors were more proliferative than *Kras*^*G12D*^ group. The result of cleaved-Caspase3 and -PARP1 manifested less cell apoptosis in *Cbx4*^*−/−*^*, Kras*^*G12D*^ mice (Fig. 1f–i and Supplementary Fig. 4). In addition, the *Cbx4*^*−/−*^*, Kras*^*G12D*^ mice succumbed sooner than the *Kras*^*G12D*^ mice (Fig. 1j). Taken together, these results prove that loss of CBX4 promotes tumorigenesis and suggests a worse prognosis in Kras^G12D^ background.

### CBX4 deletion promotes cell proliferation and invasion in long-term cell culture

Then we sought to explore the mechanism through in vitro experiments with Mouse Embryonic Fibroblast (MEF). Fluorescence Activated Cell Sorting (FACS) analysis presented apoptotic cells were over 50% in primary *Cbx4*^*−/−*^*, Kras*^*G12D*^ cells (Supplementary Fig. 5a, b). 3D cell culture suggested that even partial loss of CBX4 impeded cell proliferation and invasion (Supplementary Figs. 5c and 6). β-gal staining showed that deletion of CBX4 induced cell senescence during initial cell growth phase (Supplementary Fig. 5d).

**Fig. 2:**
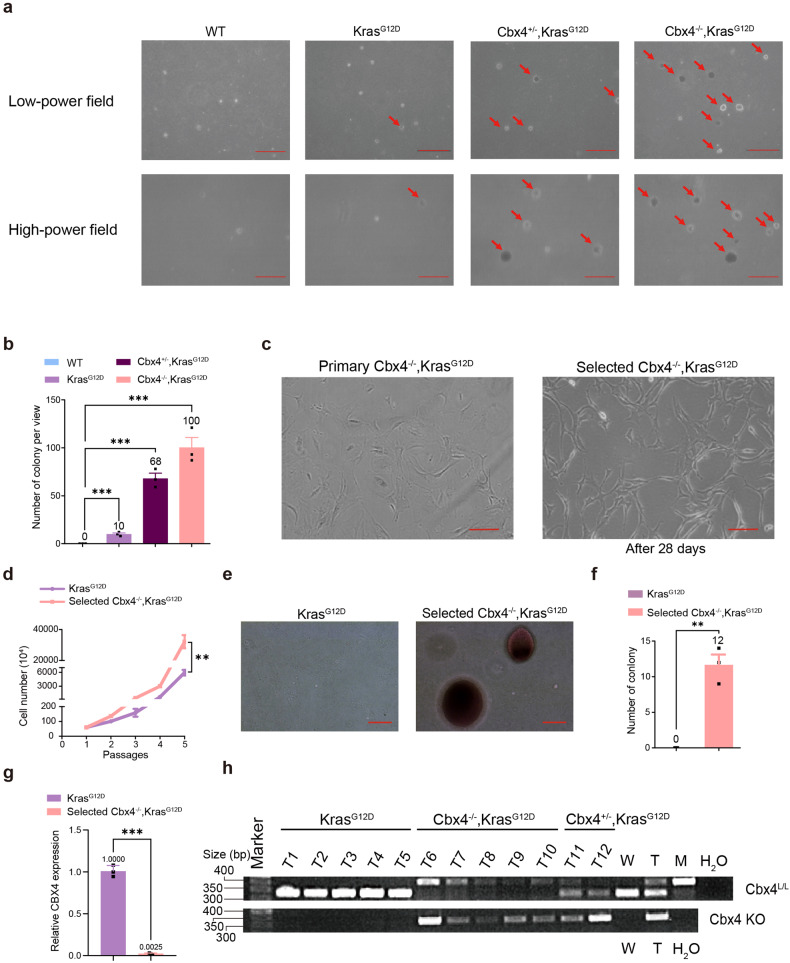
CBX4 deletion eventually promotes cell proliferation and invasion in long-term cell culture in *Kras*^*G12D*^ background. **a** Colony formation ability of *Wild-type, Kras*^*G12D*^, *Cbx4*^*+/−*^*, Kras*^*G12D*^ and *Cbx4*^*−/−*^*, Kras*^*G12D*^ MEFs was observed by soft agar assay under low and high field of view. Arrows indicate clones. Scale bar: 200 μm (up) and 100 μm (down). **b** Quantitative analysis of colony formation ability of *Wild-type, Kras*^*G12D*^, *Cbx4*^*+/−*^*, Kras*^*G12D*^ and *Cbx4*^*−/−*^*, Kras*^*G12D*^ ME. **c** During cell culture of *Cbx4*^*−/−*^*, Kras*^*G12D*^ MEFs, minority of the cells stayed alive after 28 days and were selected to culture separately. Scale bar: 200 μm. **d** Cell proliferation ability of selected *Cbx4*^*−/−*^*, Kras*^*G12D*^ MEFs compared with *Kras*^*G12D*^ by 3T3 proliferation assay. **e**, **f** Microscopic observation and clones count showed cell transformation ability of the selected *Cbx4*^*−/−*^*, Kras*^*G12D*^ MEFs. Scale bar: 50 μm. *Kras*^*G12D*^ cells were used as control. **g** CBX4 expression in *Kras*^*G12D*^ and selected *Cbx4*^*−/−*^*, Kras*^*G12D*^ MEFs. **h** Genotype of *Kras*^*G12D*^, *Cbx4*^*−/−*^*, Kras*^*G12D*^ and *Cbx4*^*+/−*^*, Kras*^*G12D*^ mice. Wild-type (W), heterozygote (T) and mutant (M) type were used for control. Data are shown as means ± SEM. **p < 0.01, ***p < 0.001

However, 4 weeks soft agar assay indicated dramatic increase in colony formation and cell transformation abilities in *Cbx4*^*−/−*^*, Kras*^*G12D*^ group (Fig. 2a, b). We further extended the 2D culture to 4 weeks, and in consistence with the soft agar assay results, we found that a small portion of *Cbx4*^*−/−*^*, Kras*^*G12D*^ MEFs survived in long-term (28 days) culture, which we called the selected cells (Fig. 2c, d). To ensure the authenticity of the result, we performed the Cre-GFP cell tracing experiments in *Cbx4*^*L/L*^*, LSL-Kras*^*G12D*^ MEFs and found that majority of Cre-GFP positive (*Cbx4*^*−/−*^*, Kras*^*G12D*^) cells underwent apoptosis, while a small amount of Cre-GFP positive cells survived and divided (Supplementary Fig. 7). Further experiments showed that selected cells acquired stronger abilities in cell proliferation and transformation compared with *Kras*^*G12D*^ cells (Fig. 2e, f). RT-qPCR and genotype of lung tissues confirmed that these selected cells and the lung tumors were indeed CBX4-deleted (Fig. 2g, h). These data unravel that a minuscule part of *Cbx4*^*−/−*^*, Kras*^*G12D*^ cells acquire stronger proliferation and transformation abilities during the long-term culture, which accounts for the increased tumorigenesis in *Cbx4*^*−/−*^*, Kras*^*G12D*^ mice.

### Typical selected Cbx4^*−/−*^, Kras^G12D^ clones gain cell proliferation and clone formation abilities in vitro

**Fig. 3:**
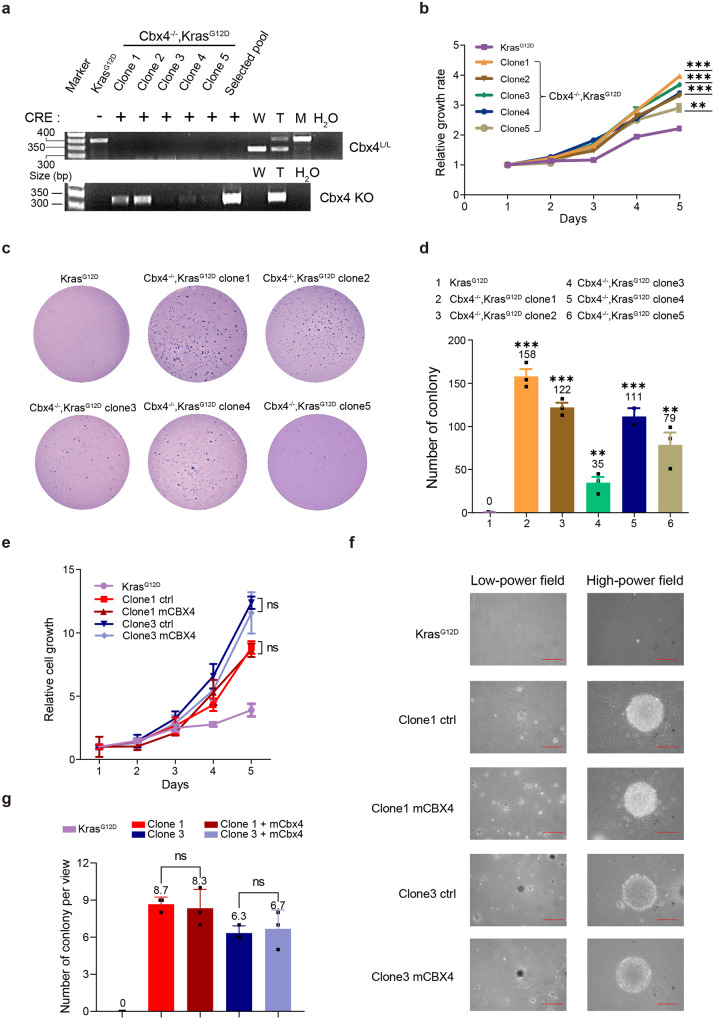
Typical selected *Cbx4*^*−/−*^*, Kras*^*G12D*^ clones gain cell proliferation and clone formation abilities in vitro which cannot be rescued by ectopic CBX4. **a** Genotype (with or without Cre recombinase) of selected *Cbx4*^*−/−*^*, Kras*^*G12D*^ clones. Wild-type (W), heterozygote (T) and mutant (M) type were used as control. **b** MTT assay of 5 selected *Cbx4*^*−/−*^*, Kras*^*G12D*^ clones and the control (*Kras*^*G12D*^ MEFs). **c**, **d** Colony formation and transformation abilities of five selected *Cbx4*^*−/−*^*, Kras*^*G12D*^ clones were shown by soft agar assay and the quantitative analysis. **e** MTT analysis to show cell proliferation with or without ectopic expression of CBX4 with the control of *Kras*^*G12D*^ cells. **f**, **g** Colony formation ability of selected *Cbx4*^*−/−*^*, Kras*^*G12D*^ cells (clone1 and clone3 representatively) with or without ectopic expression of CBX4 is determined by soft agar assay compared with the control of *Kras*^*G12D*^ MEFs. Scale bar: 200 μm (left) and 50 μm (right). Data are shown as means ± SEM. Ns *p* > 0.05, **p < 0.01, ***p < 0.001

Next, to further validate the cell proliferation and clone formation abilities of selected *Cbx4*^*−/−*^*, Kras*^*G12D*^ cells in vitro, we selected 5 typical single clones from the selected pool for further experiments. Genotype result confirmed that those clones were Cbx4 deficient (Fig. 3a). MTT assay presented that all 5 *Cbx4*^*−/−*^*, Kras*^*G12D*^ clones exhibited faster growth rates compared with *Kras*^*G12D*^ MEFs control (Fig. 3b). FACS analysis revealed a similar result by the larger percentage of proliferating cells in every selected *Cbx4*^*−/−*^*, Kras*^*G12D*^ clone (Supplementary Fig. 8). Compared with the *Kras*^*G12D*^ MEFs, *Cbx4*^*−/−*^*, Kras*^*G12D*^ clones in soft agar yielded greater colony numbers, indicating they got promotion in vitro transformation (Fig. 3c, d).

To notarize whether these alterations in the *Cbx4*^*−/−*^*, Kras*^*G12D*^ were caused by loss of environmental CBX4 or irreparable genome change, we over-expressed mCBX4 in two *Cbx4*^*−/−*^*, Kras*^*G12D*^ clones. Real-time PCR and WB confirmed the ectopic expression of mCBX4 in these two clones (Supplementary Fig. 9). However, it was indicated that cell growth couldn’t be restored by CBX4 re-introduction. So ectopic CBX4 expression was irrelevant to cell proliferation in selected *Cbx4*^*−/−*^*, Kras*^*G12D*^ clones (Fig. 3e). Colony formation assays also demonstrated that CBX4 had no reverse on the phenotype of these clones (Fig. 3f, g). This means that for selected *Cbx4*^*−/−*^*, Kras*^*G12D*^ cells, the changes in proliferation and invasion are irreversible cellular changes, rather than conditionally correlated with CBX4 expression, probably due to occurrences of oncogenic mutations and signals during the selection.

### CBX4 loss induces genome instability in selected Cbx4^*−/−*^, Kras^G12D^ MEFs

**Fig. 4:**
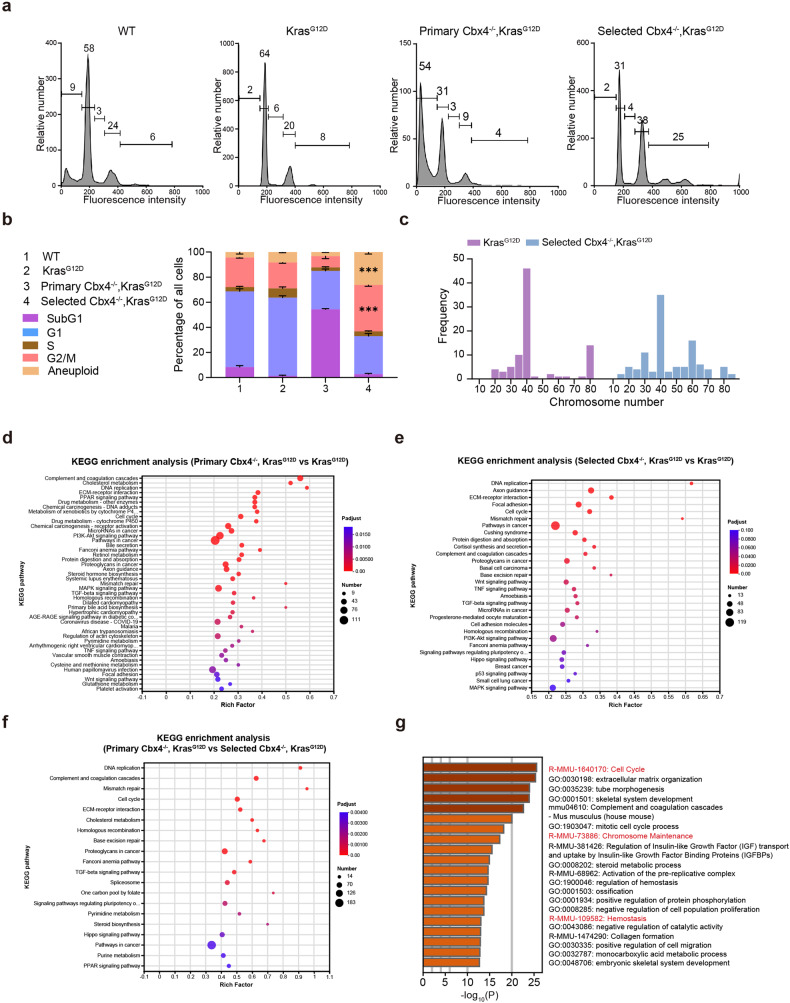
Genomic instability emerges in selected *Cbx4*^*−/−*^*, Kras*^*G12D*^ MEFs compared with *Kras*^*G12D*^ and primary *Cbx4*^*−/−*^*, Kras*^*G12D*^ MEFs. **a** FACS Analysis of *Wild-Type*, *Kras*^*G12D*^, primary *Cbx4*^*−/−*^*, Kras*^*G12D*^ and selected *Cbx4*^*−/−*^*, Kras*^*G12D*^ MEFs of cell proportion at each stage. **b** Quantitative analysis of *Wild-Type*, *Kras*^*G12D*^, primary *Cbx4*^*−/−*^*, Kras*^*G12D*^ and selected *Cbx4*^*−/−*^*, Kras*^*G12D*^ MEFs of cell proportion at each stage. **c** Karyotype analysis of *Kras*^*G12D*^ and selected *Cbx4*^*−/−*^*, Kras*^*G12D*^ MEFs. **d** KEGG enrichment analysis of primary *Cbx4*^*−/−*^*, Kras*^*G12D*^ MEFs compared with *Kras*^*G12D*^ MEFs. Several cell pathways are found altered including Wnt signaling pathway. **e** KEGG enrichment analysis of selected *Cbx4*^*−/−*^*, Kras*^*G12D*^ MEFs compared with *Kras*^*G12D*^ MEFs. Several pathways are found altered such as Hippo, PI3K and Wnt. **f** KEGG enrichment analysis of primary *Cbx4*^*−/−*^*, Kras*^*G12D*^ MEFs compared with selected *Cbx4*^*−/−*^*, Kras*^*G12D*^ MEFs. Several cell function modules are found altered like DNA replication, pathways in cancer and Hippo signaling pathway. **g** GO enrichment analysis between primary *Cbx4*^*−/−*^*, Kras*^*G12D*^ and *Kras*^*G12D*^ MEFs. Data are shown as means ± SEM. ***p < 0.001

Therefore, how those survival selected cells become primary tumor cells with the ability to proliferate and invade after the long-term culture without CBX4 should be considered. FACS analysis further confirmed that selected *Cbx4*^*−/−*^*, Kras*^*G12D*^ cells were more proliferative (more cells in G2/M and Aneuploid period) (Fig. 4a, b). Chromatin alterations can regulate gene expression and epigenetic changes in vivo, including cancer initiation and progression.^32^ The result of Karyotype analysis presented the proportion of aneuploidy of selected *Cbx4*^*−/−*^*, Kras*^*G12D*^ cells (62%) was significantly higher than *Kras*^*G12D*^ counterpart (32%), implying the unstable genomic condition after long-time culture with CBX4 knockout (Fig. 4c).

We performed Bulk RNA Sequencing to further decipher the differences between the primary *Cbx4*^*−/−*^*, Kras*^*G12D*^ cells, the selected *Cbx4*^*−/−*^*, Kras*^*G12D*^ cells and the *Kras*^*G12D*^ cells. Gene Ontology (GO) Analysis revealed thousands of genes were significantly altered in primary and selected *Cbx4*^*−/−*^*, Kras*^*G12D*^ cells compared with *Kras*^*G12D*^ cells (Fig. 4d–f and Supplementary Table 1). Further, RNA-seq data revealed significant changes in cell cycle, chromosome maintenance and hemostasis in primary *Cbx4*^*−/−*^*, Kras*^*G12D*^ cells (Fig. 4g), implying genomic instability, which was suggested by Karyotype analysis (Fig. 4c). Totally, the result shows that CBX4 loss causes chromosome instability in Kras^G12D^ background.

### P15 and P16 are upregulating in primary Cbx4^*−/−*^, Kras^G12D^ cells causing cell apoptosis

**Fig. 5:**
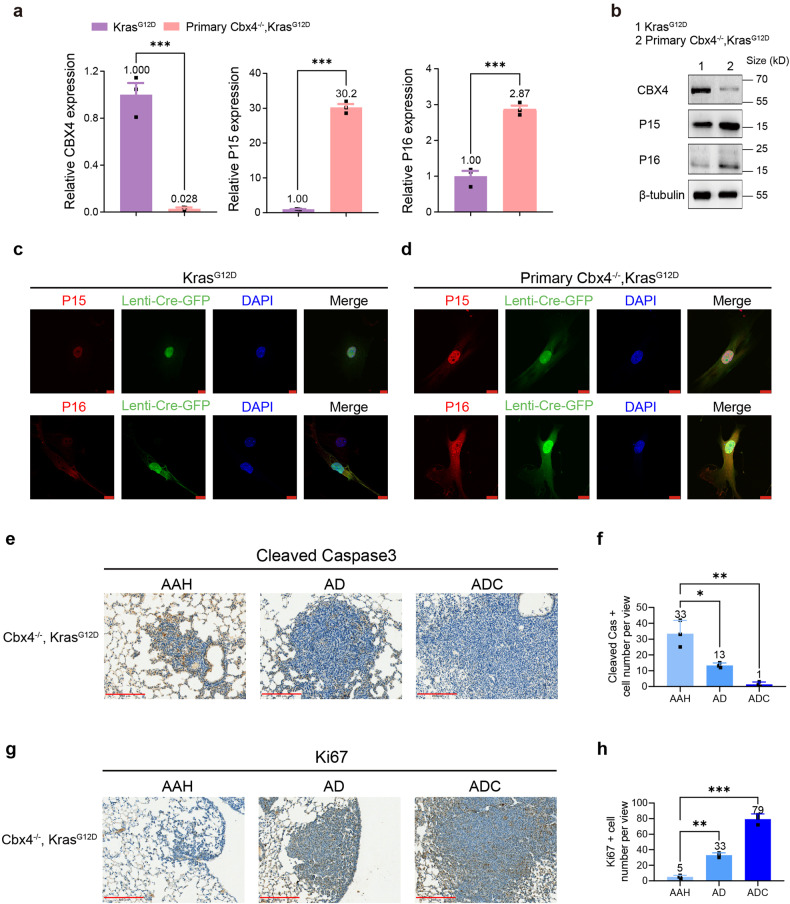
CBX4 loss up-regulates P15, P16 and causes alterations of apoptosis-related genes in primary *Cbx4*^*−/−*^*, Kras*^*G12D*^ cells. **a** Real-time PCR analysis of CBX4, P15 and P16 in *Kras*^*G12D*^ and primary *Cbx4*^*−/−*^*, Kras*^*G12D*^ MEFs. **b** Western Blot of CBX4, P15 and P16 in *Kras*^*G12D*^ and primary *Cbx4*^*−/−*^*, Kras*^*G12D*^ MEFs. **c** IF staining of P15 (red, up), P16 (red, down), Lenti-Cre-GFP (green) and DAPI (blue) in *Kras*^*G12D*^ cells. Scale bar: 10 μm. **d** IF staining of P15 (red, up), P16 (red, down), Lenti-Cre-GFP (green) and DAPI (blue) in primary *Cbx4*^*−/−*^*, Kras*^*G12D*^ cells. Scale bar: 10 μm. **e** Representative immunohistochemical staining of cleaved Caspase3 in lung sections from *Cbx4*^*−/−*^*, Kras*^*G12D*^ mice. Scale bar: 200 μm. **f** Quantitative analysis of cleaved Caspase3 positive cell number in IHC staining of lung sections of AAH, AD and ADC from *Cbx4*^*−/−*^*, Kras*^*G12D*^ mice. **g** Representative immunohistochemical staining of Ki67 in lung sections from *Cbx4*^*−/−*^*, Kras*^*G12D*^ mice. Scale bar: 200 μm. **h** Quantitative analysis of Ki67 positive cell number in IHC staining of lung sections of AAH, AD and ADC from *Cbx4*^*−/−*^*, Kras*^*G12D*^ mice. Data are shown as means ± SEM. *p < 0.05, **p < 0.01, ***p < 0.001

According to the results above, we hypothesized that the loss of CBX4 regulated the expression of some apoptosis-related genes (also tumorigenesis-related genes) during the initial cell culture process. P16 has been proved as an activating factor of tumor suppressor pathways and inactivated in many different human tumor types.^33^ To understand the above phenomenon in detail, we first performed QPCR and found that expressions of P15 and P16 were upregulated in primary *Cbx4*^*−/−*^*, Kras*^*G12D*^ cells. WB and IF also supported this observation (Fig. 5a–d), which suggested that absence of CBX4 promoted cell apoptosis in early culture by upregulating the expression of tumor suppressor genes. Moreover, we found that apoptosis-related genes were highly expressed in atypical adenomatous hyperplasia (AAH, the initial stage of LUAD), then decreased during tumor progression (Fig. 5e, f and Supplementary Fig. 10), whereas the expression of Ki67 was in the opposite trend (Fig. 5g, h).

These results provide evidence for a predominant apoptotic trend at the initial stage of CBX4 deletion and then a promotion of tumorigenesis as the few surviving cells acquires greater proliferative and invasive abilities.

### Multiple pathways involved in tumorigenesis including Hippo are changed in selected Cbx4^*−/−*^, Kras^G12D^ cells

**Fig. 6:**
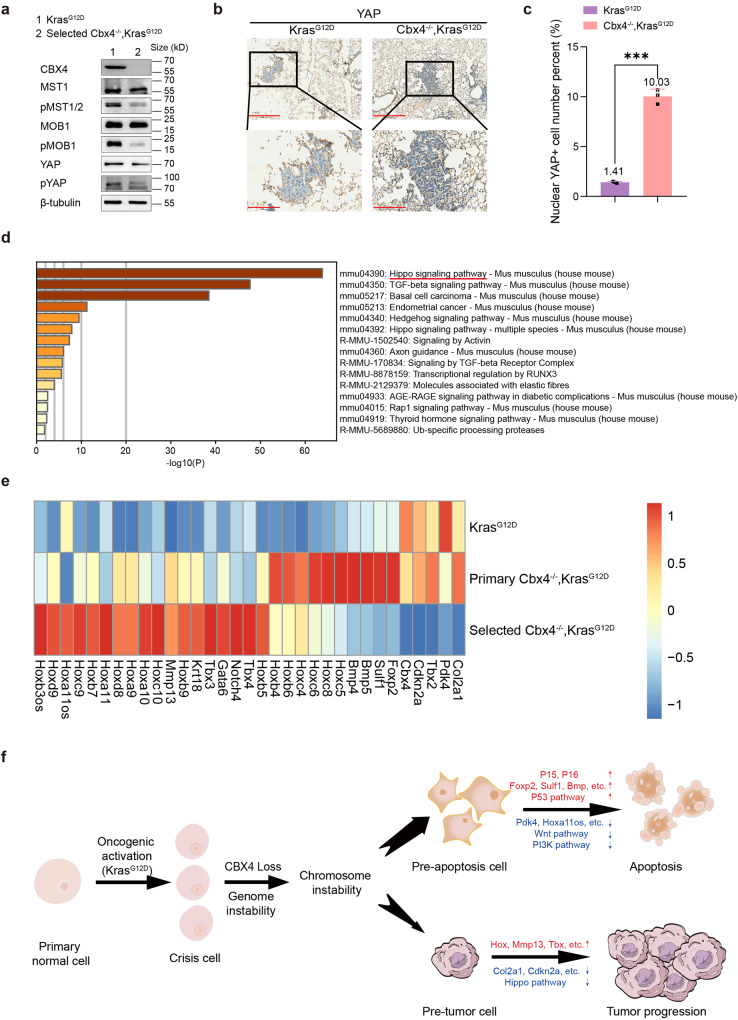
CBX4 deletion influences multiple signaling pathways including Hippo pathway and ultimately promotes Kras^G12D^ tumorigenesis. **a** Western Blot of CBX4, MST1, pMST1/2, MOB1, pMOB1, YAP, pYAP and β-tubulin in *Kras*^*G12D*^ and selected *Cbx4*^*−/−*^*, Kras*^*G12D*^ MEFs. **b** Representative immunohistochemical staining of YAP in lung sections from *Kras*^*G12D*^ and *Cbx4*^*−/−*^*, Kras*^*G12D*^ mice. Scale bar: 500 μm (up), 200 μm (down). **c** Quantitative analysis of nuclear YAP positive cell percent in IHC staining of lung sections from *Kras*^*G12D*^ and *Cbx4*^*−/−*^*, Kras*^*G12D*^ mice. **d** GO enrichment analysis focused Hippo signaling pathway based on independent analysis. **e** Expression condition of several representative genes in selected *Cbx4*^*−/−*^*, Kras*^*G12D*^ cells compared with *Kras*^*G12D*^ and primary *Cbx4*^*−/−*^*, Kras*^*G12D*^ groups. Upper one/row is *Kras*^*G12D*^, the middle one/row is primary *Cbx4*^*−/−*^*, Kras*^*G12D*^ group, the lower one/row is selected *Cbx4*^*−/−*^*, Kras*^*G12D*^ group. Red indicates more expression and blue means less expression. **f** Mechanism of CBX4 deletion in promoting tumorigenesis with Kras^G12D^ mutation. At the early stage of cell culture, P15, P16 and other apoptosis-related genes are upregulated in the *Cbx4*^*−/−*^*, Kras*^*G12D*^ cells due to chromosome instability, which lead to a large population of cell apoptosis. At the late stage of cell culture, a small population of cells survive from genomic instability acquiring stronger abilities in proliferation and transformation called “selected *Cbx4*^*−/−*^*, Kras*^*G12D*^”, due to changes of multiple pathways including inactivation of Hippo pathway, which ultimately result in tumorigenesis. Data are shown as means ± SEM. **p < 0.01, ***p < 0.001

Tumor initiation can be launched by epigenetic regulation, which participates in various processes of cells. There are often genomic changes that occur during cell apoptosis, and studies have shown that cytogenetic genome instability may inhibit programmed apoptosis through a variety of ways, so we took that into account.^34^ In addition, multiple pathways including Hippo were found to be altered in selected *Cbx4*^*−/−*^*, Kras*^*G12D*^ cells (Fig. 4d–f). The Hippo pathway has also been widely studied in lung cancer, and its activation can inhibit the occurrence and development of lung cancer.^35,36^ Abnormalities in signaling pathways lead to alteration of epigenetic modulators and unsubstantiated gene expression, which may result in tumorigenesis. Western blot showed that phosphorylated MST, MOB and YAP were decreased, and total YAP was almost unchanged, which meant Hippo signal pathway was turn-off in selected *Cbx4*^*−/−*^*, Kras*^*G12D*^ MEFs (Fig. 6a). YAP fusion has been shown to exclude the PRC2 complex in ependymoma,^37^ therefore in our study suggest CBX4 is likely to have a moderating effect on Hippo signaling pathway as well. In our study, the YAP nuclear localization increased in the *Cbx4*^*−/−*^*, Kras*^*G12D*^ mice lung tumors compared with the *Kras*^*G12D*^ mice (Fig. 6b, c). At the same time, we also noticed basal cell cancer-related signatures in selected *Cbx4*^*−/−*^*, Kras*^*G12D*^ MEFs (Fig. 6d), and we speculated that cell identity may have changed. It is a conceivable direction for later mechanism research.

We also noticed 18 Hox genes, Mmp13, Tbx, Pdk4, Bmp and other proven tumor-related genes in the list. Alterations in the expression of these genes can also promote tumorigenesis in different aspects^38–43^ (Fig. 6e). We performed QPCR to assess the expression of the other members of the PcG group and found that PHC1 and CBX2 were significantly upregulated in the selected *Cbx4*^*−/−*^*, Kras*^*G12D*^ MEFs (Supplementary Fig. 11). MTT and soft agar assays further confirmed overexpression of mPHC1 and mCBX2 promoted cell proliferation and transformation in selected *Cbx4*^*−/−*^*, Kras*^*G12D*^ cells, though knockdown of these two had the opposite effect (Supplementary Figs. 12 and 13).

In general, we believe that when CBX4 is deficient, most cells with Kras^G12D^ mutation undergo apoptosis for chromosome instability in the initial stage, but a small population of cells survive from genomic instability acquiring stronger abilities in proliferation and transformation, due to changes of multiple pathways including Hippo (Fig. 6f). Our study provides a possible mechanism of lung cancers with low CBX4 level and suggests new clues for CBX4-targeted therapies in cancer treatment.

## Discussion

Previous studies have shown that CBX4 functions as a proto-oncogene during tumorigenesis.^21^ In this study, we performed CBX4 IHC staining on the previous tumor samples,^21^ and found that a subset of LUAD patients had high CBX4 expression but there were also a significant number of LUAD cases with low CBX4 expression, leading to a worse prognosis under the background of Kras mutation, restricted to stage III (Supplementary Fig. 14). This indicated that the study was clinically instructive under certain condition. We found that most cells got apoptosis due to instability of chromosome in the initial stage caused by CBX4 loss, but minority of cells survived and improved the ability of proliferation and transformation, promoting lung tumors under Kras^G12D^ activation background. This dichotomy effect can also be seen in other CBX family members, such as CBX7, which is known to suppress cervical cancer, hepatocellular carcinoma and lung cancer,^44–46^ but the study also shows it could inhibit *P16* and activate AKT-NF-κB-miR-21 pathway to promote cell survival and stem cell-like characteristics in gastric cancer.^47^

We found that CBX4 loss triggered cell apoptosis at the early stage of the Kras^G12D^ MEFs by chromosome instability, possibly through dysregulation of *P15* and *P16*. Cell apoptosis is a form of programmed cell death that orderly and efficiently remove the damaged cells, which can be regarded as a self-contained mechanism against tumors.^48^ The expression of *P16* mediates cell apoptosis, and therefore represses tumors in the early stage of cell culture. There’s evidence that PRC1 has crucial impact on the initiation of cellular senescence by combining with the promoter of *P16* and mediating the transcription.^49^ Studies have manifested that ectopic expression of some components of PRC1 will suppress the expression of *P16*,^33^ which hints at a possible mechanism of *P16*’s up-regulation.

PcG proteins, as a known epigenetic transcriptional regulator, can recruit different components to form different PRC complexes through histone modification.^5,50,51^ Studies on the roles of these complexes in different tissues show different effects.^52^ As a model proposed in Drosophila from previous studies, PRC complexes induce chromatin compaction leading to gene silencing.^7,53^ However, the transcription factors involved in this process are not conserved in mammalian cells, so there are still no in-depth research results in related fields.

An important part of this study is the discovery of “tumorization” changes through genome instability in the survival cells of CBX4-related apoptosis described above. Our study finds CBX4 deletion promotes the genomic instability by karyotype analysis. Then 249 genetic mutations are identified in *Cbx4*^*−/−*^*, Kras*^*G12D*^ cells altogether via bioinformatics analysis (Supplementary Table 1). We notice several Hippo pathway related genes and find Hippo pathway is repressed in *Cbx4*^*−/−*^*, Kras*^*G12D*^ cells through WB. At the same time, we notice Wnt signaling pathway appear into the list. Previous articles also indicate that Wnt signaling pathway is involved in the regulation of CBX4 on LUAD formation.^21^ CBX4 has been shown associated with Wnt signaling pathway in lung cancer, and many other studies have reported that Wnt pathway activation can promote lung cancer by promoting angiogenesis and the expression of related proteins.^54,55^ The Hippo pathway has also been widely studied in lung cancer, and its activation can inhibit the occurrence and development of lung cancer.^35,36^ The interaction between CBX4 and these signaling pathways can be a probable direction for later research of mechanism. Though, the exact regulatory mechanism remains to be elucidated.

Our results so far point to the chromosomal instability caused by CBX4 deletion and the selection of MEFs in the Kras^G12D^ background, causing majority of cells to undergo apoptosis, and the survival selected cells will acquire enhanced proliferation and invasion ability, and trend toward tumor formation. If further studies can identify the pathway by which CBX4 affects genomic instability or how the altered genes or signaling pathways affect tumor formation, targeting which will be the clues for LUAD treatment.

In summary, we have shown that loss of CBX4 (as a component of PRC1) influences the chromosome stability, causing cell apoptosis mostly in the initial stage by regulating some apoptosis-related genes’ expression under Kras^G12D^ mutation background. CBX4 loss also alter a variety of tumor-associated genes and signaling pathways including Hippo pathway in the survival selected cells, finally inducing tumorigenesis. Our study can provide new clues for future studies on relative targeted therapies.

## Methods and materials

### Mouse cohorts and treatment

*LSL-Kras*^*G12D*^ mouse were generous gifts from labs of Kwok-Kin Wong. *CBX4*^*L/L*^ mice were generated by Guoliang Xu lab. All mice were cultivated at Fudan University in the environment of pathogen-free and were treated by protocols granted by the Department of Laboratory Animal Science Fudan University. All mice’ genotype was done at the age of 8 ~ 10 days using the following primers (Supplementary Table 2). All *LSL-Kras*^*G12D*^ and *Cbx4*^*L/L*^*, LSL-Kras*^*G12D*^ mice were treated with 2 × 10^6^ PFU Adeno-Cre or Lenti-Cre-GFP via nasal inhalation at the age of 8 weeks.^21^ Gross inspection and pathologic examination were conducted accordingly. Analyses of lung tumors of all cultured mice after the dissection were performed. The lung tissue of mice was inflated and fixed in 4% formalin, paraffin embedded, sectioned, and stained with hematoxylin and eosin (H&E) afterwards. Tumor numbers and sizes were analyzed using stained consecutive sections via microscopy and quantitated with ImageJ software.

### Cell culture and viral infection

Mouse embryonic fibroblasts (MEFs) were isolated from 13.5 postcoitum embryos and cultivated in DMEM medium (Thermo Fisher, 11995065) with 10% fetal bovine serum (FBS), 100 mg/ml penicillin and 100 μg/ml streptomycin. Cells were applied for functional assays after in vitro culture for at least 2 more passages. HEK-293T and A549 cells were cultured in DMEM medium with 10% FBS. Mouse CBX4 (mCBX4) were cloned into pCDH-CMV-MCS-EF1 vector for overexpression. Lentivirus based shRNAs targeting mCbx4 were cloned using MLP and pLKO.1 vectors,^21^ respectively. Lentiviral was packaged in HEK-293T cells and MEFs were infected by the viruses after filtering. The sequences of the PHC1 and CBX2-specific siRNAs are as follows:

siPHC1#1: 5’-UAAACUCGUAGACCUCCUCTT-3’,

siPHC1#2: 5’-UUUGUAGGAAGCACAGAGGTT-3’,

siCBX2#1: 5’-UCAAGUUGAAGAAGCCCACTT-3’,

siCBX2#2: 5’-UCCUCACUUUCAGGUCUAGTT-3’.

### Fluorescence activating cell sorter (FACS) assay

Virus-infected cells was collected at 80% confluency and fixed with 75% ethanol. Propidium iodine (PI) staining was conducted, and flow cytometry was applied for cell cycle analysis.

### 3-(4,5-Dimethylthiazol-2-yl)-2,5-diphenyltetrazolium bromide (MTT) assay

Virus-infected cells were plated in 96-well plate and cell viability were measured daily for 5 days. Twenty μl of MTT working solution (5 mg/ml) were added into each well and incubated for 4 h at 37 °C. Remove the supernatants and dissolve the resultant MTT formazan in 100 μl of dimethyl sulfoxide (DMSO) before reading. The absorbance at 570 and 630 nm were measured subsequently.

### 3T3 proliferation assay

To evaluate different MEFs’ cell proliferative ability, 8 × 10^4^ *Kras*^*G12D*^ and selected *Cbx4*^*−/−*^*, Kras*^*G12D*^ MEFs were seeded onto 6-well plates. The cells were split into new plates at suitable density every 3 days. Cell numbers were counted and with GraphPad Prism 4 Demo software.

### β-Gal staining of MEFs

*Wild-type*, *Kras*^*G12D*^, *Cbx4*^*+/-*^*, Kras*^*G12D*^ and *Cbx4*^*−/−*^*, Kras*^*G12D*^ MEFs were washed by 10 nM phosphate-buffered saline (PBS, Meilunbio, MA0010) and fixed with 0.2% glutaraldehyde for 5 min. Then the cells were washed 3 times in 5 min with PBS. MEFs were stained with X-gal solution (2 mM MgCl_2_, 5 mM K_3_Fe (CN)_6_, 5 mM K_4_Fe(CN)_6_, 0.01% Na Deoxycholate, 0.02% NP-40, PH to 7.4) for 12 h at 37 °C. X-gal solution was washed off with PBS before examining under a microscope.

### Three-dimensional (3D) cell culture

Matrigel (BD Biosciences) was thawed on ice at 4 °C overnight, and 24-well plates were pre-chilled at −20 °C overnight. Add 100 μl of Thawed Matrigel into each 24-well plate at 37 °C for 15 ~ 20 min to make it fully agglomerate. In total, 0.25% trypsin digested the MEFs, after centrifugation counting, the cells were suspended in the medium containing 2% Matrigel, the cell concentration was 2 × 10^4^/ml, adding 500 μl cell suspensions per well onto the coagulated Matrigel, the cells were incubated at 37 °C in 5% CO_2_ for 10 ~ 14 days, and the fresh medium containing 2% Matrigel was changed every 3 ~ 4 day. Photos were taken with a Leica light microscope.

### Soft agar assay

In total, 5000 ~ 10,000 virus-infected cells were re-suspended in culture medium with an additional 0.2% agar and layered them onto 1% agar beds in 6-well plates. The culture medium was changed every 3 days for 2 ~ 4 weeks, and the samples were subjected to 0.005% crystal violet staining. The numbers of colonies were quantitated by microscopic pictures.

### Real-time polymerase chain reaction (PCR) assay

RNA was extracted by Trizol (Invitrogen). First strand cDNA was generated using Revert Aid First Strand cDNA Synthesis Kit (Fermentas) following manufacturer’s instruction. Genomic DNA samples were extracted with Gentra Puregene Tissue Kit (Qiagen). cDNA was subjected to quantitative real-time PCR with gene-specific primers via the 7500 Fast Real-Time PCR System (Applied Biosystems) and the SYBR Green Master PCR mix (Invitrogen) (Supplementary Table 3). Beta actin was used as internal control.

### Western blot analysis

Total protein lysate was prepared by homogenization in protein loading buffer. Equal amounts of protein were separated by electrophoresis on an SDS-PAGE gel (SDS is from Abcone, L77480) and transferred onto PVDF membranes. Western blot analysis was performed using the following antibodies (Supplementary Table 4).

### RNA-seq data analysis

There were three MEFs (*Kras*^*G12D*^, primary *Cbx4*^*−/−*^*, Kras*^*G12D*^ MEFs and selected *Cbx4*^*−/−*^*, Kras*^*G12D*^ MEFs) collected for RNA-seq analysis. RNA-Seq (SE) read raw data obtained from HiSeq platform. FastQC was applied to evaluate the quality of sequencing. Raw reads were mapped by Tophat; the gene counts were conducted by htseq-count; and differentially expressed genes were selected by DESeq with FDR < 0.1.

### Immunofluorescence staining analysis

Cells were fixed in 4% formaldehyde in PBS buffer at room temperature for 10 min, washed with PBS, and permeabilized by PBST (PBS with 0.25% Triton X-100 (Abcone, X10010)). Cells and frozen sections were blocked for 30 min and incubated at 4 °C with primary antibody (Supplementary Table 5) overnight in PBSA (PBS and 3% BSA). After three times PBST washes at 15 min interval the samples were incubated with Alexa Fluor 552 conjugated goat anti-rabbit secondary antibody at a dilution of 1:1000 for 1 h at a room temperature. Nuclei were stained by DAPI. Samples were mounted with Aqua-Poly/Mount (Polysciences).

### Human LUAD samples analysis

A total of 72 pathologically confirmed human LUAD specimens and 7 normal lung specimens were collected in Fudan University Shanghai Cancer Center between January 2008 and December 2009 with written consents of patients and the approval from the Institute Research Ethics Committee. All tumor specimens were taken at the time of surgical resection. Patients were also divided into high and low CBX4 expression subgroups with its median expression value as the cut-off.^21^

### Statistical analysis

Data were presented as mean ± standard error unless otherwise indicated. Student’s *t* test or one-way analysis of variance (two-sided) was applied in multiple groups to determine the differences, and Tukey-Kramer multiple comparison test was applied for post hoc comparisons. Kaplan-Meier analysis with log-rank test was used to assess patients’ and mice’ survival between subgroups. GraphPad Prism 5 software was applied for all statistical analyses, and if *p* value < 0.05, it was statistically significant.

### Supplementary information

Supporting Information

## Data Availability

All data will be available upon reasonable request.
