# Supplementary material for: CBX4 deletion promotes tumorigenesis under KrasG12D background by inducing genomic instability
Source: Signal Transduct Target Ther. 2023 Sep 12;8:343. doi: 10.1038/s41392-023-01623-0 (PMC10495400; doi:10.1038/s41392-023-01623-0)
Supplement: Supplementary file 1 — Supporting Information [file 41392_2023_1623_MOESM1_ESM.docx]

**Additional Information**

**CBX4 deletion promotes tumorigenesis** **under Kras^G12D^ background by inducing genomic instability**

Fangzhen Chen^1*^, Wulei Hou^2*^, Xiangtian Yu^3*^, Jing Wu^1^, Zhengda Li^1^, Jietian Xu^1^, Zimu Deng^4^，Gaobin Chen^4^, Bo Liu^5^, Xiaoxing Yin^6^, Wei Yu^7^, Lei Zhang^4^, Guoliang Xu^4^, Hongbin Ji^4^, Chunmin Liang^1§^, Zuoyun Wang^1§^

**Corresponding Author:**

Zuoyun Wang, Department of Human Anatomy & Histoembryology, School of Basic Medical Sciences, Shanghai Medical College, Fudan University. Room 351, Building1, Dong-An Road 131, Shanghai, China, 200032. E-mail: [wangzuoyun@fudan.edu.cn](mailto:wangzuoyun@fudan.edu.cn).

Chunming Liang, Department of Human Anatomy & Histoembryology, School of Basic Medical Sciences, Shanghai Medical College, Fudan University. Room 208, Building9, Dong-An Road 131, Shanghai, China, 200032. E-mail: [cmliang@fudan.edu.cn](mailto:cmliang@fudan.edu.cn).

**This file includes:**

Supplementary Figure 1-14

Supplementary Table 1-5

**Figure legends:**


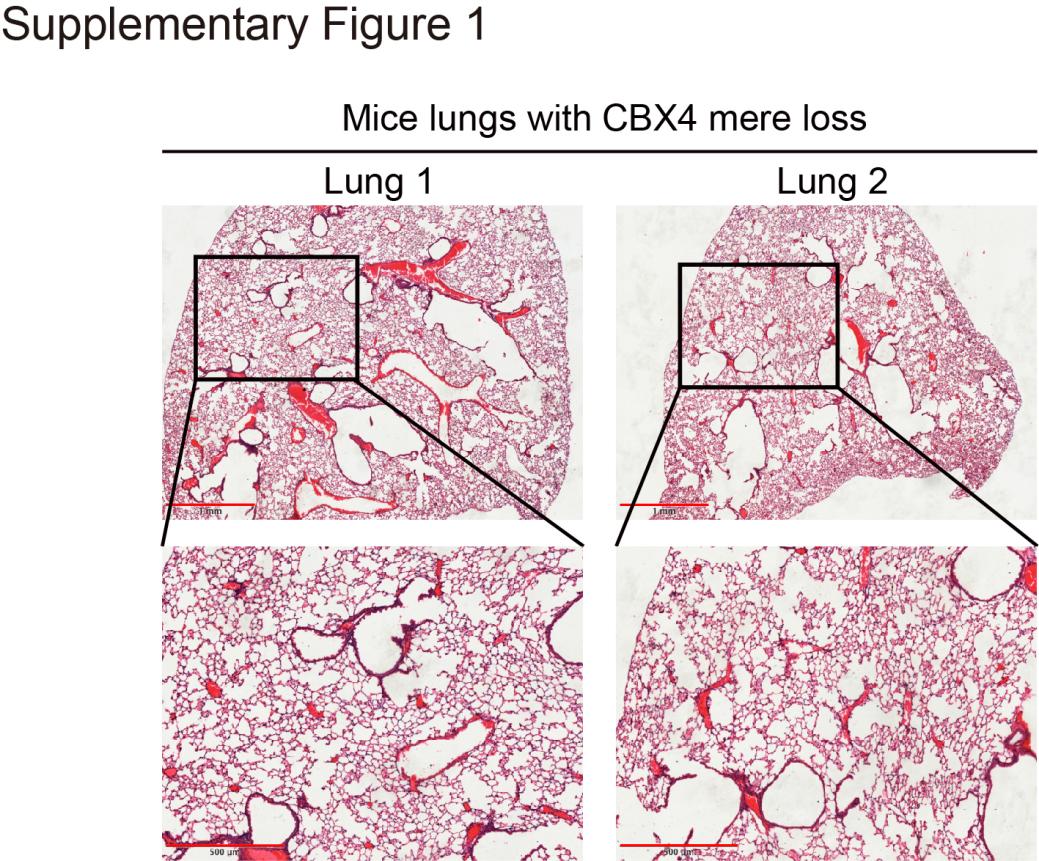


**SF1. CBX4 deletion alone does not cause tumor formation in mice lung.** Representative H&E staining of mice lung with CBX4 merely loss. Scale bar: 1 mm (up), 500 µm (down).


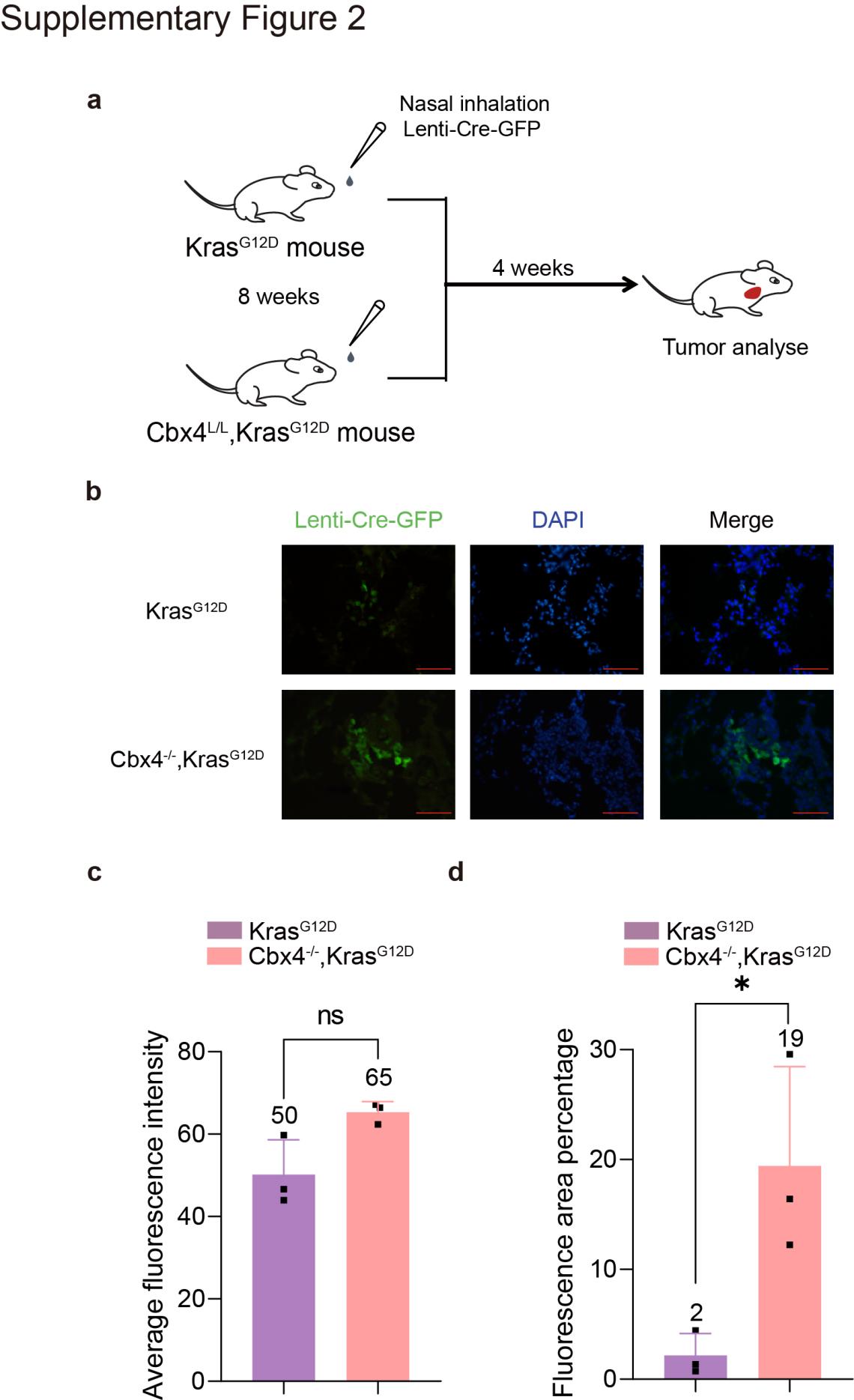


**SF2. CBX4 deletion promotes tumor-like *Kras^G12D^* cell aggregation.**

(**a**) A scheme for Lenti-Cre virus treatment in *Kras^G12D^* and *Cbx4^L/L^, Kras^G12D^* mouse model. (**b**) IF staining of *Kras^G12D^* cells (green cells means the *Kras^G12D^* activated cells infected with Lenti-Cre-GFP) and DAPI (blue) in *Kras^G12D^* and *Cbx4^-/-^, Kras^G12D^* mice. Scale bar: 500 μm. (**c**) Quantitative analysis of average fluorescence intensity of *Kras^G12D^* and *Cbx4^-/-^, Kras^G12D^* mice IF staining. (**d**) Quantitative analysis of fluorescence area percentage of *Kras^G12D^* and *Cbx4^-/-^, Kras^G12D^* mice IF staining. Data are shown as means ± SEM. Ns *P* > 0.05, **P* < 0.05.


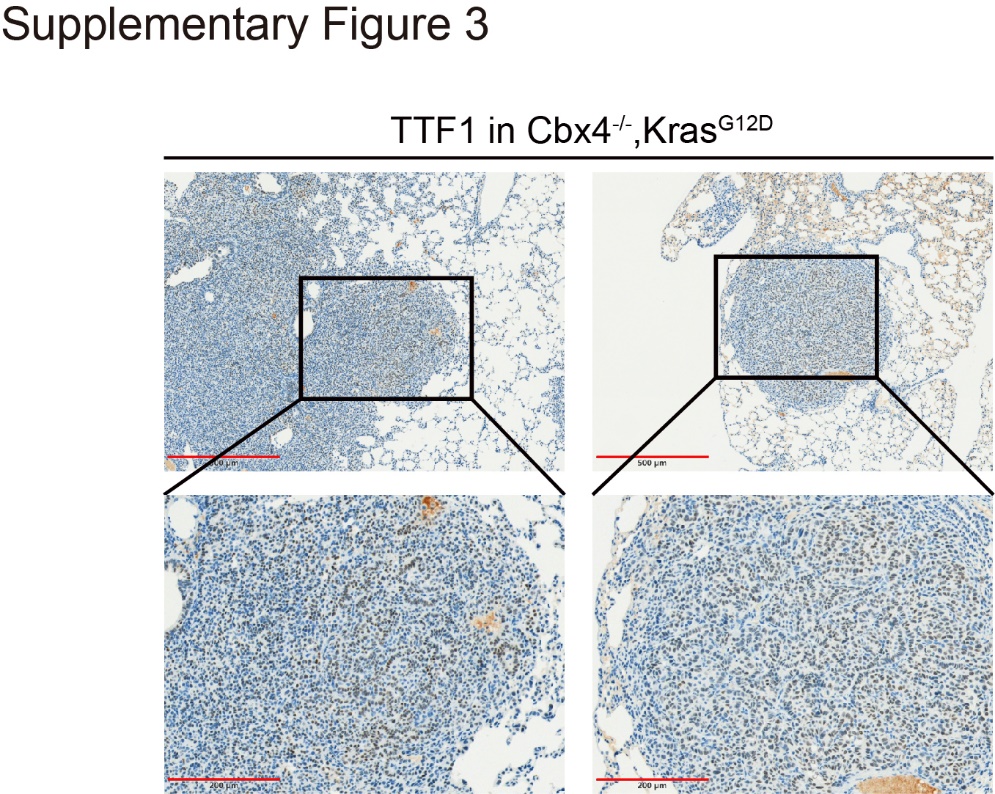


**SF3. CBX4 deletion promotes lung adenocarcinoma in *Kras^G12D^* mice model.**

Representative immunohistochemical staining of TTF1 (a typical marker of LUAD) in lung sections from *Cbx4^-/-^, Kras^G12D^* mice. Scale bar: 500 μm (up), 200 μm (down).


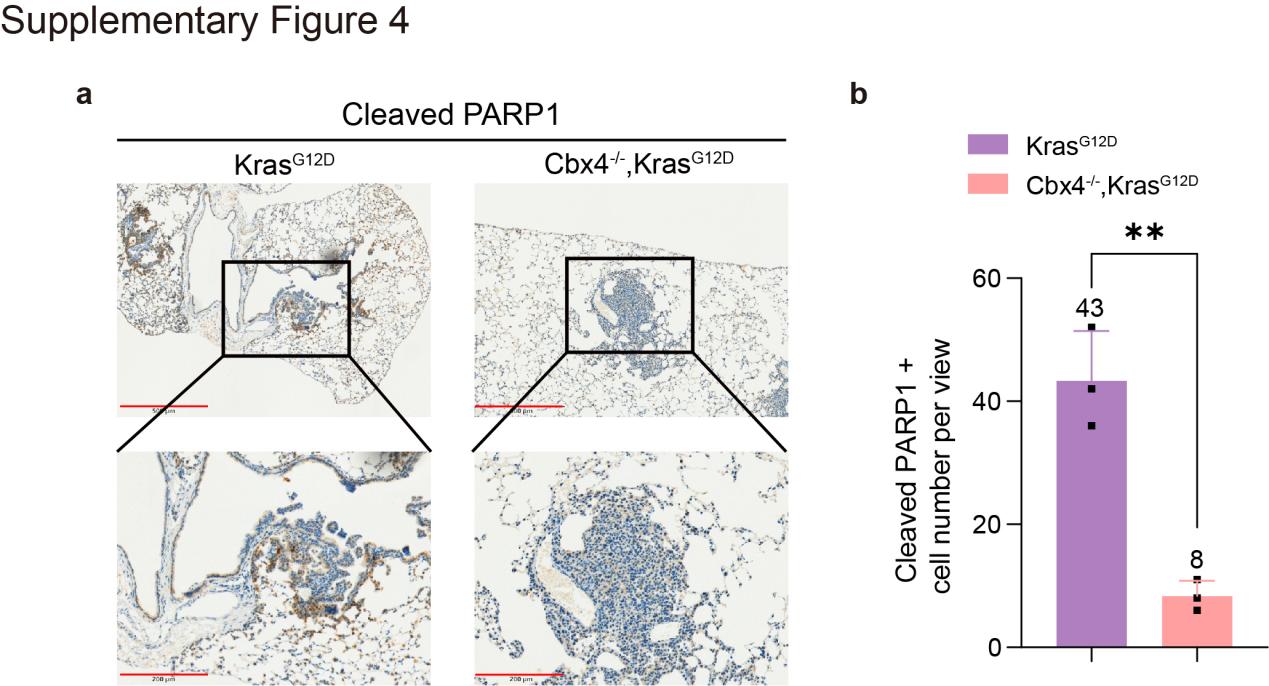


**SF4. Tumors of *Cbx4^-/-^, Kras^G12D^* mice show less cell apoptosis.**

(**a**) Representative immunohistochemical staining of cleaved PARP1 in lung sections from *Kras^G12D^* and *Cbx4^-/-^, Kras^G12D^* mice. Scale bar: 500 μm (up), 200 μm (down). (**b**) Quantitative analysis of cleaved PARP1 positive cell number in IHC staining of lung sections from *Kras^G12D^* and *Cbx4^-/-^, Kras^G12D^* mice. Data are shown as means ± SEM. ***P* < 0.01.


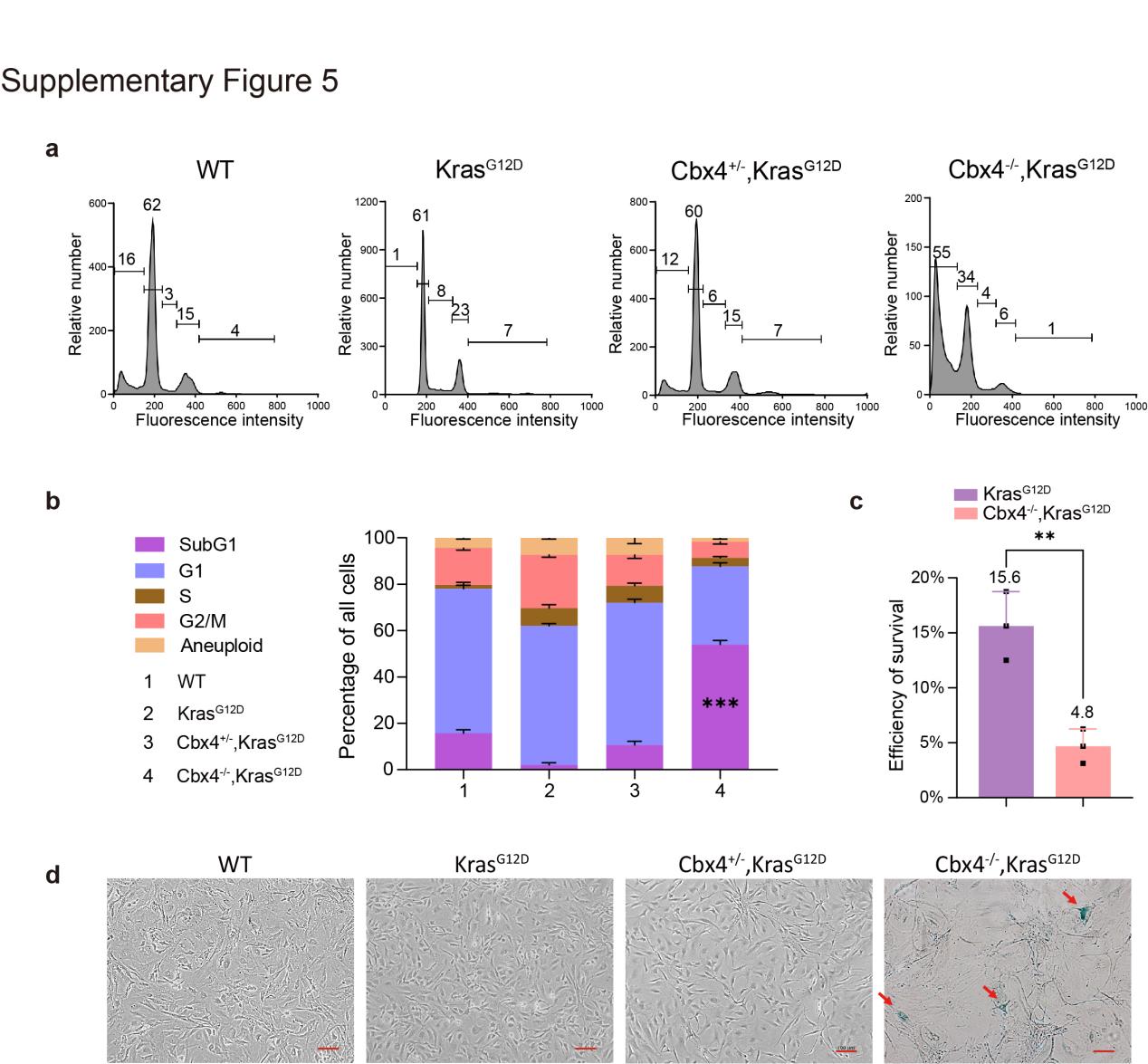


**SF****5. CBX4 deletion induces cell apoptosis in *Kras^G12D^* MEFs.**

(**a**) FACS analysis of *Wild-type*, *Kras^G12D^,* *Cbx4^+/-^, Kras^G12D^* and *Cbx4^-/-^, Kras^G12D^* MEFs of cell proportion at each stage. (**b**) Quantitative analysis of *Wild-type*, *Kras^G12D^,* *Cbx4^+/-^, Kras^G12D^* and *Cbx4^-/-^, Kras^G12D^* MEFs of cell proportion at each stage. (**c**) Cell culture of *Cbx4^-/-^, Kras^G12D^* MEFs and measured efficiency of survival compared with *Wild-type*. (**d**) β-gal galactosidase staining of *Wild-type, Kras^G12D^,* *Cbx4^+/-^, Kras^G12D^* and *Cbx4^-/-^, Kras^G12D^* MEFs. Arrows indicate positive part. Scale bar: 100 μm. Data are shown as means ± SEM. ***P* < 0.01, ****P* < 0.001.


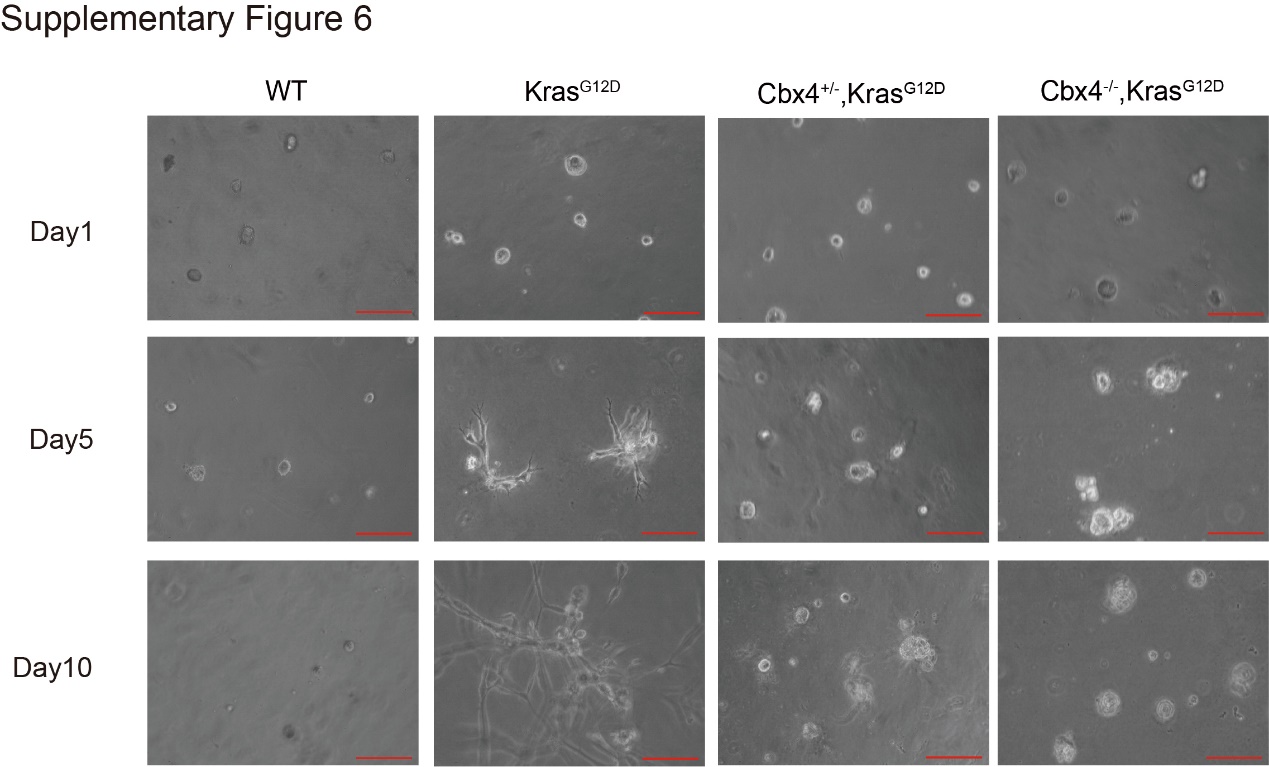


**SF6. CBX4 loss impedes cell proliferation and invasion compared with Kras^G12D^ group.**

3D cell culture of *Wild-type, Kras^G12D^,* *Cbx4^+/-^, Kras^G12D^* and *Cbx4^-/-^, Kras^G12D^*. 3D cell culture showed the growth of cells on 1st, 5th, and 10th days respectively. Scale bar: 200 μm.


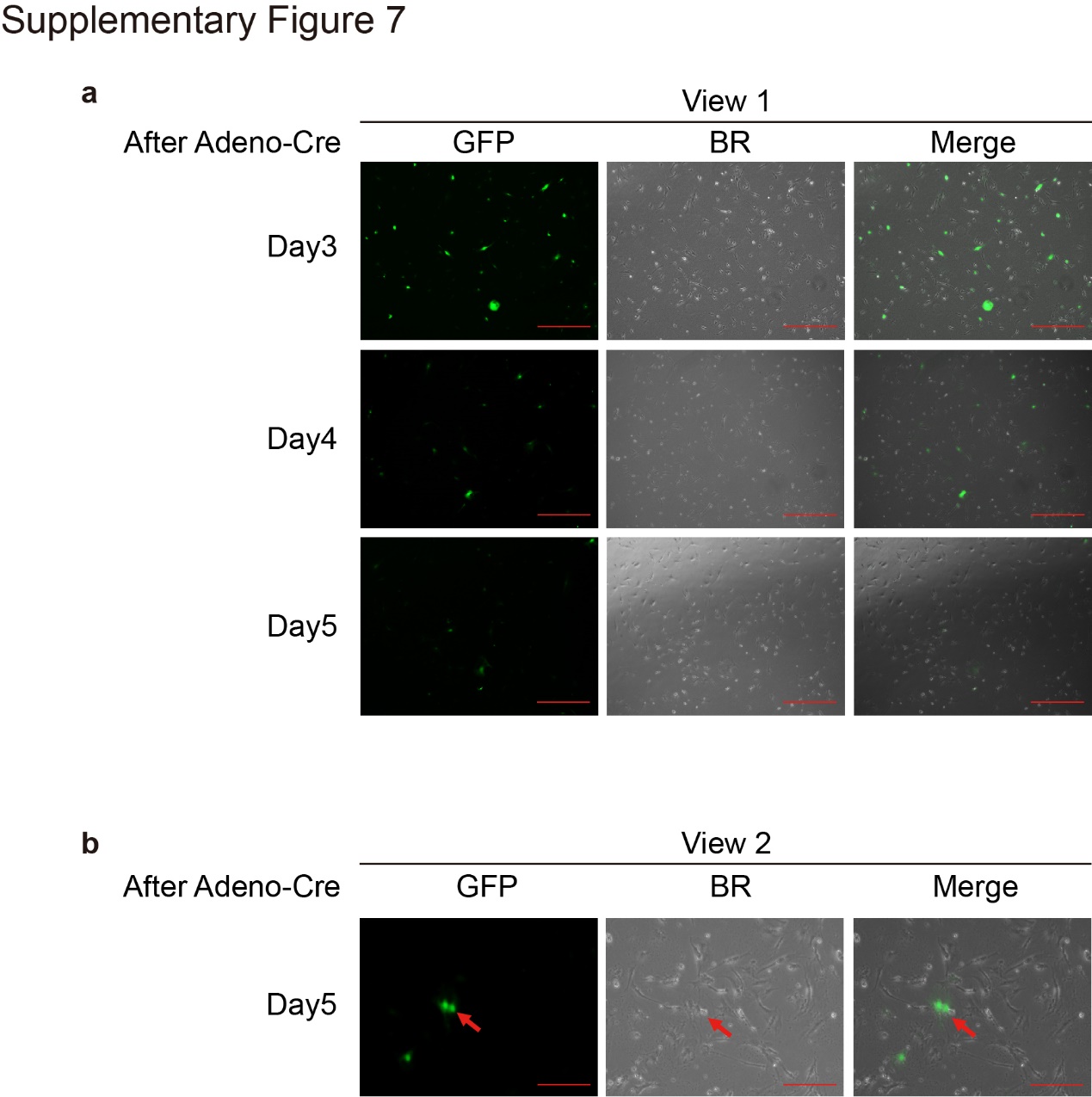


**SF7. Cre-GFP shows cell fate changes of primary *Cbx4^-/-^, Kras^G12D^* MEFs.**

(**a, b**) Fluorescence and bright light photography in day3, 4 and 5 after Adeno-Cre treatment to show the cell fate of primary *Cbx4^-/-^, Kras^G12D^* MEFs. Scale bar: 500 μm (a) and 200 μm (b).


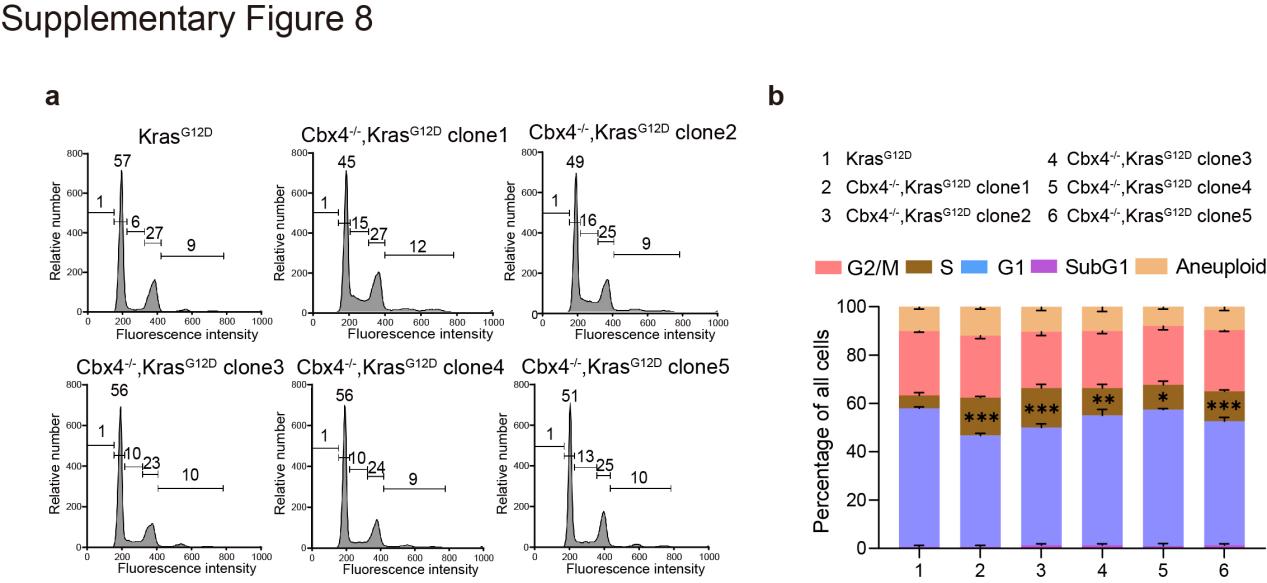


**SF8. FACS analyses of typical selected *Cbx4^-/-^, Kras^G12D^* clones.**

(**a**) FACS analysis of 5 selected *Cbx4^-/-^, Kras^G12D^* clones and simple *Kras^G12D^* MEFs. (**b**) Quantitative analysis of cell proportion at each stage. Data are shown as means ± SEM. **P* < 0.05, ***P* < 0.01, ****P* < 0.001.


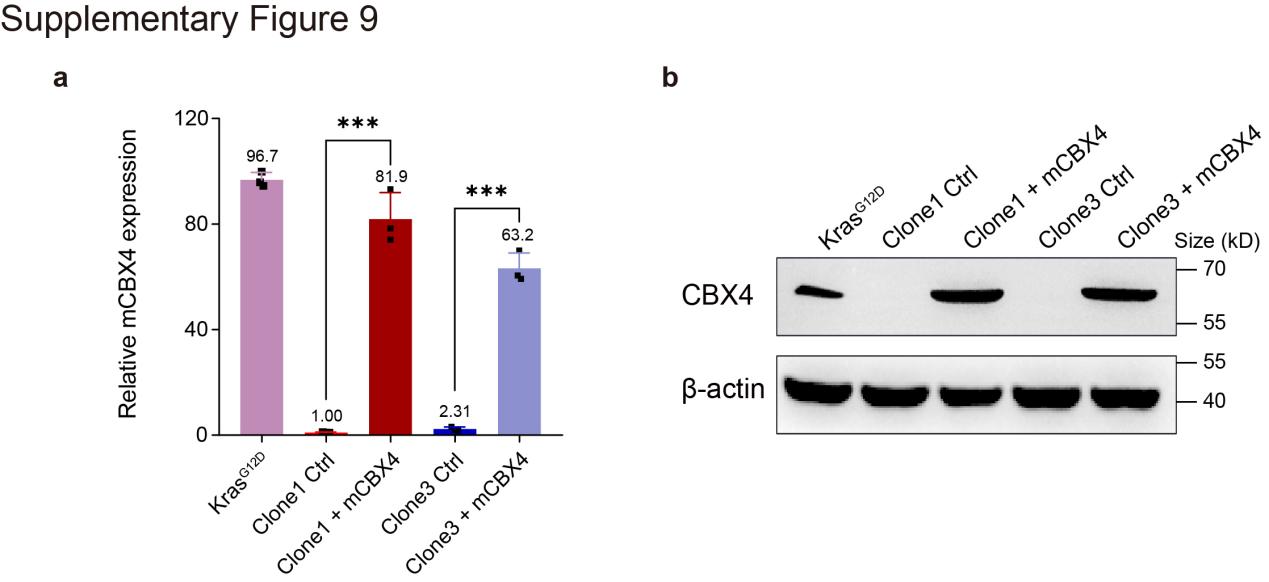


**SF9. CBX4 expression in *Kras^G12D^* and different *Cbx4^-/-^, Kras^G12D^* clones.**

(**a**) Real-time PCR analysis to show relative CBX4 expression of selected *Cbx4^-/-^, Kras^G12D^* cells with or without ectopic CBX4 compared with *Kras^G12D^* cells. (**b**) Western Blot of CBX4 expression of different selected *Cbx4^-/-^, Kras^G12D^* clones compared with *Kras^G12D^* cells. Data are shown as means ± SEM. ****P* < 0.001.


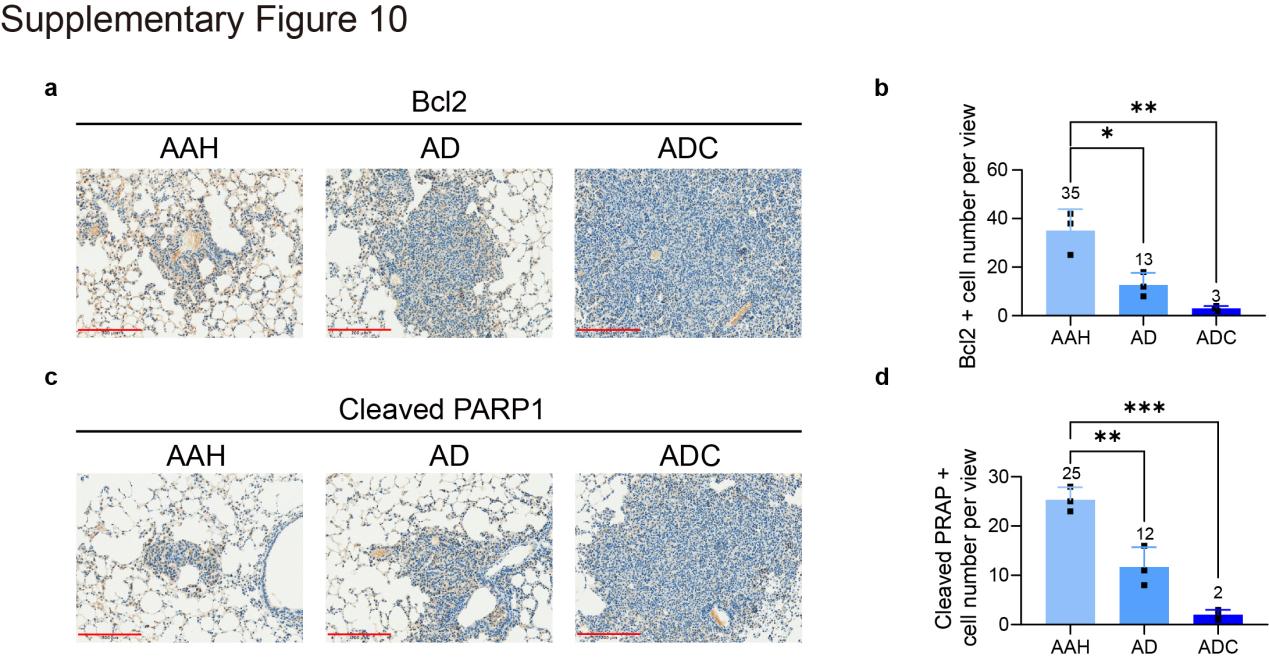


**SF10. Apoptosis-related gene Bcl2 and cleaved PARP1 decrease during the development of LUAD.**

(**a**) Representative immunohistochemical staining of Bcl2 in lung sections from *Cbx4^-/-^, Kras^G12D^* mice. Scale bar: 200 μm. (**b**) Quantitative analysis of Bcl2 positive cell number in IHC staining of lung sections of AAH, AD and ADC from *Cbx4^-/-^, Kras^G12D^* mice. (**c**) Representative immunohistochemical staining of Ki67 in lung sections from *Cbx4^-/-^, Kras^G12D^* mice. Scale bar: 200 μm. (**d**) Quantitative analysis of Ki67 positive cell number in IHC staining of lung sections of AAH, AD and ADC from *Cbx4^-/-^, Kras^G12D^* mice. Data are shown as means ± SEM. **P* < 0.05, ***P* < 0.01, ****P* < 0.001.


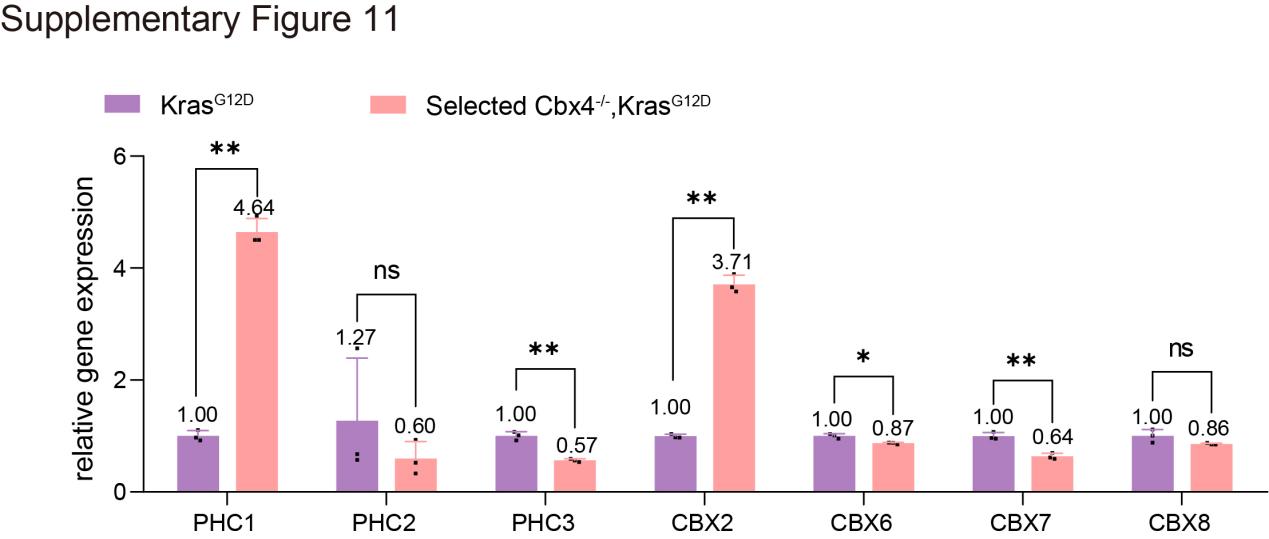


**SF11. The expression levels of PHCs and CBXs change in selected *Cbx4^-/-^, Kras^G12D^* MEFs compared with *Kras^G12D^* MEFs.**

Changes of different PHCs and CBXs’ expression of *Kras^G12D^* and selected *Cbx4^-/-^, Kras^G12D^* MEFs. Data are shown as means ± SEM. Ns *P* > 0.05, **P* < 0.05, ***P* < 0.01.


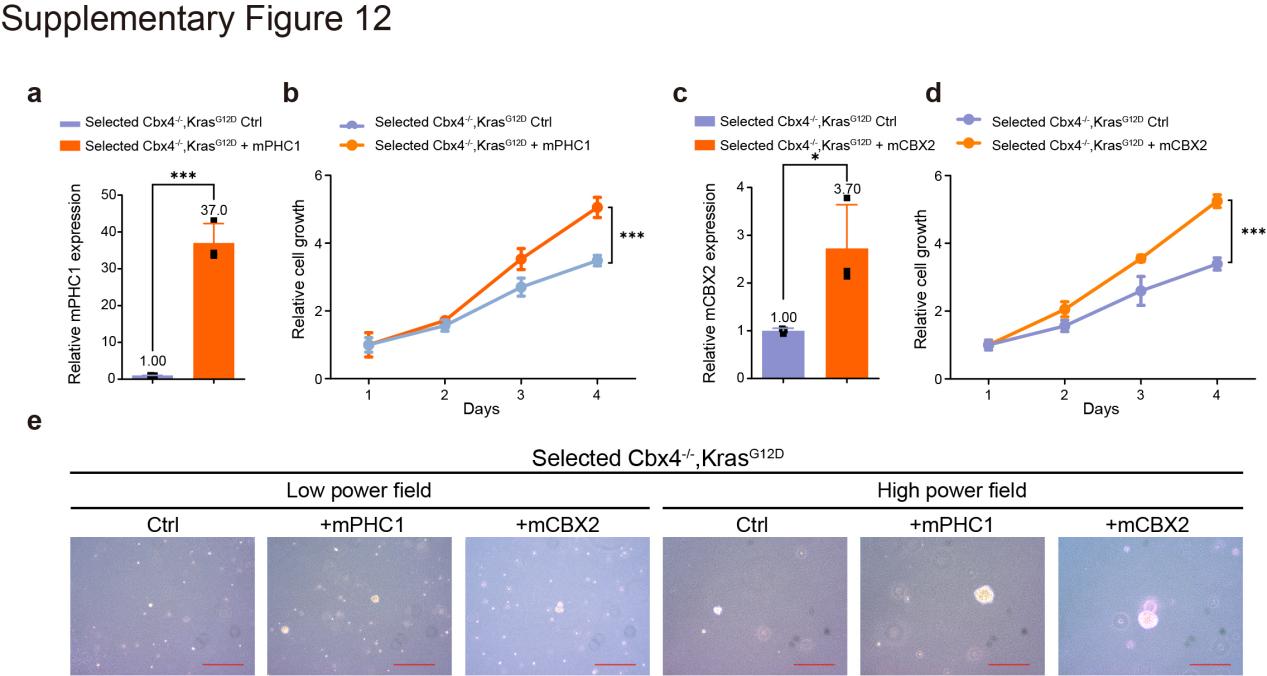


**SF12. mPHC1 and mCBX2 promote cell proliferation and transformation in selected *Cbx4^-/-^, Kras^G12D^* cells.**

(**a**) mPHC1 expression in selected *Cbx4^-/-^, Kras^G12D^* and selected *Cbx4^-/-^, Kras^G12D^* + mPHC1 cells. (**b**) MTT assay of selected *Cbx4^-/-^, Kras^G12D^* and selected *Cbx4^-/-^, Kras^G12D^* + mPHC1 group. (**c**) mCBX2 expression in selected *Cbx4^-/-^, Kras^G12D^* and selected *Cbx4^-/-^, Kras^G12D^* + mCBX2 cells. (**d**) MTT assay of selected *Cbx4^-/-^, Kras^G12D^* and selected *Cbx4^-/-^, Kras^G12D^* + mCBX2 group. (**e**) Soft agar assay in selected *Cbx4^-/-^, Kras^G12D^* cells of control, ectopic mPHC1 and mCBX2 for 14 days. Scale bar: 250 μm (left) and 100 μm (right). Data are shown as means ± SEM. **P* < 0.05, ****P* < 0.001.


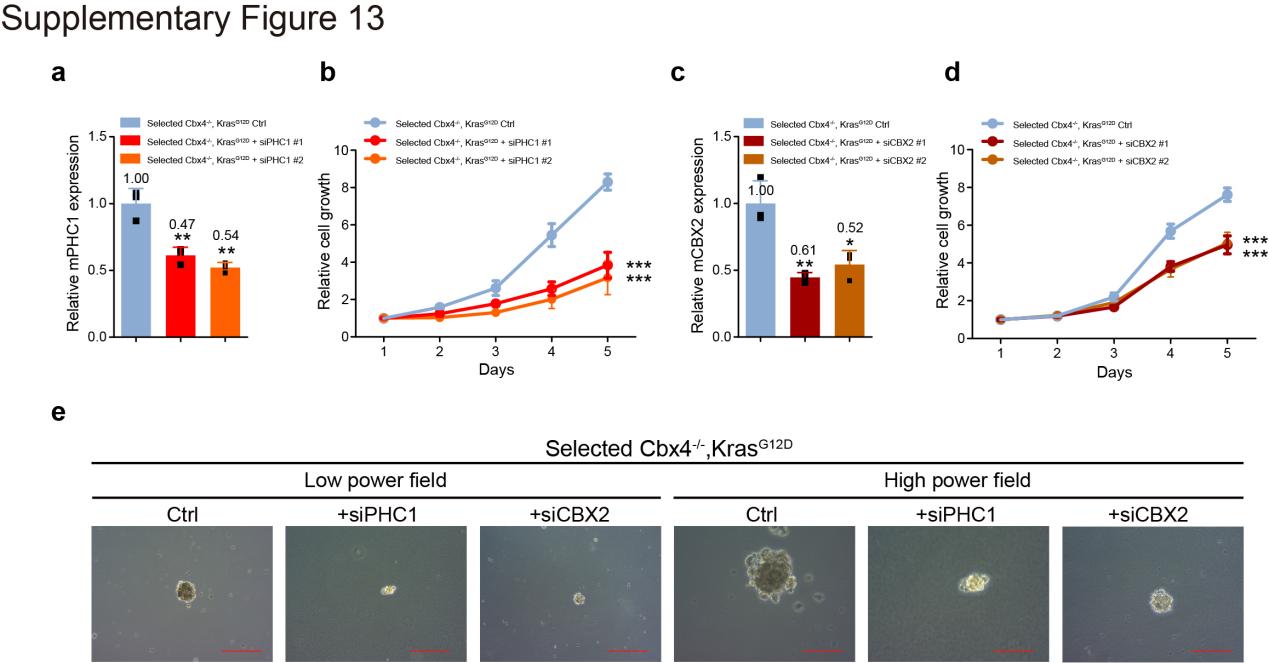


**SF13. siPHC1 and siCBX2 inhibit cell proliferation and transformation in selected *Cbx4^-/-^, Kras^G12D^* cells.**

(**a**) mPHC1 expression in selected *Cbx4^-/-^, Kras^G12D^* and selected *Cbx4^-/-^, Kras^G12D^* + siPHC1 cells. (**b**) MTT assay of selected *Cbx4^-/-^, Kras^G12D^* and selected *Cbx4^-/-^, Kras^G12D^* + siPHC1 group. (**c**) mCBX2 expression in selected *Cbx4^-/-^, Kras^G12D^* and selected *Cbx4^-/-^, Kras^G12D^* + siCBX2 cells. (**d**) MTT assay of selected *Cbx4^-/-^, Kras^G12D^* and selected *Cbx4^-/-^, Kras^G12D^* + siCBX2 group. (**e**) Soft agar assay in selected *Cbx4^-/-^, Kras^G12D^* cells of control, with siPHC1 and siCBX2 for 21 days. Scale bar: 100 μm (left) and 500 μm (right). Data are shown as means ± SEM. ***P* < 0.01, ****P* < 0.001.

**
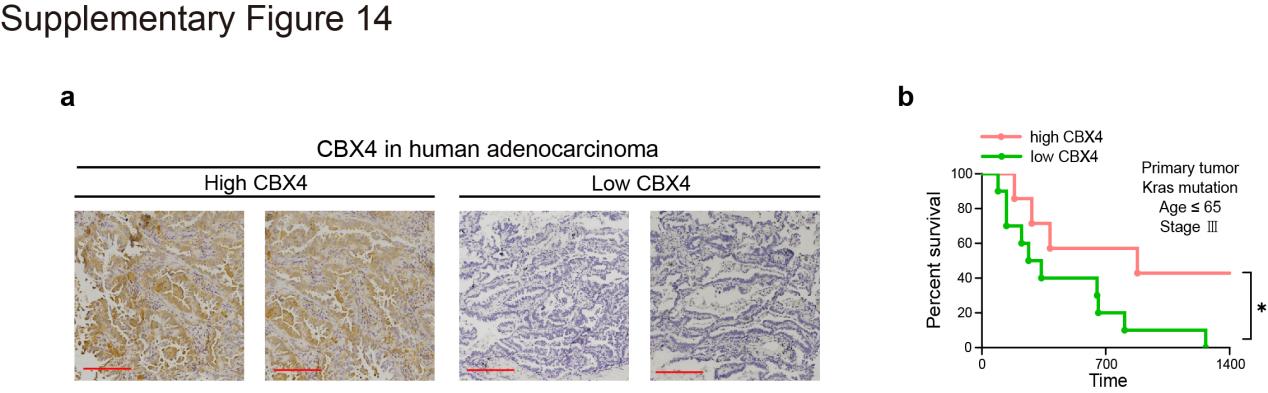
**

**SF14. Low expression of CBX4 in a part of LUAD leads to a worse prognosis under certain condition.**

(**a**) CBX4 highly expressed in a portion of LUAD and also has a low expression in some other cases. (**b**) Survival curve of high and low level of CBX4 in patients with Kras mutation primary tumor (age ≤ 65 years old, Stage Ⅲ). Data are shown as means ± SEM. **P* < 0.05.

**Supplementary Table 1-1. The list of altered genes as the result of G: profiler analysis in primary *Cbx4^-/-^, Kras^G12D^* cells compared with *Kras^G12D^* cells.**

| Gene id | Gene name | Gene description | Primary_vs_Kras^G12D^ |
| --- | --- | --- | --- |
| ENSMUSG00000028185 | Dnase2b | deoxyribonuclease II beta [Source:MGI Symbol;Acc:MGI:1913283] | yes|up |
| ENSMUSG00000028186 | Uox | urate oxidase [Source:MGI Symbol;Acc:MGI:98907] | yes|up |
| ENSMUSG00000109814 | Gm45847 | predicted gene 45847 [Source:MGI Symbol;Acc:MGI:5804962] | yes|up |
| ENSMUSG00000102840 | Gm38037 | predicted gene, 38037 [Source:MGI Symbol;Acc:MGI:5611265] | yes|down |
| ENSMUSG00000081344 | Gm14303 | predicted gene 14303 [Source:MGI Symbol;Acc:MGI:3651379] | yes|down |
| ENSMUSG00000056492 | Adgrf5 | adhesion G protein-coupled receptor F5 [Source:MGI Symbol;Acc:MGI:2182928] | yes|up |
| ENSMUSG00000020758 | Itgb4 | integrin beta 4 [Source:MGI Symbol;Acc:MGI:96613] | yes|down |
| ENSMUSG00000028238 | Atp6v0d2 | ATPase, H+ transporting, lysosomal V0 subunit D2 [Source:MGI Symbol;Acc:MGI:1924415] | yes|up |
| ENSMUSG00000033860 | Fgg | fibrinogen gamma chain [Source:MGI Symbol;Acc:MGI:95526] | yes|up |
| ENSMUSG00000068122 | Agtr2 | angiotensin II receptor, type 2 [Source:MGI Symbol;Acc:MGI:87966] | yes|down |
| ENSMUSG00000078606 | Gvin2 | GTPase, very large interferon inducible, family member 2 [Source:MGI Symbol;Acc:MGI:3782245] | yes|up |
| ENSMUSG00000020364 | Zfp354a | zinc finger protein 354A [Source:MGI Symbol;Acc:MGI:103172] | yes|up |
| ENSMUSG00000121135 | Hmga2-ps1 | high mobility group AT-hook 2, pseudogene 1 [Source:NCBI gene (formerly Entrezgene);Acc:15365] | yes|down |
| ENSMUSG00000046814 | Gchfr | GTP cyclohydrolase I feedback regulator [Source:MGI Symbol;Acc:MGI:2443977] | yes|down |
| ENSMUSG00000044285 | Ubb-ps | ubiquitin B, pseudogene [Source:MGI Symbol;Acc:MGI:3037679] | yes|down |
| ENSMUSG00000054072 | Iigp1 | interferon inducible GTPase 1 [Source:MGI Symbol;Acc:MGI:1926259] | yes|up |
| ENSMUSG00000006958 | Chrd | chordin [Source:MGI Symbol;Acc:MGI:1313268] | yes|up |
| ENSMUSG00000036766 | Dner | delta/notch-like EGF repeat containing [Source:MGI Symbol;Acc:MGI:2152889] | yes|up |
| ENSMUSG00000055805 | Fmnl1 | formin-like 1 [Source:MGI Symbol;Acc:MGI:1888994] | yes|down |
| ENSMUSG00000036768 | Kif15 | kinesin family member 15 [Source:MGI Symbol;Acc:MGI:1098258] | yes|down |
| ENSMUSG00000074384 | AI429214 | expressed sequence AI429214 [Source:MGI Symbol;Acc:MGI:2142538] | yes|up |
| ENSMUSG00000033688 | Inhca | inhibitor of carbonic anhydrase [Source:MGI Symbol;Acc:MGI:1919025] | yes|up |
| ENSMUSG00000039653 | Baat | bile acid-Coenzyme A: amino acid N-acyltransferase [Source:MGI Symbol;Acc:MGI:106642] | yes|up |
| ENSMUSG00000039652 | Cpeb3 | cytoplasmic polyadenylation element binding protein 3 [Source:MGI Symbol;Acc:MGI:2443075] | yes|up |
| ENSMUSG00000065999 | Zfp985 | zinc finger protein 985 [Source:MGI Symbol;Acc:MGI:3651986] | yes|up |
| ENSMUSG00000026208 | Des | desmin [Source:MGI Symbol;Acc:MGI:94885] | yes|up |
| ENSMUSG00000097171 | Gm17644 | predicted gene, 17644 [Source:MGI Symbol;Acc:MGI:4937278] | yes|up |
| ENSMUSG00000030087 | Klf15 | Kruppel-like factor 15 [Source:MGI Symbol;Acc:MGI:1929988] | yes|up |
| ENSMUSG00000030088 | Aldh1l1 | aldehyde dehydrogenase 1 family, member L1 [Source:MGI Symbol;Acc:MGI:1340024] | yes|up |
| ENSMUSG00000031428 | Zcchc18 | zinc finger, CCHC domain containing 18 [Source:MGI Symbol;Acc:MGI:1914245] | yes|down |
| ENSMUSG00000041836 | Ptpre | protein tyrosine phosphatase, receptor type, E [Source:MGI Symbol;Acc:MGI:97813] | yes|down |
| ENSMUSG00000031937 | Vstm5 | V-set and transmembrane domain containing 5 [Source:MGI Symbol;Acc:MGI:1916387] | yes|down |
| ENSMUSG00000115368 | Gm48942 | predicted gene, 48942 [Source:MGI Symbol;Acc:MGI:6118263] | yes|down |
| ENSMUSG00000051242 | Pcdhb9 | protocadherin beta 9 [Source:MGI Symbol;Acc:MGI:2136744] | yes|up |
| ENSMUSG00000029009 | Mthfr | methylenetetrahydrofolate reductase [Source:MGI Symbol;Acc:MGI:106639] | yes|up |
| ENSMUSG00000022053 | Ebf2 | early B cell factor 2 [Source:MGI Symbol;Acc:MGI:894332] | yes|up |
| ENSMUSG00000022055 | Nefl | neurofilament, light polypeptide [Source:MGI Symbol;Acc:MGI:97313] | yes|down |
| ENSMUSG00000031683 | Lsm6 | LSM6 homolog, U6 small nuclear RNA and mRNA degradation associated [Source:MGI Symbol;Acc:MGI:1925901] | yes|down |
| ENSMUSG00000038260 | Trpm4 | transient receptor potential cation channel, subfamily M, member 4 [Source:MGI Symbol;Acc:MGI:1915917] | yes|up |
| ENSMUSG00000025586 | Cpeb1 | cytoplasmic polyadenylation element binding protein 1 [Source:MGI Symbol;Acc:MGI:108442] | yes|up |
| ENSMUSG00000025582 | Nptx1 | neuronal pentraxin 1 [Source:MGI Symbol;Acc:MGI:107811] | yes|down |
| ENSMUSG00000029910 | Mad2l1 | MAD2 mitotic arrest deficient-like 1 [Source:MGI Symbol;Acc:MGI:1860374] | yes|down |
| ENSMUSG00000022272 | Myo10 | myosin X [Source:MGI Symbol;Acc:MGI:107716] | yes|up |
| ENSMUSG00000022582 | Ly6g | lymphocyte antigen 6 complex, locus G [Source:MGI Symbol;Acc:MGI:109440] | yes|down |
| ENSMUSG00000035274 | Tpbg | trophoblast glycoprotein [Source:MGI Symbol;Acc:MGI:1341264] | yes|down |
| ENSMUSG00000048503 | Tlcd5 | TLC domain containing 5 [Source:MGI Symbol;Acc:MGI:2685030] | yes|up |
| ENSMUSG00000038883 | Prl3a1 | prolactin family 3, subfamily a, member 1 [Source:MGI Symbol;Acc:MGI:1914250] | yes|up |
| ENSMUSG00000038884 | Shfl | shiftless antiviral inhibitor of ribosomal frameshifting [Source:MGI Symbol;Acc:MGI:2441788] | yes|up |
| ENSMUSG00000087013 | 2610027K06Rik | RIKEN cDNA 2610027K06 gene [Source:MGI Symbol;Acc:MGI:1917159] | yes|up |
| ENSMUSG00000087014 | Gm16364 | predicted gene 16364 [Source:MGI Symbol;Acc:MGI:3840142] | yes|up |
| ENSMUSG00000045031 | Cetn4 | centrin 4 [Source:MGI Symbol;Acc:MGI:2677454] | yes|down |
| ENSMUSG00000022523 | Fgf12 | fibroblast growth factor 12 [Source:MGI Symbol;Acc:MGI:109183] | yes|up |
| ENSMUSG00000021614 | Vcan | versican [Source:MGI Symbol;Acc:MGI:102889] | yes|up |
| ENSMUSG00000039137 | Whrn | whirlin [Source:MGI Symbol;Acc:MGI:2682003] | yes|up |
| ENSMUSG00000120012 |  | novel transcript | yes|down |
| ENSMUSG00000017723 | Wfdc2 | WAP four-disulfide core domain 2 [Source:MGI Symbol;Acc:MGI:1914951] | yes|down |
| ENSMUSG00000017724 | Etv4 | ets variant 4 [Source:MGI Symbol;Acc:MGI:99423] | yes|down |
| ENSMUSG00000021806 | Nid2 | nidogen 2 [Source:MGI Symbol;Acc:MGI:1298229] | yes|up |
| ENSMUSG00000021803 | Cdhr1 | cadherin-related family member 1 [Source:MGI Symbol;Acc:MGI:2157782] | yes|down |
| ENSMUSG00000048782 | Insc | INSC spindle orientation adaptor protein [Source:MGI Symbol;Acc:MGI:1917942] | yes|up |
| ENSMUSG00000107068 | Gm42742 | predicted gene 42742 [Source:MGI Symbol;Acc:MGI:5662879] | yes|down |
| ENSMUSG00000036158 | Prickle1 | prickle planar cell polarity protein 1 [Source:MGI Symbol;Acc:MGI:1916034] | yes|up |
| ENSMUSG00000033308 | Dpyd | dihydropyrimidine dehydrogenase [Source:MGI Symbol;Acc:MGI:2139667] | yes|up |
| ENSMUSG00000032878 | Ccdc85a | coiled-coil domain containing 85A [Source:MGI Symbol;Acc:MGI:2445069] | yes|up |
| ENSMUSG00000093507 | Gm20627 | predicted gene 20627 [Source:MGI Symbol;Acc:MGI:5313074] | yes|up |
| ENSMUSG00000086503 | Xist | inactive X specific transcripts [Source:MGI Symbol;Acc:MGI:98974] | yes|up |
| ENSMUSG00000097616 | 1110019D14Rik | RIKEN cDNA 1110019D14 gene [Source:MGI Symbol;Acc:MGI:1923561] | yes|up |
| ENSMUSG00000030657 | Xylt1 | xylosyltransferase 1 [Source:MGI Symbol;Acc:MGI:2451073] | yes|up |
| ENSMUSG00000112022 | Gm2436 | predicted gene 2436 [Source:MGI Symbol;Acc:MGI:3780603] | yes|down |
| ENSMUSG00000112023 | Lilrb4b | leukocyte immunoglobulin-like receptor, subfamily B, member 4B [Source:MGI Symbol;Acc:MGI:102702] | yes|up |
| ENSMUSG00000022877 | Hrg | histidine-rich glycoprotein [Source:MGI Symbol;Acc:MGI:2146636] | yes|up |
| ENSMUSG00000041642 | Kif21b | kinesin family member 21B [Source:MGI Symbol;Acc:MGI:109234] | yes|up |
| ENSMUSG00000022875 | Kng1 | kininogen 1 [Source:MGI Symbol;Acc:MGI:1097705] | yes|up |
| ENSMUSG00000076434 | Wfdc3 | WAP four-disulfide core domain 3 [Source:MGI Symbol;Acc:MGI:1923897] | yes|up |
| ENSMUSG00000027408 | Cpxm1 | carboxypeptidase X 1 (M14 family) [Source:MGI Symbol;Acc:MGI:1934569] | yes|up |
| ENSMUSG00000038793 | Lefty1 | left right determination factor 1 [Source:MGI Symbol;Acc:MGI:107405] | yes|up |
| ENSMUSG00000027611 | Procr | protein C receptor, endothelial [Source:MGI Symbol;Acc:MGI:104596] | yes|down |
| ENSMUSG00000027342 | Pcna | proliferating cell nuclear antigen [Source:MGI Symbol;Acc:MGI:97503] | yes|down |
| ENSMUSG00000048347 | Pcdhb18 | protocadherin beta 18 [Source:MGI Symbol;Acc:MGI:2136756] | yes|up |
| ENSMUSG00000051748 | Wfdc21 | WAP four-disulfide core domain 21 [Source:MGI Symbol;Acc:MGI:1913357] | yes|up |
| ENSMUSG00000058600 | Rpl30 | ribosomal protein L30 [Source:MGI Symbol;Acc:MGI:98037] | yes|down |
| ENSMUSG00000030431 | Tmem238 | transmembrane protein 238 [Source:MGI Symbol;Acc:MGI:1922935] | yes|down |
| ENSMUSG00000092274 | Neat1 | nuclear paraspeckle assembly transcript 1 (non-protein coding) [Source:MGI Symbol;Acc:MGI:1914211] | yes|up |
| ENSMUSG00000051527 | Usp29 | ubiquitin specific peptidase 29 [Source:MGI Symbol;Acc:MGI:1888998] | yes|up |
| ENSMUSG00000095562 | Gm55594 | predicted gene, 55594 [Source:MGI Symbol;Acc:MGI:6847656] | yes|up |
| ENSMUSG00000096967 | Gm26621 | predicted gene, 26621 [Source:MGI Symbol;Acc:MGI:5477115] | yes|up |
| ENSMUSG00000027962 | Vcam1 | vascular cell adhesion molecule 1 [Source:MGI Symbol;Acc:MGI:98926] | yes|down |
| ENSMUSG00000027966 | Col11a1 | collagen, type XI, alpha 1 [Source:MGI Symbol;Acc:MGI:88446] | yes|up |
| ENSMUSG00000027968 | Larp7 | La ribonucleoprotein domain family, member 7 [Source:MGI Symbol;Acc:MGI:107634] | yes|down |
| ENSMUSG00000003411 | Rab3b | RAB3B, member RAS oncogene family [Source:MGI Symbol;Acc:MGI:1917158] | yes|down |
| ENSMUSG00000031748 | Gnao1 | guanine nucleotide binding protein, alpha O [Source:MGI Symbol;Acc:MGI:95775] | yes|down |
| ENSMUSG00000023045 | Soat2 | sterol O-acyltransferase 2 [Source:MGI Symbol;Acc:MGI:1332226] | yes|up |
| ENSMUSG00000026564 | Dusp27 | dual specificity phosphatase 27 (putative) [Source:MGI Symbol;Acc:MGI:2685055] | yes|up |
| ENSMUSG00000023046 | Igfbp6 | insulin-like growth factor binding protein 6 [Source:MGI Symbol;Acc:MGI:96441] | yes|down |
| ENSMUSG00000111709 | Gm3776 | predicted gene 3776 [Source:MGI Symbol;Acc:MGI:3826440] | yes|down |
| ENSMUSG00000056174 | Col8a2 | collagen, type VIII, alpha 2 [Source:MGI Symbol;Acc:MGI:88464] | yes|up |
| ENSMUSG00000009633 | G0s2 | G0/G1 switch gene 2 [Source:MGI Symbol;Acc:MGI:1316737] | yes|up |
| ENSMUSG00000039518 | Cdsn | corneodesmosin [Source:MGI Symbol;Acc:MGI:3505689] | yes|up |
| ENSMUSG00000012123 | Crybg2 | crystallin beta-gamma domain containing 2 [Source:MGI Symbol;Acc:MGI:1334463] | yes|up |
| ENSMUSG00000094338 | H2bc13 | H2B clustered histone 13 [Source:MGI Symbol;Acc:MGI:2448403] | yes|down |
| ENSMUSG00000061524 | Zic2 | zinc finger protein of the cerebellum 2 [Source:MGI Symbol;Acc:MGI:106679] | yes|up |
| ENSMUSG00000009185 | Ccl8 | chemokine (C-C motif) ligand 8 [Source:MGI Symbol;Acc:MGI:101878] | yes|up |
| ENSMUSG00000045934 | Mtmr11 | myotubularin related protein 11 [Source:MGI Symbol;Acc:MGI:2652817] | yes|down |
| ENSMUSG00000042616 | Oscp1 | organic solute carrier partner 1 [Source:MGI Symbol;Acc:MGI:1916308] | yes|down |
| ENSMUSG00000042613 | Pbxip1 | pre B cell leukemia transcription factor interacting protein 1 [Source:MGI Symbol;Acc:MGI:2441670] | yes|up |
| ENSMUSG00000067780 | Pi15 | peptidase inhibitor 15 [Source:MGI Symbol;Acc:MGI:1934659] | yes|up |
| ENSMUSG00000035443 | Thyn1 | thymocyte nuclear protein 1 [Source:MGI Symbol;Acc:MGI:1925112] | yes|down |
| ENSMUSG00000020739 | Nup85 | nucleoporin 85 [Source:MGI Symbol;Acc:MGI:3046173] | yes|down |
| ENSMUSG00000028259 | Fhl5 | four and a half LIM domains 5 [Source:MGI Symbol;Acc:MGI:1913192] | yes|up |
| ENSMUSG00000063445 | Nmral1 | NmrA-like family domain containing 1 [Source:MGI Symbol;Acc:MGI:1915074] | yes|down |
| ENSMUSG00000046997 | Spsb4 | splA/ryanodine receptor domain and SOCS box containing 4 [Source:MGI Symbol;Acc:MGI:2183445] | yes|up |
| ENSMUSG00000100975 | Gm28875 | predicted gene 28875 [Source:MGI Symbol;Acc:MGI:5579581] | yes|down |
| ENSMUSG00000020340 | Cyfip2 | cytoplasmic FMR1 interacting protein 2 [Source:MGI Symbol;Acc:MGI:1924134] | yes|down |
| ENSMUSG00000107478 | Gm45234 | predicted gene 45234 [Source:MGI Symbol;Acc:MGI:5753810] | yes|down |
| ENSMUSG00000107383 | Gm4366 | predicted gene 4366 [Source:MGI Symbol;Acc:MGI:3782551] | yes|down |
| ENSMUSG00000054728 | Phactr1 | phosphatase and actin regulator 1 [Source:MGI Symbol;Acc:MGI:2659021] | yes|down |
| ENSMUSG00000107385 | C330024D21Rik | RIKEN cDNA C330024D21 gene [Source:MGI Symbol;Acc:MGI:2444116] | yes|down |
| ENSMUSG00000054720 | Lrrc8c | leucine rich repeat containing 8 family, member C [Source:MGI Symbol;Acc:MGI:2140839] | yes|down |
| ENSMUSG00000099927 | Gm8226 | predicted gene 8226 [Source:MGI Symbol;Acc:MGI:3643827] | yes|up |
| ENSMUSG00000024084 | Qpct | glutaminyl-peptide cyclotransferase (glutaminyl cyclase) [Source:MGI Symbol;Acc:MGI:1917786] | yes|up |
| ENSMUSG00000074818 | Pdzd7 | PDZ domain containing 7 [Source:MGI Symbol;Acc:MGI:3608325] | yes|up |
| ENSMUSG00000024087 | Cyp1b1 | cytochrome P450, family 1, subfamily b, polypeptide 1 [Source:MGI Symbol;Acc:MGI:88590] | yes|up |
| ENSMUSG00000073842 | Mup7 | major urinary protein 7 [Source:MGI Symbol;Acc:MGI:3709615] | yes|down |
| ENSMUSG00000037166 | Ppp1r14a | protein phosphatase 1, regulatory inhibitor subunit 14A [Source:MGI Symbol;Acc:MGI:1931139] | yes|up |
| ENSMUSG00000037161 | Mgarp | mitochondria localized glutamic acid rich protein [Source:MGI Symbol;Acc:MGI:1914999] | yes|up |
| ENSMUSG00000019066 | Rab3d | RAB3D, member RAS oncogene family [Source:MGI Symbol;Acc:MGI:97844] | yes|up |
| ENSMUSG00000040183 | Ankrd6 | ankyrin repeat domain 6 [Source:MGI Symbol;Acc:MGI:2154278] | yes|down |
| ENSMUSG00000040181 | Fmo1 | flavin containing monooxygenase 1 [Source:MGI Symbol;Acc:MGI:1310002] | yes|up |
| ENSMUSG00000120298 |  | novel transcript | yes|up |
| ENSMUSG00000024558 | Mapk4 | mitogen-activated protein kinase 4 [Source:MGI Symbol;Acc:MGI:2444559] | yes|up |
| ENSMUSG00000024556 | Me2 | malic enzyme 2, NAD(+)-dependent, mitochondrial [Source:MGI Symbol;Acc:MGI:2147351] | yes|down |
| ENSMUSG00000000120 | Ngfr | nerve growth factor receptor (TNFR superfamily, member 16) [Source:MGI Symbol;Acc:MGI:97323] | yes|up |
| ENSMUSG00000000690 | Hoxb6 | homeobox B6 [Source:MGI Symbol;Acc:MGI:96187] | yes|up |
| ENSMUSG00000000126 | Wnt9a | wingless-type MMTV integration site family, member 9A [Source:MGI Symbol;Acc:MGI:2446084] | yes|up |
| ENSMUSG00000026779 | Mastl | microtubule associated serine/threonine kinase-like [Source:MGI Symbol;Acc:MGI:1914371] | yes|down |
| ENSMUSG00000026773 | Pfkfb3 | 6-phosphofructo-2-kinase/fructose-2,6-biphosphatase 3 [Source:MGI Symbol;Acc:MGI:2181202] | yes|up |
| ENSMUSG00000097156 | Gm3764 | predicted gene 3764 [Source:MGI Symbol;Acc:MGI:3781938] | yes|down |
| ENSMUSG00000067203 | H2-K2 | histocompatibility 2, K region locus 2 [Source:MGI Symbol;Acc:MGI:95906] | yes|up |
| ENSMUSG00000038128 | Camk4 | calcium/calmodulin-dependent protein kinase IV [Source:MGI Symbol;Acc:MGI:88258] | yes|up |
| ENSMUSG00000041361 | Myzap | myocardial zonula adherens protein [Source:MGI Symbol;Acc:MGI:2142908] | yes|down |
| ENSMUSG00000041362 | Shtn1 | shootin 1 [Source:MGI Symbol;Acc:MGI:1918903] | yes|down |
| ENSMUSG00000031075 | Ano1 | anoctamin 1, calcium activated chloride channel [Source:MGI Symbol;Acc:MGI:2142149] | yes|down |
| ENSMUSG00000030359 | Pzp | PZP, alpha-2-macroglobulin like [Source:MGI Symbol;Acc:MGI:87854] | yes|up |
| ENSMUSG00000030357 | Fkbp4 | FK506 binding protein 4 [Source:MGI Symbol;Acc:MGI:95543] | yes|down |
| ENSMUSG00000030351 | Tspan11 | tetraspanin 11 [Source:MGI Symbol;Acc:MGI:1915748] | yes|down |
| ENSMUSG00000030353 | Tead4 | TEA domain family member 4 [Source:MGI Symbol;Acc:MGI:106907] | yes|down |
| ENSMUSG00000030352 | Tspan9 | tetraspanin 9 [Source:MGI Symbol;Acc:MGI:1924558] | yes|down |
| ENSMUSG00000104814 | Gm42979 | predicted gene 42979 [Source:MGI Symbol;Acc:MGI:5663116] | yes|up |
| ENSMUSG00000059049 | Frem1 | Fras1 related extracellular matrix protein 1 [Source:MGI Symbol;Acc:MGI:2670972] | yes|down |
| ENSMUSG00000022070 | Bora | bora, aurora kinase A activator [Source:MGI Symbol;Acc:MGI:1924994] | yes|down |
| ENSMUSG00000049699 | Ucn2 | urocortin 2 [Source:MGI Symbol;Acc:MGI:2176375] | yes|up |
| ENSMUSG00000057497 | Fam136a | family with sequence similarity 136, member A [Source:MGI Symbol;Acc:MGI:1913738] | yes|down |
| ENSMUSG00000075704 | Txnrd2 | thioredoxin reductase 2 [Source:MGI Symbol;Acc:MGI:1347023] | yes|down |
| ENSMUSG00000026955 | Sapcd2 | suppressor APC domain containing 2 [Source:MGI Symbol;Acc:MGI:1919330] | yes|down |
| ENSMUSG00000026958 | Dpp7 | dipeptidylpeptidase 7 [Source:MGI Symbol;Acc:MGI:1933213] | yes|up |
| ENSMUSG00000098573 | Gm27232 | predicted gene 27232 [Source:MGI Symbol;Acc:MGI:5521075] | yes|down |
| ENSMUSG00000052485 | Tmem171 | transmembrane protein 171 [Source:MGI Symbol;Acc:MGI:2685751] | yes|down |
| ENSMUSG00000029836 | Cbx3 | chromobox 3 [Source:MGI Symbol;Acc:MGI:108515] | yes|down |
| ENSMUSG00000029287 | Tgfbr3 | transforming growth factor, beta receptor III [Source:MGI Symbol;Acc:MGI:104637] | yes|down |
| ENSMUSG00000022253 | Nadk2 | NAD kinase 2, mitochondrial [Source:MGI Symbol;Acc:MGI:1915896] | yes|down |
| ENSMUSG00000029280 | Smr3a | submaxillary gland androgen regulated protein 3A [Source:MGI Symbol;Acc:MGI:102763] | yes|down |
| ENSMUSG00000029283 | Cdc7 | cell division cycle 7 (S. cerevisiae) [Source:MGI Symbol;Acc:MGI:1309511] | yes|down |
| ENSMUSG00000066154 | Mup3 | major urinary protein 3 [Source:MGI Symbol;Acc:MGI:97235] | yes|up |
| ENSMUSG00000032057 | Hoatz | HOATZ cilia and flagella associated protein [Source:MGI Symbol;Acc:MGI:1921013] | yes|down |
| ENSMUSG00000045092 | S1pr1 | sphingosine-1-phosphate receptor 1 [Source:MGI Symbol;Acc:MGI:1096355] | yes|down |
| ENSMUSG00000031004 | Mki67 | antigen identified by monoclonal antibody Ki 67 [Source:MGI Symbol;Acc:MGI:106035] | yes|down |
| ENSMUSG00000057143 | Trim12c | tripartite motif-containing 12C [Source:MGI Symbol;Acc:MGI:4821183] | yes|up |
| ENSMUSG00000028736 | Pax7 | paired box 7 [Source:MGI Symbol;Acc:MGI:97491] | yes|down |
| ENSMUSG00000102411 | Gm36936 | predicted gene, 36936 [Source:MGI Symbol;Acc:MGI:5610164] | yes|up |
| ENSMUSG00000102145 | Gm38056 | predicted gene, 38056 [Source:MGI Symbol;Acc:MGI:5611284] | yes|down |
| ENSMUSG00000102418 | Sh2d1b1 | SH2 domain containing 1B1 [Source:MGI Symbol;Acc:MGI:1349420] | yes|down |
| ENSMUSG00000052516 | Robo2 | roundabout guidance receptor 2 [Source:MGI Symbol;Acc:MGI:1890110] | yes|up |
| ENSMUSG00000028832 | Stmn1 | stathmin 1 [Source:MGI Symbol;Acc:MGI:96739] | yes|down |
| ENSMUSG00000028088 | Fmo5 | flavin containing monooxygenase 5 [Source:MGI Symbol;Acc:MGI:1310004] | yes|up |
| ENSMUSG00000028089 | Chd1l | chromodomain helicase DNA binding protein 1-like [Source:MGI Symbol;Acc:MGI:1915308] | yes|down |
| ENSMUSG00000034459 | Ifit1 | interferon-induced protein with tetratricopeptide repeats 1 [Source:MGI Symbol;Acc:MGI:99450] | yes|up |
| ENSMUSG00000034456 | Uroc1 | urocanase domain containing 1 [Source:MGI Symbol;Acc:MGI:2385332] | yes|up |
| ENSMUSG00000034981 | Parm1 | prostate androgen-regulated mucin-like protein 1 [Source:MGI Symbol;Acc:MGI:2443349] | yes|up |
| ENSMUSG00000039116 | Adgrg6 | adhesion G protein-coupled receptor G6 [Source:MGI Symbol;Acc:MGI:1916151] | yes|up |
| ENSMUSG00000009376 | Met | met proto-oncogene [Source:MGI Symbol;Acc:MGI:96969] | yes|down |
| ENSMUSG00000074151 | Nlrc5 | NLR family, CARD domain containing 5 [Source:MGI Symbol;Acc:MGI:3612191] | yes|up |
| ENSMUSG00000009378 | Slc16a12 | solute carrier family 16 (monocarboxylic acid transporters), member 12 [Source:MGI Symbol;Acc:MGI:2147716] | yes|up |
| ENSMUSG00000013089 | Etv5 | ets variant 5 [Source:MGI Symbol;Acc:MGI:1096867] | yes|down |
| ENSMUSG00000106714 | Gm42546 | predicted gene 42546 [Source:MGI Symbol;Acc:MGI:5662683] | yes|up |
| ENSMUSG00000053646 | Plxnb1 | plexin B1 [Source:MGI Symbol;Acc:MGI:2154238] | yes|up |
| ENSMUSG00000053647 | Gper1 | G protein-coupled estrogen receptor 1 [Source:MGI Symbol;Acc:MGI:1924104] | yes|up |
| ENSMUSG00000053641 | Dennd4a | DENN/MADD domain containing 4A [Source:MGI Symbol;Acc:MGI:2142979] | yes|up |
| ENSMUSG00000086746 | Gm15222 | predicted gene 15222 [Source:MGI Symbol;Acc:MGI:3705297] | yes|up |
| ENSMUSG00000033985 | Tesk2 | testis-specific kinase 2 [Source:MGI Symbol;Acc:MGI:2385204] | yes|down |
| ENSMUSG00000033981 | Gria2 | glutamate receptor, ionotropic, AMPA2 (alpha 2) [Source:MGI Symbol;Acc:MGI:95809] | yes|up |
| ENSMUSG00000002997 | Prkar2b | protein kinase, cAMP dependent regulatory, type II beta [Source:MGI Symbol;Acc:MGI:97760] | yes|down |
| ENSMUSG00000002992 | Apoc2 | apolipoprotein C2 [Source:MGI Symbol;Acc:MGI:88054] | yes|up |
| ENSMUSG00000002228 | Ppm1j | protein phosphatase 1J [Source:MGI Symbol;Acc:MGI:1919137] | yes|up |
| ENSMUSG00000041112 | Elmo1 | engulfment and cell motility 1 [Source:MGI Symbol;Acc:MGI:2153044] | yes|up |
| ENSMUSG00000023829 | Slc22a1 | solute carrier family 22 (organic cation transporter), member 1 [Source:MGI Symbol;Acc:MGI:108111] | yes|up |
| ENSMUSG00000044098 | Rsbn1 | rosbin, round spermatid basic protein 1 [Source:MGI Symbol;Acc:MGI:2444993] | yes|down |
| ENSMUSG00000032855 | Pkd1 | polycystin 1, transient receptor potential channel interacting [Source:MGI Symbol;Acc:MGI:97603] | yes|up |
| ENSMUSG00000078349 | AW011738 | expressed sequence AW011738 [Source:MGI Symbol;Acc:MGI:2140540] | yes|up |
| ENSMUSG00000020083 | Fam241b | family with sequence similarity 241, member B [Source:MGI Symbol;Acc:MGI:1917144] | yes|up |
| ENSMUSG00000063651 | Cnfn | cornifelin [Source:MGI Symbol;Acc:MGI:1919633] | yes|down |
| ENSMUSG00000020086 | Macroh2a2 | macroH2A.2 histone [Source:MGI Symbol;Acc:MGI:3037658] | yes|down |
| ENSMUSG00000075289 | Carns1 | carnosine synthase 1 [Source:MGI Symbol;Acc:MGI:2147595] | yes|down |
| ENSMUSG00000030980 | Knop1 | lysine rich nucleolar protein 1 [Source:MGI Symbol;Acc:MGI:1913606] | yes|down |
| ENSMUSG00000054555 | Adam12 | a disintegrin and metallopeptidase domain 12 (meltrin alpha) [Source:MGI Symbol;Acc:MGI:105378] | yes|up |
| ENSMUSG00000030638 | Sh3gl3 | SH3-domain GRB2-like 3 [Source:MGI Symbol;Acc:MGI:700011] | yes|down |
| ENSMUSG00000048368 | Omd | osteomodulin [Source:MGI Symbol;Acc:MGI:1350918] | yes|down |
| ENSMUSG00000108483 | Gm45184 | predicted gene 45184 [Source:MGI Symbol;Acc:MGI:5753760] | yes|down |
| ENSMUSG00000020882 | Cacnb1 | calcium channel, voltage-dependent, beta 1 subunit [Source:MGI Symbol;Acc:MGI:102522] | yes|up |
| ENSMUSG00000020884 | Asgr1 | asialoglycoprotein receptor 1 [Source:MGI Symbol;Acc:MGI:88081] | yes|up |
| ENSMUSG00000033721 | Vav3 | vav 3 oncogene [Source:MGI Symbol;Acc:MGI:1888518] | yes|down |
| ENSMUSG00000063268 | Parp10 | poly (ADP-ribose) polymerase family, member 10 [Source:MGI Symbol;Acc:MGI:3712326] | yes|up |
| ENSMUSG00000058624 | Gda | guanine deaminase [Source:MGI Symbol;Acc:MGI:95678] | yes|down |
| ENSMUSG00000050410 | Tcf19 | transcription factor 19 [Source:MGI Symbol;Acc:MGI:103180] | yes|down |
| ENSMUSG00000005611 | Irag1 | inositol 1,4,5-triphosphate receptor associated 1 [Source:MGI Symbol;Acc:MGI:1338023] | yes|up |
| ENSMUSG00000055172 | C1ra | complement component 1, r subcomponent A [Source:MGI Symbol;Acc:MGI:1355313] | yes|up |
| ENSMUSG00000030188 | Magohb | mago homolog B, exon junction complex core component [Source:MGI Symbol;Acc:MGI:1913691] | yes|down |
| ENSMUSG00000030189 | Ybx3 | Y box protein 3 [Source:MGI Symbol;Acc:MGI:2137670] | yes|down |
| ENSMUSG00000097090 | Gm26724 | predicted gene, 26724 [Source:MGI Symbol;Acc:MGI:5477218] | yes|up |
| ENSMUSG00000085696 | Hoxaas3 | Hoxa cluster antisense RNA 3 [Source:MGI Symbol;Acc:MGI:1919878] | yes|up |
| ENSMUSG00000030187 | Klra2 | killer cell lectin-like receptor, subfamily A, member 2 [Source:MGI Symbol;Acc:MGI:101906] | yes|down |
| ENSMUSG00000104665 | Gm43366 | predicted gene 43366 [Source:MGI Symbol;Acc:MGI:5663503] | yes|up |
| ENSMUSG00000042745 | Id1 | inhibitor of DNA binding 1, HLH protein [Source:MGI Symbol;Acc:MGI:96396] | yes|down |
| ENSMUSG00000045328 | Cenpe | centromere protein E [Source:MGI Symbol;Acc:MGI:1098230] | yes|down |
| ENSMUSG00000048776 | Pthlh | parathyroid hormone-like peptide [Source:MGI Symbol;Acc:MGI:97800] | yes|down |
| ENSMUSG00000031762 | Mt2 | metallothionein 2 [Source:MGI Symbol;Acc:MGI:97172] | yes|up |
| ENSMUSG00000026542 | Apcs | amyloid P component, serum [Source:MGI Symbol;Acc:MGI:98229] | yes|up |
| ENSMUSG00000026547 | Tagln2 | transgelin 2 [Source:MGI Symbol;Acc:MGI:1312985] | yes|down |
| ENSMUSG00000023066 | Rttn | rotatin [Source:MGI Symbol;Acc:MGI:2179288] | yes|down |
| ENSMUSG00000023064 | Sncg | synuclein, gamma [Source:MGI Symbol;Acc:MGI:1298397] | yes|down |
| ENSMUSG00000118588 |  | serine (or cysteine) peptidase inhibitor, clade A, member 3G (Serpina3g) pseudogene | yes|up |
| ENSMUSG00000115759 | Gm18787 | predicted gene, 18787 [Source:MGI Symbol;Acc:MGI:5010972] | yes|up |
| ENSMUSG00000025931 | Paqr8 | progestin and adipoQ receptor family member VIII [Source:MGI Symbol;Acc:MGI:1921479] | yes|up |
| ENSMUSG00000056158 | Car10 | carbonic anhydrase 10 [Source:MGI Symbol;Acc:MGI:1919855] | yes|up |
| ENSMUSG00000025934 | Gsta3 | glutathione S-transferase, alpha 3 [Source:MGI Symbol;Acc:MGI:95856] | yes|up |
| ENSMUSG00000056153 | Socs6 | suppressor of cytokine signaling 6 [Source:MGI Symbol;Acc:MGI:1924885] | yes|down |
| ENSMUSG00000104610 | Gm567 | predicted gene 567 [Source:MGI Symbol;Acc:MGI:2685413] | yes|down |
| ENSMUSG00000029630 | Cyp3a25 | cytochrome P450, family 3, subfamily a, polypeptide 25 [Source:MGI Symbol;Acc:MGI:1930638] | yes|up |
| ENSMUSG00000003617 | Cp | ceruloplasmin [Source:MGI Symbol;Acc:MGI:88476] | yes|up |
| ENSMUSG00000105827 | H2bc18 | H2B clustered histone 18 [Source:MGI Symbol;Acc:MGI:2448413] | yes|up |
| ENSMUSG00000069303 | H2bc24 | H2B clustered histone 24 [Source:MGI Symbol;Acc:MGI:3710645] | yes|up |
| ENSMUSG00000069305 | H4c18 | H4 clustered histone 18 [Source:MGI Symbol;Acc:MGI:4843992] | yes|up |
| ENSMUSG00000069307 | H2bc23 | H2B clustered histone 23 [Source:MGI Symbol;Acc:MGI:3702051] | yes|up |
| ENSMUSG00000046152 | Fut10 | fucosyltransferase 10 [Source:MGI Symbol;Acc:MGI:2384748] | yes|down |
| ENSMUSG00000002055 | Spag5 | sperm associated antigen 5 [Source:MGI Symbol;Acc:MGI:1927470] | yes|down |
| ENSMUSG00000046794 | Ppp1r3b | protein phosphatase 1, regulatory subunit 3B [Source:MGI Symbol;Acc:MGI:2177268] | yes|up |
| ENSMUSG00000016356 | Col20a1 | collagen, type XX, alpha 1 [Source:MGI Symbol;Acc:MGI:1920618] | yes|up |
| ENSMUSG00000054404 | Slfn5 | schlafen 5 [Source:MGI Symbol;Acc:MGI:1329004] | yes|up |
| ENSMUSG00000038984 | Tspyl5 | testis-specific protein, Y-encoded-like 5 [Source:MGI Symbol;Acc:MGI:2442458] | yes|up |
| ENSMUSG00000069874 | Irgm2 | immunity-related GTPase family M member 2 [Source:MGI Symbol;Acc:MGI:1926262] | yes|up |
| ENSMUSG00000034353 | Ramp1 | receptor (calcitonin) activity modifying protein 1 [Source:MGI Symbol;Acc:MGI:1858418] | yes|up |
| ENSMUSG00000034903 | Cobll1 | Cobl-like 1 [Source:MGI Symbol;Acc:MGI:2442894] | yes|down |
| ENSMUSG00000028278 | Rragd | Ras-related GTP binding D [Source:MGI Symbol;Acc:MGI:1098604] | yes|up |
| ENSMUSG00000092412 | Gm20507 | predicted gene 20507 [Source:MGI Symbol;Acc:MGI:5141972] | yes|up |
| ENSMUSG00000028128 | F3 | coagulation factor III [Source:MGI Symbol;Acc:MGI:88381] | yes|up |
| ENSMUSG00000045268 | Zfp691 | zinc finger protein 691 [Source:MGI Symbol;Acc:MGI:3041163] | yes|up |
| ENSMUSG00000032118 | Fez1 | fasciculation and elongation protein zeta 1 (zygin I) [Source:MGI Symbol;Acc:MGI:2670976] | yes|down |
| ENSMUSG00000032113 | Chek1 | checkpoint kinase 1 [Source:MGI Symbol;Acc:MGI:1202065] | yes|down |
| ENSMUSG00000001506 | Col1a1 | collagen, type I, alpha 1 [Source:MGI Symbol;Acc:MGI:88467] | yes|up |
| ENSMUSG00000001507 | Itga3 | integrin alpha 3 [Source:MGI Symbol;Acc:MGI:96602] | yes|down |
| ENSMUSG00000006221 | Hspb7 | heat shock protein family, member 7 (cardiovascular) [Source:MGI Symbol;Acc:MGI:1352494] | yes|up |
| ENSMUSG00000023505 | Cdca3 | cell division cycle associated 3 [Source:MGI Symbol;Acc:MGI:1315198] | yes|down |
| ENSMUSG00000024713 | Pcsk5 | proprotein convertase subtilisin/kexin type 5 [Source:MGI Symbol;Acc:MGI:97515] | yes|up |
| ENSMUSG00000040747 | Cd53 | CD53 antigen [Source:MGI Symbol;Acc:MGI:88341] | yes|up |
| ENSMUSG00000026712 | Mrc1 | mannose receptor, C type 1 [Source:MGI Symbol;Acc:MGI:97142] | yes|down |
| ENSMUSG00000002297 | Dbf4 | DBF4 zinc finger [Source:MGI Symbol;Acc:MGI:1351328] | yes|down |
| ENSMUSG00000050621 | Rps27rt | ribosomal protein S27, retrogene [Source:MGI Symbol;Acc:MGI:3704345] | yes|down |
| ENSMUSG00000108037 | Gm44597 | predicted gene 44597 [Source:MGI Symbol;Acc:MGI:5753173] | yes|up |
| ENSMUSG00000050628 | Ubald2 | UBA-like domain containing 2 [Source:MGI Symbol;Acc:MGI:1914635] | yes|down |
| ENSMUSG00000086266 | Igf2os | insulin-like growth factor 2, opposite strand [Source:MGI Symbol;Acc:MGI:1195257] | yes|up |
| ENSMUSG00000038618 | Rassf7 | Ras association (RalGDS/AF-6) domain family (N-terminal) member 7 [Source:MGI Symbol;Acc:MGI:1914235] | yes|down |
| ENSMUSG00000010651 | Acaa1b | acetyl-Coenzyme A acyltransferase 1B [Source:MGI Symbol;Acc:MGI:3605455] | yes|up |
| ENSMUSG00000027499 | Pkia | protein kinase inhibitor, alpha [Source:MGI Symbol;Acc:MGI:104747] | yes|up |
| ENSMUSG00000027496 | Aurka | aurora kinase A [Source:MGI Symbol;Acc:MGI:894678] | yes|down |
| ENSMUSG00000000365 | Rnf17 | ring finger protein 17 [Source:MGI Symbol;Acc:MGI:1353419] | yes|down |
| ENSMUSG00000031972 | Acta1 | actin alpha 1, skeletal muscle [Source:MGI Symbol;Acc:MGI:87902] | yes|up |
| ENSMUSG00000072066 | 6720489N17Rik | RIKEN cDNA 6720489N17 gene [Source:MGI Symbol;Acc:MGI:2443901] | yes|down |
| ENSMUSG00000118138 | Gm50322 | predicted gene, 50322 [Source:MGI Symbol;Acc:MGI:6303183] | yes|down |
| ENSMUSG00000022015 | Tnfsf11 | tumor necrosis factor (ligand) superfamily, member 11 [Source:MGI Symbol;Acc:MGI:1100089] | yes|down |
| ENSMUSG00000022018 | Rgcc | regulator of cell cycle [Source:MGI Symbol;Acc:MGI:1913464] | yes|up |
| ENSMUSG00000020528 | Prpsap2 | phosphoribosyl pyrophosphate synthetase-associated protein 2 [Source:MGI Symbol;Acc:MGI:2384838] | yes|down |
| ENSMUSG00000056836 | Pin4-ps | peptidylprolyl cis/trans isomerase, NIMA-interacting 4, pseudogene [Source:MGI Symbol;Acc:MGI:3643618] | yes|down |
| ENSMUSG00000031024 | Denn2b | DENN domain containing 2B [Source:MGI Symbol;Acc:MGI:108517] | yes|up |
| ENSMUSG00000031026 | Trim66 | tripartite motif-containing 66 [Source:MGI Symbol;Acc:MGI:2152406] | yes|up |
| ENSMUSG00000066687 | Zbtb16 | zinc finger and BTB domain containing 16 [Source:MGI Symbol;Acc:MGI:103222] | yes|up |
| ENSMUSG00000028718 | Stil | Scl/Tal1 interrupting locus [Source:MGI Symbol;Acc:MGI:107477] | yes|down |
| ENSMUSG00000028860 | Sytl1 | synaptotagmin-like 1 [Source:MGI Symbol;Acc:MGI:1933365] | yes|down |
| ENSMUSG00000028713 | Cyp4b1 | cytochrome P450, family 4, subfamily b, polypeptide 1 [Source:MGI Symbol;Acc:MGI:103225] | yes|up |
| ENSMUSG00000028715 | Cyp4a14 | cytochrome P450, family 4, subfamily a, polypeptide 14 [Source:MGI Symbol;Acc:MGI:1096550] | yes|up |
| ENSMUSG00000028864 | Hgf | hepatocyte growth factor [Source:MGI Symbol;Acc:MGI:96079] | yes|up |
| ENSMUSG00000034613 | Ppm1h | protein phosphatase 1H (PP2C domain containing) [Source:MGI Symbol;Acc:MGI:2442087] | yes|up |
| ENSMUSG00000021388 | Aspn | asporin [Source:MGI Symbol;Acc:MGI:1913945] | yes|up |
| ENSMUSG00000029409 | U90926 | cDNA sequence U90926 [Source:MGI Symbol;Acc:MGI:1930915] | yes|down |
| ENSMUSG00000042793 | Lgr6 | leucine-rich repeat-containing G protein-coupled receptor 6 [Source:MGI Symbol;Acc:MGI:2441805] | yes|down |
| ENSMUSG00000025762 | Larp1b | La ribonucleoprotein domain family, member 1B [Source:MGI Symbol;Acc:MGI:1914604] | yes|up |
| ENSMUSG00000061482 | H4c4 | H4 clustered histone 4 [Source:MGI Symbol;Acc:MGI:2448423] | yes|up |
| ENSMUSG00000021650 | Ptcd2 | pentatricopeptide repeat domain 2 [Source:MGI Symbol;Acc:MGI:1916177] | yes|down |
| ENSMUSG00000005994 | Tyrp1 | tyrosinase-related protein 1 [Source:MGI Symbol;Acc:MGI:98881] | yes|up |
| ENSMUSG00000093930 | Hmgcs1 | 3-hydroxy-3-methylglutaryl-Coenzyme A synthase 1 [Source:MGI Symbol;Acc:MGI:107592] | yes|up |
| ENSMUSG00000061808 | Ttr | transthyretin [Source:MGI Symbol;Acc:MGI:98865] | yes|up |
| ENSMUSG00000035875 | AI182371 | expressed sequence AI182371 [Source:MGI Symbol;Acc:MGI:2138853] | yes|up |
| ENSMUSG00000053626 | Tll1 | tolloid-like [Source:MGI Symbol;Acc:MGI:106923] | yes|up |
| ENSMUSG00000112963 | Gm6093 | predicted gene 6093 [Source:MGI Symbol;Acc:MGI:3648840] | yes|up |
| ENSMUSG00000032024 | Clmp | CXADR-like membrane protein [Source:MGI Symbol;Acc:MGI:1918816] | yes|down |
| ENSMUSG00000086725 | A630052C17Rik | RIKEN cDNA A630052C17 gene [Source:MGI Symbol;Acc:MGI:2444673] | yes|up |
| ENSMUSG00000035704 | Alg8 | asparagine-linked glycosylation 8 (alpha-1,3-glucosyltransferase) [Source:MGI Symbol;Acc:MGI:2141959] | yes|down |
| ENSMUSG00000120059 |  | novel transcript | yes|up |
| ENSMUSG00000036111 | Lmo1 | LIM domain only 1 [Source:MGI Symbol;Acc:MGI:102812] | yes|down |
| ENSMUSG00000022837 | Iqcb1 | IQ calmodulin-binding motif containing 1 [Source:MGI Symbol;Acc:MGI:2443764] | yes|down |
| ENSMUSG00000022836 | Mylk | myosin, light polypeptide kinase [Source:MGI Symbol;Acc:MGI:894806] | yes|up |
| ENSMUSG00000051910 | Sox6 | SRY (sex determining region Y)-box 6 [Source:MGI Symbol;Acc:MGI:98368] | yes|up |
| ENSMUSG00000075266 | Cenpw | centromere protein W [Source:MGI Symbol;Acc:MGI:1913561] | yes|down |
| ENSMUSG00000058420 | Syt17 | synaptotagmin XVII [Source:MGI Symbol;Acc:MGI:104966] | yes|up |
| ENSMUSG00000097305 | Gm17276 | predicted gene, 17276 [Source:MGI Symbol;Acc:MGI:4936910] | yes|up |
| ENSMUSG00000037649 | H2-DMa | histocompatibility 2, class II, locus DMa [Source:MGI Symbol;Acc:MGI:95921] | yes|up |
| ENSMUSG00000026141 | Col19a1 | collagen, type XIX, alpha 1 [Source:MGI Symbol;Acc:MGI:1095415] | yes|up |
| ENSMUSG00000039774 | Galnt12 | polypeptide N-acetylgalactosaminyltransferase 12 [Source:MGI Symbol;Acc:MGI:2444664] | yes|down |
| ENSMUSG00000031561 | Tenm3 | teneurin transmembrane protein 3 [Source:MGI Symbol;Acc:MGI:1345183] | yes|up |
| ENSMUSG00000041798 | Gck | glucokinase [Source:MGI Symbol;Acc:MGI:1270854] | yes|up |
| ENSMUSG00000031562 | Dctd | dCMP deaminase [Source:MGI Symbol;Acc:MGI:2444529] | yes|down |
| ENSMUSG00000005672 | Kit | KIT proto-oncogene receptor tyrosine kinase [Source:MGI Symbol;Acc:MGI:96677] | yes|up |
| ENSMUSG00000037465 | Klf10 | Kruppel-like factor 10 [Source:MGI Symbol;Acc:MGI:1101353] | yes|down |
| ENSMUSG00000023266 | Frs3 | fibroblast growth factor receptor substrate 3 [Source:MGI Symbol;Acc:MGI:2135965] | yes|up |
| ENSMUSG00000031700 | Gpt2 | glutamic pyruvate transaminase (alanine aminotransferase) 2 [Source:MGI Symbol;Acc:MGI:1915391] | yes|up |
| ENSMUSG00000023000 | Dhh | desert hedgehog [Source:MGI Symbol;Acc:MGI:94891] | yes|down |
| ENSMUSG00000026039 | Sgo2a | shugoshin 2A [Source:MGI Symbol;Acc:MGI:1098767] | yes|down |
| ENSMUSG00000000957 | Mmp14 | matrix metallopeptidase 14 (membrane-inserted) [Source:MGI Symbol;Acc:MGI:101900] | yes|up |
| ENSMUSG00000056133 | Unc93a2 | unc-93 homolog A2 [Source:MGI Symbol;Acc:MGI:3712668] | yes|up |
| ENSMUSG00000000958 | Slc7a7 | solute carrier family 7 (cationic amino acid transporter, y+ system), member 7 [Source:MGI Symbol;Acc:MGI:1337120] | yes|up |
| ENSMUSG00000073633 | Fbxo36 | F-box protein 36 [Source:MGI Symbol;Acc:MGI:1289192] | yes|down |
| ENSMUSG00000047797 | Gjb1 | gap junction protein, beta 1 [Source:MGI Symbol;Acc:MGI:95719] | yes|up |
| ENSMUSG00000049555 | Tmie | transmembrane inner ear [Source:MGI Symbol;Acc:MGI:2159400] | yes|up |
| ENSMUSG00000062110 | Scfd2 | Sec1 family domain containing 2 [Source:MGI Symbol;Acc:MGI:2443446] | yes|down |
| ENSMUSG00000015312 | Gadd45b | growth arrest and DNA-damage-inducible 45 beta [Source:MGI Symbol;Acc:MGI:107776] | yes|up |
| ENSMUSG00000098650 | Commd1b | COMM domain containing 1B [Source:MGI Symbol;Acc:MGI:5547784] | yes|down |
| ENSMUSG00000029656 | C8b | complement component 8, beta polypeptide [Source:MGI Symbol;Acc:MGI:88236] | yes|up |
| ENSMUSG00000022602 | Arc | activity regulated cytoskeletal-associated protein [Source:MGI Symbol;Acc:MGI:88067] | yes|down |
| ENSMUSG00000029875 | Ccdc184 | coiled-coil domain containing 184 [Source:MGI Symbol;Acc:MGI:2146066] | yes|down |
| ENSMUSG00000029659 | Medag | mesenteric estrogen dependent adipogenesis [Source:MGI Symbol;Acc:MGI:1917967] | yes|up |
| ENSMUSG00000038168 | P3h2 | prolyl 3-hydroxylase 2 [Source:MGI Symbol;Acc:MGI:2146663] | yes|up |
| ENSMUSG00000057262 | Rpl15-ps6 | ribosomal protein L15, pseudogene 6 [Source:MGI Symbol;Acc:MGI:3642192] | yes|down |
| ENSMUSG00000083708 | Gm13123 | predicted gene 13123 [Source:MGI Symbol;Acc:MGI:3651473] | yes|down |
| ENSMUSG00000071984 | Fndc1 | fibronectin type III domain containing 1 [Source:MGI Symbol;Acc:MGI:1915905] | yes|up |
| ENSMUSG00000120261 |  | novel transcript | yes|up |
| ENSMUSG00000095427 | Rps2-ps6 | ribosomal protein S2, pseudogene 6 [Source:MGI Symbol;Acc:MGI:3644876] | yes|up |
| ENSMUSG00000064360 | mt-Nd3 | mitochondrially encoded NADH dehydrogenase 3 [Source:MGI Symbol;Acc:MGI:102499] | yes|down |
| ENSMUSG00000032204 | Aqp9 | aquaporin 9 [Source:MGI Symbol;Acc:MGI:1891066] | yes|up |
| ENSMUSG00000024486 | Hbegf | heparin-binding EGF-like growth factor [Source:MGI Symbol;Acc:MGI:96070] | yes|down |
| ENSMUSG00000100937 | Nscme3l | NSE3 homolog, SMC5-SMC6 complex component like [Source:MGI Symbol;Acc:MGI:1922805] | yes|up |
| ENSMUSG00000016150 | Tenm1 | teneurin transmembrane protein 1 [Source:MGI Symbol;Acc:MGI:1345185] | yes|up |
| ENSMUSG00000054766 | Set | SET nuclear oncogene [Source:MGI Symbol;Acc:MGI:1860267] | yes|down |
| ENSMUSG00000045539 | Sprr3 | small proline-rich protein 3 [Source:MGI Symbol;Acc:MGI:1330237] | yes|up |
| ENSMUSG00000061292 | Cyp3a59 | cytochrome P450, family 3, subfamily a, polypeptide 59 [Source:MGI Symbol;Acc:MGI:3769707] | yes|up |
| ENSMUSG00000032425 | Zfp949 | zinc finger protein 949 [Source:MGI Symbol;Acc:MGI:1918890] | yes|down |
| ENSMUSG00000032420 | Nt5e | 5' nucleotidase, ecto [Source:MGI Symbol;Acc:MGI:99782] | yes|down |
| ENSMUSG00000113216 | Gm40841 | predicted gene, 40841 [Source:MGI Symbol;Acc:MGI:5623726] | yes|up |
| ENSMUSG00000018263 | Tbx5 | T-box 5 [Source:MGI Symbol;Acc:MGI:102541] | yes|up |
| ENSMUSG00000031145 | Prickle3 | prickle planar cell polarity protein 3 [Source:MGI Symbol;Acc:MGI:1859635] | yes|up |
| ENSMUSG00000060534 | Dcc | deleted in colorectal carcinoma [Source:MGI Symbol;Acc:MGI:94869] | yes|up |
| ENSMUSG00000079575 | Rbpj-ps3 | recombination signal binding protein for immunoglobulin kappa J region, pseudogene 3 [Source:MGI Symbol;Acc:MGI:96525] | yes|down |
| ENSMUSG00000060459 | Kng2 | kininogen 2 [Source:MGI Symbol;Acc:MGI:3027157] | yes|up |
| ENSMUSG00000061780 | Cfd | complement factor D (adipsin) [Source:MGI Symbol;Acc:MGI:87931] | yes|up |
| ENSMUSG00000033554 | Dph5 | diphthamide biosynthesis 5 [Source:MGI Symbol;Acc:MGI:1916990] | yes|down |
| ENSMUSG00000024598 | Fbn2 | fibrillin 2 [Source:MGI Symbol;Acc:MGI:95490] | yes|up |
| ENSMUSG00000024593 | Megf10 | multiple EGF-like-domains 10 [Source:MGI Symbol;Acc:MGI:2685177] | yes|up |
| ENSMUSG00000024590 | Lmnb1 | lamin B1 [Source:MGI Symbol;Acc:MGI:96795] | yes|down |
| ENSMUSG00000060981 | H4c8 | H4 clustered histone 8 [Source:MGI Symbol;Acc:MGI:2448427] | yes|up |
| ENSMUSG00000049303 | Syt12 | synaptotagmin XII [Source:MGI Symbol;Acc:MGI:2159601] | yes|up |
| ENSMUSG00000003849 | Nqo1 | NAD(P)H dehydrogenase, quinone 1 [Source:MGI Symbol;Acc:MGI:103187] | yes|down |
| ENSMUSG00000086283 | 2810433D01Rik | RIKEN cDNA 2810433D01 gene [Source:MGI Symbol;Acc:MGI:1924382] | yes|up |
| ENSMUSG00000060989 | Gm11847 | predicted gene 11847 [Source:MGI Symbol;Acc:MGI:3651787] | yes|down |
| ENSMUSG00000060988 | Galnt13 | polypeptide N-acetylgalactosaminyltransferase 13 [Source:MGI Symbol;Acc:MGI:2139447] | yes|down |
| ENSMUSG00000097440 | Gm6277 | predicted gene 6277 [Source:MGI Symbol;Acc:MGI:3779583] | yes|up |
| ENSMUSG00000070572 | Trmt112-ps2 | tRNA methyltransferase 11-2, pseudogene 2 [Source:MGI Symbol;Acc:MGI:3651720] | yes|down |
| ENSMUSG00000121088 |  | novel transcript, sense intronic to Szrd1 | yes|up |
| ENSMUSG00000047492 | Inhbe | inhibin beta-E [Source:MGI Symbol;Acc:MGI:109269] | yes|up |
| ENSMUSG00000047496 | Rnf152 | ring finger protein 152 [Source:MGI Symbol;Acc:MGI:2443787] | yes|up |
| ENSMUSG00000047497 | Adamts12 | a disintegrin-like and metallopeptidase (reprolysin type) with thrombospondin type 1 motif, 12 [Source:MGI Symbol;Acc:MGI:2146046] | yes|up |
| ENSMUSG00000026196 | Bard1 | BRCA1 associated RING domain 1 [Source:MGI Symbol;Acc:MGI:1328361] | yes|down |
| ENSMUSG00000026193 | Fn1 | fibronectin 1 [Source:MGI Symbol;Acc:MGI:95566] | yes|up |
| ENSMUSG00000022034 | Esco2 | establishment of sister chromatid cohesion N-acetyltransferase 2 [Source:MGI Symbol;Acc:MGI:1919238] | yes|down |
| ENSMUSG00000022033 | Pbk | PDZ binding kinase [Source:MGI Symbol;Acc:MGI:1289156] | yes|down |
| ENSMUSG00000022032 | Scara5 | scavenger receptor class A, member 5 [Source:MGI Symbol;Acc:MGI:1918395] | yes|down |
| ENSMUSG00000022296 | Baalc | brain and acute leukemia, cytoplasmic [Source:MGI Symbol;Acc:MGI:1928704] | yes|down |
| ENSMUSG00000006403 | Adamts4 | a disintegrin-like and metallopeptidase (reprolysin type) with thrombospondin type 1 motif, 4 [Source:MGI Symbol;Acc:MGI:1339949] | yes|up |
| ENSMUSG00000105987 | AI506816 | expressed sequence AI506816 [Source:MGI Symbol;Acc:MGI:2140929] | yes|up |
| ENSMUSG00000055322 | Tns1 | tensin 1 [Source:MGI Symbol;Acc:MGI:104552] | yes|up |
| ENSMUSG00000052005 | Gm9864 | predicted gene 9864 [Source:MGI Symbol;Acc:MGI:3708663] | yes|up |
| ENSMUSG00000085395 | Gm13056 | predicted gene 13056 [Source:MGI Symbol;Acc:MGI:3650725] | yes|down |
| ENSMUSG00000027203 | Dut | deoxyuridine triphosphatase [Source:MGI Symbol;Acc:MGI:1346051] | yes|down |
| ENSMUSG00000085396 | Firre | functional intergenic repeating RNA element [Source:MGI Symbol;Acc:MGI:2147989] | yes|up |
| ENSMUSG00000027200 | Sema6d | sema domain, transmembrane domain (TM), and cytoplasmic domain, (semaphorin) 6D [Source:MGI Symbol;Acc:MGI:2387661] | yes|down |
| ENSMUSG00000021670 | Hmgcr | 3-hydroxy-3-methylglutaryl-Coenzyme A reductase [Source:MGI Symbol;Acc:MGI:96159] | yes|up |
| ENSMUSG00000039155 | Cdh26 | cadherin-like 26 [Source:MGI Symbol;Acc:MGI:2685856] | yes|down |
| ENSMUSG00000035246 | Pcyt1b | phosphate cytidylyltransferase 1, choline, beta isoform [Source:MGI Symbol;Acc:MGI:2147987] | yes|down |
| ENSMUSG00000067608 | Pcna-ps2 | proliferating cell nuclear antigen pseudogene 2 [Source:MGI Symbol;Acc:MGI:97505] | yes|up |
| ENSMUSG00000069117 | Rps18-ps6 | ribosomal protein S18, pseudogene 6 [Source:MGI Symbol;Acc:MGI:3642298] | yes|up |
| ENSMUSG00000025746 | Il6 | interleukin 6 [Source:MGI Symbol;Acc:MGI:96559] | yes|up |
| ENSMUSG00000028599 | Tnfrsf1b | tumor necrosis factor receptor superfamily, member 1b [Source:MGI Symbol;Acc:MGI:1314883] | yes|down |
| ENSMUSG00000093919 | Gm20760 | predicted gene, 20760 [Source:MGI Symbol;Acc:MGI:5434116] | yes|down |
| ENSMUSG00000021719 | Rgs7bp | regulator of G-protein signalling 7 binding protein [Source:MGI Symbol;Acc:MGI:106334] | yes|up |
| ENSMUSG00000074115 | Saa1 | serum amyloid A 1 [Source:MGI Symbol;Acc:MGI:98221] | yes|up |
| ENSMUSG00000021714 | Cenpk | centromere protein K [Source:MGI Symbol;Acc:MGI:1926210] | yes|down |
| ENSMUSG00000016526 | Dyrk3 | dual-specificity tyrosine-(Y)-phosphorylation regulated kinase 3 [Source:MGI Symbol;Acc:MGI:1330300] | yes|down |
| ENSMUSG00000016520 | Lnx2 | ligand of numb-protein X 2 [Source:MGI Symbol;Acc:MGI:2155959] | yes|up |
| ENSMUSG00000038393 | Txnip | thioredoxin interacting protein [Source:MGI Symbol;Acc:MGI:1889549] | yes|up |
| ENSMUSG00000035811 | Ugt2b35 | UDP glucuronosyltransferase 2 family, polypeptide B35 [Source:MGI Symbol;Acc:MGI:3576100] | yes|up |
| ENSMUSG00000028333 | Anp32b | acidic (leucine-rich) nuclear phosphoprotein 32 family, member B [Source:MGI Symbol;Acc:MGI:1914878] | yes|down |
| ENSMUSG00000028337 | Coro2a | coronin, actin binding protein 2A [Source:MGI Symbol;Acc:MGI:1345966] | yes|down |
| ENSMUSG00000049097 | Ankrd34a | ankyrin repeat domain 34A [Source:MGI Symbol;Acc:MGI:3617846] | yes|up |
| ENSMUSG00002076161 | Rn7sk | RNA, 7SK, nuclear [Source:MGI Symbol;Acc:MGI:103186] | yes|down |
| ENSMUSG00000032092 | Mpzl2 | myelin protein zero-like 2 [Source:MGI Symbol;Acc:MGI:1289160] | yes|down |
| ENSMUSG00000049091 | Sephs2 | selenophosphate synthetase 2 [Source:MGI Symbol;Acc:MGI:108388] | yes|down |
| ENSMUSG00000052911 | Lamb2 | laminin, beta 2 [Source:MGI Symbol;Acc:MGI:99916] | yes|up |
| ENSMUSG00000020102 | Slc16a7 | solute carrier family 16 (monocarboxylic acid transporters), member 7 [Source:MGI Symbol;Acc:MGI:1330284] | yes|up |
| ENSMUSG00000020105 | Lrig3 | leucine-rich repeats and immunoglobulin-like domains 3 [Source:MGI Symbol;Acc:MGI:2443955] | yes|up |
| ENSMUSG00000036139 | Hoxc9 | homeobox C9 [Source:MGI Symbol;Acc:MGI:96199] | yes|up |
| ENSMUSG00000020108 | Ddit4 | DNA-damage-inducible transcript 4 [Source:MGI Symbol;Acc:MGI:1921997] | yes|up |
| ENSMUSG00000117278 | Gm36684 | predicted gene, 36684 [Source:MGI Symbol;Acc:MGI:5595843] | yes|down |
| ENSMUSG00000036390 | Gadd45a | growth arrest and DNA-damage-inducible 45 alpha [Source:MGI Symbol;Acc:MGI:107799] | yes|up |
| ENSMUSG00000032899 | Styk1 | serine/threonine/tyrosine kinase 1 [Source:MGI Symbol;Acc:MGI:2141396] | yes|down |
| ENSMUSG00000039842 | Mcph1 | microcephaly, primary autosomal recessive 1 [Source:MGI Symbol;Acc:MGI:2443308] | yes|down |
| ENSMUSG00000032892 | Rangrf | RAN guanine nucleotide release factor [Source:MGI Symbol;Acc:MGI:1889073] | yes|down |
| ENSMUSG00000107552 | Gm44096 | predicted gene, 44096 [Source:MGI Symbol;Acc:MGI:5690488] | yes|up |
| ENSMUSG00000044320 | 1700001O22Rik | RIKEN cDNA 1700001O22 gene [Source:MGI Symbol;Acc:MGI:1923631] | yes|down |
| ENSMUSG00000078302 | Foxd1 | forkhead box D1 [Source:MGI Symbol;Acc:MGI:1347463] | yes|up |
| ENSMUSG00000078300 | Gm2606 | predicted pseudogene 2606 [Source:MGI Symbol;Acc:MGI:3780774] | yes|down |
| ENSMUSG00000020044 | Timp3 | tissue inhibitor of metalloproteinase 3 [Source:MGI Symbol;Acc:MGI:98754] | yes|up |
| ENSMUSG00000020042 | Btbd11 | BTB (POZ) domain containing 11 [Source:MGI Symbol;Acc:MGI:1921257] | yes|down |
| ENSMUSG00000048327 | Ckap2l | cytoskeleton associated protein 2-like [Source:MGI Symbol;Acc:MGI:1917716] | yes|down |
| ENSMUSG00000051851 | Rtl8c | retrotransposon Gag like 8C [Source:MGI Symbol;Acc:MGI:1920115] | yes|down |
| ENSMUSG00000051855 | Mest | mesoderm specific transcript [Source:MGI Symbol;Acc:MGI:96968] | yes|up |
| ENSMUSG00000048856 | Slc25a47 | solute carrier family 25, member 47 [Source:MGI Symbol;Acc:MGI:2144766] | yes|up |
| ENSMUSG00000016942 | Tmprss6 | transmembrane serine protease 6 [Source:MGI Symbol;Acc:MGI:1919003] | yes|up |
| ENSMUSG00000037664 | Cdkn1c | cyclin-dependent kinase inhibitor 1C (P57) [Source:MGI Symbol;Acc:MGI:104564] | yes|up |
| ENSMUSG00000097324 | Carmn | cardiac mesoderm enhancer-associated non-coding RNA [Source:MGI Symbol;Acc:MGI:4439832] | yes|up |
| ENSMUSG00000037660 | Gdf7 | growth differentiation factor 7 [Source:MGI Symbol;Acc:MGI:95690] | yes|down |
| ENSMUSG00000054510 | Gm14461 | predicted gene 14461 [Source:MGI Symbol;Acc:MGI:3651589] | yes|up |
| ENSMUSG00000033762 | Recql4 | RecQ protein-like 4 [Source:MGI Symbol;Acc:MGI:1931028] | yes|down |
| ENSMUSG00000023243 | Kcnk5 | potassium channel, subfamily K, member 5 [Source:MGI Symbol;Acc:MGI:1336175] | yes|down |
| ENSMUSG00000030494 | Rhpn2 | rhophilin, Rho GTPase binding protein 2 [Source:MGI Symbol;Acc:MGI:1289234] | yes|down |
| ENSMUSG00000040204 | Pclaf | PCNA clamp associated factor [Source:MGI Symbol;Acc:MGI:1915276] | yes|down |
| ENSMUSG00000099759 | 1700030C10Rik | RIKEN cDNA 1700030C10 gene [Source:MGI Symbol;Acc:MGI:1916763] | yes|up |
| ENSMUSG00000112449 | Srp54b | signal recognition particle 54B [Source:MGI Symbol;Acc:MGI:3714357] | yes|up |
| ENSMUSG00000112441 | Gm48898 | predicted gene, 48898 [Source:MGI Symbol;Acc:MGI:6098663] | yes|down |
| ENSMUSG00000050323 | Ndufaf6 | NADH:ubiquinone oxidoreductase complex assembly factor 6 [Source:MGI Symbol;Acc:MGI:1924197] | yes|down |
| ENSMUSG00000005124 | Ccn4 | cellular communication network factor 4 [Source:MGI Symbol;Acc:MGI:1197008] | yes|up |
| ENSMUSG00000005125 | Ndrg1 | N-myc downstream regulated gene 1 [Source:MGI Symbol;Acc:MGI:1341799] | yes|up |
| ENSMUSG00000031549 | Ido2 | indoleamine 2,3-dioxygenase 2 [Source:MGI Symbol;Acc:MGI:2142489] | yes|up |
| ENSMUSG00000026504 | Sdccag8 | serologically defined colon cancer antigen 8 [Source:MGI Symbol;Acc:MGI:1924066] | yes|down |
| ENSMUSG00000026051 | Ecrg4 | ECRG4 augurin precursor [Source:MGI Symbol;Acc:MGI:1926146] | yes|up |
| ENSMUSG00000109324 | Prmt1 | protein arginine N-methyltransferase 1 [Source:MGI Symbol;Acc:MGI:107846] | yes|down |
| ENSMUSG00000030236 | Slco1b2 | solute carrier organic anion transporter family, member 1b2 [Source:MGI Symbol;Acc:MGI:1351899] | yes|up |
| ENSMUSG00000030237 | Slco1a4 | solute carrier organic anion transporter family, member 1a4 [Source:MGI Symbol;Acc:MGI:1351896] | yes|down |
| ENSMUSG00000056445 | Hoxaas2 | Hoxa cluster antisense RNA 2 [Source:MGI Symbol;Acc:MGI:1913890] | yes|up |
| ENSMUSG00000031725 | Ces1f | carboxylesterase 1F [Source:MGI Symbol;Acc:MGI:2142687] | yes|up |
| ENSMUSG00000049538 | Adamts16 | a disintegrin-like and metallopeptidase (reprolysin type) with thrombospondin type 1 motif, 16 [Source:MGI Symbol;Acc:MGI:2429637] | yes|up |
| ENSMUSG00000022197 | Pdzd2 | PDZ domain containing 2 [Source:MGI Symbol;Acc:MGI:1922394] | yes|down |
| ENSMUSG00000022199 | Slc22a17 | solute carrier family 22 (organic cation transporter), member 17 [Source:MGI Symbol;Acc:MGI:1926225] | yes|up |
| ENSMUSG00000038005 | Hpf1 | histone PARylation factor 1 [Source:MGI Symbol;Acc:MGI:1919862] | yes|down |
| ENSMUSG00000038007 | Acer2 | alkaline ceramidase 2 [Source:MGI Symbol;Acc:MGI:1920932] | yes|up |
| ENSMUSG00000085237 | Gm15406 | predicted gene 15406 [Source:MGI Symbol;Acc:MGI:3705112] | yes|down |
| ENSMUSG00000022371 | Col14a1 | collagen, type XIV, alpha 1 [Source:MGI Symbol;Acc:MGI:1341272] | yes|up |
| ENSMUSG00000022372 | Sla | src-like adaptor [Source:MGI Symbol;Acc:MGI:104295] | yes|up |
| ENSMUSG00000029671 | Wnt16 | wingless-type MMTV integration site family, member 16 [Source:MGI Symbol;Acc:MGI:2136018] | yes|up |
| ENSMUSG00000029674 | Limk1 | LIM-domain containing, protein kinase [Source:MGI Symbol;Acc:MGI:104572] | yes|down |
| ENSMUSG00000029859 | Epha1 | Eph receptor A1 [Source:MGI Symbol;Acc:MGI:107381] | yes|down |
| ENSMUSG00000047261 | Gap43 | growth associated protein 43 [Source:MGI Symbol;Acc:MGI:95639] | yes|down |
| ENSMUSG00000031886 | Ces2e | carboxylesterase 2E [Source:MGI Symbol;Acc:MGI:2443170] | yes|up |
| ENSMUSG00000038146 | Notch3 | notch 3 [Source:MGI Symbol;Acc:MGI:99460] | yes|up |
| ENSMUSG00000102516 | Gm38340 | predicted gene, 38340 [Source:MGI Symbol;Acc:MGI:5611568] | yes|up |
| ENSMUSG00000120936 |  | novel transcript | yes|up |
| ENSMUSG00000072844 | G530011O06Rik | RIKEN cDNA G530011O06 gene [Source:MGI Symbol;Acc:MGI:3603513] | yes|up |
| ENSMUSG00000072849 | Serpina1e | serine (or cysteine) peptidase inhibitor, clade A, member 1E [Source:MGI Symbol;Acc:MGI:891967] | yes|up |
| ENSMUSG00000110013 | 1190028D05Rik | RIKEN cDNA 1190028D05 gene [Source:MGI Symbol;Acc:MGI:3036227] | yes|up |
| ENSMUSG00000028108 | Ecm1 | extracellular matrix protein 1 [Source:MGI Symbol;Acc:MGI:103060] | yes|down |
| ENSMUSG00000034800 | Zfp661 | zinc finger protein 661 [Source:MGI Symbol;Acc:MGI:1919430] | yes|up |
| ENSMUSG00000006678 | Pola1 | polymerase (DNA directed), alpha 1 [Source:MGI Symbol;Acc:MGI:99660] | yes|down |
| ENSMUSG00000047910 | Pcdhb16 | protocadherin beta 16 [Source:MGI Symbol;Acc:MGI:2136752] | yes|up |
| ENSMUSG00000062822 | 4833420G17Rik | RIKEN cDNA 4833420G17 gene [Source:MGI Symbol;Acc:MGI:1914642] | yes|down |
| ENSMUSG00000014226 | Cacybp | calcyclin binding protein [Source:MGI Symbol;Acc:MGI:1270839] | yes|down |
| ENSMUSG00000111497 | Gm38431 | predicted gene, 38431 [Source:MGI Symbol;Acc:MGI:5621316] | yes|down |
| ENSMUSG00000102222 | Pcdhga10 | protocadherin gamma subfamily A, 10 [Source:MGI Symbol;Acc:MGI:1935227] | yes|up |
| ENSMUSG00000102224 | 4930447F24Rik | RIKEN cDNA 4930447F24 gene [Source:MGI Symbol;Acc:MGI:1924123] | yes|down |
| ENSMUSG00000045515 | Pou3f3 | POU domain, class 3, transcription factor 3 [Source:MGI Symbol;Acc:MGI:102564] | yes|down |
| ENSMUSG00000037685 | Atp8a1 | ATPase, aminophospholipid transporter (APLT), class I, type 8A, member 1 [Source:MGI Symbol;Acc:MGI:1330848] | yes|up |
| ENSMUSG00000032400 | Zwilch | zwilch kinetochore protein [Source:MGI Symbol;Acc:MGI:1915264] | yes|down |
| ENSMUSG00000019880 | Rspo3 | R-spondin 3 [Source:MGI Symbol;Acc:MGI:1920030] | yes|up |
| ENSMUSG00000021508 | Cxcl14 | chemokine (C-X-C motif) ligand 14 [Source:MGI Symbol;Acc:MGI:1888514] | yes|up |
| ENSMUSG00000079223 | Gm8778 | predicted gene 8778 [Source:MGI Symbol;Acc:MGI:3779812] | yes|up |
| ENSMUSG00000079553 | Kifc1 | kinesin family member C1 [Source:MGI Symbol;Acc:MGI:109596] | yes|down |
| ENSMUSG00000060550 | H2-Q7 | histocompatibility 2, Q region locus 7 [Source:MGI Symbol;Acc:MGI:95936] | yes|up |
| ENSMUSG00000079225 | Gm9531 | predicted gene 9531 [Source:MGI Symbol;Acc:MGI:3779940] | yes|down |
| ENSMUSG00000001819 | Hoxd13 | homeobox D13 [Source:MGI Symbol;Acc:MGI:96205] | yes|down |
| ENSMUSG00000010064 | Slc38a3 | solute carrier family 38, member 3 [Source:MGI Symbol;Acc:MGI:1923507] | yes|up |
| ENSMUSG00000001815 | Evx2 | even-skipped homeobox 2 [Source:MGI Symbol;Acc:MGI:95462] | yes|down |
| ENSMUSG00000033578 | Tmem35a | transmembrane protein 35A [Source:MGI Symbol;Acc:MGI:1914814] | yes|down |
| ENSMUSG00000033576 | Apol6 | apolipoprotein L 6 [Source:MGI Symbol;Acc:MGI:1919189] | yes|up |
| ENSMUSG00000091405 | H4c14 | H4 clustered histone 14 [Source:MGI Symbol;Acc:MGI:2140113] | yes|up |
| ENSMUSG00000022978 | Mis18a | MIS18 kinetochore protein A [Source:MGI Symbol;Acc:MGI:1913828] | yes|down |
| ENSMUSG00000112148 | Lilrb4a | leukocyte immunoglobulin-like receptor, subfamily B, member 4A [Source:MGI Symbol;Acc:MGI:102701] | yes|up |
| ENSMUSG00000000142 | Axin2 | axin 2 [Source:MGI Symbol;Acc:MGI:1270862] | yes|up |
| ENSMUSG00000113637 | Gm7049 | predicted gene 7049 [Source:MGI Symbol;Acc:MGI:3646400] | yes|down |
| ENSMUSG00000037053 | Azgp1 | alpha-2-glycoprotein 1, zinc [Source:MGI Symbol;Acc:MGI:103163] | yes|up |
| ENSMUSG00000030000 | Add2 | adducin 2 (beta) [Source:MGI Symbol;Acc:MGI:87919] | yes|down |
| ENSMUSG00000097466 | D430036J16Rik | RIKEN cDNA D430036J16 gene [Source:MGI Symbol;Acc:MGI:2441977] | yes|up |
| ENSMUSG00000097467 | Gm26737 | predicted gene, 26737 [Source:MGI Symbol;Acc:MGI:5477231] | yes|down |
| ENSMUSG00000027221 | Chst1 | carbohydrate sulfotransferase 1 [Source:MGI Symbol;Acc:MGI:1924219] | yes|down |
| ENSMUSG00000027597 | Ahcy | S-adenosylhomocysteine hydrolase [Source:MGI Symbol;Acc:MGI:87968] | yes|up |
| ENSMUSG00000112324 | Gm47939 | predicted gene, 47939 [Source:MGI Symbol;Acc:MGI:6097203] | yes|up |
| ENSMUSG00000066258 | Trim12a | tripartite motif-containing 12A [Source:MGI Symbol;Acc:MGI:1923931] | yes|down |
| ENSMUSG00000027048 | Abcb11 | ATP-binding cassette, sub-family B (MDR/TAP), member 11 [Source:MGI Symbol;Acc:MGI:1351619] | yes|up |
| ENSMUSG00000080981 | Gm12161 | predicted gene 12161 [Source:MGI Symbol;Acc:MGI:3649484] | yes|down |
| ENSMUSG00000014813 | Stc1 | stanniocalcin 1 [Source:MGI Symbol;Acc:MGI:109131] | yes|up |
| ENSMUSG00000029999 | Tgfa | transforming growth factor alpha [Source:MGI Symbol;Acc:MGI:98724] | yes|down |
| ENSMUSG00000055653 | Gpc3 | glypican 3 [Source:MGI Symbol;Acc:MGI:104903] | yes|up |
| ENSMUSG00000040078 | Ptges3-ps | prostaglandin E synthase 3, pseudogene [Source:MGI Symbol;Acc:MGI:3704271] | yes|down |
| ENSMUSG00000039529 | Atp8b1 | ATPase, class I, type 8B, member 1 [Source:MGI Symbol;Acc:MGI:1859665] | yes|up |
| ENSMUSG00000109244 | Gm44751 | predicted gene 44751 [Source:MGI Symbol;Acc:MGI:5753327] | yes|up |
| ENSMUSG00000048583 | Igf2 | insulin-like growth factor 2 [Source:MGI Symbol;Acc:MGI:96434] | yes|up |
| ENSMUSG00000084350 | Znf41-ps | ZNF41, pseudogene [Source:MGI Symbol;Acc:MGI:1917255] | yes|down |
| ENSMUSG00000025504 | Eps8l2 | EPS8-like 2 [Source:MGI Symbol;Acc:MGI:2138828] | yes|down |
| ENSMUSG00000017861 | Mybl2 | myeloblastosis oncogene-like 2 [Source:MGI Symbol;Acc:MGI:101785] | yes|down |
| ENSMUSG00000017868 | Sgk2 | serum/glucocorticoid regulated kinase 2 [Source:MGI Symbol;Acc:MGI:1351318] | yes|up |
| ENSMUSG00000034656 | Cacna1a | calcium channel, voltage-dependent, P/Q type, alpha 1A subunit [Source:MGI Symbol;Acc:MGI:109482] | yes|down |
| ENSMUSG00000021693 | Kif2a | kinesin family member 2A [Source:MGI Symbol;Acc:MGI:108390] | yes|down |
| ENSMUSG00000021697 | Depdc1b | DEP domain containing 1B [Source:MGI Symbol;Acc:MGI:2145425] | yes|down |
| ENSMUSG00000029445 | Hpd | 4-hydroxyphenylpyruvic acid dioxygenase [Source:MGI Symbol;Acc:MGI:96213] | yes|up |
| ENSMUSG00000029335 | Bmp3 | bone morphogenetic protein 3 [Source:MGI Symbol;Acc:MGI:88179] | yes|down |
| ENSMUSG00000029334 | Prkg2 | protein kinase, cGMP-dependent, type II [Source:MGI Symbol;Acc:MGI:108173] | yes|down |
| ENSMUSG00000022416 | Cacna1i | calcium channel, voltage-dependent, alpha 1I subunit [Source:MGI Symbol;Acc:MGI:2178051] | yes|down |
| ENSMUSG00000022419 | Deptor | DEP domain containing MTOR-interacting protein [Source:MGI Symbol;Acc:MGI:2146322] | yes|down |
| ENSMUSG00000057657 | Rps18-ps3 | ribosomal protein S18, pseudogene 3 [Source:MGI Symbol;Acc:MGI:3642474] | yes|up |
| ENSMUSG00000042759 | Apobr | apolipoprotein B receptor [Source:MGI Symbol;Acc:MGI:2176230] | yes|up |
| ENSMUSG00000042757 | Tmem108 | transmembrane protein 108 [Source:MGI Symbol;Acc:MGI:1932411] | yes|up |
| ENSMUSG00000094724 | Rnaset2b | ribonuclease T2B [Source:MGI Symbol;Acc:MGI:3702087] | yes|up |
| ENSMUSG00000024521 | Pmaip1 | phorbol-12-myristate-13-acetate-induced protein 1 [Source:MGI Symbol;Acc:MGI:1930146] | yes|up |
| ENSMUSG00000046434 | Hnrnpa1 | heterogeneous nuclear ribonucleoprotein A1 [Source:MGI Symbol;Acc:MGI:104820] | yes|down |
| ENSMUSG00000052688 | Rab7b | RAB7B, member RAS oncogene family [Source:MGI Symbol;Acc:MGI:2442295] | yes|down |
| ENSMUSG00000052684 | Jun | jun proto-oncogene [Source:MGI Symbol;Acc:MGI:96646] | yes|down |
| ENSMUSG00000071708 | Sms | spermine synthase [Source:MGI Symbol;Acc:MGI:109490] | yes|down |
| ENSMUSG00000035834 | Polr3g | polymerase (RNA) III (DNA directed) polypeptide G [Source:MGI Symbol;Acc:MGI:1914736] | yes|down |
| ENSMUSG00000064080 | Fbln2 | fibulin 2 [Source:MGI Symbol;Acc:MGI:95488] | yes|up |
| ENSMUSG00000028312 | Smc2 | structural maintenance of chromosomes 2 [Source:MGI Symbol;Acc:MGI:106067] | yes|down |
| ENSMUSG00000002289 | Angptl4 | angiopoietin-like 4 [Source:MGI Symbol;Acc:MGI:1888999] | yes|down |
| ENSMUSG00000052974 | Cyp2f2 | cytochrome P450, family 2, subfamily f, polypeptide 2 [Source:MGI Symbol;Acc:MGI:88608] | yes|up |
| ENSMUSG00000068551 | Zfp467 | zinc finger protein 467 [Source:MGI Symbol;Acc:MGI:1916160] | yes|up |
| ENSMUSG00000078546 | Zfp995 | zinc finger protein 995 [Source:MGI Symbol;Acc:MGI:1917331] | yes|down |
| ENSMUSG00000020123 | Avpr1a | arginine vasopressin receptor 1A [Source:MGI Symbol;Acc:MGI:1859216] | yes|up |
| ENSMUSG00000020121 | Srgap1 | SLIT-ROBO Rho GTPase activating protein 1 [Source:MGI Symbol;Acc:MGI:2152936] | yes|up |
| ENSMUSG00000110206 | Flt3l | FMS-like tyrosine kinase 3 ligand [Source:MGI Symbol;Acc:MGI:95560] | yes|up |
| ENSMUSG00000053835 | H2-T24 | histocompatibility 2, T region locus 24 [Source:MGI Symbol;Acc:MGI:95958] | yes|up |
| ENSMUSG00000075224 | Lrrc55 | leucine rich repeat containing 55 [Source:MGI Symbol;Acc:MGI:2685197] | yes|up |
| ENSMUSG00000020023 | Tmcc3 | transmembrane and coiled coil domains 3 [Source:MGI Symbol;Acc:MGI:2442900] | yes|down |
| ENSMUSG00000053886 | Sh2d4a | SH2 domain containing 4A [Source:MGI Symbol;Acc:MGI:1919531] | yes|up |
| ENSMUSG00000021999 | Cpb2 | carboxypeptidase B2 (plasma) [Source:MGI Symbol;Acc:MGI:1891837] | yes|up |
| ENSMUSG00000021998 | Lcp1 | lymphocyte cytosolic protein 1 [Source:MGI Symbol;Acc:MGI:104808] | yes|up |
| ENSMUSG00000021996 | Esd | esterase D/formylglutathione hydrolase [Source:MGI Symbol;Acc:MGI:95421] | yes|down |
| ENSMUSG00000021991 | Cacna2d3 | calcium channel, voltage-dependent, alpha2/delta subunit 3 [Source:MGI Symbol;Acc:MGI:1338890] | yes|down |
| ENSMUSG00000021993 | Mipep | mitochondrial intermediate peptidase [Source:MGI Symbol;Acc:MGI:1917728] | yes|down |
| ENSMUSG00000107577 | Gm44103 | predicted gene, 44103 [Source:MGI Symbol;Acc:MGI:5690495] | yes|down |
| ENSMUSG00000078896 | Zfp965 | zinc finger protein 965 [Source:MGI Symbol;Acc:MGI:3779822] | yes|down |
| ENSMUSG00000078897 | Gm4724 | predicted gene 4724 [Source:MGI Symbol;Acc:MGI:3782904] | yes|down |
| ENSMUSG00000078894 | 2210418O10Rik | RIKEN cDNA 2210418O10 gene [Source:MGI Symbol;Acc:MGI:1924208] | yes|down |
| ENSMUSG00000001156 | Mxd1 | MAX dimerization protein 1 [Source:MGI Symbol;Acc:MGI:96908] | yes|up |
| ENSMUSG00000079378 | Gm8279 | predicted gene 8279 [Source:MGI Symbol;Acc:MGI:3779794] | yes|down |
| ENSMUSG00000054931 | Zkscan4 | zinc finger with KRAB and SCAN domains 4 [Source:MGI Symbol;Acc:MGI:3649412] | yes|up |
| ENSMUSG00000078898 | Zfp968 | zinc finger protein 968 [Source:MGI Symbol;Acc:MGI:3782903] | yes|down |
| ENSMUSG00000027699 | Ect2 | ect2 oncogene [Source:MGI Symbol;Acc:MGI:95281] | yes|down |
| ENSMUSG00000027690 | Slc2a2 | solute carrier family 2 (facilitated glucose transporter), member 2 [Source:MGI Symbol;Acc:MGI:1095438] | yes|up |
| ENSMUSG00000027692 | Tnik | TRAF2 and NCK interacting kinase [Source:MGI Symbol;Acc:MGI:1916264] | yes|up |
| ENSMUSG00000096617 | Gm5559 | predicted gene 5559 [Source:MGI Symbol;Acc:MGI:3779498] | yes|down |
| ENSMUSG00000121383 | Zfp264 | zinc finger protein 264 [Source:NCBI gene (formerly Entrezgene);Acc:115485607] | yes|up |
| ENSMUSG00000101309 | Gm29397 | predicted gene 29397 [Source:MGI Symbol;Acc:MGI:5580103] | yes|down |
| ENSMUSG00000116121 | Gm49486 | predicted gene, 49486 [Source:MGI Symbol;Acc:MGI:6155161] | yes|down |
| ENSMUSG00000032218 | Ccnb2 | cyclin B2 [Source:MGI Symbol;Acc:MGI:88311] | yes|down |
| ENSMUSG00000040264 | Gbp2b | guanylate binding protein 2b [Source:MGI Symbol;Acc:MGI:95666] | yes|up |
| ENSMUSG00000040263 | Klhdc4 | kelch domain containing 4 [Source:MGI Symbol;Acc:MGI:2384569] | yes|down |
| ENSMUSG00000033031 | Cip2a | cell proliferation regulating inhibitor of protein phosphatase 2A [Source:MGI Symbol;Acc:MGI:2146335] | yes|down |
| ENSMUSG00000033036 | Gm7879 | predicted pseudogene 7879 [Source:MGI Symbol;Acc:MGI:3645078] | yes|down |
| ENSMUSG00000024253 | Dync2li1 | dynein cytoplasmic 2 light intermediate chain 1 [Source:MGI Symbol;Acc:MGI:1913996] | yes|down |
| ENSMUSG00000024924 | Vldlr | very low density lipoprotein receptor [Source:MGI Symbol;Acc:MGI:98935] | yes|up |
| ENSMUSG00000024254 | Abcg8 | ATP binding cassette subfamily G member 8 [Source:MGI Symbol;Acc:MGI:1914720] | yes|up |
| ENSMUSG00000036856 | Wnt4 | wingless-type MMTV integration site family, member 4 [Source:MGI Symbol;Acc:MGI:98957] | yes|up |
| ENSMUSG00000023224 | Serping1 | serine (or cysteine) peptidase inhibitor, clade G, member 1 [Source:MGI Symbol;Acc:MGI:894696] | yes|up |
| ENSMUSG00000041827 | Oasl1 | 2'-5' oligoadenylate synthetase-like 1 [Source:MGI Symbol;Acc:MGI:2180849] | yes|up |
| ENSMUSG00000059481 | Plg | plasminogen [Source:MGI Symbol;Acc:MGI:97620] | yes|up |
| ENSMUSG00000054827 | Cyp2c50 | cytochrome P450, family 2, subfamily c, polypeptide 50 [Source:MGI Symbol;Acc:MGI:2147497] | yes|up |
| ENSMUSG00000059336 | Slc14a1 | solute carrier family 14 (urea transporter), member 1 [Source:MGI Symbol;Acc:MGI:1351654] | yes|down |
| ENSMUSG00000055489 | Ano5 | anoctamin 5 [Source:MGI Symbol;Acc:MGI:3576659] | yes|up |
| ENSMUSG00000044201 | Cdc25c | cell division cycle 25C [Source:MGI Symbol;Acc:MGI:88350] | yes|down |
| ENSMUSG00000030214 | Plbd1 | phospholipase B domain containing 1 [Source:MGI Symbol;Acc:MGI:1914107] | yes|up |
| ENSMUSG00000097709 | 2810429I04Rik | RIKEN cDNA 2810429I04 gene [Source:MGI Symbol;Acc:MGI:1924187] | yes|down |
| ENSMUSG00000015354 | Pcolce2 | procollagen C-endopeptidase enhancer 2 [Source:MGI Symbol;Acc:MGI:1923727] | yes|down |
| ENSMUSG00000040483 | Xaf1 | XIAP associated factor 1 [Source:MGI Symbol;Acc:MGI:3772572] | yes|up |
| ENSMUSG00000056468 | 5730596B20Rik | RIKEN cDNA 5730596B20 gene [Source:MGI Symbol;Acc:MGI:1924830] | yes|up |
| ENSMUSG00000031927 | 1700012B09Rik | RIKEN cDNA 1700012B09 gene [Source:MGI Symbol;Acc:MGI:1916575] | yes|down |
| ENSMUSG00000062591 | Tubb4a | tubulin, beta 4A class IVA [Source:MGI Symbol;Acc:MGI:107848] | yes|up |
| ENSMUSG00000031297 | Slc7a3 | solute carrier family 7 (cationic amino acid transporter, y+ system), member 3 [Source:MGI Symbol;Acc:MGI:1100521] | yes|up |
| ENSMUSG00000022177 | Haus4 | HAUS augmin-like complex, subunit 4 [Source:MGI Symbol;Acc:MGI:1261794] | yes|down |
| ENSMUSG00000049511 | Htr1b | 5-hydroxytryptamine (serotonin) receptor 1B [Source:MGI Symbol;Acc:MGI:96274] | yes|up |
| ENSMUSG00000038022 | Mindy4 | MINDY lysine 48 deubiquitinase 4 [Source:MGI Symbol;Acc:MGI:3583959] | yes|down |
| ENSMUSG00000081058 | H3c15 | H3 clustered histone 15 [Source:MGI Symbol;Acc:MGI:2448357] | yes|up |
| ENSMUSG00000110195 | Pde2a | phosphodiesterase 2A, cGMP-stimulated [Source:MGI Symbol;Acc:MGI:2446107] | yes|up |
| ENSMUSG00000032554 | Trf | transferrin [Source:MGI Symbol;Acc:MGI:98821] | yes|up |
| ENSMUSG00000029838 | Ptn | pleiotrophin [Source:MGI Symbol;Acc:MGI:97804] | yes|up |
| ENSMUSG00000014599 | Csf1 | colony stimulating factor 1 (macrophage) [Source:MGI Symbol;Acc:MGI:1339753] | yes|down |
| ENSMUSG00000012296 | Tjap1 | tight junction associated protein 1 [Source:MGI Symbol;Acc:MGI:1921344] | yes|down |
| ENSMUSG00000048647 | Exd1 | exonuclease 3'-5' domain containing 1 [Source:MGI Symbol;Acc:MGI:3045306] | yes|up |
| ENSMUSG00000043613 | Mmp3 | matrix metallopeptidase 3 [Source:MGI Symbol;Acc:MGI:97010] | yes|down |
| ENSMUSG00000066705 | Fxyd6 | FXYD domain-containing ion transport regulator 6 [Source:MGI Symbol;Acc:MGI:1890226] | yes|up |
| ENSMUSG00000016194 | Hsd11b1 | hydroxysteroid 11-beta dehydrogenase 1 [Source:MGI Symbol;Acc:MGI:103562] | yes|up |
| ENSMUSG00000078154 | Gm12184 | predicted gene 12184 [Source:MGI Symbol;Acc:MGI:3652174] | yes|down |
| ENSMUSG00000069893 | 9930111J21Rik1 | RIKEN cDNA 9930111J21 gene 1 [Source:MGI Symbol;Acc:MGI:3041173] | yes|down |
| ENSMUSG00000034825 | Nrip3 | nuclear receptor interacting protein 3 [Source:MGI Symbol;Acc:MGI:1925843] | yes|up |
| ENSMUSG00000071856 | Mcc | mutated in colorectal cancers [Source:MGI Symbol;Acc:MGI:96930] | yes|down |
| ENSMUSG00000120375 |  | novel transcript | yes|up |
| ENSMUSG00000042256 | Ptchd4 | patched domain containing 4 [Source:MGI Symbol;Acc:MGI:1920485] | yes|up |
| ENSMUSG00000045573 | Penk | preproenkephalin [Source:MGI Symbol;Acc:MGI:104629] | yes|down |
| ENSMUSG00000032179 | Bmp5 | bone morphogenetic protein 5 [Source:MGI Symbol;Acc:MGI:88181] | yes|up |
| ENSMUSG00000032177 | Pde4a | phosphodiesterase 4A, cAMP specific [Source:MGI Symbol;Acc:MGI:99558] | yes|down |
| ENSMUSG00000116594 | Gm49601 | predicted gene, 49601 [Source:MGI Symbol;Acc:MGI:6215011] | yes|up |
| ENSMUSG00000062713 | Sim2 | single-minded family bHLH transcription factor 2 [Source:MGI Symbol;Acc:MGI:98307] | yes|up |
| ENSMUSG00000045284 | Dcaf12l1 | DDB1 and CUL4 associated factor 12-like 1 [Source:MGI Symbol;Acc:MGI:2444462] | yes|up |
| ENSMUSG00000061167 | Rpl15-ps3 | ribosomal protein L15, pseudogene 3 [Source:MGI Symbol;Acc:MGI:3782952] | yes|up |
| ENSMUSG00000044155 | Lsm8 | LSM8 homolog, U6 small nuclear RNA associated [Source:MGI Symbol;Acc:MGI:1923772] | yes|down |
| ENSMUSG00000112129 | Pbld1 | phenazine biosynthesis-like protein domain containing 1 [Source:MGI Symbol;Acc:MGI:1915621] | yes|up |
| ENSMUSG00000037035 | Inhbb | inhibin beta-B [Source:MGI Symbol;Acc:MGI:96571] | yes|up |
| ENSMUSG00000037034 | Pax1 | paired box 1 [Source:MGI Symbol;Acc:MGI:97485] | yes|up |
| ENSMUSG00000037031 | Tspan15 | tetraspanin 15 [Source:MGI Symbol;Acc:MGI:1917673] | yes|down |
| ENSMUSG00000037032 | Apbb1 | amyloid beta (A4) precursor protein-binding, family B, member 1 [Source:MGI Symbol;Acc:MGI:107765] | yes|down |
| ENSMUSG00000014603 | Alx3 | aristaless-like homeobox 3 [Source:MGI Symbol;Acc:MGI:1277097] | yes|down |
| ENSMUSG00000014602 | Kif1a | kinesin family member 1A [Source:MGI Symbol;Acc:MGI:108391] | yes|up |
| ENSMUSG00000027204 | Fbn1 | fibrillin 1 [Source:MGI Symbol;Acc:MGI:95489] | yes|up |
| ENSMUSG00000027208 | Fgf7 | fibroblast growth factor 7 [Source:MGI Symbol;Acc:MGI:95521] | yes|down |
| ENSMUSG00000063142 | Kcnma1 | potassium large conductance calcium-activated channel, subfamily M, alpha member 1 [Source:MGI Symbol;Acc:MGI:99923] | yes|up |
| ENSMUSG00000027068 | Dhrs9 | dehydrogenase/reductase (SDR family) member 9 [Source:MGI Symbol;Acc:MGI:2442798] | yes|down |
| ENSMUSG00000109222 | Gm10297 | predicted pseudogene 10297 [Source:MGI Symbol;Acc:MGI:3642512] | yes|down |
| ENSMUSG00000039501 | Znfx1 | zinc finger, NFX1-type containing 1 [Source:MGI Symbol;Acc:MGI:2138982] | yes|up |
| ENSMUSG00000055675 | Kbtbd11 | kelch repeat and BTB (POZ) domain containing 11 [Source:MGI Symbol;Acc:MGI:1922151] | yes|up |
| ENSMUSG00000006445 | Epha2 | Eph receptor A2 [Source:MGI Symbol;Acc:MGI:95278] | yes|down |
| ENSMUSG00000085028 | Slc2a4rg-ps | Slc2a4 regulator, pseudogene [Source:MGI Symbol;Acc:MGI:3651388] | yes|up |
| ENSMUSG00000040017 | Saa4 | serum amyloid A 4 [Source:MGI Symbol;Acc:MGI:98224] | yes|up |
| ENSMUSG00000085023 | Gm12744 | predicted gene 12744 [Source:MGI Symbol;Acc:MGI:3702539] | yes|down |
| ENSMUSG00000061897 | Gm14292 | predicted gene 14292 [Source:MGI Symbol;Acc:MGI:3649964] | yes|down |
| ENSMUSG00000074527 | Gm14296 | predicted gene 14296 [Source:MGI Symbol;Acc:MGI:3708667] | yes|down |
| ENSMUSG00000026335 | Pam | peptidylglycine alpha-amidating monooxygenase [Source:MGI Symbol;Acc:MGI:97475] | yes|down |
| ENSMUSG00000024049 | Myom1 | myomesin 1 [Source:MGI Symbol;Acc:MGI:1341430] | yes|up |
| ENSMUSG00000022438 | Parvb | parvin, beta [Source:MGI Symbol;Acc:MGI:2153063] | yes|down |
| ENSMUSG00000105053 | Gm43064 | predicted gene 43064 [Source:MGI Symbol;Acc:MGI:5663201] | yes|up |
| ENSMUSG00000025789 | St8sia2 | ST8 alpha-N-acetyl-neuraminide alpha-2,8-sialyltransferase 2 [Source:MGI Symbol;Acc:MGI:106020] | yes|up |
| ENSMUSG00000025785 | Exosc7 | exosome component 7 [Source:MGI Symbol;Acc:MGI:1913696] | yes|down |
| ENSMUSG00000025780 | Itih5 | inter-alpha-trypsin inhibitor, heavy chain 5 [Source:MGI Symbol;Acc:MGI:1925751] | yes|down |
| ENSMUSG00000046410 | Kcnk6 | potassium inwardly-rectifying channel, subfamily K, member 6 [Source:MGI Symbol;Acc:MGI:1891291] | yes|up |
| ENSMUSG00000074336 | Apoc4 | apolipoprotein C-IV [Source:MGI Symbol;Acc:MGI:87878] | yes|up |
| ENSMUSG00000035504 | Reep6 | receptor accessory protein 6 [Source:MGI Symbol;Acc:MGI:1917585] | yes|up |
| ENSMUSG00000020493 | Prr11 | proline rich 11 [Source:MGI Symbol;Acc:MGI:2444496] | yes|down |
| ENSMUSG00000028378 | Ptgr1 | prostaglandin reductase 1 [Source:MGI Symbol;Acc:MGI:1914353] | yes|down |
| ENSMUSG00000028370 | Pappa | pregnancy-associated plasma protein A [Source:MGI Symbol;Acc:MGI:97479] | yes|up |
| ENSMUSG00000018381 | Abi3 | ABI family member 3 [Source:MGI Symbol;Acc:MGI:1913860] | yes|down |
| ENSMUSG00000070327 | Rnf213 | ring finger protein 213 [Source:MGI Symbol;Acc:MGI:1289196] | yes|up |
| ENSMUSG00000030774 | Pak1 | p21 (RAC1) activated kinase 1 [Source:MGI Symbol;Acc:MGI:1339975] | yes|down |
| ENSMUSG00000078521 | Aunip | aurora kinase A and ninein interacting protein [Source:MGI Symbol;Acc:MGI:1917135] | yes|down |
| ENSMUSG00000020140 | Lgr5 | leucine rich repeat containing G protein coupled receptor 5 [Source:MGI Symbol;Acc:MGI:1341817] | yes|up |
| ENSMUSG00000020142 | Slc1a4 | solute carrier family 1 (glutamate/neutral amino acid transporter), member 4 [Source:MGI Symbol;Acc:MGI:2135601] | yes|up |
| ENSMUSG00000020000 | Moxd1 | monooxygenase, DBH-like 1 [Source:MGI Symbol;Acc:MGI:1921582] | yes|up |
| ENSMUSG00000020007 | Il20ra | interleukin 20 receptor, alpha [Source:MGI Symbol;Acc:MGI:3605069] | yes|up |
| ENSMUSG00000022895 | Ets2 | E26 avian leukemia oncogene 2, 3' domain [Source:MGI Symbol;Acc:MGI:95456] | yes|up |
| ENSMUSG00000022894 | Adamts5 | a disintegrin-like and metallopeptidase (reprolysin type) with thrombospondin type 1 motif, 5 (aggrecanase-2) [Source:MGI Symbol;Acc:MGI:1346321] | yes|up |
| ENSMUSG00000018566 | Slc2a4 | solute carrier family 2 (facilitated glucose transporter), member 4 [Source:MGI Symbol;Acc:MGI:95758] | yes|up |
| ENSMUSG00000022893 | Adamts1 | a disintegrin-like and metallopeptidase (reprolysin type) with thrombospondin type 1 motif, 1 [Source:MGI Symbol;Acc:MGI:109249] | yes|up |
| ENSMUSG00000022892 | App | amyloid beta (A4) precursor protein [Source:MGI Symbol;Acc:MGI:88059] | yes|up |
| ENSMUSG00000017652 | Cd40 | CD40 antigen [Source:MGI Symbol;Acc:MGI:88336] | yes|up |
| ENSMUSG00000039081 | Zfp503 | zinc finger protein 503 [Source:MGI Symbol;Acc:MGI:1353644] | yes|up |
| ENSMUSG00000087303 | Lipo2 | lipase, member O2 [Source:MGI Symbol;Acc:MGI:3644466] | yes|down |
| ENSMUSG00000051817 | Sox12 | SRY (sex determining region Y)-box 12 [Source:MGI Symbol;Acc:MGI:98360] | yes|down |
| ENSMUSG00000032231 | Anxa2 | annexin A2 [Source:MGI Symbol;Acc:MGI:88246] | yes|down |
| ENSMUSG00000039883 | Lrrc17 | leucine rich repeat containing 17 [Source:MGI Symbol;Acc:MGI:1921761] | yes|up |
| ENSMUSG00000032238 | Rora | RAR-related orphan receptor alpha [Source:MGI Symbol;Acc:MGI:104661] | yes|up |
| ENSMUSG00000037628 | Cdkn3 | cyclin-dependent kinase inhibitor 3 [Source:MGI Symbol;Acc:MGI:1919641] | yes|down |
| ENSMUSG00000030909 | Anks4b | ankyrin repeat and sterile alpha motif domain containing 4B [Source:MGI Symbol;Acc:MGI:1919324] | yes|up |
| ENSMUSG00000037621 | Atoh8 | atonal bHLH transcription factor 8 [Source:MGI Symbol;Acc:MGI:1918343] | yes|down |
| ENSMUSG00000115867 | Gm17753 | predicted gene, 17753 [Source:MGI Symbol;Acc:MGI:5009838] | yes|up |
| ENSMUSG00000048142 | Nat8l | N-acetyltransferase 8-like [Source:MGI Symbol;Acc:MGI:2447776] | yes|up |
| ENSMUSG00000000266 | Mid2 | midline 2 [Source:MGI Symbol;Acc:MGI:1344333] | yes|up |
| ENSMUSG00000037482 | Erv3 | endogenous retroviral sequence 3 [Source:MGI Symbol;Acc:MGI:1919245] | yes|up |
| ENSMUSG00000061959 | Ces1e | carboxylesterase 1E [Source:MGI Symbol;Acc:MGI:95432] | yes|up |
| ENSMUSG00000041261 | Car8 | carbonic anhydrase 8 [Source:MGI Symbol;Acc:MGI:88253] | yes|up |
| ENSMUSG00000050368 | Hoxd10 | homeobox D10 [Source:MGI Symbol;Acc:MGI:96202] | yes|up |
| ENSMUSG00000041268 | Dmxl2 | Dmx-like 2 [Source:MGI Symbol;Acc:MGI:2444630] | yes|up |
| ENSMUSG00000043219 | Hoxa6 | homeobox A6 [Source:MGI Symbol;Acc:MGI:96178] | yes|up |
| ENSMUSG00000049281 | Scn3b | sodium channel, voltage-gated, type III, beta [Source:MGI Symbol;Acc:MGI:1918882] | yes|up |
| ENSMUSG00000038591 | Colec10 | collectin sub-family member 10 [Source:MGI Symbol;Acc:MGI:3606482] | yes|up |
| ENSMUSG00000005233 | Spc25 | SPC25, NDC80 kinetochore complex component, homolog (S. cerevisiae) [Source:MGI Symbol;Acc:MGI:1913692] | yes|down |
| ENSMUSG00000022157 | Mcpt8 | mast cell protease 8 [Source:MGI Symbol;Acc:MGI:1261780] | yes|down |
| ENSMUSG00000071793 | 2610005L07Rik | RIKEN cDNA 2610005L07 gene [Source:MGI Symbol;Acc:MGI:1914283] | yes|up |
| ENSMUSG00000041431 | Ccnb1 | cyclin B1 [Source:MGI Symbol;Acc:MGI:88302] | yes|down |
| ENSMUSG00000006585 | Cdt1 | chromatin licensing and DNA replication factor 1 [Source:MGI Symbol;Acc:MGI:1914427] | yes|down |
| ENSMUSG00000006586 | Runx1t1 | RUNX1 translocation partner 1 [Source:MGI Symbol;Acc:MGI:104793] | yes|up |
| ENSMUSG00000068962 | Zfp114 | zinc finger protein 114 [Source:MGI Symbol;Acc:MGI:3037815] | yes|down |
| ENSMUSG00000006638 | Abhd1 | abhydrolase domain containing 1 [Source:MGI Symbol;Acc:MGI:1931013] | yes|down |
| ENSMUSG00000057751 | Megf6 | multiple EGF-like-domains 6 [Source:MGI Symbol;Acc:MGI:1919351] | yes|up |
| ENSMUSG00000075590 | Nrbp2 | nuclear receptor binding protein 2 [Source:MGI Symbol;Acc:MGI:2385017] | yes|up |
| ENSMUSG00000003038 | Hmgn2 | high mobility group nucleosomal binding domain 2 [Source:MGI Symbol;Acc:MGI:96136] | yes|down |
| ENSMUSG00000075593 | Gal3st4 | galactose-3-O-sulfotransferase 4 [Source:MGI Symbol;Acc:MGI:1916254] | yes|up |
| ENSMUSG00000043635 | Adamts3 | a disintegrin-like and metallopeptidase (reprolysin type) with thrombospondin type 1 motif, 3 [Source:MGI Symbol;Acc:MGI:3045353] | yes|up |
| ENSMUSG00000043631 | Ecm2 | extracellular matrix protein 2, female organ and adipocyte specific [Source:MGI Symbol;Acc:MGI:3039578] | yes|up |
| ENSMUSG00000029765 | Plxna4 | plexin A4 [Source:MGI Symbol;Acc:MGI:2179061] | yes|up |
| ENSMUSG00000095912 | Gm3317 | predicted gene 3317 [Source:MGI Symbol;Acc:MGI:3781495] | yes|down |
| ENSMUSG00000028141 | Oaz3 | ornithine decarboxylase antizyme 3 [Source:MGI Symbol;Acc:MGI:1858170] | yes|down |
| ENSMUSG00000105345 | BC030343 | cDNA sequence BC030343 [Source:MGI Symbol;Acc:MGI:2679267] | yes|up |
| ENSMUSG00000038700 | Hoxb5 | homeobox B5 [Source:MGI Symbol;Acc:MGI:96186] | yes|up |
| ENSMUSG00000045777 | Ifitm10 | interferon induced transmembrane protein 10 [Source:MGI Symbol;Acc:MGI:2444776] | yes|down |
| ENSMUSG00000050965 | Prkca | protein kinase C, alpha [Source:MGI Symbol;Acc:MGI:97595] | yes|down |
| ENSMUSG00000024067 | Dpy30 | dpy-30, histone methyltransferase complex regulatory subunit [Source:MGI Symbol;Acc:MGI:1913560] | yes|down |
| ENSMUSG00000024065 | Ehd3 | EH-domain containing 3 [Source:MGI Symbol;Acc:MGI:1928900] | yes|up |
| ENSMUSG00000039384 | Dusp10 | dual specificity phosphatase 10 [Source:MGI Symbol;Acc:MGI:1927070] | yes|down |
| ENSMUSG00000068747 | Sort1 | sortilin 1 [Source:MGI Symbol;Acc:MGI:1338015] | yes|up |
| ENSMUSG00000015533 | Itga2 | integrin alpha 2 [Source:MGI Symbol;Acc:MGI:96600] | yes|down |
| ENSMUSG00000068748 | Ptprz1 | protein tyrosine phosphatase, receptor type Z, polypeptide 1 [Source:MGI Symbol;Acc:MGI:97816] | yes|up |
| ENSMUSG00000002325 | Irf9 | interferon regulatory factor 9 [Source:MGI Symbol;Acc:MGI:107587] | yes|up |
| ENSMUSG00000034422 | Parp14 | poly (ADP-ribose) polymerase family, member 14 [Source:MGI Symbol;Acc:MGI:1919489] | yes|up |
| ENSMUSG00000000673 | Haao | 3-hydroxyanthranilate 3,4-dioxygenase [Source:MGI Symbol;Acc:MGI:1349444] | yes|up |
| ENSMUSG00000107230 | Gm19265 | predicted gene, 19265 [Source:MGI Symbol;Acc:MGI:5011450] | yes|up |
| ENSMUSG00000117990 | Gm32027 | predicted gene, 32027 [Source:MGI Symbol;Acc:MGI:5591186] | yes|up |
| ENSMUSG00000071424 | Grid2 | glutamate receptor, ionotropic, delta 2 [Source:MGI Symbol;Acc:MGI:95813] | yes|up |
| ENSMUSG00000038692 | Hoxb4 | homeobox B4 [Source:MGI Symbol;Acc:MGI:96185] | yes|up |
| ENSMUSG00000001670 | Tat | tyrosine aminotransferase [Source:MGI Symbol;Acc:MGI:98487] | yes|up |
| ENSMUSG00000027261 | Hao1 | hydroxyacid oxidase 1, liver [Source:MGI Symbol;Acc:MGI:96011] | yes|up |
| ENSMUSG00000020949 | Fkbp3 | FK506 binding protein 3 [Source:MGI Symbol;Acc:MGI:1353460] | yes|down |
| ENSMUSG00000020948 | Klhl28 | kelch-like 28 [Source:MGI Symbol;Acc:MGI:1913939] | yes|up |
| ENSMUSG00000033467 | Crlf2 | cytokine receptor-like factor 2 [Source:MGI Symbol;Acc:MGI:1889506] | yes|up |
| ENSMUSG00000101174 | Hoxd4 | homeobox D4 [Source:MGI Symbol;Acc:MGI:96208] | yes|up |
| ENSMUSG00000000094 | Tbx4 | T-box 4 [Source:MGI Symbol;Acc:MGI:102556] | yes|down |
| ENSMUSG00000037542 | Aldh8a1 | aldehyde dehydrogenase 8 family, member A1 [Source:MGI Symbol;Acc:MGI:2653900] | yes|up |
| ENSMUSG00000030041 | M1ap | meiosis 1 associated protein [Source:MGI Symbol;Acc:MGI:1315200] | yes|down |
| ENSMUSG00000037544 | Dlgap5 | DLG associated protein 5 [Source:MGI Symbol;Acc:MGI:2183453] | yes|down |
| ENSMUSG00000037010 | Apln | apelin [Source:MGI Symbol;Acc:MGI:1353624] | yes|down |
| ENSMUSG00000019590 | Cyb561 | cytochrome b-561 [Source:MGI Symbol;Acc:MGI:103253] | yes|down |
| ENSMUSG00000040658 | Dnph1 | 2'-deoxynucleoside 5'-phosphate N-hydrolase 1 [Source:MGI Symbol;Acc:MGI:3039376] | yes|down |
| ENSMUSG00000041923 | Nol4 | nucleolar protein 4 [Source:MGI Symbol;Acc:MGI:2441684] | yes|down |
| ENSMUSG00000041926 | Rnpep | arginyl aminopeptidase (aminopeptidase B) [Source:MGI Symbol;Acc:MGI:2384902] | yes|down |
| ENSMUSG00000021268 | Meg3 | maternally expressed 3 [Source:MGI Symbol;Acc:MGI:1202886] | yes|up |
| ENSMUSG00000026683 | Nuf2 | NUF2, NDC80 kinetochore complex component [Source:MGI Symbol;Acc:MGI:1914227] | yes|down |
| ENSMUSG00000023411 | Nfatc4 | nuclear factor of activated T cells, cytoplasmic, calcineurin dependent 4 [Source:MGI Symbol;Acc:MGI:1920431] | yes|up |
| ENSMUSG00000026688 | Mgst3 | microsomal glutathione S-transferase 3 [Source:MGI Symbol;Acc:MGI:1913697] | yes|down |
| ENSMUSG00000040037 | Negr1 | neuronal growth regulator 1 [Source:MGI Symbol;Acc:MGI:2444846] | yes|down |
| ENSMUSG00000040034 | Nup43 | nucleoporin 43 [Source:MGI Symbol;Acc:MGI:1917162] | yes|down |
| ENSMUSG00000040033 | Stat2 | signal transducer and activator of transcription 2 [Source:MGI Symbol;Acc:MGI:103039] | yes|up |
| ENSMUSG00000034163 | Zfc3h1 | zinc finger, C3H1-type containing [Source:MGI Symbol;Acc:MGI:2446143] | yes|up |
| ENSMUSG00000034161 | Scx | scleraxis [Source:MGI Symbol;Acc:MGI:102934] | yes|up |
| ENSMUSG00000074505 | Fat3 | FAT atypical cadherin 3 [Source:MGI Symbol;Acc:MGI:2444314] | yes|up |
| ENSMUSG00000074506 | Gm10705 | predicted gene 10705 [Source:MGI Symbol;Acc:MGI:3708678] | yes|down |
| ENSMUSG00000020953 | Coch | cochlin [Source:MGI Symbol;Acc:MGI:1278313] | yes|down |
| ENSMUSG00000029153 | Ociad2 | OCIA domain containing 2 [Source:MGI Symbol;Acc:MGI:1916377] | yes|down |
| ENSMUSG00000026355 | Mcm6 | minichromosome maintenance complex component 6 [Source:MGI Symbol;Acc:MGI:1298227] | yes|down |
| ENSMUSG00000062296 | Trank1 | tetratricopeptide repeat and ankyrin repeat containing 1 [Source:MGI Symbol;Acc:MGI:1341834] | yes|up |
| ENSMUSG00000007041 | Clic1 | chloride intracellular channel 1 [Source:MGI Symbol;Acc:MGI:2148924] | yes|down |
| ENSMUSG00000029379 | Cxcl3 | chemokine (C-X-C motif) ligand 3 [Source:MGI Symbol;Acc:MGI:3037818] | yes|down |
| ENSMUSG00000029378 | Areg | amphiregulin [Source:MGI Symbol;Acc:MGI:88068] | yes|down |
| ENSMUSG00000022454 | Nell2 | NEL-like 2 [Source:MGI Symbol;Acc:MGI:1858510] | yes|up |
| ENSMUSG00000029377 | Ereg | epiregulin [Source:MGI Symbol;Acc:MGI:107508] | yes|down |
| ENSMUSG00000029371 | Cxcl5 | chemokine (C-X-C motif) ligand 5 [Source:MGI Symbol;Acc:MGI:1096868] | yes|down |
| ENSMUSG00000029370 | Rassf6 | Ras association (RalGDS/AF-6) domain family member 6 [Source:MGI Symbol;Acc:MGI:1920496] | yes|down |
| ENSMUSG00000029372 | Ppbp | pro-platelet basic protein [Source:MGI Symbol;Acc:MGI:1888712] | yes|down |
| ENSMUSG00000020475 | Pgam2 | phosphoglycerate mutase 2 [Source:MGI Symbol;Acc:MGI:1933118] | yes|up |
| ENSMUSG00000047562 | Mmp10 | matrix metallopeptidase 10 [Source:MGI Symbol;Acc:MGI:97007] | yes|up |
| ENSMUSG00000015890 | Amdhd1 | amidohydrolase domain containing 1 [Source:MGI Symbol;Acc:MGI:1919011] | yes|up |
| ENSMUSG00000098158 | Gm4804 | predicted gene 4804 [Source:MGI Symbol;Acc:MGI:3646648] | yes|down |
| ENSMUSG00000034785 | Dio1 | deiodinase, iodothyronine, type I [Source:MGI Symbol;Acc:MGI:94896] | yes|up |
| ENSMUSG00000049871 | Nlrc3 | NLR family, CARD domain containing 3 [Source:MGI Symbol;Acc:MGI:2444070] | yes|down |
| ENSMUSG00000029260 | Ugt2b34 | UDP glucuronosyltransferase 2 family, polypeptide B34 [Source:MGI Symbol;Acc:MGI:2140962] | yes|up |
| ENSMUSG00000035566 | Pcdh17 | protocadherin 17 [Source:MGI Symbol;Acc:MGI:2684924] | yes|up |
| ENSMUSG00000020160 | Meis1 | Meis homeobox 1 [Source:MGI Symbol;Acc:MGI:104717] | yes|up |
| ENSMUSG00000064043 | Trerf1 | transcriptional regulating factor 1 [Source:MGI Symbol;Acc:MGI:2442086] | yes|down |
| ENSMUSG00000028356 | Ambp | alpha 1 microglobulin/bikunin precursor [Source:MGI Symbol;Acc:MGI:88002] | yes|up |
| ENSMUSG00000025105 | Bnc1 | basonuclin 1 [Source:MGI Symbol;Acc:MGI:1097164] | yes|down |
| ENSMUSG00000016018 | Mtrex | Mtr4 exosome RNA helicase [Source:MGI Symbol;Acc:MGI:1919448] | yes|down |
| ENSMUSG00000035783 | Acta2 | actin alpha 2, smooth muscle, aorta [Source:MGI Symbol;Acc:MGI:87909] | yes|up |
| ENSMUSG00000078503 | Zfp990 | zinc finger protein 990 [Source:MGI Symbol;Acc:MGI:3652161] | yes|up |
| ENSMUSG00000087143 | A830082K12Rik | RIKEN cDNA A830082K12 gene [Source:MGI Symbol;Acc:MGI:2443527] | yes|down |
| ENSMUSG00000017639 | Rab11fip4 | RAB11 family interacting protein 4 (class II) [Source:MGI Symbol;Acc:MGI:2442920] | yes|up |
| ENSMUSG00000018509 | Cenpv | centromere protein V [Source:MGI Symbol;Acc:MGI:1920389] | yes|down |
| ENSMUSG00000080115 | Eef1akmt3 | EEF1A lysine methyltransferase 3 [Source:MGI Symbol;Acc:MGI:3645330] | yes|up |
| ENSMUSG00000032783 | Troap | trophinin associated protein [Source:MGI Symbol;Acc:MGI:1925983] | yes|down |
| ENSMUSG00000107535 | Gm44183 | predicted gene, 44183 [Source:MGI Symbol;Acc:MGI:5690575] | yes|up |
| ENSMUSG00000019368 | Sec14l4 | SEC14-like lipid binding 4 [Source:MGI Symbol;Acc:MGI:2144095] | yes|up |
| ENSMUSG00000087366 | Junos | jun proto-oncogene, opposite strand [Source:MGI Symbol;Acc:MGI:2652837] | yes|down |
| ENSMUSG00000048387 | Osr1 | odd-skipped related transcription factor 1 [Source:MGI Symbol;Acc:MGI:1344424] | yes|down |
| ENSMUSG00000033256 | Shf | Src homology 2 domain containing F [Source:MGI Symbol;Acc:MGI:3613669] | yes|down |
| ENSMUSG00000024479 | Mal2 | mal, T cell differentiation protein 2 [Source:MGI Symbol;Acc:MGI:2146021] | yes|up |
| ENSMUSG00000032257 | Ankk1 | ankyrin repeat and kinase domain containing 1 [Source:MGI Symbol;Acc:MGI:3045301] | yes|down |
| ENSMUSG00000032254 | Kif23 | kinesin family member 23 [Source:MGI Symbol;Acc:MGI:1919069] | yes|down |
| ENSMUSG00000074280 | Gm6166 | predicted gene 6166 [Source:MGI Symbol;Acc:MGI:3645893] | yes|up |
| ENSMUSG00000032252 | Glce | glucuronyl C5-epimerase [Source:MGI Symbol;Acc:MGI:2136405] | yes|up |
| ENSMUSG00000071073 | Lrrc73 | leucine rich repeat containing 73 [Source:MGI Symbol;Acc:MGI:2684934] | yes|down |
| ENSMUSG00000000402 | Egfl6 | EGF-like-domain, multiple 6 [Source:MGI Symbol;Acc:MGI:1858599] | yes|up |
| ENSMUSG00000019102 | Aldh3a1 | aldehyde dehydrogenase family 3, subfamily A1 [Source:MGI Symbol;Acc:MGI:1353451] | yes|down |
| ENSMUSG00000079015 | Serpina1c | serine (or cysteine) peptidase inhibitor, clade A, member 1C [Source:MGI Symbol;Acc:MGI:891969] | yes|up |
| ENSMUSG00000079014 | Serpina3i | serine (or cysteine) peptidase inhibitor, clade A, member 3I [Source:MGI Symbol;Acc:MGI:2182841] | yes|up |
| ENSMUSG00000000247 | Lhx2 | LIM homeobox protein 2 [Source:MGI Symbol;Acc:MGI:96785] | yes|down |
| ENSMUSG00000005360 | Slc1a3 | solute carrier family 1 (glial high affinity glutamate transporter), member 3 [Source:MGI Symbol;Acc:MGI:99917] | yes|down |
| ENSMUSG00000024210 | Ip6k3 | inositol hexaphosphate kinase 3 [Source:MGI Symbol;Acc:MGI:3045325] | yes|down |
| ENSMUSG00000079012 | Serpina3m | serine (or cysteine) peptidase inhibitor, clade A, member 3M [Source:MGI Symbol;Acc:MGI:98378] | yes|up |
| ENSMUSG00000049128 | Ivl | involucrin [Source:MGI Symbol;Acc:MGI:96626] | yes|up |
| ENSMUSG00000054863 | Tafa5 | TAFA chemokine like family member 5 [Source:MGI Symbol;Acc:MGI:2146182] | yes|down |
| ENSMUSG00000004099 | Dnmt1 | DNA methyltransferase (cytosine-5) 1 [Source:MGI Symbol;Acc:MGI:94912] | yes|down |
| ENSMUSG00000004098 | Col5a3 | collagen, type V, alpha 3 [Source:MGI Symbol;Acc:MGI:1858212] | yes|up |
| ENSMUSG00000050345 | 4930486L24Rik | RIKEN cDNA 4930486L24 gene [Source:MGI Symbol;Acc:MGI:1922258] | yes|up |
| ENSMUSG00000005148 | Klf5 | Kruppel-like factor 5 [Source:MGI Symbol;Acc:MGI:1338056] | yes|down |
| ENSMUSG00000038068 | Rnf144b | ring finger protein 144B [Source:MGI Symbol;Acc:MGI:2384986] | yes|up |
| ENSMUSG00000030522 | Mtmr10 | myotubularin related protein 10 [Source:MGI Symbol;Acc:MGI:2142292] | yes|down |
| ENSMUSG00000030523 | Trpm1 | transient receptor potential cation channel, subfamily M, member 1 [Source:MGI Symbol;Acc:MGI:1330305] | yes|up |
| ENSMUSG00000030254 | Rad18 | RAD18 E3 ubiquitin protein ligase [Source:MGI Symbol;Acc:MGI:1890476] | yes|down |
| ENSMUSG00000030525 | Chrna7 | cholinergic receptor, nicotinic, alpha polypeptide 7 [Source:MGI Symbol;Acc:MGI:99779] | yes|up |
| ENSMUSG00000030528 | Blm | Bloom syndrome, RecQ like helicase [Source:MGI Symbol;Acc:MGI:1328362] | yes|down |
| ENSMUSG00000059195 | Gm12715 | predicted gene 12715 [Source:MGI Symbol;Acc:MGI:3650759] | yes|down |
| ENSMUSG00000070704 | Ugt2b36 | UDP glucuronosyltransferase 2 family, polypeptide B36 [Source:MGI Symbol;Acc:MGI:3576103] | yes|up |
| ENSMUSG00000121258 |  | novel transcript | yes|down |
| ENSMUSG00000027748 | Trpc4 | transient receptor potential cation channel, subfamily C, member 4 [Source:MGI Symbol;Acc:MGI:109525] | yes|up |
| ENSMUSG00000056648 | Hoxb8 | homeobox B8 [Source:MGI Symbol;Acc:MGI:96189] | yes|up |
| ENSMUSG00000106190 | Gm20768 | predicted gene, 20768 [Source:MGI Symbol;Acc:MGI:5434124] | yes|down |
| ENSMUSG00000031785 | Adgrg1 | adhesion G protein-coupled receptor G1 [Source:MGI Symbol;Acc:MGI:1340051] | yes|down |
| ENSMUSG00000057596 | Trim30d | tripartite motif-containing 30D [Source:MGI Symbol;Acc:MGI:3035181] | yes|down |
| ENSMUSG00000056427 | Slit3 | slit guidance ligand 3 [Source:MGI Symbol;Acc:MGI:1315202] | yes|up |
| ENSMUSG00000051439 | Cd14 | CD14 antigen [Source:MGI Symbol;Acc:MGI:88318] | yes|down |
| ENSMUSG00000056429 | Tgoln1 | trans-golgi network protein [Source:MGI Symbol;Acc:MGI:105080] | yes|up |
| ENSMUSG00000090215 | Trim34b | tripartite motif-containing 34B [Source:MGI Symbol;Acc:MGI:4821264] | yes|up |
| ENSMUSG00000031129 | Slc9a9 | solute carrier family 9 (sodium/hydrogen exchanger), member 9 [Source:MGI Symbol;Acc:MGI:2679732] | yes|up |
| ENSMUSG00000034258 | Flvcr2 | feline leukemia virus subgroup C cellular receptor 2 [Source:MGI Symbol;Acc:MGI:2384974] | yes|up |
| ENSMUSG00000048450 | Msx1 | msh homeobox 1 [Source:MGI Symbol;Acc:MGI:97168] | yes|down |
| ENSMUSG00000034573 | Ptpn13 | protein tyrosine phosphatase, non-receptor type 13 [Source:MGI Symbol;Acc:MGI:103293] | yes|down |
| ENSMUSG00000039252 | Lgi2 | leucine-rich repeat LGI family, member 2 [Source:MGI Symbol;Acc:MGI:2180196] | yes|up |
| ENSMUSG00000039257 | Vstm2b | V-set and transmembrane domain containing 2B [Source:MGI Symbol;Acc:MGI:1914525] | yes|up |
| ENSMUSG00000084128 | Esrp2 | epithelial splicing regulatory protein 2 [Source:MGI Symbol;Acc:MGI:1924661] | yes|up |
| ENSMUSG00000034317 | Trim59 | tripartite motif-containing 59 [Source:MGI Symbol;Acc:MGI:1914199] | yes|down |
| ENSMUSG00000028164 | Manba | mannosidase, beta A, lysosomal [Source:MGI Symbol;Acc:MGI:88175] | yes|up |
| ENSMUSG00000034311 | Kif4 | kinesin family member 4 [Source:MGI Symbol;Acc:MGI:108389] | yes|down |
| ENSMUSG00000025665 | Rps6ka6 | ribosomal protein S6 kinase polypeptide 6 [Source:MGI Symbol;Acc:MGI:1914321] | yes|down |
| ENSMUSG00000021175 | Cdca7l | cell division cycle associated 7 like [Source:MGI Symbol;Acc:MGI:2384982] | yes|down |
| ENSMUSG00000046591 | Ticrr | TOPBP1-interacting checkpoint and replication regulator [Source:MGI Symbol;Acc:MGI:1924261] | yes|down |
| ENSMUSG00000046598 | Bdh1 | 3-hydroxybutyrate dehydrogenase, type 1 [Source:MGI Symbol;Acc:MGI:1919161] | yes|up |
| ENSMUSG00000103887 | Gm37008 | predicted gene, 37008 [Source:MGI Symbol;Acc:MGI:5610236] | yes|up |
| ENSMUSG00000021569 | Trip13 | thyroid hormone receptor interactor 13 [Source:MGI Symbol;Acc:MGI:1916966] | yes|down |
| ENSMUSG00000046623 | Gjb4 | gap junction protein, beta 4 [Source:MGI Symbol;Acc:MGI:95722] | yes|down |
| ENSMUSG00000021567 | Nkd2 | naked cuticle 2 [Source:MGI Symbol;Acc:MGI:1919543] | yes|up |
| ENSMUSG00000052854 | Nrk | Nik related kinase [Source:MGI Symbol;Acc:MGI:1351326] | yes|up |
| ENSMUSG00000035041 | Creb3l3 | cAMP responsive element binding protein 3-like 3 [Source:MGI Symbol;Acc:MGI:2384786] | yes|up |
| ENSMUSG00000045822 | Zswim3 | zinc finger SWIM-type containing 3 [Source:MGI Symbol;Acc:MGI:1914788] | yes|up |
| ENSMUSG00000110386 | Gm42031 | predicted gene, 42031 [Source:MGI Symbol;Acc:MGI:5624916] | yes|up |
| ENSMUSG00000019823 | Mical1 | microtubule associated monooxygenase, calponin and LIM domain containing 1 [Source:MGI Symbol;Acc:MGI:2385847] | yes|up |
| ENSMUSG00000036782 | Klhl13 | kelch-like 13 [Source:MGI Symbol;Acc:MGI:1914705] | yes|up |
| ENSMUSG00000001657 | Hoxc8 | homeobox C8 [Source:MGI Symbol;Acc:MGI:96198] | yes|up |
| ENSMUSG00000010080 | Epn3 | epsin 3 [Source:MGI Symbol;Acc:MGI:1919139] | yes|down |
| ENSMUSG00000027249 | F2 | coagulation factor II [Source:MGI Symbol;Acc:MGI:88380] | yes|up |
| ENSMUSG00000027536 | Chmp4c | charged multivesicular body protein 4C [Source:MGI Symbol;Acc:MGI:1913621] | yes|down |
| ENSMUSG00000033446 | Lpar6 | lysophosphatidic acid receptor 6 [Source:MGI Symbol;Acc:MGI:1914418] | yes|down |
| ENSMUSG00000014704 | Hoxa2 | homeobox A2 [Source:MGI Symbol;Acc:MGI:96174] | yes|up |
| ENSMUSG00000030064 | Frmd4b | FERM domain containing 4B [Source:MGI Symbol;Acc:MGI:2141794] | yes|down |
| ENSMUSG00000115431 | Gm3219 | predicted pseudogene 3219 [Source:MGI Symbol;Acc:MGI:3781398] | yes|down |
| ENSMUSG00000041907 | Gpr45 | G protein-coupled receptor 45 [Source:MGI Symbol;Acc:MGI:2135882] | yes|down |
| ENSMUSG00000019647 | Sema6a | sema domain, transmembrane domain (TM), and cytoplasmic domain, (semaphorin) 6A [Source:MGI Symbol;Acc:MGI:1203727] | yes|up |
| ENSMUSG00000021364 | Elovl2 | elongation of very long chain fatty acids (FEN1/Elo2, SUR4/Elo3, yeast)-like 2 [Source:MGI Symbol;Acc:MGI:1858960] | yes|up |
| ENSMUSG00000021365 | Nedd9 | neural precursor cell expressed, developmentally down-regulated gene 9 [Source:MGI Symbol;Acc:MGI:97302] | yes|up |
| ENSMUSG00000021368 | Tbc1d7 | TBC1 domain family, member 7 [Source:MGI Symbol;Acc:MGI:1914296] | yes|down |
| ENSMUSG00000029177 | Cenpa | centromere protein A [Source:MGI Symbol;Acc:MGI:88375] | yes|down |
| ENSMUSG00000047501 | Cldn4 | claudin 4 [Source:MGI Symbol;Acc:MGI:1313314] | yes|up |
| ENSMUSG00000019987 | Arg1 | arginase, liver [Source:MGI Symbol;Acc:MGI:88070] | yes|up |
| ENSMUSG00000022479 | Vdr | vitamin D (1,25-dihydroxyvitamin D3) receptor [Source:MGI Symbol;Acc:MGI:103076] | yes|down |
| ENSMUSG00000035237 | Lcat | lecithin cholesterol acyltransferase [Source:MGI Symbol;Acc:MGI:96755] | yes|up |
| ENSMUSG00000035540 | Gc | vitamin D binding protein [Source:MGI Symbol;Acc:MGI:95669] | yes|up |
| ENSMUSG00000020181 | Nav3 | neuron navigator 3 [Source:MGI Symbol;Acc:MGI:2183703] | yes|down |
| ENSMUSG00000020180 | Snrpd3 | small nuclear ribonucleoprotein D3 [Source:MGI Symbol;Acc:MGI:1914582] | yes|down |
| ENSMUSG00000020182 | Ddc | dopa decarboxylase [Source:MGI Symbol;Acc:MGI:94876] | yes|up |
| ENSMUSG00000020185 | E2f7 | E2F transcription factor 7 [Source:MGI Symbol;Acc:MGI:1289147] | yes|down |
| ENSMUSG00000025127 | Gcgr | glucagon receptor [Source:MGI Symbol;Acc:MGI:99572] | yes|up |
| ENSMUSG00000064023 | Klk8 | kallikrein related-peptidase 8 [Source:MGI Symbol;Acc:MGI:1343327] | yes|down |
| ENSMUSG00000025128 | Bhlhe22 | basic helix-loop-helix family, member e22 [Source:MGI Symbol;Acc:MGI:1930001] | yes|down |
| ENSMUSG00000109953 | 5430430B14Rik | RIKEN cDNA 5430430B14 gene [Source:MGI Symbol;Acc:MGI:1918674] | yes|up |
| ENSMUSG00000049811 | Fam161a | family with sequence similarity 161, member A [Source:MGI Symbol;Acc:MGI:1921123] | yes|up |
| ENSMUSG00000066554 | Gm10167 | predicted pseudogene 10167 [Source:MGI Symbol;Acc:MGI:3704263] | yes|up |
| ENSMUSG00000001467 | Cyp51 | cytochrome P450, family 51 [Source:MGI Symbol;Acc:MGI:106040] | yes|up |
| ENSMUSG00000063698 | Sfxn4 | sideroflexin 4 [Source:MGI Symbol;Acc:MGI:2137680] | yes|down |
| ENSMUSG00000045103 | Dmd | dystrophin, muscular dystrophy [Source:MGI Symbol;Acc:MGI:94909] | yes|up |
| ENSMUSG00000045104 | Ldhb-ps | lactate dehydrogenase B, pseudogene [Source:MGI Symbol;Acc:MGI:3645435] | yes|up |
| ENSMUSG00000063696 | Gm8730 | predicted pseudogene 8730 [Source:MGI Symbol;Acc:MGI:3644565] | yes|down |
| ENSMUSG00000032014 | Oaf | out at first homolog [Source:MGI Symbol;Acc:MGI:94852] | yes|down |
| ENSMUSG00000110841 | Gpx4-ps2 | glutathione peroxidase 4, pseudogene 2 [Source:MGI Symbol;Acc:MGI:3779731] | yes|down |
| ENSMUSG00000078922 | Tgtp1 | T cell specific GTPase 1 [Source:MGI Symbol;Acc:MGI:98734] | yes|up |
| ENSMUSG00000002847 | Pla1a | phospholipase A1 member A [Source:MGI Symbol;Acc:MGI:1934677] | yes|up |
| ENSMUSG00000079092 | Prl2c2 | prolactin family 2, subfamily c, member 2 [Source:MGI Symbol;Acc:MGI:97618] | yes|down |
| ENSMUSG00000079037 | Prnp | prion protein [Source:MGI Symbol;Acc:MGI:97769] | yes|up |
| ENSMUSG00000050107 | Haspin | histone H3 associated protein kinase [Source:MGI Symbol;Acc:MGI:1194498] | yes|down |
| ENSMUSG00000049107 | Ntf3 | neurotrophin 3 [Source:MGI Symbol;Acc:MGI:97380] | yes|up |
| ENSMUSG00000049100 | Pcdh10 | protocadherin 10 [Source:MGI Symbol;Acc:MGI:1338042] | yes|up |
| ENSMUSG00000024232 | Bambi | BMP and activin membrane-bound inhibitor [Source:MGI Symbol;Acc:MGI:1915260] | yes|down |
| ENSMUSG00000024989 | Cep55 | centrosomal protein 55 [Source:MGI Symbol;Acc:MGI:1921357] | yes|down |
| ENSMUSG00000032902 | Slc16a1 | solute carrier family 16 (monocarboxylic acid transporters), member 1 [Source:MGI Symbol;Acc:MGI:106013] | yes|down |
| ENSMUSG00000026589 | Sec16b | SEC16 homolog B (S. cerevisiae) [Source:MGI Symbol;Acc:MGI:2148802] | yes|up |
| ENSMUSG00000026582 | Sele | selectin, endothelial cell [Source:MGI Symbol;Acc:MGI:98278] | yes|down |
| ENSMUSG00000059422 | Gm8116 | predicted gene 8116 [Source:MGI Symbol;Acc:MGI:3648797] | yes|down |
| ENSMUSG00000121491 | 2700099C18Rik | NDC80 homolog, kinetochore complex component pseudogene [Source:NCBI gene (formerly Entrezgene);Acc:77022] | yes|down |
| ENSMUSG00000027188 | Pamr1 | peptidase domain containing associated with muscle regeneration 1 [Source:MGI Symbol;Acc:MGI:2445082] | yes|up |
| ENSMUSG00000041731 | Pgm5 | phosphoglucomutase 5 [Source:MGI Symbol;Acc:MGI:1925668] | yes|up |
| ENSMUSG00000038085 | Cnbd2 | cyclic nucleotide binding domain containing 2 [Source:MGI Symbol;Acc:MGI:1918123] | yes|up |
| ENSMUSG00000038086 | Hspb2 | heat shock protein 2 [Source:MGI Symbol;Acc:MGI:1916503] | yes|up |
| ENSMUSG00000030500 | Slc17a6 | solute carrier family 17 (sodium-dependent inorganic phosphate cotransporter), member 6 [Source:MGI Symbol;Acc:MGI:2156052] | yes|down |
| ENSMUSG00000097762 | 4732463B04Rik | RIKEN cDNA 4732463B04 gene [Source:MGI Symbol;Acc:MGI:3642483] | yes|up |
| ENSMUSG00000027765 | P2ry1 | purinergic receptor P2Y, G-protein coupled 1 [Source:MGI Symbol;Acc:MGI:105049] | yes|up |
| ENSMUSG00000070720 | Tmem200b | transmembrane protein 200B [Source:MGI Symbol;Acc:MGI:3646343] | yes|down |
| ENSMUSG00000027761 | Aadac | arylacetamide deacetylase [Source:MGI Symbol;Acc:MGI:1915008] | yes|up |
| ENSMUSG00000066319 | Rtp3 | receptor transporter protein 3 [Source:MGI Symbol;Acc:MGI:2446841] | yes|up |
| ENSMUSG00000022111 | Uchl3 | ubiquitin carboxyl-terminal esterase L3 (ubiquitin thiolesterase) [Source:MGI Symbol;Acc:MGI:1355274] | yes|down |
| ENSMUSG00000022114 | Spry2 | sprouty RTK signaling antagonist 2 [Source:MGI Symbol;Acc:MGI:1345138] | yes|down |
| ENSMUSG00000091900 | Gm4353 | predicted gene 4353 [Source:MGI Symbol;Acc:MGI:3782538] | yes|down |
| ENSMUSG00000039680 | Mrps6 | mitochondrial ribosomal protein S6 [Source:MGI Symbol;Acc:MGI:2153111] | yes|down |
| ENSMUSG00000039683 | Sdk1 | sidekick cell adhesion molecule 1 [Source:MGI Symbol;Acc:MGI:2444413] | yes|down |
| ENSMUSG00000031109 | Enox2 | ecto-NOX disulfide-thiol exchanger 2 [Source:MGI Symbol;Acc:MGI:2384799] | yes|down |
| ENSMUSG00000090231 | Cfb | complement factor B [Source:MGI Symbol;Acc:MGI:105975] | yes|up |
| ENSMUSG00000031101 | Sash3 | SAM and SH3 domain containing 3 [Source:MGI Symbol;Acc:MGI:1921381] | yes|up |
| ENSMUSG00000031107 | Rbmx2 | RNA binding motif protein, X-linked 2 [Source:MGI Symbol;Acc:MGI:1919414] | yes|down |
| ENSMUSG00000108942 | Gm44660 | predicted gene 44660 [Source:MGI Symbol;Acc:MGI:5753236] | yes|up |
| ENSMUSG00000043673 | Kcns3 | potassium voltage-gated channel, delayed-rectifier, subfamily S, member 3 [Source:MGI Symbol;Acc:MGI:1098804] | yes|up |
| ENSMUSG00000027460 | Angpt4 | angiopoietin 4 [Source:MGI Symbol;Acc:MGI:1336887] | yes|up |
| ENSMUSG00000039232 | Stx11 | syntaxin 11 [Source:MGI Symbol;Acc:MGI:1921982] | yes|up |
| ENSMUSG00000039239 | Tgfb2 | transforming growth factor, beta 2 [Source:MGI Symbol;Acc:MGI:98726] | yes|up |
| ENSMUSG00000089756 | Zfp966 | zinc finger protein 966 [Source:MGI Symbol;Acc:MGI:3709288] | yes|down |
| ENSMUSG00000034881 | Tbxa2r | thromboxane A2 receptor [Source:MGI Symbol;Acc:MGI:98496] | yes|up |
| ENSMUSG00000034883 | Lrr1 | leucine rich repeat protein 1 [Source:MGI Symbol;Acc:MGI:1916956] | yes|down |
| ENSMUSG00000021115 | Vrk1 | vaccinia related kinase 1 [Source:MGI Symbol;Acc:MGI:1261847] | yes|down |
| ENSMUSG00000050926 | Dcaf12l2 | DDB1 and CUL4 associated factor 12-like 2 [Source:MGI Symbol;Acc:MGI:2445178] | yes|up |
| ENSMUSG00000032487 | Ptgs2 | prostaglandin-endoperoxide synthase 2 [Source:MGI Symbol;Acc:MGI:97798] | yes|down |
| ENSMUSG00000042109 | Csdc2 | cold shock domain containing C2, RNA binding [Source:MGI Symbol;Acc:MGI:2146027] | yes|up |
| ENSMUSG00000035681 | Kcnc2 | potassium voltage gated channel, Shaw-related subfamily, member 2 [Source:MGI Symbol;Acc:MGI:96668] | yes|down |
| ENSMUSG00000035683 | Melk | maternal embryonic leucine zipper kinase [Source:MGI Symbol;Acc:MGI:106924] | yes|down |
| ENSMUSG00000042102 | Dmgdh | dimethylglycine dehydrogenase precursor [Source:MGI Symbol;Acc:MGI:1921379] | yes|up |
| ENSMUSG00000061232 | H2-K1 | histocompatibility 2, K1, K region [Source:MGI Symbol;Acc:MGI:95904] | yes|up |
| ENSMUSG00000120319 |  | novel transcript, antisense to Tpk1and KO:Tpk1 | yes|up |
| ENSMUSG00000020642 | Rnf144a | ring finger protein 144A [Source:MGI Symbol;Acc:MGI:1344401] | yes|up |
| ENSMUSG00000036019 | Tmtc2 | transmembrane and tetratricopeptide repeat containing 2 [Source:MGI Symbol;Acc:MGI:1914057] | yes|up |
| ENSMUSG00000020641 | Rsad2 | radical S-adenosyl methionine domain containing 2 [Source:MGI Symbol;Acc:MGI:1929628] | yes|up |
| ENSMUSG00000020649 | Rrm2 | ribonucleotide reductase M2 [Source:MGI Symbol;Acc:MGI:98181] | yes|down |
| ENSMUSG00000039908 | Slc26a11 | solute carrier family 26, member 11 [Source:MGI Symbol;Acc:MGI:2444589] | yes|up |
| ENSMUSG00000039903 | Eva1c | eva-1 homolog C (C. elegans) [Source:MGI Symbol;Acc:MGI:1918217] | yes|down |
| ENSMUSG00000078249 | Hmga1b | high mobility group AT-hook 1B [Source:MGI Symbol;Acc:MGI:96161] | yes|down |
| ENSMUSG00000078247 | Airn | antisense Igf2r RNA [Source:MGI Symbol;Acc:MGI:1353471] | yes|up |
| ENSMUSG00000027514 | Zbp1 | Z-DNA binding protein 1 [Source:MGI Symbol;Acc:MGI:1927449] | yes|up |
| ENSMUSG00000058290 | Espl1 | extra spindle pole bodies 1, separase [Source:MGI Symbol;Acc:MGI:2146156] | yes|down |
| ENSMUSG00000017400 | Stac2 | SH3 and cysteine rich domain 2 [Source:MGI Symbol;Acc:MGI:2144518] | yes|down |
| ENSMUSG00000033429 | Mcee | methylmalonyl CoA epimerase [Source:MGI Symbol;Acc:MGI:1920974] | yes|down |
| ENSMUSG00000033427 | Upb1 | ureidopropionase, beta [Source:MGI Symbol;Acc:MGI:2143535] | yes|up |
| ENSMUSG00000096370 | Gm21992 | predicted gene 21992 [Source:MGI Symbol;Acc:MGI:5439461] | yes|up |
| ENSMUSG00000033420 | Antxr1 | anthrax toxin receptor 1 [Source:MGI Symbol;Acc:MGI:1916788] | yes|up |
| ENSMUSG00000041696 | Rasl12 | RAS-like, family 12 [Source:MGI Symbol;Acc:MGI:1918034] | yes|up |
| ENSMUSG00000050737 | Ptges | prostaglandin E synthase [Source:MGI Symbol;Acc:MGI:1927593] | yes|down |
| ENSMUSG00000101609 | Kcnq1ot1 | KCNQ1 overlapping transcript 1 [Source:MGI Symbol;Acc:MGI:1926855] | yes|up |
| ENSMUSG00000029093 | Sorcs2 | sortilin-related VPS10 domain containing receptor 2 [Source:MGI Symbol;Acc:MGI:1932289] | yes|up |
| ENSMUSG00000040618 | Pck2 | phosphoenolpyruvate carboxykinase 2 (mitochondrial) [Source:MGI Symbol;Acc:MGI:1860456] | yes|up |
| ENSMUSG00000115410 | 2810457G06Rik | RIKEN cDNA 2810457G06 gene [Source:MGI Symbol;Acc:MGI:1920074] | yes|up |
| ENSMUSG00000040612 | Ildr2 | immunoglobulin-like domain containing receptor 2 [Source:MGI Symbol;Acc:MGI:1196370] | yes|up |
| ENSMUSG00000082229 | Nap1l2 | nucleosome assembly protein 1-like 2 [Source:MGI Symbol;Acc:MGI:106654] | yes|down |
| ENSMUSG00000026177 | Slc11a1 | solute carrier family 11 (proton-coupled divalent metal ion transporters), member 1 [Source:MGI Symbol;Acc:MGI:1345275] | yes|up |
| ENSMUSG00000026170 | Cyp27a1 | cytochrome P450, family 27, subfamily a, polypeptide 1 [Source:MGI Symbol;Acc:MGI:88594] | yes|up |
| ENSMUSG00000015053 | Gata2 | GATA binding protein 2 [Source:MGI Symbol;Acc:MGI:95662] | yes|down |
| ENSMUSG00000051065 | Mb21d2 | Mab-21 domain containing 2 [Source:MGI Symbol;Acc:MGI:1917028] | yes|down |
| ENSMUSG00000021340 | Gpld1 | glycosylphosphatidylinositol specific phospholipase D1 [Source:MGI Symbol;Acc:MGI:106604] | yes|up |
| ENSMUSG00000057880 | Abat | 4-aminobutyrate aminotransferase [Source:MGI Symbol;Acc:MGI:2443582] | yes|up |
| ENSMUSG00000105651 | 1700017M07Rik | RIKEN cDNA 1700017M07 gene [Source:MGI Symbol;Acc:MGI:1923648] | yes|up |
| ENSMUSG00000038745 | Nlrp6 | NLR family, pyrin domain containing 6 [Source:MGI Symbol;Acc:MGI:2141990] | yes|up |
| ENSMUSG00000005958 | Ephb3 | Eph receptor B3 [Source:MGI Symbol;Acc:MGI:104770] | yes|up |
| ENSMUSG00000005950 | P2rx5 | purinergic receptor P2X, ligand-gated ion channel, 5 [Source:MGI Symbol;Acc:MGI:2137026] | yes|up |
| ENSMUSG00000029119 | Man2b2 | mannosidase 2, alpha B2 [Source:MGI Symbol;Acc:MGI:1195262] | yes|up |
| ENSMUSG00000081752 | Sms-ps | spermine synthase, pseudogene [Source:MGI Symbol;Acc:MGI:3705601] | yes|down |
| ENSMUSG00000026825 | Dnm1 | dynamin 1 [Source:MGI Symbol;Acc:MGI:107384] | yes|down |
| ENSMUSG00000026826 | Nr4a2 | nuclear receptor subfamily 4, group A, member 2 [Source:MGI Symbol;Acc:MGI:1352456] | yes|down |
| ENSMUSG00000026822 | Lcn2 | lipocalin 2 [Source:MGI Symbol;Acc:MGI:96757] | yes|up |
| ENSMUSG00000093726 | Gm20667 | predicted gene 20667 [Source:MGI Symbol;Acc:MGI:5313114] | yes|down |
| ENSMUSG00000020431 | Adcy1 | adenylate cyclase 1 [Source:MGI Symbol;Acc:MGI:99677] | yes|up |
| ENSMUSG00000020432 | Tcn2 | transcobalamin 2 [Source:MGI Symbol;Acc:MGI:98534] | yes|up |
| ENSMUSG00000085995 | Gm2788 | predicted gene 2788 [Source:MGI Symbol;Acc:MGI:3780956] | yes|down |
| ENSMUSG00000028641 | P3h1 | prolyl 3-hydroxylase 1 [Source:MGI Symbol;Acc:MGI:1888921] | yes|up |
| ENSMUSG00000016239 | Lonrf3 | LON peptidase N-terminal domain and ring finger 3 [Source:MGI Symbol;Acc:MGI:1921615] | yes|up |
| ENSMUSG00000095547 | Gm10719 | predicted gene 10719 [Source:MGI Symbol;Acc:MGI:3641690] | yes|up |
| ENSMUSG00000057037 | Cfhr1 | complement factor H-related 1 [Source:MGI Symbol;Acc:MGI:2138169] | yes|up |
| ENSMUSG00000028399 | Ptprd | protein tyrosine phosphatase, receptor type, D [Source:MGI Symbol;Acc:MGI:97812] | yes|up |
| ENSMUSG00000025140 | Pycr1 | pyrroline-5-carboxylate reductase 1 [Source:MGI Symbol;Acc:MGI:2384795] | yes|up |
| ENSMUSG00000043441 | Gpr149 | G protein-coupled receptor 149 [Source:MGI Symbol;Acc:MGI:2443628] | yes|down |
| ENSMUSG00000052605 | Isoc2b | isochorismatase domain containing 2b [Source:MGI Symbol;Acc:MGI:1914691] | yes|up |
| ENSMUSG00000006360 | Crip1 | cysteine-rich protein 1 (intestinal) [Source:MGI Symbol;Acc:MGI:88501] | yes|down |
| ENSMUSG00000001403 | Ube2c | ubiquitin-conjugating enzyme E2C [Source:MGI Symbol;Acc:MGI:1915862] | yes|down |
| ENSMUSG00000042312 | S100a13 | S100 calcium binding protein A13 [Source:MGI Symbol;Acc:MGI:109581] | yes|down |
| ENSMUSG00000032076 | Cadm1 | cell adhesion molecule 1 [Source:MGI Symbol;Acc:MGI:1889272] | yes|down |
| ENSMUSG00000062737 | Prl3d2 | prolactin family 3, subfamily d, member 1 [Source:MGI Symbol;Acc:MGI:2660935] | yes|down |
| ENSMUSG00000032079 | Apoa5 | apolipoprotein A-V [Source:MGI Symbol;Acc:MGI:1913363] | yes|up |
| ENSMUSG00000120104 |  | novel transcript | yes|down |
| ENSMUSG00000053801 | Grwd1 | glutamate-rich WD repeat containing 1 [Source:MGI Symbol;Acc:MGI:2141989] | yes|down |
| ENSMUSG00000004018 | Fancl | Fanconi anemia, complementation group L [Source:MGI Symbol;Acc:MGI:1914280] | yes|down |
| ENSMUSG00000021913 | Ogdhl | oxoglutarate dehydrogenase-like [Source:MGI Symbol;Acc:MGI:3616088] | yes|down |
| ENSMUSG00000032291 | Crabp1 | cellular retinoic acid binding protein I [Source:MGI Symbol;Acc:MGI:88490] | yes|down |
| ENSMUSG00000081996 | Gm7153 | predicted gene 7153 [Source:MGI Symbol;Acc:MGI:3644674] | yes|up |
| ENSMUSG00000044258 | Ctla2a | cytotoxic T lymphocyte-associated protein 2 alpha [Source:MGI Symbol;Acc:MGI:88554] | yes|up |
| ENSMUSG00000044254 | Pcsk9 | proprotein convertase subtilisin/kexin type 9 [Source:MGI Symbol;Acc:MGI:2140260] | yes|up |
| ENSMUSG00000033214 | Slitrk5 | SLIT and NTRK-like family, member 5 [Source:MGI Symbol;Acc:MGI:2679448] | yes|up |
| ENSMUSG00000033213 | AA467197 | expressed sequence AA467197 [Source:MGI Symbol;Acc:MGI:3034182] | yes|down |
| ENSMUSG00000042992 | Borcs5 | BLOC-1 related complex subunit 5 [Source:MGI Symbol;Acc:MGI:1915024] | yes|down |
| ENSMUSG00000023391 | Dlx2 | distal-less homeobox 2 [Source:MGI Symbol;Acc:MGI:94902] | yes|down |
| ENSMUSG00000000751 | Rpa1 | replication protein A1 [Source:MGI Symbol;Acc:MGI:1915525] | yes|down |
| ENSMUSG00000038539 | Atf5 | activating transcription factor 5 [Source:MGI Symbol;Acc:MGI:2141857] | yes|up |
| ENSMUSG00000032925 | Itgbl1 | integrin, beta-like 1 [Source:MGI Symbol;Acc:MGI:2443439] | yes|up |
| ENSMUSG00000038530 | Rgs4 | regulator of G-protein signaling 4 [Source:MGI Symbol;Acc:MGI:108409] | yes|up |
| ENSMUSG00000042997 | Nhlrc3 | NHL repeat containing 3 [Source:MGI Symbol;Acc:MGI:2444520] | yes|up |
| ENSMUSG00000020392 | Cdkn2aipnl | CDKN2A interacting protein N-terminal like [Source:MGI Symbol;Acc:MGI:1261797] | yes|down |
| ENSMUSG00000020399 | Havcr2 | hepatitis A virus cellular receptor 2 [Source:MGI Symbol;Acc:MGI:2159682] | yes|down |
| ENSMUSG00000041757 | Plekha6 | pleckstrin homology domain containing, family A member 6 [Source:MGI Symbol;Acc:MGI:2388662] | yes|down |
| ENSMUSG00000066842 | Hmcn1 | hemicentin 1 [Source:MGI Symbol;Acc:MGI:2685047] | yes|up |
| ENSMUSG00000047757 | Fancb | Fanconi anemia, complementation group B [Source:MGI Symbol;Acc:MGI:2448558] | yes|down |
| ENSMUSG00000030562 | Nox4 | NADPH oxidase 4 [Source:MGI Symbol;Acc:MGI:1354184] | yes|up |
| ENSMUSG00000030560 | Ctsc | cathepsin C [Source:MGI Symbol;Acc:MGI:109553] | yes|up |
| ENSMUSG00000056978 | Hamp2 | hepcidin antimicrobial peptide 2 [Source:MGI Symbol;Acc:MGI:2153530] | yes|up |
| ENSMUSG00000056973 | Ces1d | carboxylesterase 1D [Source:MGI Symbol;Acc:MGI:2148202] | yes|up |
| ENSMUSG00000051652 | Lrrc3 | leucine rich repeat containing 3 [Source:MGI Symbol;Acc:MGI:2447899] | yes|up |
| ENSMUSG00000056602 | Fry | FRY microtubule binding protein [Source:MGI Symbol;Acc:MGI:2443895] | yes|up |
| ENSMUSG00000090031 | 4732440D04Rik | RIKEN cDNA 4732440D04 gene [Source:MGI Symbol;Acc:MGI:3604103] | yes|up |
| ENSMUSG00000003585 | Sec14l2 | SEC14-like lipid binding 2 [Source:MGI Symbol;Acc:MGI:1915065] | yes|up |
| ENSMUSG00000040152 | Thbs1 | thrombospondin 1 [Source:MGI Symbol;Acc:MGI:98737] | yes|up |
| ENSMUSG00000104713 | Gbp6 | guanylate binding protein 6 [Source:MGI Symbol;Acc:MGI:2140937] | yes|up |
| ENSMUSG00000039376 | Synpo2l | synaptopodin 2-like [Source:MGI Symbol;Acc:MGI:1916010] | yes|up |
| ENSMUSG00000082414 | Gm13303 | predicted gene 13303 [Source:MGI Symbol;Acc:MGI:3705775] | yes|up |
| ENSMUSG00000046805 | Mpeg1 | macrophage expressed gene 1 [Source:MGI Symbol;Acc:MGI:1333743] | yes|up |
| ENSMUSG00000046807 | Lrrc75b | leucine rich repeat containing 75B [Source:MGI Symbol;Acc:MGI:2143657] | yes|down |
| ENSMUSG00000021280 | Exoc3l4 | exocyst complex component 3-like 4 [Source:MGI Symbol;Acc:MGI:1921363] | yes|down |
| ENSMUSG00000074482 | Gm16589 | predicted gene 16589 [Source:MGI Symbol;Acc:MGI:4418561] | yes|down |
| ENSMUSG00000021135 | Slc10a1 | solute carrier family 10 (sodium/bile acid cotransporter family), member 1 [Source:MGI Symbol;Acc:MGI:97379] | yes|up |
| ENSMUSG00000073102 | Drc1 | dynein regulatory complex subunit 1 [Source:MGI Symbol;Acc:MGI:2685906] | yes|up |
| ENSMUSG00000021130 | Galnt16 | polypeptide N-acetylgalactosaminyltransferase 16 [Source:MGI Symbol;Acc:MGI:1917754] | yes|up |
| ENSMUSG00000074738 | Fndc10 | fibronectin type III domain containing 10 [Source:MGI Symbol;Acc:MGI:2444790] | yes|down |
| ENSMUSG00000089774 | Slc5a3 | solute carrier family 5 (inositol transporters), member 3 [Source:MGI Symbol;Acc:MGI:1858226] | yes|down |
| ENSMUSG00000003053 | Cyp2c29 | cytochrome P450, family 2, subfamily c, polypeptide 29 [Source:MGI Symbol;Acc:MGI:103238] | yes|up |
| ENSMUSG00000003477 | Inmt | indolethylamine N-methyltransferase [Source:MGI Symbol;Acc:MGI:102963] | yes|up |
| ENSMUSG00000038295 | Atg9b | autophagy related 9B [Source:MGI Symbol;Acc:MGI:2685420] | yes|down |
| ENSMUSG00000035916 | Ptprq | protein tyrosine phosphatase, receptor type, Q [Source:MGI Symbol;Acc:MGI:1096349] | yes|up |
| ENSMUSG00000085875 | Gm12905 | predicted gene 12905 [Source:MGI Symbol;Acc:MGI:3702581] | yes|up |
| ENSMUSG00000028073 | Pear1 | platelet endothelial aggregation receptor 1 [Source:MGI Symbol;Acc:MGI:1920432] | yes|down |
| ENSMUSG00000035000 | Dpp4 | dipeptidylpeptidase 4 [Source:MGI Symbol;Acc:MGI:94919] | yes|up |
| ENSMUSG00000020661 | Dnmt3a | DNA methyltransferase 3A [Source:MGI Symbol;Acc:MGI:1261827] | yes|up |
| ENSMUSG00000045868 | Gvin1 | GTPase, very large interferon inducible 1 [Source:MGI Symbol;Acc:MGI:1921808] | yes|down |
| ENSMUSG00000044408 | Sptssa | serine palmitoyltransferase, small subunit A [Source:MGI Symbol;Acc:MGI:1913399] | yes|down |
| ENSMUSG00000019866 | Crybg1 | crystallin beta-gamma domain containing 1 [Source:MGI Symbol;Acc:MGI:109544] | yes|down |
| ENSMUSG00000004872 | Pax3 | paired box 3 [Source:MGI Symbol;Acc:MGI:97487] | yes|down |
| ENSMUSG00000075528 | Aarsd1 | alanyl-tRNA synthetase domain containing 1 [Source:MGI Symbol;Acc:MGI:1916934] | yes|down |
| ENSMUSG00000027574 | Nkain4 | Na+/K+ transporting ATPase interacting 4 [Source:MGI Symbol;Acc:MGI:1915372] | yes|up |
| ENSMUSG00000020256 | Aldh1l2 | aldehyde dehydrogenase 1 family, member L2 [Source:MGI Symbol;Acc:MGI:2444680] | yes|up |
| ENSMUSG00000020250 | Txnrd1 | thioredoxin reductase 1 [Source:MGI Symbol;Acc:MGI:1354175] | yes|down |
| ENSMUSG00000045319 | Proser2 | proline and serine rich 2 [Source:MGI Symbol;Acc:MGI:2442238] | yes|down |
| ENSMUSG00000003549 | Ercc1 | excision repair cross-complementing rodent repair deficiency, complementation group 1 [Source:MGI Symbol;Acc:MGI:95412] | yes|down |
| ENSMUSG00000058729 | Lin9 | lin-9 DREAM MuvB core complex component [Source:MGI Symbol;Acc:MGI:1919818] | yes|down |
| ENSMUSG00000001348 | Acp5 | acid phosphatase 5, tartrate resistant [Source:MGI Symbol;Acc:MGI:87883] | yes|up |
| ENSMUSG00000001349 | Cnn1 | calponin 1 [Source:MGI Symbol;Acc:MGI:104979] | yes|up |
| ENSMUSG00000099876 | Gm29650 | predicted gene 29650 [Source:MGI Symbol;Acc:MGI:5580356] | yes|down |
| ENSMUSG00000036437 | Npy1r | neuropeptide Y receptor Y1 [Source:MGI Symbol;Acc:MGI:104963] | yes|up |
| ENSMUSG00000024376 | Epb41l4a | erythrocyte membrane protein band 4.1 like 4a [Source:MGI Symbol;Acc:MGI:103007] | yes|down |
| ENSMUSG00000024378 | Stard4 | StAR-related lipid transfer (START) domain containing 4 [Source:MGI Symbol;Acc:MGI:2156764] | yes|up |
| ENSMUSG00000062683 | Atp5g2 | ATP synthase, H+ transporting, mitochondrial F0 complex, subunit C2 (subunit 9) [Source:MGI Symbol;Acc:MGI:1915192] | yes|down |
| ENSMUSG00000015488 | Cacfd1 | calcium channel flower domain containing 1 [Source:MGI Symbol;Acc:MGI:1924317] | yes|up |
| ENSMUSG00000040093 | Bmf | BCL2 modifying factor [Source:MGI Symbol;Acc:MGI:2176433] | yes|up |
| ENSMUSG00000000560 | Gabra2 | gamma-aminobutyric acid (GABA) A receptor, subunit alpha 2 [Source:MGI Symbol;Acc:MGI:95614] | yes|up |
| ENSMUSG00000072082 | Ccnf | cyclin F [Source:MGI Symbol;Acc:MGI:102551] | yes|down |
| ENSMUSG00000026158 | Ogfrl1 | opioid growth factor receptor-like 1 [Source:MGI Symbol;Acc:MGI:1917405] | yes|down |
| ENSMUSG00000026622 | Nek2 | NIMA (never in mitosis gene a)-related expressed kinase 2 [Source:MGI Symbol;Acc:MGI:109359] | yes|down |
| ENSMUSG00000097042 | Gm17491 | predicted gene, 17491 [Source:MGI Symbol;Acc:MGI:4937125] | yes|down |
| ENSMUSG00000055301 | Adh7 | alcohol dehydrogenase 7 (class IV), mu or sigma polypeptide [Source:MGI Symbol;Acc:MGI:87926] | yes|down |
| ENSMUSG00000031379 | Pir | pirin [Source:MGI Symbol;Acc:MGI:1916906] | yes|down |
| ENSMUSG00000031803 | B3gnt3 | UDP-GlcNAc:betaGal beta-1,3-N-acetylglucosaminyltransferase 3 [Source:MGI Symbol;Acc:MGI:2152535] | yes|down |
| ENSMUSG00000057948 | Unc13d | unc-13 homolog D [Source:MGI Symbol;Acc:MGI:1917700] | yes|down |
| ENSMUSG00000110631 | Gm42047 | predicted gene, 42047 [Source:MGI Symbol;Acc:MGI:5624932] | yes|down |
| ENSMUSG00000042804 | Gpr153 | G protein-coupled receptor 153 [Source:MGI Symbol;Acc:MGI:1916157] | yes|up |
| ENSMUSG00000094786 | Gm14403 | predicted gene 14403 [Source:MGI Symbol;Acc:MGI:3649813] | yes|up |
| ENSMUSG00000022706 | Mrpl40 | mitochondrial ribosomal protein L40 [Source:MGI Symbol;Acc:MGI:1332635] | yes|down |
| ENSMUSG00000026873 | Phf19 | PHD finger protein 19 [Source:MGI Symbol;Acc:MGI:1921266] | yes|down |
| ENSMUSG00000026841 | Fibcd1 | fibrinogen C domain containing 1 [Source:MGI Symbol;Acc:MGI:2138953] | yes|down |
| ENSMUSG00000020415 | Pttg1 | pituitary tumor-transforming gene 1 [Source:MGI Symbol;Acc:MGI:1353578] | yes|down |
| ENSMUSG00000046324 | Ermp1 | endoplasmic reticulum metallopeptidase 1 [Source:MGI Symbol;Acc:MGI:106250] | yes|down |
| ENSMUSG00000029394 | Cdk2ap1 | CDK2 (cyclin-dependent kinase 2)-associated protein 1 [Source:MGI Symbol;Acc:MGI:1202069] | yes|down |
| ENSMUSG00000045672 | Col27a1 | collagen, type XXVII, alpha 1 [Source:MGI Symbol;Acc:MGI:2672118] | yes|up |
| ENSMUSG00000006342 | Susd2 | sushi domain containing 2 [Source:MGI Symbol;Acc:MGI:1918983] | yes|up |
| ENSMUSG00000035493 | Tgfbi | transforming growth factor, beta induced [Source:MGI Symbol;Acc:MGI:99959] | yes|up |
| ENSMUSG00000042379 | Esm1 | endothelial cell-specific molecule 1 [Source:MGI Symbol;Acc:MGI:1918940] | yes|down |
| ENSMUSG00000045410 | Akr1e1 | aldo-keto reductase family 1, member E1 [Source:MGI Symbol;Acc:MGI:1914758] | yes|down |
| ENSMUSG00000025161 | Slc16a3 | solute carrier family 16 (monocarboxylic acid transporters), member 3 [Source:MGI Symbol;Acc:MGI:1933438] | yes|up |
| ENSMUSG00000028445 | Enho | energy homeostasis associated [Source:MGI Symbol;Acc:MGI:1916888] | yes|up |
| ENSMUSG00000114638 | Gm31834 | predicted gene, 31834 [Source:MGI Symbol;Acc:MGI:5590993] | yes|down |
| ENSMUSG00000032726 | Bmp8a | bone morphogenetic protein 8a [Source:MGI Symbol;Acc:MGI:104515] | yes|up |
| ENSMUSG00000067889 | Sptbn2 | spectrin beta, non-erythrocytic 2 [Source:MGI Symbol;Acc:MGI:1313261] | yes|up |
| ENSMUSG00000019303 | Psmc3ip | proteasome (prosome, macropain) 26S subunit, ATPase 3, interacting protein [Source:MGI Symbol;Acc:MGI:1098610] | yes|down |
| ENSMUSG00000087381 | Gm16008 | predicted gene 16008 [Source:MGI Symbol;Acc:MGI:3802048] | yes|up |
| ENSMUSG00000033965 | Slc16a2 | solute carrier family 16 (monocarboxylic acid transporters), member 2 [Source:MGI Symbol;Acc:MGI:1203732] | yes|up |
| ENSMUSG00000024413 | Npc1 | NPC intracellular cholesterol transporter 1 [Source:MGI Symbol;Acc:MGI:1097712] | yes|up |
| ENSMUSG00000042985 | Upk3b | uroplakin 3B [Source:MGI Symbol;Acc:MGI:2140882] | yes|up |
| ENSMUSG00000050370 | Ch25h | cholesterol 25-hydroxylase [Source:MGI Symbol;Acc:MGI:1333869] | yes|up |
| ENSMUSG00000059187 | Tafa1 | TAFA chemokine like family member 1 [Source:MGI Symbol;Acc:MGI:2443695] | yes|down |
| ENSMUSG00000001281 | Itgb7 | integrin beta 7 [Source:MGI Symbol;Acc:MGI:96616] | yes|down |
| ENSMUSG00000002885 | Adgre5 | adhesion G protein-coupled receptor E5 [Source:MGI Symbol;Acc:MGI:1347095] | yes|up |
| ENSMUSG00000036602 | Alx1 | ALX homeobox 1 [Source:MGI Symbol;Acc:MGI:104621] | yes|down |
| ENSMUSG00000032946 | Rasgrp2 | RAS, guanyl releasing protein 2 [Source:MGI Symbol;Acc:MGI:1333849] | yes|up |
| ENSMUSG00000086477 | Gm15506 | predicted gene 15506 [Source:MGI Symbol;Acc:MGI:3782954] | yes|up |
| ENSMUSG00000067235 | H2-Q10 | histocompatibility 2, Q region locus 10 [Source:MGI Symbol;Acc:MGI:95929] | yes|up |
| ENSMUSG00000030761 | Myo7a | myosin VIIA [Source:MGI Symbol;Acc:MGI:104510] | yes|up |
| ENSMUSG00000030762 | Aqp8 | aquaporin 8 [Source:MGI Symbol;Acc:MGI:1195271] | yes|up |
| ENSMUSG00000121185 |  | novel transcript, antisense to Slc38a1 | yes|up |
| ENSMUSG00000121184 |  | novel transcript, sense intronic to Atp6v0a1 | yes|up |
| ENSMUSG00000038070 | Cntln | centlein, centrosomal protein [Source:MGI Symbol;Acc:MGI:2443104] | yes|down |
| ENSMUSG00000072476 | Gm9008 | predicted pseudogene 9008 [Source:MGI Symbol;Acc:MGI:3644000] | yes|up |
| ENSMUSG00000024140 | Epas1 | endothelial PAS domain protein 1 [Source:MGI Symbol;Acc:MGI:109169] | yes|up |
| ENSMUSG00000054889 | Dsp | desmoplakin [Source:MGI Symbol;Acc:MGI:109611] | yes|up |
| ENSMUSG00000072692 | Rpl37rt | ribosomal protein L37, retrotransposed [Source:MGI Symbol;Acc:MGI:3651519] | yes|down |
| ENSMUSG00000037266 | Rsrp1 | arginine/serine rich protein 1 [Source:MGI Symbol;Acc:MGI:106498] | yes|up |
| ENSMUSG00000040134 | Rdh7 | retinol dehydrogenase 7 [Source:MGI Symbol;Acc:MGI:1860517] | yes|up |
| ENSMUSG00000056486 | Chn1 | chimerin 1 [Source:MGI Symbol;Acc:MGI:1915674] | yes|up |
| ENSMUSG00000046865 | Fbl | fibrillarin [Source:MGI Symbol;Acc:MGI:95486] | yes|down |
| ENSMUSG00000039396 | Neil3 | nei like 3 (E. coli) [Source:MGI Symbol;Acc:MGI:2384588] | yes|down |
| ENSMUSG00000029095 | Ablim2 | actin-binding LIM protein 2 [Source:MGI Symbol;Acc:MGI:2385758] | yes|up |
| ENSMUSG00000029096 | Htra3 | HtrA serine peptidase 3 [Source:MGI Symbol;Acc:MGI:1925808] | yes|up |
| ENSMUSG00000057534 | Elobl | elongin B-like [Source:MGI Symbol;Acc:MGI:1860403] | yes|up |
| ENSMUSG00000071414 | Gm6736 | predicted gene 6736 [Source:MGI Symbol;Acc:MGI:3643048] | yes|down |
| ENSMUSG00000025420 | Katnal2 | katanin p60 subunit A-like 2 [Source:MGI Symbol;Acc:MGI:1924234] | yes|down |
| ENSMUSG00000034040 | Galnt17 | polypeptide N-acetylgalactosaminyltransferase 17 [Source:MGI Symbol;Acc:MGI:2137594] | yes|up |
| ENSMUSG00000025429 | Pstpip2 | proline-serine-threonine phosphatase-interacting protein 2 [Source:MGI Symbol;Acc:MGI:1335088] | yes|down |
| ENSMUSG00000061666 | Gdpd1 | glycerophosphodiester phosphodiesterase domain containing 1 [Source:MGI Symbol;Acc:MGI:1913819] | yes|up |
| ENSMUSG00000056222 | Spock1 | sparc/osteonectin, cwcv and kazal-like domains proteoglycan 1 [Source:MGI Symbol;Acc:MGI:105371] | yes|up |
| ENSMUSG00000029270 | Dipk1a | divergent protein kinase domain 1A [Source:MGI Symbol;Acc:MGI:1914516] | yes|down |
| ENSMUSG00000057286 | St6galnac2 | ST6 (alpha-N-acetyl-neuraminyl-2,3-beta-galactosyl-1,3)-N-acetylgalactosaminide alpha-2,6-sialyltransferase 2 [Source:MGI Symbol;Acc:MGI:107553] | yes|down |
| ENSMUSG00000025395 | Prim1 | DNA primase, p49 subunit [Source:MGI Symbol;Acc:MGI:97757] | yes|down |
| ENSMUSG00000038400 | Pmepa1 | prostate transmembrane protein, androgen induced 1 [Source:MGI Symbol;Acc:MGI:1929600] | yes|up |
| ENSMUSG00000039033 | Tasp1 | taspase, threonine aspartase 1 [Source:MGI Symbol;Acc:MGI:1923062] | yes|down |
| ENSMUSG00000085811 | Cep112it | centrosomal protein 112, intronic transcript [Source:MGI Symbol;Acc:MGI:3649867] | yes|up |
| ENSMUSG00000002384 | Bmp8b | bone morphogenetic protein 8b [Source:MGI Symbol;Acc:MGI:107335] | yes|down |
| ENSMUSG00000046688 | Tifa | TRAF-interacting protein with forkhead-associated domain [Source:MGI Symbol;Acc:MGI:2182965] | yes|up |
| ENSMUSG00000095193 | Gm20939 | predicted gene, 20939 [Source:MGI Symbol;Acc:MGI:5434295] | yes|up |
| ENSMUSG00000020609 | Apob | apolipoprotein B [Source:MGI Symbol;Acc:MGI:88052] | yes|up |
| ENSMUSG00000045083 | Lingo2 | leucine rich repeat and Ig domain containing 2 [Source:MGI Symbol;Acc:MGI:2442298] | yes|down |
| ENSMUSG00000069922 | Ces3a | carboxylesterase 3A [Source:MGI Symbol;Acc:MGI:102773] | yes|up |
| ENSMUSG00000064215 | Ifi27 | interferon, alpha-inducible protein 27 [Source:MGI Symbol;Acc:MGI:1277180] | yes|up |
| ENSMUSG00000019846 | Lama4 | laminin, alpha 4 [Source:MGI Symbol;Acc:MGI:109321] | yes|up |
| ENSMUSG00000010797 | Wnt2 | wingless-type MMTV integration site family, member 2 [Source:MGI Symbol;Acc:MGI:98954] | yes|down |
| ENSMUSG00000027559 | Car3 | carbonic anhydrase 3 [Source:MGI Symbol;Acc:MGI:88270] | yes|up |
| ENSMUSG00000027555 | Car13 | carbonic anhydrase 13 [Source:MGI Symbol;Acc:MGI:1931322] | yes|down |
| ENSMUSG00000070594 | Cfhr4 | complement factor H-related 4 [Source:MGI Symbol;Acc:MGI:3646434] | yes|up |
| ENSMUSG00000021301 | Hecw1 | HECT, C2 and WW domain containing E3 ubiquitin protein ligase 1 [Source:MGI Symbol;Acc:MGI:2444115] | yes|up |
| ENSMUSG00000002944 | Cd36 | CD36 molecule [Source:MGI Symbol;Acc:MGI:107899] | yes|up |
| ENSMUSG00000017446 | C1qtnf1 | C1q and tumor necrosis factor related protein 1 [Source:MGI Symbol;Acc:MGI:1919254] | yes|up |
| ENSMUSG00000090862 | Rps13 | ribosomal protein S13 [Source:MGI Symbol;Acc:MGI:1915302] | yes|down |
| ENSMUSG00000037725 | Ckap2 | cytoskeleton associated protein 2 [Source:MGI Symbol;Acc:MGI:1931797] | yes|down |
| ENSMUSG00000037894 | H2az1 | H2A.Z variant histone 1 [Source:MGI Symbol;Acc:MGI:1888388] | yes|down |
| ENSMUSG00000054162 | Spock3 | sparc/osteonectin, cwcv and kazal-like domains proteoglycan 3 [Source:MGI Symbol;Acc:MGI:1920152] | yes|up |
| ENSMUSG00000068890 | Lce1a2 | late cornified envelope 1A2 [Source:MGI Symbol;Acc:MGI:1920972] | yes|up |
| ENSMUSG00000033170 | Card10 | caspase recruitment domain family, member 10 [Source:MGI Symbol;Acc:MGI:2146012] | yes|up |
| ENSMUSG00000036411 | 9530077C05Rik | RIKEN cDNA 9530077C05 gene [Source:MGI Symbol;Acc:MGI:1915533] | yes|down |
| ENSMUSG00000036412 | Arsi | arylsulfatase i [Source:MGI Symbol;Acc:MGI:2670959] | yes|up |
| ENSMUSG00000112096 | A430103D13Rik | RIKEN cDNA A430103D13 gene [Source:MGI Symbol;Acc:MGI:1925025] | yes|down |
| ENSMUSG00000024600 | Slc27a6 | solute carrier family 27 (fatty acid transporter), member 6 [Source:MGI Symbol;Acc:MGI:3036230] | yes|up |
| ENSMUSG00000071178 | Serpina1b | serine (or cysteine) preptidase inhibitor, clade A, member 1B [Source:MGI Symbol;Acc:MGI:891970] | yes|up |
| ENSMUSG00000113149 | Gm49383 | predicted gene, 49383 [Source:MGI Symbol;Acc:MGI:6121605] | yes|down |
| ENSMUSG00000071177 | Serpina1d | serine (or cysteine) peptidase inhibitor, clade A, member 1D [Source:MGI Symbol;Acc:MGI:891968] | yes|up |
| ENSMUSG00000027800 | Tm4sf1 | transmembrane 4 superfamily member 1 [Source:MGI Symbol;Acc:MGI:104678] | yes|down |
| ENSMUSG00000061878 | Sphk1 | sphingosine kinase 1 [Source:MGI Symbol;Acc:MGI:1316649] | yes|up |
| ENSMUSG00000091736 | Yy2 | Yy2 transcription factor [Source:MGI Symbol;Acc:MGI:3837947] | yes|down |
| ENSMUSG00000030110 | Ret | ret proto-oncogene [Source:MGI Symbol;Acc:MGI:97902] | yes|up |
| ENSMUSG00000055561 | Spink5 | serine peptidase inhibitor, Kazal type 5 [Source:MGI Symbol;Acc:MGI:1919682] | yes|up |
| ENSMUSG00000093594 | Gm20707 | predicted gene 20707 [Source:MGI Symbol;Acc:MGI:5313154] | yes|down |
| ENSMUSG00000026605 | Cenpf | centromere protein F [Source:MGI Symbol;Acc:MGI:1313302] | yes|down |
| ENSMUSG00000097061 | 9330151L19Rik | RIKEN cDNA 9330151L19 gene [Source:MGI Symbol;Acc:MGI:3041168] | yes|up |
| ENSMUSG00000046733 | Gprc5a | G protein-coupled receptor, family C, group 5, member A [Source:MGI Symbol;Acc:MGI:1891250] | yes|down |
| ENSMUSG00000056035 | Cyp3a11 | cytochrome P450, family 3, subfamily a, polypeptide 11 [Source:MGI Symbol;Acc:MGI:88609] | yes|up |
| ENSMUSG00000051022 | Hs3st1 | heparan sulfate (glucosamine) 3-O-sulfotransferase 1 [Source:MGI Symbol;Acc:MGI:1201606] | yes|down |
| ENSMUSG00000056031 | 9330154J02Rik | RIKEN cDNA 9330154J02 gene [Source:MGI Symbol;Acc:MGI:3045244] | yes|down |
| ENSMUSG00000119954 | Gm35546 | predicted gene, 35546 [Source:NCBI gene (formerly Entrezgene);Acc:102639175] | yes|up |
| ENSMUSG00000031824 | 6430548M08Rik | RIKEN cDNA 6430548M08 gene [Source:MGI Symbol;Acc:MGI:2443793] | yes|up |
| ENSMUSG00000031825 | Crispld2 | cysteine-rich secretory protein LCCL domain containing 2 [Source:MGI Symbol;Acc:MGI:1926142] | yes|up |
| ENSMUSG00000031827 | Cotl1 | coactosin-like 1 (Dictyostelium) [Source:MGI Symbol;Acc:MGI:1919292] | yes|down |
| ENSMUSG00000031821 | Gins2 | GINS complex subunit 2 (Psf2 homolog) [Source:MGI Symbol;Acc:MGI:1921019] | yes|down |
| ENSMUSG00000031822 | Gse1 | genetic suppressor element 1, coiled-coil protein [Source:MGI Symbol;Acc:MGI:1098275] | yes|down |
| ENSMUSG00000022324 | Matn2 | matrilin 2 [Source:MGI Symbol;Acc:MGI:109613] | yes|down |
| ENSMUSG00000041592 | Sdk2 | sidekick cell adhesion molecule 2 [Source:MGI Symbol;Acc:MGI:2443847] | yes|up |
| ENSMUSG00000014496 | Ankrd28 | ankyrin repeat domain 28 [Source:MGI Symbol;Acc:MGI:2145661] | yes|down |
| ENSMUSG00000063297 | Luzp2 | leucine zipper protein 2 [Source:MGI Symbol;Acc:MGI:1889615] | yes|down |
| ENSMUSG00000006715 | Gmnn | geminin [Source:MGI Symbol;Acc:MGI:1927344] | yes|down |
| ENSMUSG00000038379 | Ttk | Ttk protein kinase [Source:MGI Symbol;Acc:MGI:1194921] | yes|down |
| ENSMUSG00000035258 | Abi3bp | ABI family member 3 binding protein [Source:MGI Symbol;Acc:MGI:2444583] | yes|up |
| ENSMUSG00000120850 |  | novel transcript, sense intronic to Enox1 | yes|down |
| ENSMUSG00000038375 | Trp53inp2 | transformation related protein 53 inducible nuclear protein 2 [Source:MGI Symbol;Acc:MGI:1915978] | yes|up |
| ENSMUSG00000120856 |  | novel transcript, antisense to Celsr3and Slc26a6 | yes|up |
| ENSMUSG00000038370 | Pcp4l1 | Purkinje cell protein 4-like 1 [Source:MGI Symbol;Acc:MGI:1913675] | yes|up |
| ENSMUSG00000020474 | Polm | polymerase (DNA directed), mu [Source:MGI Symbol;Acc:MGI:1860191] | yes|down |
| ENSMUSG00000026866 | Kynu | kynureninase [Source:MGI Symbol;Acc:MGI:1918039] | yes|up |
| ENSMUSG00000001021 | S100a3 | S100 calcium binding protein A3 [Source:MGI Symbol;Acc:MGI:1338849] | yes|down |
| ENSMUSG00000022995 | Enah | ENAH actin regulator [Source:MGI Symbol;Acc:MGI:108360] | yes|up |
| ENSMUSG00000107928 | Gm45140 | predicted gene 45140 [Source:MGI Symbol;Acc:MGI:5753716] | yes|down |
| ENSMUSG00000032397 | Tipin | timeless interacting protein [Source:MGI Symbol;Acc:MGI:1921571] | yes|down |
| ENSMUSG00000047878 | A4galt | alpha 1,4-galactosyltransferase [Source:MGI Symbol;Acc:MGI:3512453] | yes|up |
| ENSMUSG00000057074 | Ces1g | carboxylesterase 1G [Source:MGI Symbol;Acc:MGI:88378] | yes|up |
| ENSMUSG00000045659 | Plekha7 | pleckstrin homology domain containing, family A member 7 [Source:MGI Symbol;Acc:MGI:2445094] | yes|up |
| ENSMUSG00000042686 | Jph1 | junctophilin 1 [Source:MGI Symbol;Acc:MGI:1891495] | yes|up |
| ENSMUSG00000062960 | Kdr | kinase insert domain protein receptor [Source:MGI Symbol;Acc:MGI:96683] | yes|up |
| ENSMUSG00000028600 | Podn | podocan [Source:MGI Symbol;Acc:MGI:2674939] | yes|up |
| ENSMUSG00000042436 | Mfap4 | microfibrillar-associated protein 4 [Source:MGI Symbol;Acc:MGI:1342276] | yes|up |
| ENSMUSG00000034706 | Dnai2 | dynein axonemal intermediate chain 2 [Source:MGI Symbol;Acc:MGI:2685574] | yes|up |
| ENSMUSG00000021070 | Bdkrb2 | bradykinin receptor, beta 2 [Source:MGI Symbol;Acc:MGI:102845] | yes|down |
| ENSMUSG00000052133 | Sema5b | sema domain, seven thrombospondin repeats (type 1 and type 1-like), transmembrane domain (TM) and short cytoplasmic domain, (semaphorin) 5B [Source:MGI Symbol;Acc:MGI:107555] | yes|up |
| ENSMUSG00000023927 | Satb1 | special AT-rich sequence binding protein 1 [Source:MGI Symbol;Acc:MGI:105084] | yes|up |
| ENSMUSG00000028933 | Xrcc2 | X-ray repair complementing defective repair in Chinese hamster cells 2 [Source:MGI Symbol;Acc:MGI:1927345] | yes|down |
| ENSMUSG00000050860 | Phospho1 | phosphatase, orphan 1 [Source:MGI Symbol;Acc:MGI:2447348] | yes|up |
| ENSMUSG00000017146 | Brca1 | breast cancer 1, early onset [Source:MGI Symbol;Acc:MGI:104537] | yes|down |
| ENSMUSG00000074207 | Adh1 | alcohol dehydrogenase 1 (class I) [Source:MGI Symbol;Acc:MGI:87921] | yes|up |
| ENSMUSG00000024164 | C3 | complement component 3 [Source:MGI Symbol;Acc:MGI:88227] | yes|up |
| ENSMUSG00000116657 | Gm49774 | predicted gene, 49774 [Source:MGI Symbol;Acc:MGI:6215288] | yes|down |
| ENSMUSG00000016637 | Ift27 | intraflagellar transport 27 [Source:MGI Symbol;Acc:MGI:1914292] | yes|down |
| ENSMUSG00000120144 |  | novel transcript | yes|down |
| ENSMUSG00000002718 | Cse1l | chromosome segregation 1-like (S. cerevisiae) [Source:MGI Symbol;Acc:MGI:1339951] | yes|down |
| ENSMUSG00000053519 | Kcnip1 | Kv channel-interacting protein 1 [Source:MGI Symbol;Acc:MGI:1917607] | yes|down |
| ENSMUSG00000042359 | Osbpl6 | oxysterol binding protein-like 6 [Source:MGI Symbol;Acc:MGI:2139014] | yes|down |
| ENSMUSG00000018581 | Dnah11 | dynein, axonemal, heavy chain 11 [Source:MGI Symbol;Acc:MGI:1100864] | yes|up |
| ENSMUSG00000083087 | Gm11249 | predicted gene 11249 [Source:MGI Symbol;Acc:MGI:3650834] | yes|up |
| ENSMUSG00000024292 | Cyp4f14 | cytochrome P450, family 4, subfamily f, polypeptide 14 [Source:MGI Symbol;Acc:MGI:1927669] | yes|up |
| ENSMUSG00000024299 | Adamts10 | a disintegrin-like and metallopeptidase (reprolysin type) with thrombospondin type 1 motif, 10 [Source:MGI Symbol;Acc:MGI:2449112] | yes|up |
| ENSMUSG00000044217 | Aqp5 | aquaporin 5 [Source:MGI Symbol;Acc:MGI:106215] | yes|down |
| ENSMUSG00000107317 | Gm19719 | predicted gene, 19719 [Source:MGI Symbol;Acc:MGI:5011904] | yes|up |
| ENSMUSG00000117300 | Gm18736 | predicted gene, 18736 [Source:MGI Symbol;Acc:MGI:5010921] | yes|down |
| ENSMUSG00000021048 | Mthfd1 | methylenetetrahydrofolate dehydrogenase (NADP+ dependent), methenyltetrahydrofolate cyclohydrolase, formyltetrahydrofolate synthase [Source:MGI Symbol;Acc:MGI:1342005] | yes|down |
| ENSMUSG00000101878 | Gm8203 | predicted pseudogene 8203 [Source:MGI Symbol;Acc:MGI:3646499] | yes|down |
| ENSMUSG00000037405 | Icam1 | intercellular adhesion molecule 1 [Source:MGI Symbol;Acc:MGI:96392] | yes|up |
| ENSMUSG00000038578 | Susd1 | sushi domain containing 1 [Source:MGI Symbol;Acc:MGI:3651543] | yes|up |
| ENSMUSG00000063767 | S100a7a | S100 calcium binding protein A7A [Source:MGI Symbol;Acc:MGI:2687194] | yes|down |
| ENSMUSG00000121430 |  | novel transcript | yes|down |
| ENSMUSG00000024833 | Pola2 | polymerase (DNA directed), alpha 2 [Source:MGI Symbol;Acc:MGI:99690] | yes|down |
| ENSMUSG00000030701 | Plekhb1 | pleckstrin homology domain containing, family B (evectins) member 1 [Source:MGI Symbol;Acc:MGI:1351469] | yes|up |
| ENSMUSG00000070780 | Rbm47 | RNA binding motif protein 47 [Source:MGI Symbol;Acc:MGI:2384294] | yes|down |
| ENSMUSG00000048216 | Gpr85 | G protein-coupled receptor 85 [Source:MGI Symbol;Acc:MGI:1927851] | yes|down |
| ENSMUSG00000048138 | Dmrt2 | doublesex and mab-3 related transcription factor 2 [Source:MGI Symbol;Acc:MGI:1330307] | yes|up |
| ENSMUSG00000090077 | Lime1 | Lck interacting transmembrane adaptor 1 [Source:MGI Symbol;Acc:MGI:1919949] | yes|up |
| ENSMUSG00000006529 | Itih1 | inter-alpha trypsin inhibitor, heavy chain 1 [Source:MGI Symbol;Acc:MGI:96618] | yes|up |
| ENSMUSG00000006522 | Itih3 | inter-alpha trypsin inhibitor, heavy chain 3 [Source:MGI Symbol;Acc:MGI:96620] | yes|up |
| ENSMUSG00000030834 | Abcc6 | ATP-binding cassette, sub-family C (CFTR/MRP), member 6 [Source:MGI Symbol;Acc:MGI:1351634] | yes|up |
| ENSMUSG00000092365 | BC023719 | cDNA sequence BC023719 [Source:MGI Symbol;Acc:MGI:3574836] | yes|up |
| ENSMUSG00000102752 | Gm7694 | predicted gene 7694 [Source:MGI Symbol;Acc:MGI:3649135] | yes|up |
| ENSMUSG00000073494 | Sh2d1b2 | SH2 domain containing 1B2 [Source:MGI Symbol;Acc:MGI:3622649] | yes|down |
| ENSMUSG00000031613 | Hpgd | hydroxyprostaglandin dehydrogenase 15 (NAD) [Source:MGI Symbol;Acc:MGI:108085] | yes|up |
| ENSMUSG00000031610 | Scrg1 | scrapie responsive gene 1 [Source:MGI Symbol;Acc:MGI:1328308] | yes|up |
| ENSMUSG00000031616 | Ednra | endothelin receptor type A [Source:MGI Symbol;Acc:MGI:105923] | yes|up |
| ENSMUSG00000003541 | Ier3 | immediate early response 3 [Source:MGI Symbol;Acc:MGI:104814] | yes|down |
| ENSMUSG00000026475 | Rgs16 | regulator of G-protein signaling 16 [Source:MGI Symbol;Acc:MGI:108407] | yes|down |
| ENSMUSG00000003545 | Fosb | FBJ osteosarcoma oncogene B [Source:MGI Symbol;Acc:MGI:95575] | yes|down |
| ENSMUSG00000025408 | Ddit3 | DNA-damage inducible transcript 3 [Source:MGI Symbol;Acc:MGI:109247] | yes|up |
| ENSMUSG00000061436 | Hipk2 | homeodomain interacting protein kinase 2 [Source:MGI Symbol;Acc:MGI:1314872] | yes|up |
| ENSMUSG00000061601 | Pclo | piccolo (presynaptic cytomatrix protein) [Source:MGI Symbol;Acc:MGI:1349390] | yes|up |
| ENSMUSG00000061603 | Akap6 | A kinase (PRKA) anchor protein 6 [Source:MGI Symbol;Acc:MGI:3050566] | yes|up |
| ENSMUSG00000073147 | 5031425E22Rik | RIKEN cDNA 5031425E22 gene [Source:MGI Symbol;Acc:MGI:1923227] | yes|up |
| ENSMUSG00000029217 | Tec | tec protein tyrosine kinase [Source:MGI Symbol;Acc:MGI:98662] | yes|up |
| ENSMUSG00000029212 | Gabrb1 | gamma-aminobutyric acid (GABA) A receptor, subunit beta 1 [Source:MGI Symbol;Acc:MGI:95619] | yes|up |
| ENSMUSG00000062006 | Rpl34 | ribosomal protein L34 [Source:MGI Symbol;Acc:MGI:1915686] | yes|down |
| ENSMUSG00000120442 |  | novel transcript | yes|up |
| ENSMUSG00000020598 | Nrcam | neuronal cell adhesion molecule [Source:MGI Symbol;Acc:MGI:104750] | yes|down |
| ENSMUSG00000020623 | Map2k6 | mitogen-activated protein kinase kinase 6 [Source:MGI Symbol;Acc:MGI:1346870] | yes|down |
| ENSMUSG00000028037 | Ifi44 | interferon-induced protein 44 [Source:MGI Symbol;Acc:MGI:2443016] | yes|up |
| ENSMUSG00000028031 | Dkk2 | dickkopf WNT signaling pathway inhibitor 2 [Source:MGI Symbol;Acc:MGI:1890663] | yes|down |
| ENSMUSG00000034936 | Arl4d | ADP-ribosylation factor-like 4D [Source:MGI Symbol;Acc:MGI:1933155] | yes|down |
| ENSMUSG00000068231 | Vmn1r43 | vomeronasal 1 receptor 43 [Source:MGI Symbol;Acc:MGI:2148510] | yes|down |
| ENSMUSG00000053985 | Zfp14 | zinc finger protein 14 [Source:MGI Symbol;Acc:MGI:99160] | yes|up |
| ENSMUSG00000052364 | B630019K06Rik | RIKEN cDNA B630019K06 gene [Source:MGI Symbol;Acc:MGI:2147918] | yes|down |
| ENSMUSG00000078776 | 9530053A07Rik | RIKEN cDNA 9530053A07 gene [Source:MGI Symbol;Acc:MGI:2442118] | yes|down |
| ENSMUSG00000078773 | Rad54b | RAD54 homolog B (S. cerevisiae) [Source:MGI Symbol;Acc:MGI:3605986] | yes|down |
| ENSMUSG00000078771 | Evi2a | ecotropic viral integration site 2a [Source:MGI Symbol;Acc:MGI:95458] | yes|down |
| ENSMUSG00000033377 | Palmd | palmdelphin [Source:MGI Symbol;Acc:MGI:2148896] | yes|up |
| ENSMUSG00000032578 | Cish | cytokine inducible SH2-containing protein [Source:MGI Symbol;Acc:MGI:103159] | yes|up |
| ENSMUSG00000024338 | Psmb8 | proteasome (prosome, macropain) subunit, beta type 8 (large multifunctional peptidase 7) [Source:MGI Symbol;Acc:MGI:1346527] | yes|up |
| ENSMUSG00000116226 | Gm49502 | predicted gene, 49502 [Source:MGI Symbol;Acc:MGI:6155188] | yes|up |
| ENSMUSG00000019539 | Rcn3 | reticulocalbin 3, EF-hand calcium binding domain [Source:MGI Symbol;Acc:MGI:1277122] | yes|up |
| ENSMUSG00000040329 | Il7 | interleukin 7 [Source:MGI Symbol;Acc:MGI:96561] | yes|down |
| ENSMUSG00000028197 | Col24a1 | collagen, type XXIV, alpha 1 [Source:MGI Symbol;Acc:MGI:1918605] | yes|up |
| ENSMUSG00000032265 | Tent5a | terminal nucleotidyltransferase 5A [Source:MGI Symbol;Acc:MGI:2670964] | yes|up |
| ENSMUSG00000050069 | Grem2 | gremlin 2, DAN family BMP antagonist [Source:MGI Symbol;Acc:MGI:1344367] | yes|down |
| ENSMUSG00000005268 | Prlr | prolactin receptor [Source:MGI Symbol;Acc:MGI:97763] | yes|up |
| ENSMUSG00000024620 | Pdgfrb | platelet derived growth factor receptor, beta polypeptide [Source:MGI Symbol;Acc:MGI:97531] | yes|up |
| ENSMUSG00000024330 | Col11a2 | collagen, type XI, alpha 2 [Source:MGI Symbol;Acc:MGI:88447] | yes|up |
| ENSMUSG00000026664 | Phyh | phytanoyl-CoA hydroxylase [Source:MGI Symbol;Acc:MGI:891978] | yes|up |
| ENSMUSG00000025405 | Inhbc | inhibin beta-C [Source:MGI Symbol;Acc:MGI:105932] | yes|up |
| ENSMUSG00000026669 | Mcm10 | minichromosome maintenance 10 replication initiation factor [Source:MGI Symbol;Acc:MGI:1917274] | yes|down |
| ENSMUSG00000020836 | Coro6 | coronin 6 [Source:MGI Symbol;Acc:MGI:2183448] | yes|up |
| ENSMUSG00000020831 | 0610010K14Rik | RIKEN cDNA 0610010K14 gene [Source:MGI Symbol;Acc:MGI:1915609] | yes|down |
| ENSMUSG00000041180 | Hectd2 | HECT domain E3 ubiquitin protein ligase 2 [Source:MGI Symbol;Acc:MGI:2442663] | yes|up |
| ENSMUSG00000041633 | Kctd12b | potassium channel tetramerisation domain containing 12b [Source:MGI Symbol;Acc:MGI:2444667] | yes|down |
| ENSMUSG00000093577 | Gm20632 | predicted gene 20632 [Source:MGI Symbol;Acc:MGI:5313079] | yes|up |
| ENSMUSG00000042988 | Notum | notum palmitoleoyl-protein carboxylesterase [Source:MGI Symbol;Acc:MGI:1924833] | yes|down |
| ENSMUSG00000097000 | Gm17435 | predicted gene, 17435 [Source:MGI Symbol;Acc:MGI:4937069] | yes|up |
| ENSMUSG00000040584 | Abcb1a | ATP-binding cassette, sub-family B (MDR/TAP), member 1A [Source:MGI Symbol;Acc:MGI:97570] | yes|down |
| ENSMUSG00000121377 | Gm5113 | predicted gene 5113 [Source:NCBI gene (formerly Entrezgene);Acc:330503] | yes|up |
| ENSMUSG00000041986 | Elmod1 | ELMO/CED-12 domain containing 1 [Source:MGI Symbol;Acc:MGI:3583900] | yes|up |
| ENSMUSG00000047676 | Rpsa-ps10 | ribosomal protein SA, pseudogene 10 [Source:MGI Symbol;Acc:MGI:3704228] | yes|down |
| ENSMUSG00000031848 | Lsm4 | LSM4 homolog, U6 small nuclear RNA and mRNA degradation associated [Source:MGI Symbol;Acc:MGI:1354692] | yes|down |
| ENSMUSG00000118516 | Cyp2d13 | cytochrome P450, family 2, subfamily d, polypeptide 13 [Source:MGI Symbol;Acc:MGI:88605] | yes|up |
| ENSMUSG00000085645 | Hoxb5os | homeobox B5 and homeobox B6, opposite strand [Source:MGI Symbol;Acc:MGI:1922645] | yes|up |
| ENSMUSG00000036894 | Rap2b | RAP2B, member of RAS oncogene family [Source:MGI Symbol;Acc:MGI:1921262] | yes|down |
| ENSMUSG00000036896 | C1qc | complement component 1, q subcomponent, C chain [Source:MGI Symbol;Acc:MGI:88225] | yes|up |
| ENSMUSG00000056054 | S100a8 | S100 calcium binding protein A8 (calgranulin A) [Source:MGI Symbol;Acc:MGI:88244] | yes|down |
| ENSMUSG00000073791 | Efcab7 | EF-hand calcium binding domain 7 [Source:MGI Symbol;Acc:MGI:2385199] | yes|down |
| ENSMUSG00000022747 | St3gal6 | ST3 beta-galactoside alpha-2,3-sialyltransferase 6 [Source:MGI Symbol;Acc:MGI:1888707] | yes|down |
| ENSMUSG00000022748 | Cmss1 | cms small ribosomal subunit 1 [Source:MGI Symbol;Acc:MGI:1913747] | yes|down |
| ENSMUSG00000031262 | Cenpi | centromere protein I [Source:MGI Symbol;Acc:MGI:2147897] | yes|down |
| ENSMUSG00000020451 | Limk2 | LIM motif-containing protein kinase 2 [Source:MGI Symbol;Acc:MGI:1197517] | yes|up |
| ENSMUSG00000104413 | Gm37065 | predicted gene, 37065 [Source:MGI Symbol;Acc:MGI:5610293] | yes|down |
| ENSMUSG00000102324 | Gm19721 | predicted gene, 19721 [Source:MGI Symbol;Acc:MGI:5011906] | yes|up |
| ENSMUSG00000039457 | Ppl | periplakin [Source:MGI Symbol;Acc:MGI:1194898] | yes|down |
| ENSMUSG00000029687 | Ezh2 | enhancer of zeste 2 polycomb repressive complex 2 subunit [Source:MGI Symbol;Acc:MGI:107940] | yes|down |
| ENSMUSG00000095526 | Gm10243 | predicted gene 10243 [Source:MGI Symbol;Acc:MGI:3704266] | yes|up |
| ENSMUSG00000025213 | Kazald1 | Kazal-type serine peptidase inhibitor domain 1 [Source:MGI Symbol;Acc:MGI:2147606] | yes|down |
| ENSMUSG00000023908 | Pkmyt1 | protein kinase, membrane associated tyrosine/threonine 1 [Source:MGI Symbol;Acc:MGI:2137630] | yes|down |
| ENSMUSG00000068396 | Rpl34-ps1 | ribosomal protein L34, pseudogene 1 [Source:MGI Symbol;Acc:MGI:3704270] | yes|up |
| ENSMUSG00000068397 | Gm10240 | predicted gene 10240 [Source:MGI Symbol;Acc:MGI:3704454] | yes|down |
| ENSMUSG00000045636 | Mtus1 | mitochondrial tumor suppressor 1 [Source:MGI Symbol;Acc:MGI:2142572] | yes|up |
| ENSMUSG00000102813 | Gm37795 | predicted gene, 37795 [Source:MGI Symbol;Acc:MGI:5611023] | yes|up |
| ENSMUSG00000021469 | Msx2 | msh homeobox 2 [Source:MGI Symbol;Acc:MGI:97169] | yes|down |
| ENSMUSG00000045180 | Shroom2 | shroom family member 2 [Source:MGI Symbol;Acc:MGI:107194] | yes|up |
| ENSMUSG00000038721 | Hoxb7 | homeobox B7 [Source:MGI Symbol;Acc:MGI:96188] | yes|up |
| ENSMUSG00000032184 | Lysmd2 | LysM, putative peptidoglycan-binding, domain containing 2 [Source:MGI Symbol;Acc:MGI:1917332] | yes|down |
| ENSMUSG00000107379 | Gm43126 | predicted gene 43126 [Source:MGI Symbol;Acc:MGI:5663263] | yes|down |
| ENSMUSG00000105366 | Gm43719 | predicted gene 43719 [Source:MGI Symbol;Acc:MGI:5663856] | yes|down |
| ENSMUSG00000120162 |  | novel transcript | yes|up |
| ENSMUSG00000030131 | Mug2 | murinoglobulin 2 [Source:MGI Symbol;Acc:MGI:99836] | yes|up |
| ENSMUSG00000074882 | Cyp2c68 | cytochrome P450, family 2, subfamily c, polypeptide 68 [Source:MGI Symbol;Acc:MGI:3612287] | yes|up |
| ENSMUSG00000086924 | Gm11766 | predicted gene 11766 [Source:MGI Symbol;Acc:MGI:3650673] | yes|up |
| ENSMUSG00000121149 |  | novel transcript, antisense to Btbd19 | yes|up |
| ENSMUSG00000020330 | Hmmr | hyaluronan mediated motility receptor (RHAMM) [Source:MGI Symbol;Acc:MGI:104667] | yes|down |
| ENSMUSG00000027456 | Sdcbp2 | syndecan binding protein (syntenin) 2 [Source:MGI Symbol;Acc:MGI:2385156] | yes|down |
| ENSMUSG00000027454 | Gins1 | GINS complex subunit 1 (Psf1 homolog) [Source:MGI Symbol;Acc:MGI:1916520] | yes|down |
| ENSMUSG00000024810 | Il33 | interleukin 33 [Source:MGI Symbol;Acc:MGI:1924375] | yes|up |
| ENSMUSG00000097277 | 2900076A07Rik | RIKEN cDNA 2900076A07 gene [Source:MGI Symbol;Acc:MGI:1920242] | yes|up |
| ENSMUSG00000050530 | Fam171a1 | family with sequence similarity 171, member A1 [Source:MGI Symbol;Acc:MGI:2442917] | yes|down |
| ENSMUSG00000037110 | Ralgapa2 | Ral GTPase activating protein, alpha subunit 2 (catalytic) [Source:MGI Symbol;Acc:MGI:3036245] | yes|down |
| ENSMUSG00000001020 | S100a4 | S100 calcium binding protein A4 [Source:MGI Symbol;Acc:MGI:1330282] | yes|down |
| ENSMUSG00000030726 | Pold3 | polymerase (DNA-directed), delta 3, accessory subunit [Source:MGI Symbol;Acc:MGI:1915217] | yes|down |
| ENSMUSG00000056917 | Sipa1 | signal-induced proliferation associated gene 1 [Source:MGI Symbol;Acc:MGI:107576] | yes|down |
| ENSMUSG00000032725 | Folr2 | folate receptor 2 (fetal) [Source:MGI Symbol;Acc:MGI:95569] | yes|up |
| ENSMUSG00000033632 | AW554918 | expressed sequence AW554918 [Source:MGI Symbol;Acc:MGI:2147376] | yes|down |
| ENSMUSG00000039601 | Rcan2 | regulator of calcineurin 2 [Source:MGI Symbol;Acc:MGI:1858219] | yes|up |
| ENSMUSG00000033634 | Nat8f2 | N-acetyltransferase 8 (GCN5-related) family member 2 [Source:MGI Symbol;Acc:MGI:2136446] | yes|up |
| ENSMUSG00000041840 | Haus1 | HAUS augmin-like complex, subunit 1 [Source:MGI Symbol;Acc:MGI:2385076] | yes|down |
| ENSMUSG00000041842 | Fhdc1 | FH2 domain containing 1 [Source:MGI Symbol;Acc:MGI:2684972] | yes|down |
| ENSMUSG00000031451 | Gas6 | growth arrest specific 6 [Source:MGI Symbol;Acc:MGI:95660] | yes|up |
| ENSMUSG00000090053 | Pakap | paralemmin A kinase anchor protein [Source:MGI Symbol;Acc:MGI:5141924] | yes|up |
| ENSMUSG00000119972 |  | novel transcript | yes|down |
| ENSMUSG00000034009 | Rxfp1 | relaxin/insulin-like family peptide receptor 1 [Source:MGI Symbol;Acc:MGI:2682211] | yes|up |
| ENSMUSG00000031673 | Cdh11 | cadherin 11 [Source:MGI Symbol;Acc:MGI:99217] | yes|up |
| ENSMUSG00000071454 | Dtnb | dystrobrevin, beta [Source:MGI Symbol;Acc:MGI:1203728] | yes|down |
| ENSMUSG00000026417 | Pigr | polymeric immunoglobulin receptor [Source:MGI Symbol;Acc:MGI:103080] | yes|up |
| ENSMUSG00000023176 | Cpn2 | carboxypeptidase N, polypeptide 2 [Source:MGI Symbol;Acc:MGI:1919006] | yes|up |
| ENSMUSG00000071451 | Psmg4 | proteasome (prosome, macropain) assembly chaperone 4 [Source:MGI Symbol;Acc:MGI:1916916] | yes|down |
| ENSMUSG00000106951 | 5930430L01Rik | RIKEN cDNA 5930430L01 gene [Source:MGI Symbol;Acc:MGI:2443110] | yes|down |
| ENSMUSG00000067279 | Ppp1r3c | protein phosphatase 1, regulatory subunit 3C [Source:MGI Symbol;Acc:MGI:1858229] | yes|up |
| ENSMUSG00000067276 | Capn6 | calpain 6 [Source:MGI Symbol;Acc:MGI:1100850] | yes|up |
| ENSMUSG00000118181 | Gm53015 | predicted gene, 53015 [Source:MGI Symbol;Acc:MGI:6388904] | yes|down |
| ENSMUSG00000056267 | Cep70 | centrosomal protein 70 [Source:MGI Symbol;Acc:MGI:1915371] | yes|down |
| ENSMUSG00000074794 | Arrdc3 | arrestin domain containing 3 [Source:MGI Symbol;Acc:MGI:2145242] | yes|down |
| ENSMUSG00000074793 | Hspa12b | heat shock protein 12B [Source:MGI Symbol;Acc:MGI:1919880] | yes|down |
| ENSMUSG00000063953 | Amd2 | S-adenosylmethionine decarboxylase 2 [Source:MGI Symbol;Acc:MGI:1333111] | yes|down |
| ENSMUSG00000063954 | H2ac19 | H2A clustered histone 19 [Source:MGI Symbol;Acc:MGI:2448283] | yes|up |
| ENSMUSG00000083833 | Gm13841 | predicted gene 13841 [Source:MGI Symbol;Acc:MGI:3650890] | yes|down |
| ENSMUSG00000047443 | Erfe | erythroferrone [Source:MGI Symbol;Acc:MGI:3606476] | yes|up |
| ENSMUSG00000021485 | Mxd3 | Max dimerization protein 3 [Source:MGI Symbol;Acc:MGI:104987] | yes|down |
| ENSMUSG00000074479 | Rtraf-ps | RNA transcription, translation and transport factor, pseudogene [Source:MGI Symbol;Acc:MGI:3642446] | yes|down |
| ENSMUSG00000035351 | Nup37 | nucleoporin 37 [Source:MGI Symbol;Acc:MGI:1919964] | yes|down |
| ENSMUSG00000035357 | Pdzrn3 | PDZ domain containing RING finger 3 [Source:MGI Symbol;Acc:MGI:1933157] | yes|up |
| ENSMUSG00000117406 | Ntn3 | netrin 3 [Source:MGI Symbol;Acc:MGI:1341188] | yes|up |
| ENSMUSG00000074183 | Gsta1 | glutathione S-transferase, alpha 1 (Ya) [Source:MGI Symbol;Acc:MGI:1095417] | yes|down |
| ENSMUSG00000028542 | Slc6a9 | solute carrier family 6 (neurotransmitter transporter, glycine), member 9 [Source:MGI Symbol;Acc:MGI:95760] | yes|up |
| ENSMUSG00000028011 | Tdo2 | tryptophan 2,3-dioxygenase [Source:MGI Symbol;Acc:MGI:1928486] | yes|up |
| ENSMUSG00000028549 | Itgb3bp | integrin beta 3 binding protein (beta3-endonexin) [Source:MGI Symbol;Acc:MGI:1914983] | yes|down |
| ENSMUSG00000034912 | Mdga2 | MAM domain containing glycosylphosphatidylinositol anchor 2 [Source:MGI Symbol;Acc:MGI:2444706] | yes|down |
| ENSMUSG00000052565 | H1f3 | H1.3 linker histone, cluster member [Source:MGI Symbol;Acc:MGI:107502] | yes|up |
| ENSMUSG00000092545 | Gm20319 | predicted gene, 20319 [Source:MGI Symbol;Acc:MGI:5012504] | yes|up |
| ENSMUSG00000018427 | Ypel2 | yippee like 2 [Source:MGI Symbol;Acc:MGI:1925114] | yes|up |
| ENSMUSG00000053965 | Pde5a | phosphodiesterase 5A, cGMP-specific [Source:MGI Symbol;Acc:MGI:2651499] | yes|up |
| ENSMUSG00000115074 | Ndor1 | NADPH dependent diflavin oxidoreductase 1 [Source:MGI Symbol;Acc:MGI:1926047] | yes|up |
| ENSMUSG00000052305 | Hbb-bs | hemoglobin, beta adult s chain [Source:MGI Symbol;Acc:MGI:5474852] | yes|up |
| ENSMUSG00000039187 | Fanci | Fanconi anemia, complementation group I [Source:MGI Symbol;Acc:MGI:2384790] | yes|down |
| ENSMUSG00000032374 | Plod2 | procollagen lysine, 2-oxoglutarate 5-dioxygenase 2 [Source:MGI Symbol;Acc:MGI:1347007] | yes|up |
| ENSMUSG00000013367 | Iglon5 | IgLON family member 5 [Source:MGI Symbol;Acc:MGI:2686277] | yes|up |
| ENSMUSG00000110980 | Gm47204 | predicted gene, 47204 [Source:MGI Symbol;Acc:MGI:6096003] | yes|down |
| ENSMUSG00000036186 | Dipk1b | divergent protein kinase domain 1B [Source:MGI Symbol;Acc:MGI:1927576] | yes|down |
| ENSMUSG00000033355 | Rtp4 | receptor transporter protein 4 [Source:MGI Symbol;Acc:MGI:1915025] | yes|up |
| ENSMUSG00000036181 | H1f2 | H1.2 linker histone, cluster member [Source:MGI Symbol;Acc:MGI:1931526] | yes|up |
| ENSMUSG00000079491 | H2-T10 | histocompatibility 2, T region locus 10 [Source:MGI Symbol;Acc:MGI:95942] | yes|up |
| ENSMUSG00000044393 | Dsg2 | desmoglein 2 [Source:MGI Symbol;Acc:MGI:1196466] | yes|up |
| ENSMUSG00000037580 | Gch1 | GTP cyclohydrolase 1 [Source:MGI Symbol;Acc:MGI:95675] | yes|up |
| ENSMUSG00000049044 | Rapgef4 | Rap guanine nucleotide exchange factor (GEF) 4 [Source:MGI Symbol;Acc:MGI:1917723] | yes|up |
| ENSMUSG00000026646 | Suv39h2 | suppressor of variegation 3-9 2 [Source:MGI Symbol;Acc:MGI:1890396] | yes|down |
| ENSMUSG00000036452 | Arhgap26 | Rho GTPase activating protein 26 [Source:MGI Symbol;Acc:MGI:1918552] | yes|up |
| ENSMUSG00000116972 | Gm6278 | predicted gene 6278 [Source:MGI Symbol;Acc:MGI:3644862] | yes|down |
| ENSMUSG00000030111 | A2m | alpha-2-macroglobulin [Source:MGI Symbol;Acc:MGI:2449119] | yes|up |
| ENSMUSG00000030446 | Zfp273 | zinc finger protein 273 [Source:MGI Symbol;Acc:MGI:3036278] | yes|down |
| ENSMUSG00000097028 | Ptgs2os | prostaglandin-endoperoxide synthase 2, opposite strand [Source:MGI Symbol;Acc:MGI:2443180] | yes|down |
| ENSMUSG00000110569 | Gm18860 | predicted gene, 18860 [Source:MGI Symbol;Acc:MGI:5011045] | yes|down |
| ENSMUSG00000007080 | Pole | polymerase (DNA directed), epsilon [Source:MGI Symbol;Acc:MGI:1196391] | yes|down |
| ENSMUSG00000027661 | Slc2a10 | solute carrier family 2 (facilitated glucose transporter), member 10 [Source:MGI Symbol;Acc:MGI:2156687] | yes|up |
| ENSMUSG00000027843 | Ptpn22 | protein tyrosine phosphatase, non-receptor type 22 (lymphoid) [Source:MGI Symbol;Acc:MGI:107170] | yes|down |
| ENSMUSG00000031860 | Pbx4 | pre B cell leukemia homeobox 4 [Source:MGI Symbol;Acc:MGI:1931321] | yes|down |
| ENSMUSG00000031861 | Lpar2 | lysophosphatidic acid receptor 2 [Source:MGI Symbol;Acc:MGI:1858422] | yes|down |
| ENSMUSG00000004371 | Il11 | interleukin 11 [Source:MGI Symbol;Acc:MGI:107613] | yes|down |
| ENSMUSG00000056073 | Grik2 | glutamate receptor, ionotropic, kainate 2 (beta 2) [Source:MGI Symbol;Acc:MGI:95815] | yes|down |
| ENSMUSG00000022766 | Serpind1 | serine (or cysteine) peptidase inhibitor, clade D, member 1 [Source:MGI Symbol;Acc:MGI:96051] | yes|up |
| ENSMUSG00000090555 | Gm8893 | predicted gene 8893 [Source:MGI Symbol;Acc:MGI:3779818] | yes|up |
| ENSMUSG00000107962 | Gm43980 | predicted gene, 43980 [Source:MGI Symbol;Acc:MGI:5690372] | yes|up |
| ENSMUSG00000104434 | Gm37421 | predicted gene, 37421 [Source:MGI Symbol;Acc:MGI:5610649] | yes|up |
| ENSMUSG00000098915 | Rpl15-ps2 | ribosomal protein L15, pseudogene 2 [Source:MGI Symbol;Acc:MGI:3648255] | yes|down |
| ENSMUSG00000039476 | Prrx2 | paired related homeobox 2 [Source:MGI Symbol;Acc:MGI:98218] | yes|down |
| ENSMUSG00000009687 | Fxyd5 | FXYD domain-containing ion transport regulator 5 [Source:MGI Symbol;Acc:MGI:1201785] | yes|down |
| ENSMUSG00000095098 | Ccdc85b | coiled-coil domain containing 85B [Source:MGI Symbol;Acc:MGI:2147607] | yes|down |
| ENSMUSG00000089678 | Agxt2 | alanine-glyoxylate aminotransferase 2 [Source:MGI Symbol;Acc:MGI:2146052] | yes|up |
| ENSMUSG00000074639 | Rdh16f2 | RDH16 family member 2 [Source:MGI Symbol;Acc:MGI:3583955] | yes|up |
| ENSMUSG00000074637 | Sox2 | SRY (sex determining region Y)-box 2 [Source:MGI Symbol;Acc:MGI:98364] | yes|down |
| ENSMUSG00000074634 | Tmem267 | transmembrane protein 267 [Source:MGI Symbol;Acc:MGI:3648543] | yes|down |
| ENSMUSG00000039063 | Echdc3 | enoyl Coenzyme A hydratase domain containing 3 [Source:MGI Symbol;Acc:MGI:1915106] | yes|down |
| ENSMUSG00000039065 | Atpsckmt | ATP synthase C subunit lysine N-methyltransferase [Source:MGI Symbol;Acc:MGI:1915323] | yes|down |
| ENSMUSG00000023963 | Cyp39a1 | cytochrome P450, family 39, subfamily a, polypeptide 1 [Source:MGI Symbol;Acc:MGI:1927096] | yes|up |
| ENSMUSG00000023960 | Enpp5 | ectonucleotide pyrophosphatase/phosphodiesterase 5 [Source:MGI Symbol;Acc:MGI:1933830] | yes|up |
| ENSMUSG00000028970 | Abcb1b | ATP-binding cassette, sub-family B (MDR/TAP), member 1B [Source:MGI Symbol;Acc:MGI:97568] | yes|down |
| ENSMUSG00000100658 | F730311O21Rik | RIKEN cDNA F730311O21 gene [Source:MGI Symbol;Acc:MGI:3643355] | yes|down |
| ENSMUSG00000068115 | Ninl | ninein-like [Source:MGI Symbol;Acc:MGI:1925427] | yes|down |
| ENSMUSG00000035168 | Tanc1 | tetratricopeptide repeat, ankyrin repeat and coiled-coil containing 1 [Source:MGI Symbol;Acc:MGI:1914110] | yes|up |
| ENSMUSG00000042462 | Dctpp1 | dCTP pyrophosphatase 1 [Source:MGI Symbol;Acc:MGI:1913672] | yes|down |
| ENSMUSG00000053552 | Ebf4 | early B cell factor 4 [Source:MGI Symbol;Acc:MGI:2385972] | yes|up |
| ENSMUSG00000019942 | Cdk1 | cyclin-dependent kinase 1 [Source:MGI Symbol;Acc:MGI:88351] | yes|down |
| ENSMUSG00000033831 | Fgb | fibrinogen beta chain [Source:MGI Symbol;Acc:MGI:99501] | yes|up |
| ENSMUSG00000073705 | Cenps | centromere protein S [Source:MGI Symbol;Acc:MGI:1917178] | yes|down |
| ENSMUSG00000064115 | Cadm2 | cell adhesion molecule 2 [Source:MGI Symbol;Acc:MGI:2442722] | yes|up |
| ENSMUSG00000079852 | Klra4 | killer cell lectin-like receptor, subfamily A, member 4 [Source:MGI Symbol;Acc:MGI:101904] | yes|down |
| ENSMUSG00000001228 | Uhrf1 | ubiquitin-like, containing PHD and RING finger domains, 1 [Source:MGI Symbol;Acc:MGI:1338889] | yes|down |
| ENSMUSG00000078452 | Raet1d | retinoic acid early transcript delta [Source:MGI Symbol;Acc:MGI:1861032] | yes|down |
| ENSMUSG00000048251 | Bcl11b | B cell leukemia/lymphoma 11B [Source:MGI Symbol;Acc:MGI:1929913] | yes|up |
| ENSMUSG00000058022 | Adtrp | androgen dependent TFPI regulating protein [Source:MGI Symbol;Acc:MGI:1924596] | yes|up |
| ENSMUSG00000008384 | Sertad1 | SERTA domain containing 1 [Source:MGI Symbol;Acc:MGI:1913438] | yes|down |
| ENSMUSG00000026785 | Pkn3 | protein kinase N3 [Source:MGI Symbol;Acc:MGI:2388285] | yes|down |
| ENSMUSG00000026786 | Apbb1ip | amyloid beta (A4) precursor protein-binding, family B, member 1 interacting protein [Source:MGI Symbol;Acc:MGI:1861354] | yes|down |
| ENSMUSG00000015652 | Steap1 | six transmembrane epithelial antigen of the prostate 1 [Source:MGI Symbol;Acc:MGI:1917608] | yes|down |
| ENSMUSG00000019768 | Esr1 | estrogen receptor 1 (alpha) [Source:MGI Symbol;Acc:MGI:1352467] | yes|down |
| ENSMUSG00000015656 | Hspa8 | heat shock protein 8 [Source:MGI Symbol;Acc:MGI:105384] | yes|down |
| ENSMUSG00000037206 | Islr | immunoglobulin superfamily containing leucine-rich repeat [Source:MGI Symbol;Acc:MGI:1349645] | yes|up |
| ENSMUSG00000026259 | Ngef | neuronal guanine nucleotide exchange factor [Source:MGI Symbol;Acc:MGI:1858414] | yes|down |
| ENSMUSG00000019761 | Krt10 | keratin 10 [Source:MGI Symbol;Acc:MGI:96685] | yes|down |
| ENSMUSG00000034023 | Fancd2 | Fanconi anemia, complementation group D2 [Source:MGI Symbol;Acc:MGI:2448480] | yes|down |
| ENSMUSG00000026437 | Cdk18 | cyclin-dependent kinase 18 [Source:MGI Symbol;Acc:MGI:97518] | yes|down |
| ENSMUSG00000026435 | Slc45a3 | solute carrier family 45, member 3 [Source:MGI Symbol;Acc:MGI:1922082] | yes|up |
| ENSMUSG00000071478 | H2ac7 | H2A clustered histone 7 [Source:MGI Symbol;Acc:MGI:2448289] | yes|up |
| ENSMUSG00000030306 | Tmtc1 | transmembrane and tetratricopeptide repeat containing 1 [Source:MGI Symbol;Acc:MGI:3039590] | yes|up |
| ENSMUSG00000015243 | Abca1 | ATP-binding cassette, sub-family A (ABC1), member 1 [Source:MGI Symbol;Acc:MGI:99607] | yes|up |
| ENSMUSG00000056531 | Ccdc18 | coiled-coil domain containing 18 [Source:MGI Symbol;Acc:MGI:1922974] | yes|down |
| ENSMUSG00000094622 | Gm3055 | predicted gene 3055 [Source:MGI Symbol;Acc:MGI:3809197] | yes|down |
| ENSMUSG00000005836 | Gata6 | GATA binding protein 6 [Source:MGI Symbol;Acc:MGI:107516] | yes|up |
| ENSMUSG00000029032 | Arhgef16 | Rho guanine nucleotide exchange factor (GEF) 16 [Source:MGI Symbol;Acc:MGI:2446219] | yes|up |
| ENSMUSG00000047428 | Dlk2 | delta like non-canonical Notch ligand 2 [Source:MGI Symbol;Acc:MGI:2146838] | yes|down |
| ENSMUSG00000047420 | Fam180a | family with sequence similarity 180, member A [Source:MGI Symbol;Acc:MGI:3039626] | yes|up |
| ENSMUSG00000105112 | Gm42778 | predicted gene 42778 [Source:MGI Symbol;Acc:MGI:5662915] | yes|up |
| ENSMUSG00000097820 | E530011L22Rik | RIKEN cDNA E530011L22 gene [Source:MGI Symbol;Acc:MGI:2443770] | yes|down |
| ENSMUSG00000038740 | Mvb12b | multivesicular body subunit 12B [Source:MGI Symbol;Acc:MGI:1919793] | yes|down |
| ENSMUSG00000034687 | Fras1 | Fraser extracellular matrix complex subunit 1 [Source:MGI Symbol;Acc:MGI:2385368] | yes|up |
| ENSMUSG00000028789 | Azin2 | antizyme inhibitor 2 [Source:MGI Symbol;Acc:MGI:2442093] | yes|up |
| ENSMUSG00000022206 | Npr3 | natriuretic peptide receptor 3 [Source:MGI Symbol;Acc:MGI:97373] | yes|up |
| ENSMUSG00000022203 | Efs | embryonal Fyn-associated substrate [Source:MGI Symbol;Acc:MGI:105311] | yes|up |
| ENSMUSG00000033533 | Acsm1 | acyl-CoA synthetase medium-chain family member 1 [Source:MGI Symbol;Acc:MGI:2152200] | yes|up |
| ENSMUSG00000057113 | Npm1 | nucleophosmin 1 [Source:MGI Symbol;Acc:MGI:106184] | yes|down |
| ENSMUSG00000117422 | CJ186046Rik | Riken cDNA CJ186046 gene [Source:MGI Symbol;Acc:MGI:5319478] | yes|down |
| ENSMUSG00000110340 | 1600027J07Rik | RIKEN cDNA 1600027J07 gene [Source:MGI Symbol;Acc:MGI:1917044] | yes|up |
| ENSMUSG00000025001 | Hells | helicase, lymphoid specific [Source:MGI Symbol;Acc:MGI:106209] | yes|down |
| ENSMUSG00000028560 | Usp1 | ubiquitin specific peptidase 1 [Source:MGI Symbol;Acc:MGI:2385198] | yes|down |
| ENSMUSG00000028553 | Angptl3 | angiopoietin-like 3 [Source:MGI Symbol;Acc:MGI:1353627] | yes|up |
| ENSMUSG00000043259 | Fam13c | family with sequence similarity 13, member C [Source:MGI Symbol;Acc:MGI:1918971] | yes|up |
| ENSMUSG00000094081 | Gm20826 | predicted gene, 20826 [Source:MGI Symbol;Acc:MGI:5434182] | yes|up |
| ENSMUSG00000032352 | Lrrc1 | leucine rich repeat containing 1 [Source:MGI Symbol;Acc:MGI:2442313] | yes|down |
| ENSMUSG00000074971 | Fibin | fin bud initiation factor homolog (zebrafish) [Source:MGI Symbol;Acc:MGI:1914856] | yes|up |
| ENSMUSG00000017716 | Birc5 | baculoviral IAP repeat-containing 5 [Source:MGI Symbol;Acc:MGI:1203517] | yes|down |
| ENSMUSG00000074182 | Znhit6 | zinc finger, HIT type 6 [Source:MGI Symbol;Acc:MGI:1916996] | yes|down |
| ENSMUSG00000021835 | Bmp4 | bone morphogenetic protein 4 [Source:MGI Symbol;Acc:MGI:88180] | yes|up |
| ENSMUSG00000021831 | Ero1a | endoplasmic reticulum oxidoreductase 1 alpha [Source:MGI Symbol;Acc:MGI:1354385] | yes|up |
| ENSMUSG00000042501 | Cpa6 | carboxypeptidase A6 [Source:MGI Symbol;Acc:MGI:3045348] | yes|up |
| ENSMUSG00000062609 | Kcnj15 | potassium inwardly-rectifying channel, subfamily J, member 15 [Source:MGI Symbol;Acc:MGI:1310000] | yes|up |
| ENSMUSG00000073234 | Gm8773 | predicted gene 8773 [Source:MGI Symbol;Acc:MGI:3646213] | yes|down |
| ENSMUSG00000026715 | Serpinc1 | serine (or cysteine) peptidase inhibitor, clade C (antithrombin), member 1 [Source:MGI Symbol;Acc:MGI:88095] | yes|up |
| ENSMUSG00000024660 | Incenp | inner centromere protein [Source:MGI Symbol;Acc:MGI:1313288] | yes|down |
| ENSMUSG00000024663 | Rab3il1 | RAB3A interacting protein (rabin3)-like 1 [Source:MGI Symbol;Acc:MGI:1922010] | yes|up |
| ENSMUSG00000079465 | Col4a3 | collagen, type IV, alpha 3 [Source:MGI Symbol;Acc:MGI:104688] | yes|up |
| ENSMUSG00000032826 | Ank2 | ankyrin 2, brain [Source:MGI Symbol;Acc:MGI:88025] | yes|up |
| ENSMUSG00000030680 | Pagr1a | PAXIP1 associated glutamate rich protein 1A [Source:MGI Symbol;Acc:MGI:1914528] | yes|up |
| ENSMUSG00000033488 | Cryzl2 | crystallin zeta like 2 [Source:MGI Symbol;Acc:MGI:2448516] | yes|up |
| ENSMUSG00000057914 | Cacnb2 | calcium channel, voltage-dependent, beta 2 subunit [Source:MGI Symbol;Acc:MGI:894644] | yes|down |
| ENSMUSG00000022840 | Adcy5 | adenylate cyclase 5 [Source:MGI Symbol;Acc:MGI:99673] | yes|up |
| ENSMUSG00000047638 | Nr1h4 | nuclear receptor subfamily 1, group H, member 4 [Source:MGI Symbol;Acc:MGI:1352464] | yes|up |
| ENSMUSG00000047631 | Apof | apolipoprotein F [Source:MGI Symbol;Acc:MGI:104539] | yes|up |
| ENSMUSG00000027318 | Adam33 | a disintegrin and metallopeptidase domain 33 [Source:MGI Symbol;Acc:MGI:1341813] | yes|up |
| ENSMUSG00000027869 | Hsd3b6 | hydroxy-delta-5-steroid dehydrogenase, 3 beta- and steroid delta-isomerase 6 [Source:MGI Symbol;Acc:MGI:109598] | yes|down |
| ENSMUSG00000027641 | Rbl1 | RB transcriptional corepressor like 1 [Source:MGI Symbol;Acc:MGI:103300] | yes|down |
| ENSMUSG00000041498 | Kif14 | kinesin family member 14 [Source:MGI Symbol;Acc:MGI:1098226] | yes|down |
| ENSMUSG00000041491 | Cep78 | centrosomal protein 78 [Source:MGI Symbol;Acc:MGI:1924386] | yes|down |
| ENSMUSG00000052302 | Tbc1d30 | TBC1 domain family, member 30 [Source:MGI Symbol;Acc:MGI:1921944] | yes|up |
| ENSMUSG00000096935 | 1700113A16Rik | RIKEN cDNA 1700113A16 gene [Source:MGI Symbol;Acc:MGI:1923892] | yes|down |
| ENSMUSG00000020032 | Nuak1 | NUAK family, SNF1-like kinase, 1 [Source:MGI Symbol;Acc:MGI:1925226] | yes|up |
| ENSMUSG00000115700 | Gm7517 | predicted gene 7517 [Source:MGI Symbol;Acc:MGI:3643004] | yes|down |
| ENSMUSG00000046949 | Nqo2 | N-ribosyldihydronicotinamide quinone reductase 2 [Source:MGI Symbol;Acc:MGI:104513] | yes|down |
| ENSMUSG00000098934 | Gvin-ps4 | GTPase, very large interferon inducible, pseudogene 4 [Source:MGI Symbol;Acc:MGI:5011038] | yes|down |
| ENSMUSG00000073640 | Rpl27-ps3 | ribosomal protein L27, pseudogene 3 [Source:MGI Symbol;Acc:MGI:3646174] | yes|down |
| ENSMUSG00000052155 | Acvr2a | activin receptor IIA [Source:MGI Symbol;Acc:MGI:102806] | yes|up |
| ENSMUSG00000039196 | Orm1 | orosomucoid 1 [Source:MGI Symbol;Acc:MGI:97443] | yes|up |
| ENSMUSG00000047894 | Ang2 | angiogenin, ribonuclease A family, member 2 [Source:MGI Symbol;Acc:MGI:104984] | yes|up |
| ENSMUSG00000062181 | Ces3b | carboxylesterase 3B [Source:MGI Symbol;Acc:MGI:3644960] | yes|up |
| ENSMUSG00000034762 | Glis1 | GLIS family zinc finger 1 [Source:MGI Symbol;Acc:MGI:2386723] | yes|down |
| ENSMUSG00000034765 | Dusp5 | dual specificity phosphatase 5 [Source:MGI Symbol;Acc:MGI:2685183] | yes|down |
| ENSMUSG00000029570 | Lfng | LFNG O-fucosylpeptide 3-beta-N-acetylglucosaminyltransferase [Source:MGI Symbol;Acc:MGI:1095413] | yes|down |
| ENSMUSG00000023940 | Sgo1 | shugoshin 1 [Source:MGI Symbol;Acc:MGI:1919665] | yes|down |
| ENSMUSG00000023945 | Slc5a7 | solute carrier family 5 (choline transporter), member 7 [Source:MGI Symbol;Acc:MGI:1927126] | yes|up |
| ENSMUSG00000069516 | Lyz2 | lysozyme 2 [Source:MGI Symbol;Acc:MGI:96897] | yes|up |
| ENSMUSG00000042662 | Dusp15 | dual specificity phosphatase-like 15 [Source:MGI Symbol;Acc:MGI:1934928] | yes|up |
| ENSMUSG00000083246 | Gm11839 | predicted gene 11839 [Source:MGI Symbol;Acc:MGI:3651162] | yes|up |
| ENSMUSG00000044548 | Dact1 | dishevelled-binding antagonist of beta-catenin 1 [Source:MGI Symbol;Acc:MGI:1891740] | yes|up |
| ENSMUSG00000078612 | Fyb2 | FYN binding protein 2 [Source:MGI Symbol;Acc:MGI:2685466] | yes|down |
| ENSMUSG00000019961 | Tmpo | thymopoietin [Source:MGI Symbol;Acc:MGI:106920] | yes|down |
| ENSMUSG00000019960 | Dusp6 | dual specificity phosphatase 6 [Source:MGI Symbol;Acc:MGI:1914853] | yes|down |
| ENSMUSG00000113061 | Rps18-ps5 | ribosomal protein S18, pseudogene 5 [Source:MGI Symbol;Acc:MGI:3649931] | yes|up |
| ENSMUSG00000054263 | Lifr | LIF receptor alpha [Source:MGI Symbol;Acc:MGI:96788] | yes|up |
| ENSMUSG00000072893 | 4933439C10Rik | RIKEN cDNA 4933439C10 gene [Source:MGI Symbol;Acc:MGI:1921726] | yes|up |
| ENSMUSG00000033676 | Gabrb3 | gamma-aminobutyric acid (GABA) A receptor, subunit beta 3 [Source:MGI Symbol;Acc:MGI:95621] | yes|up |
| ENSMUSG00000026235 | Epha4 | Eph receptor A4 [Source:MGI Symbol;Acc:MGI:98277] | yes|up |
| ENSMUSG00000026234 | Ncl | nucleolin [Source:MGI Symbol;Acc:MGI:97286] | yes|down |
| ENSMUSG00000026238 | Ptma | prothymosin alpha [Source:MGI Symbol;Acc:MGI:97803] | yes|down |
| ENSMUSG00000030895 | Hpx | hemopexin [Source:MGI Symbol;Acc:MGI:105112] | yes|up |
| ENSMUSG00000059751 | Rps3a3 | ribosomal protein S3A3 [Source:MGI Symbol;Acc:MGI:3643406] | yes|down |
| ENSMUSG00000021226 | Acot2 | acyl-CoA thioesterase 2 [Source:MGI Symbol;Acc:MGI:2159605] | yes|up |
| ENSMUSG00000050587 | Lrrc4c | leucine rich repeat containing 4C [Source:MGI Symbol;Acc:MGI:2442636] | yes|up |
| ENSMUSG00000038668 | Lpar1 | lysophosphatidic acid receptor 1 [Source:MGI Symbol;Acc:MGI:108429] | yes|down |
| ENSMUSG00000062061 | Obp2a | odorant binding protein 2A [Source:MGI Symbol;Acc:MGI:2387617] | yes|down |
| ENSMUSG00000051235 | Gen1 | GEN1, Holliday junction 5' flap endonuclease [Source:MGI Symbol;Acc:MGI:2443149] | yes|down |
| ENSMUSG00000029019 | Nppb | natriuretic peptide type B [Source:MGI Symbol;Acc:MGI:97368] | yes|down |
| ENSMUSG00000022040 | Ephx2 | epoxide hydrolase 2, cytoplasmic [Source:MGI Symbol;Acc:MGI:99500] | yes|up |
| ENSMUSG00000020577 | Tspan13 | tetraspanin 13 [Source:MGI Symbol;Acc:MGI:1913359] | yes|up |
| ENSMUSG00000085890 | Tnfsf13os | tumor necrosis factor (ligand) superfamily, member 13, opposite strand [Source:MGI Symbol;Acc:MGI:1919587] | yes|down |
| ENSMUSG00000028766 | Alpl | alkaline phosphatase, liver/bone/kidney [Source:MGI Symbol;Acc:MGI:87983] | yes|up |
| ENSMUSG00000043091 | Tuba1c | tubulin, alpha 1C [Source:MGI Symbol;Acc:MGI:1095409] | yes|down |
| ENSMUSG00000022596 | Slurp1 | secreted Ly6/Plaur domain containing 1 [Source:MGI Symbol;Acc:MGI:1930923] | yes|down |
| ENSMUSG00000022265 | Ank | progressive ankylosis [Source:MGI Symbol;Acc:MGI:3045421] | yes|down |
| ENSMUSG00000006490 | Prl8a9 | prolactin family8, subfamily a, member 9 [Source:MGI Symbol;Acc:MGI:1914560] | yes|down |
| ENSMUSG00000072915 | Gm12258 | predicted gene 12258 [Source:MGI Symbol;Acc:MGI:3651534] | yes|up |
| ENSMUSG00000025069 | Gsto2 | glutathione S-transferase omega 2 [Source:MGI Symbol;Acc:MGI:1915464] | yes|down |
| ENSMUSG00000020681 | Ace | angiotensin I converting enzyme (peptidyl-dipeptidase A) 1 [Source:MGI Symbol;Acc:MGI:87874] | yes|down |
| ENSMUSG00000025064 | Col17a1 | collagen, type XVII, alpha 1 [Source:MGI Symbol;Acc:MGI:88450] | yes|down |
| ENSMUSG00000032202 | Rab27a | RAB27A, member RAS oncogene family [Source:MGI Symbol;Acc:MGI:1861441] | yes|up |
| ENSMUSG00000114154 | Gm48597 | predicted gene, 48597 [Source:MGI Symbol;Acc:MGI:6098174] | yes|down |
| ENSMUSG00000052520 | Cyp2j5 | cytochrome P450, family 2, subfamily j, polypeptide 5 [Source:MGI Symbol;Acc:MGI:1270149] | yes|up |
| ENSMUSG00000023015 | Racgap1 | Rac GTPase-activating protein 1 [Source:MGI Symbol;Acc:MGI:1349423] | yes|down |
| ENSMUSG00000026532 | Spta1 | spectrin alpha, erythrocytic 1 [Source:MGI Symbol;Acc:MGI:98385] | yes|up |
| ENSMUSG00000004897 | Hdgf | heparin binding growth factor [Source:MGI Symbol;Acc:MGI:1194494] | yes|down |
| ENSMUSG00000042050 | Dync2i1 | dynein 2 intermediate chain 1 [Source:MGI Symbol;Acc:MGI:2445085] | yes|down |
| ENSMUSG00000045005 | Fzd5 | frizzled class receptor 5 [Source:MGI Symbol;Acc:MGI:108571] | yes|down |
| ENSMUSG00000032332 | Col12a1 | collagen, type XII, alpha 1 [Source:MGI Symbol;Acc:MGI:88448] | yes|up |
| ENSMUSG00000023885 | Thbs2 | thrombospondin 2 [Source:MGI Symbol;Acc:MGI:98738] | yes|up |
| ENSMUSG00000017737 | Mmp9 | matrix metallopeptidase 9 [Source:MGI Symbol;Acc:MGI:97011] | yes|down |
| ENSMUSG00000021811 | Dnajc9 | DnaJ heat shock protein family (Hsp40) member C9 [Source:MGI Symbol;Acc:MGI:1915326] | yes|down |
| ENSMUSG00000055612 | Cdca7 | cell division cycle associated 7 [Source:MGI Symbol;Acc:MGI:1914203] | yes|down |
| ENSMUSG00000033318 | Gstt2 | glutathione S-transferase, theta 2 [Source:MGI Symbol;Acc:MGI:106188] | yes|up |
| ENSMUSG00000096006 | Gm21596 | predicted gene, 21596 [Source:MGI Symbol;Acc:MGI:5434951] | yes|down |
| ENSMUSG00000049001 | Ndnf | neuron-derived neurotrophic factor [Source:MGI Symbol;Acc:MGI:1915419] | yes|up |
| ENSMUSG00000032802 | Srxn1 | sulfiredoxin 1 homolog (S. cerevisiae) [Source:MGI Symbol;Acc:MGI:104971] | yes|down |
| ENSMUSG00000086513 | Gvin-ps1 | GTPase, very large interferon inducible, pseudogene 1 [Source:MGI Symbol;Acc:MGI:1924950] | yes|up |
| ENSMUSG00000113491 | Gm19221 | predicted gene, 19221 [Source:MGI Symbol;Acc:MGI:5011406] | yes|up |
| ENSMUSG00000056121 | Fez2 | fasciculation and elongation protein zeta 2 (zygin II) [Source:MGI Symbol;Acc:MGI:2675856] | yes|down |
| ENSMUSG00000117001 | Gm49858 | predicted gene, 49858 [Source:MGI Symbol;Acc:MGI:6270531] | yes|up |
| ENSMUSG00000099583 | H3c4 | H3 clustered histone 4 [Source:MGI Symbol;Acc:MGI:2448322] | yes|up |
| ENSMUSG00000036492 | Rnf39 | ring finger protein 39 [Source:MGI Symbol;Acc:MGI:2156378] | yes|down |
| ENSMUSG00000022860 | Chodl | chondrolectin [Source:MGI Symbol;Acc:MGI:2179069] | yes|up |
| ENSMUSG00000022861 | Dgkg | diacylglycerol kinase, gamma [Source:MGI Symbol;Acc:MGI:105060] | yes|up |
| ENSMUSG00000022868 | Ahsg | alpha-2-HS-glycoprotein [Source:MGI Symbol;Acc:MGI:107189] | yes|up |
| ENSMUSG00000076441 | Ass1 | argininosuccinate synthetase 1 [Source:MGI Symbol;Acc:MGI:88090] | yes|down |
| ENSMUSG00000030409 | Dmpk | dystrophia myotonica-protein kinase [Source:MGI Symbol;Acc:MGI:94906] | yes|up |
| ENSMUSG00000010175 | Prox1 | prospero homeobox 1 [Source:MGI Symbol;Acc:MGI:97772] | yes|up |
| ENSMUSG00000027624 | Epb41l1 | erythrocyte membrane protein band 4.1 like 1 [Source:MGI Symbol;Acc:MGI:103010] | yes|down |
| ENSMUSG00000070661 | Rnf186 | ring finger protein 186 [Source:MGI Symbol;Acc:MGI:1914075] | yes|up |
| ENSMUSG00000004151 | Etv1 | ets variant 1 [Source:MGI Symbol;Acc:MGI:99254] | yes|down |
| ENSMUSG00000027331 | Knstrn | kinetochore-localized astrin/SPAG5 binding [Source:MGI Symbol;Acc:MGI:1289298] | yes|down |
| ENSMUSG00000027330 | Cdc25b | cell division cycle 25B [Source:MGI Symbol;Acc:MGI:99701] | yes|down |
| ENSMUSG00000048355 | Arxes1 | adipocyte-related X-chromosome expressed sequence 1 [Source:MGI Symbol;Acc:MGI:1923469] | yes|up |
| ENSMUSG00000027339 | Rassf2 | Ras association (RalGDS/AF-6) domain family member 2 [Source:MGI Symbol;Acc:MGI:2442060] | yes|down |
| ENSMUSG00000027338 | Prnd | prion like protein doppel [Source:MGI Symbol;Acc:MGI:1346999] | yes|up |
| ENSMUSG00000026678 | Rgs5 | regulator of G-protein signaling 5 [Source:MGI Symbol;Acc:MGI:1098434] | yes|up |
| ENSMUSG00000040525 | Cblc | Casitas B-lineage lymphoma c [Source:MGI Symbol;Acc:MGI:1931457] | yes|up |
| ENSMUSG00000040296 | Ddx58 | DEAD/H box helicase 58 [Source:MGI Symbol;Acc:MGI:2442858] | yes|up |
| ENSMUSG00000011179 | Odc1 | ornithine decarboxylase, structural 1 [Source:MGI Symbol;Acc:MGI:97402] | yes|down |
| ENSMUSG00000051537 | Gm5124 | predicted pseudogene 5124 [Source:MGI Symbol;Acc:MGI:3643622] | yes|down |
| ENSMUSG00000039783 | Kmo | kynurenine 3-monooxygenase (kynurenine 3-hydroxylase) [Source:MGI Symbol;Acc:MGI:2138151] | yes|up |
| ENSMUSG00000039438 | Ttc36 | tetratricopeptide repeat domain 36 [Source:MGI Symbol;Acc:MGI:2384760] | yes|up |
| ENSMUSG00000031200 | Mtcp1 | mature T cell proliferation 1 [Source:MGI Symbol;Acc:MGI:102699] | yes|up |
| ENSMUSG00000031756 | Cenpn | centromere protein N [Source:MGI Symbol;Acc:MGI:1919405] | yes|down |
| ENSMUSG00000031209 | Heph | hephaestin [Source:MGI Symbol;Acc:MGI:1332240] | yes|up |
| ENSMUSG00000040749 | Siah1b | siah E3 ubiquitin protein ligase 1B [Source:MGI Symbol;Acc:MGI:108063] | yes|down |
| ENSMUSG00000026574 | Dpt | dermatopontin [Source:MGI Symbol;Acc:MGI:1928392] | yes|up |
| ENSMUSG00000026578 | Ccdc181 | coiled-coil domain containing 181 [Source:MGI Symbol;Acc:MGI:1922145] | yes|down |
| ENSMUSG00000085154 | C130046K22Rik | RIKEN cDNA C130046K22 gene [Source:MGI Symbol;Acc:MGI:3026935] | yes|down |
| ENSMUSG00000082519 | Vamp7-ps | vesicle-associated membrane protein 7, pseudogene [Source:MGI Symbol;Acc:MGI:3652239] | yes|up |
| ENSMUSG00000022673 | Mcm4 | minichromosome maintenance complex component 4 [Source:MGI Symbol;Acc:MGI:103199] | yes|down |
| ENSMUSG00000009646 | Pla2g12b | phospholipase A2, group XIIB [Source:MGI Symbol;Acc:MGI:1917086] | yes|up |
| ENSMUSG00000003644 | Rps6ka1 | ribosomal protein S6 kinase polypeptide 1 [Source:MGI Symbol;Acc:MGI:104558] | yes|down |
| ENSMUSG00000012483 | Rpa3 | replication protein A3 [Source:MGI Symbol;Acc:MGI:1915490] | yes|down |
| ENSMUSG00000025909 | Sntg1 | syntrophin, gamma 1 [Source:MGI Symbol;Acc:MGI:1918346] | yes|up |
| ENSMUSG00000046295 | Ankle1 | ankyrin repeat and LEM domain containing 1 [Source:MGI Symbol;Acc:MGI:1918775] | yes|down |
| ENSMUSG00000001025 | S100a6 | S100 calcium binding protein A6 (calcyclin) [Source:MGI Symbol;Acc:MGI:1339467] | yes|down |
| ENSMUSG00000029591 | Ung | uracil DNA glycosylase [Source:MGI Symbol;Acc:MGI:109352] | yes|down |
| ENSMUSG00000029597 | Sds | serine dehydratase [Source:MGI Symbol;Acc:MGI:98270] | yes|up |
| ENSMUSG00000029596 | Sdsl | serine dehydratase-like [Source:MGI Symbol;Acc:MGI:2182607] | yes|up |
| ENSMUSG00000047216 | Cdh19 | cadherin 19, type 2 [Source:MGI Symbol;Acc:MGI:3588198] | yes|up |
| ENSMUSG00000042606 | Hirip3 | HIRA interacting protein 3 [Source:MGI Symbol;Acc:MGI:2142364] | yes|down |
| ENSMUSG00000042607 | Asb4 | ankyrin repeat and SOCS box-containing 4 [Source:MGI Symbol;Acc:MGI:1929751] | yes|up |
| ENSMUSG00000042604 | Kcna4 | potassium voltage-gated channel, shaker-related subfamily, member 4 [Source:MGI Symbol;Acc:MGI:96661] | yes|down |
| ENSMUSG00000049929 | Lpar4 | lysophosphatidic acid receptor 4 [Source:MGI Symbol;Acc:MGI:1925384] | yes|up |
| ENSMUSG00000046743 | Fat4 | FAT atypical cadherin 4 [Source:MGI Symbol;Acc:MGI:3045256] | yes|up |
| ENSMUSG00000109829 | Gm45605 | predicted gene 45605 [Source:MGI Symbol;Acc:MGI:5791441] | yes|up |
| ENSMUSG00000068335 | Dok1 | docking protein 1 [Source:MGI Symbol;Acc:MGI:893587] | yes|down |
| ENSMUSG00000035121 | Neil2 | nei like 2 (E. coli) [Source:MGI Symbol;Acc:MGI:2686058] | yes|up |
| ENSMUSG00000035439 | Haus8 | 4HAUS augmin-like complex, subunit 8 [Source:MGI Symbol;Acc:MGI:1923728] | yes|down |
| ENSMUSG00000000049 | Apoh | apolipoprotein H [Source:MGI Symbol;Acc:MGI:88058] | yes|up |
| ENSMUSG00000049420 | Tmem200a | transmembrane protein 200A [Source:MGI Symbol;Acc:MGI:1924470] | yes|down |
| ENSMUSG00000100963 | Gm28372 | predicted gene 28372 [Source:MGI Symbol;Acc:MGI:5579078] | yes|up |
| ENSMUSG00000078674 | Mup18 | major urinary protein 18 [Source:MGI Symbol;Acc:MGI:3705220] | yes|up |
| ENSMUSG00000078672 | Mup20 | major urinary protein 20 [Source:MGI Symbol;Acc:MGI:3651981] | yes|up |
| ENSMUSG00000078673 | Mup19 | major urinary protein 19 [Source:MGI Symbol;Acc:MGI:3705235] | yes|up |
| ENSMUSG00000078670 | Fam174b | family with sequence similarity 174, member B [Source:MGI Symbol;Acc:MGI:3698178] | yes|up |
| ENSMUSG00000039607 | Rbms3 | RNA binding motif, single stranded interacting protein [Source:MGI Symbol;Acc:MGI:2444477] | yes|up |
| ENSMUSG00000045211 | Nudt18 | nudix (nucleoside diphosphate linked moiety X)-type motif 18 [Source:MGI Symbol;Acc:MGI:2385853] | yes|up |
| ENSMUSG00000121127 |  | novel transcript | yes|down |
| ENSMUSG00000070473 | Cldn3 | claudin 3 [Source:MGI Symbol;Acc:MGI:1329044] | yes|up |
| ENSMUSG00000020354 | Sgcd | sarcoglycan, delta (dystrophin-associated glycoprotein) [Source:MGI Symbol;Acc:MGI:1346525] | yes|down |
| ENSMUSG00000113261 | Gm47404 | predicted gene, 47404 [Source:MGI Symbol;Acc:MGI:6096334] | yes|up |
| ENSMUSG00000113262 | Gm48551 | predicted gene, 48551 [Source:MGI Symbol;Acc:MGI:6098103] | yes|up |
| ENSMUSG00000034614 | Pik3ip1 | phosphoinositide-3-kinase interacting protein 1 [Source:MGI Symbol;Acc:MGI:1917016] | yes|up |
| ENSMUSG00000078490 | Cfap74 | cilia and flagella associated protein 74 [Source:MGI Symbol;Acc:MGI:1917130] | yes|up |
| ENSMUSG00000078496 | Zfp982 | zinc finger protein 982 [Source:MGI Symbol;Acc:MGI:3701121] | yes|down |
| ENSMUSG00000078495 | Zfp984 | zinc finger protein 984 [Source:MGI Symbol;Acc:MGI:3651978] | yes|down |
| ENSMUSG00000117922 | Gm50397 | predicted gene, 50397 [Source:MGI Symbol;Acc:MGI:6303305] | yes|down |
| ENSMUSG00000109482 | Gm4756 | predicted gene 4756 [Source:MGI Symbol;Acc:MGI:3644906] | yes|up |
| ENSMUSG00000036777 | Anln | anillin, actin binding protein [Source:MGI Symbol;Acc:MGI:1920174] | yes|down |
| ENSMUSG00000021591 | Glrx | glutaredoxin [Source:MGI Symbol;Acc:MGI:2135625] | yes|down |
| ENSMUSG00000031980 | Agt | angiotensinogen (serpin peptidase inhibitor, clade A, member 8) [Source:MGI Symbol;Acc:MGI:87963] | yes|up |
| ENSMUSG00000021596 | Mctp1 | multiple C2 domains, transmembrane 1 [Source:MGI Symbol;Acc:MGI:1926021] | yes|down |
| ENSMUSG00000027855 | Sycp1 | synaptonemal complex protein 1 [Source:MGI Symbol;Acc:MGI:105931] | yes|up |
| ENSMUSG00000041449 | Serpina3h | serine (or cysteine) peptidase inhibitor, clade A, member 3H [Source:MGI Symbol;Acc:MGI:2182839] | yes|up |
| ENSMUSG00000024529 | Lox | lysyl oxidase [Source:MGI Symbol;Acc:MGI:96817] | yes|up |
| ENSMUSG00000005410 | Mcm5 | minichromosome maintenance complex component 5 [Source:MGI Symbol;Acc:MGI:103197] | yes|down |
| ENSMUSG00000086181 | C230034O21Rik | RIKEN cDNA C230034O21 gene [Source:MGI Symbol;Acc:MGI:2441892] | yes|up |
| ENSMUSG00000026748 | Plxdc2 | plexin domain containing 2 [Source:MGI Symbol;Acc:MGI:1914698] | yes|up |
| ENSMUSG00000023330 | Dtwd1 | DTW domain containing 1 [Source:MGI Symbol;Acc:MGI:1916435] | yes|down |
| ENSMUSG00000067219 | Nipal1 | NIPA-like domain containing 1 [Source:MGI Symbol;Acc:MGI:1917951] | yes|down |
| ENSMUSG00000067212 | H2-T23 | histocompatibility 2, T region locus 23 [Source:MGI Symbol;Acc:MGI:95957] | yes|up |
| ENSMUSG00000052397 | Ezr | ezrin [Source:MGI Symbol;Acc:MGI:98931] | yes|down |
| ENSMUSG00000040990 | Sh3kbp1 | SH3-domain kinase binding protein 1 [Source:MGI Symbol;Acc:MGI:1889583] | yes|down |
| ENSMUSG00000056758 | Hmga2 | high mobility group AT-hook 2 [Source:MGI Symbol;Acc:MGI:101761] | yes|down |
| ENSMUSG00000040998 | Npnt | nephronectin [Source:MGI Symbol;Acc:MGI:2148811] | yes|down |
| ENSMUSG00000041351 | Rap1gap | Rap1 GTPase-activating protein [Source:MGI Symbol;Acc:MGI:109338] | yes|down |
| ENSMUSG00000031928 | Mre11a | MRE11A homolog A, double strand break repair nuclease [Source:MGI Symbol;Acc:MGI:1100512] | yes|down |
| ENSMUSG00000031438 | Rnf128 | ring finger protein 128 [Source:MGI Symbol;Acc:MGI:1914139] | yes|up |
| ENSMUSG00000031432 | Prps1 | phosphoribosyl pyrophosphate synthetase 1 [Source:MGI Symbol;Acc:MGI:97775] | yes|down |
| ENSMUSG00000031431 | Tsc22d3 | TSC22 domain family, member 3 [Source:MGI Symbol;Acc:MGI:1196284] | yes|up |
| ENSMUSG00000086233 | Gm11816 | predicted gene 11816 [Source:MGI Symbol;Acc:MGI:3650294] | yes|down |
| ENSMUSG00000038642 | Ctss | cathepsin S [Source:MGI Symbol;Acc:MGI:107341] | yes|up |
| ENSMUSG00000038641 | Akr1d1 | aldo-keto reductase family 1, member D1 [Source:MGI Symbol;Acc:MGI:2384785] | yes|up |
| ENSMUSG00000038644 | Pold1 | polymerase (DNA directed), delta 1, catalytic subunit [Source:MGI Symbol;Acc:MGI:97741] | yes|down |
| ENSMUSG00000030346 | Rad51ap1 | RAD51 associated protein 1 [Source:MGI Symbol;Acc:MGI:1098224] | yes|down |
| ENSMUSG00000104861 | 3110039M20Rik | RIKEN cDNA 3110039M20 gene [Source:MGI Symbol;Acc:MGI:1914543] | yes|down |
| ENSMUSG00000094664 | Rpl35a-ps6 | ribosomal protein L35A, pseudogene 6 [Source:MGI Symbol;Acc:MGI:3704258] | yes|down |
| ENSMUSG00000073411 | H2-D1 | histocompatibility 2, D region locus 1 [Source:MGI Symbol;Acc:MGI:95896] | yes|up |
| ENSMUSG00000092386 | Gm20536 | predicted gene 20536 [Source:MGI Symbol;Acc:MGI:5142001] | yes|up |
| ENSMUSG00000073418 | C4b | complement component 4B (Chido blood group) [Source:MGI Symbol;Acc:MGI:88228] | yes|up |
| ENSMUSG00000038252 | Ncapd2 | non-SMC condensin I complex, subunit D2 [Source:MGI Symbol;Acc:MGI:1915548] | yes|down |
| ENSMUSG00000038253 | Hoxa5 | homeobox A5 [Source:MGI Symbol;Acc:MGI:96177] | yes|up |
| ENSMUSG00000032243 | Itga11 | integrin alpha 11 [Source:MGI Symbol;Acc:MGI:2442114] | yes|up |
| ENSMUSG00000025597 | Klhl4 | kelch-like 4 [Source:MGI Symbol;Acc:MGI:2442829] | yes|up |
| ENSMUSG00000022240 | Ctnnd2 | catenin (cadherin associated protein), delta 2 [Source:MGI Symbol;Acc:MGI:1195966] | yes|down |
| ENSMUSG00000029925 | Tbxas1 | thromboxane A synthase 1, platelet [Source:MGI Symbol;Acc:MGI:98497] | yes|up |
| ENSMUSG00000028744 | Slc66a1 | solute carrier family 66 member 1 [Source:MGI Symbol;Acc:MGI:2384837] | yes|up |
| ENSMUSG00000102425 | Gm26616 | predicted gene, 26616 [Source:MGI Symbol;Acc:MGI:5477110] | yes|up |
| ENSMUSG00000048572 | Tmem252 | transmembrane protein 252 [Source:MGI Symbol;Acc:MGI:3583948] | yes|up |
| ENSMUSG00000052504 | Epha3 | Eph receptor A3 [Source:MGI Symbol;Acc:MGI:99612] | yes|up |
| ENSMUSG00000029436 | Mmp17 | matrix metallopeptidase 17 [Source:MGI Symbol;Acc:MGI:1346076] | yes|up |
| ENSMUSG00000084071 | Gm13378 | predicted gene 13378 [Source:MGI Symbol;Acc:MGI:3650302] | yes|down |
| ENSMUSG00000037991 | Rmi2 | RecQ mediated genome instability 2 [Source:MGI Symbol;Acc:MGI:2685383] | yes|down |
| ENSMUSG00000037995 | Igsf9 | immunoglobulin superfamily, member 9 [Source:MGI Symbol;Acc:MGI:2135283] | yes|down |
| ENSMUSG00000085105 | Gm12758 | predicted gene 12758 [Source:MGI Symbol;Acc:MGI:3702543] | yes|down |
| ENSMUSG00000035842 | Ddx11 | DEAD/H box helicase 11 [Source:MGI Symbol;Acc:MGI:2443590] | yes|down |
| ENSMUSG00000025738 | Fbxl16 | F-box and leucine-rich repeat protein 16 [Source:MGI Symbol;Acc:MGI:2448488] | yes|up |
| ENSMUSG00000032313 | Tmem266 | transmembrane protein 266 [Source:MGI Symbol;Acc:MGI:2142980] | yes|down |
| ENSMUSG00000060314 | Zfp941 | zinc finger protein 941 [Source:MGI Symbol;Acc:MGI:3039601] | yes|down |
| ENSMUSG00000060317 | Acnat2 | acyl-coenzyme A amino acid N-acyltransferase 2 [Source:MGI Symbol;Acc:MGI:2444345] | yes|up |
| ENSMUSG00000114598 | D130062J10Rik | RIKEN cDNA D130062J10 gene [Source:MGI Symbol;Acc:MGI:2441949] | yes|down |
| ENSMUSG00000026414 | Tnnt2 | troponin T2, cardiac [Source:MGI Symbol;Acc:MGI:104597] | yes|up |
| ENSMUSG00000021747 | Cfap20dc | CFAP20 domain containing [Source:MGI Symbol;Acc:MGI:1926154] | yes|down |
| ENSMUSG00000093656 | Gm20628 | predicted gene 20628 [Source:MGI Symbol;Acc:MGI:5313075] | yes|up |
| ENSMUSG00000036169 | Sostdc1 | sclerostin domain containing 1 [Source:MGI Symbol;Acc:MGI:1913292] | yes|down |
| ENSMUSG00000033998 | Kcnk1 | potassium channel, subfamily K, member 1 [Source:MGI Symbol;Acc:MGI:109322] | yes|up |
| ENSMUSG00000038415 | Foxq1 | forkhead box Q1 [Source:MGI Symbol;Acc:MGI:1298228] | yes|down |
| ENSMUSG00000044083 | Efcab8 | EF-hand calcium binding domain 8 [Source:MGI Symbol;Acc:MGI:3644206] | yes|up |
| ENSMUSG00000044080 | S100a1 | S100 calcium binding protein A1 [Source:MGI Symbol;Acc:MGI:1338917] | yes|down |
| ENSMUSG00000038418 | Egr1 | early growth response 1 [Source:MGI Symbol;Acc:MGI:95295] | yes|down |
| ENSMUSG00000037780 | Mbl1 | mannose-binding lectin (protein A) 1 [Source:MGI Symbol;Acc:MGI:96923] | yes|up |
| ENSMUSG00000070392 | Gm20634 | predicted gene 20634 [Source:MGI Symbol;Acc:MGI:5313081] | yes|up |
| ENSMUSG00000063415 | Cyp26b1 | cytochrome P450, family 26, subfamily b, polypeptide 1 [Source:MGI Symbol;Acc:MGI:2176159] | yes|down |
| ENSMUSG00000022809 | Nr1i2 | nuclear receptor subfamily 1, group I, member 2 [Source:MGI Symbol;Acc:MGI:1337040] | yes|up |
| ENSMUSG00000022803 | Popdc2 | popeye domain containing 2 [Source:MGI Symbol;Acc:MGI:1930150] | yes|up |
| ENSMUSG00000054545 | Ugt1a6a | UDP glucuronosyltransferase 1 family, polypeptide A6A [Source:MGI Symbol;Acc:MGI:2137698] | yes|down |
| ENSMUSG00000051736 | Fam229b | family with sequence similarity 229, member B [Source:MGI Symbol;Acc:MGI:1913587] | yes|down |
| ENSMUSG00000048376 | F2r | coagulation factor II (thrombin) receptor [Source:MGI Symbol;Acc:MGI:101802] | yes|up |
| ENSMUSG00000096688 | Mup17 | major urinary protein 17 [Source:MGI Symbol;Acc:MGI:3705217] | yes|up |
| ENSMUSG00000027603 | Ggt7 | gamma-glutamyltransferase 7 [Source:MGI Symbol;Acc:MGI:1913385] | yes|up |
| ENSMUSG00000027353 | Mcm8 | minichromosome maintenance 8 homologous recombination repair factor [Source:MGI Symbol;Acc:MGI:1913884] | yes|down |
| ENSMUSG00000027359 | Slc27a2 | solute carrier family 27 (fatty acid transporter), member 2 [Source:MGI Symbol;Acc:MGI:1347099] | yes|up |
| ENSMUSG00000033730 | Egr3 | early growth response 3 [Source:MGI Symbol;Acc:MGI:1306780] | yes|down |
| ENSMUSG00000031594 | Fgl1 | fibrinogen-like protein 1 [Source:MGI Symbol;Acc:MGI:102795] | yes|up |
| ENSMUSG00000031595 | Pdgfrl | platelet-derived growth factor receptor-like [Source:MGI Symbol;Acc:MGI:1916047] | yes|up |
| ENSMUSG00000031596 | Slc7a2 | solute carrier family 7 (cationic amino acid transporter, y+ system), member 2 [Source:MGI Symbol;Acc:MGI:99828] | yes|up |
| ENSMUSG00000097084 | Foxl1 | forkhead box L1 [Source:MGI Symbol;Acc:MGI:1347469] | yes|down |
| ENSMUSG00000030423 | Pop4 | processing of precursor 4, ribonuclease P/MRP family, (S. cerevisiae) [Source:MGI Symbol;Acc:MGI:1913411] | yes|up |
| ENSMUSG00000097081 | Gm10425 | predicted gene 10425 [Source:MGI Symbol;Acc:MGI:3642471] | yes|down |
| ENSMUSG00000037348 | Paqr7 | progestin and adipoQ receptor family member VII [Source:MGI Symbol;Acc:MGI:1919154] | yes|up |
| ENSMUSG00000040502 | Marchf9 | membrane associated ring-CH-type finger 9 [Source:MGI Symbol;Acc:MGI:2446144] | yes|up |
| ENSMUSG00000074796 | Slc4a11 | solute carrier family 4, sodium bicarbonate transporter-like, member 11 [Source:MGI Symbol;Acc:MGI:2138987] | yes|down |
| ENSMUSG00000096971 | 4930556M19Rik | RIKEN cDNA 4930556M19 gene [Source:MGI Symbol;Acc:MGI:1922509] | yes|down |
| ENSMUSG00000027977 | Ndst3 | N-deacetylase/N-sulfotransferase (heparan glucosaminyl) 3 [Source:MGI Symbol;Acc:MGI:1932544] | yes|down |
| ENSMUSG00000023070 | Rgn | regucalcin [Source:MGI Symbol;Acc:MGI:108024] | yes|up |
| ENSMUSG00000023072 | Cep89 | centrosomal protein 89 [Source:MGI Symbol;Acc:MGI:1919390] | yes|down |
| ENSMUSG00000040767 | Snrnp25 | small nuclear ribonucleoprotein 25 (U11/U12) [Source:MGI Symbol;Acc:MGI:1925622] | yes|down |
| ENSMUSG00000104498 | Gm37110 | predicted gene, 37110 [Source:MGI Symbol;Acc:MGI:5610338] | yes|down |
| ENSMUSG00000056144 | Trim34a | tripartite motif-containing 34A [Source:MGI Symbol;Acc:MGI:2137359] | yes|up |
| ENSMUSG00000056148 | Rdh9 | retinol dehydrogenase 9 [Source:MGI Symbol;Acc:MGI:2143528] | yes|up |
| ENSMUSG00000029605 | Oas1b | 2'-5' oligoadenylate synthetase 1B [Source:MGI Symbol;Acc:MGI:97430] | yes|up |
| ENSMUSG00000025925 | Terf1 | telomeric repeat binding factor 1 [Source:MGI Symbol;Acc:MGI:109634] | yes|down |
| ENSMUSG00000025927 | Tfap2b | transcription factor AP-2 beta [Source:MGI Symbol;Acc:MGI:104672] | yes|down |
| ENSMUSG00000061535 | C1qtnf7 | C1q and tumor necrosis factor related protein 7 [Source:MGI Symbol;Acc:MGI:1925911] | yes|up |
| ENSMUSG00000061533 | Cep128 | centrosomal protein 128 [Source:MGI Symbol;Acc:MGI:1922466] | yes|down |
| ENSMUSG00000001517 | Foxm1 | forkhead box M1 [Source:MGI Symbol;Acc:MGI:1347487] | yes|down |
| ENSMUSG00000047230 | Cldn2 | claudin 2 [Source:MGI Symbol;Acc:MGI:1276110] | yes|up |
| ENSMUSG00000036908 | Unc93b1 | unc-93 homolog B1, TLR signaling regulator [Source:MGI Symbol;Acc:MGI:1859307] | yes|up |
| ENSMUSG00000024386 | Proc | protein C [Source:MGI Symbol;Acc:MGI:97771] | yes|up |
| ENSMUSG00000097960 | A330074K22Rik | RIKEN cDNA A330074K22 gene [Source:MGI Symbol;Acc:MGI:3045392] | yes|down |
| ENSMUSG00000036904 | Fzd8 | frizzled class receptor 8 [Source:MGI Symbol;Acc:MGI:108460] | yes|up |
| ENSMUSG00000036905 | C1qb | complement component 1, q subcomponent, beta polypeptide [Source:MGI Symbol;Acc:MGI:88224] | yes|up |
| ENSMUSG00000036902 | Neto2 | neuropilin (NRP) and tolloid (TLL)-like 2 [Source:MGI Symbol;Acc:MGI:1921763] | yes|down |
| ENSMUSG00000034853 | Acot11 | acyl-CoA thioesterase 11 [Source:MGI Symbol;Acc:MGI:1913736] | yes|up |
| ENSMUSG00000034855 | Cxcl10 | chemokine (C-X-C motif) ligand 10 [Source:MGI Symbol;Acc:MGI:1352450] | yes|up |
| ENSMUSG00000121484 | Serpina3h | serine (or cysteine) peptidase inhibitor, clade A, member 3H [Source:NCBI gene (formerly Entrezgene);Acc:546546] | yes|up |
| ENSMUSG00000043531 | Sorcs1 | sortilin-related VPS10 domain containing receptor 1 [Source:MGI Symbol;Acc:MGI:1929666] | yes|up |
| ENSMUSG00000049907 | Rasl11b | RAS-like, family 11, member B [Source:MGI Symbol;Acc:MGI:1916189] | yes|up |
| ENSMUSG00000016349 | Eef1a2 | eukaryotic translation elongation factor 1 alpha 2 [Source:MGI Symbol;Acc:MGI:1096317] | yes|down |
| ENSMUSG00000121486 | Zfp783 | zinc finger protein 783 [Source:NCBI gene (formerly Entrezgene);Acc:232785] | yes|up |
| ENSMUSG00000035455 | Fignl1 | fidgetin-like 1 [Source:MGI Symbol;Acc:MGI:1890648] | yes|down |
| ENSMUSG00000035104 | Eva1a | eva-1 homolog A (C. elegans) [Source:MGI Symbol;Acc:MGI:2385247] | yes|up |
| ENSMUSG00000035451 | Foxa1 | forkhead box A1 [Source:MGI Symbol;Acc:MGI:1347472] | yes|up |
| ENSMUSG00000042489 | Clspn | claspin [Source:MGI Symbol;Acc:MGI:2445153] | yes|down |
| ENSMUSG00000035459 | Stab2 | stabilin 2 [Source:MGI Symbol;Acc:MGI:2178743] | yes|up |
| ENSMUSG00000075520 | Malrd1 | MAM and LDL receptor class A domain containing 1 [Source:MGI Symbol;Acc:MGI:1928271] | yes|down |
| ENSMUSG00000103034 | Gm8797 | predicted pseudogene 8797 [Source:MGI Symbol;Acc:MGI:3643769] | yes|down |
| ENSMUSG00000064317 | Gm10146 | predicted gene 10146 [Source:MGI Symbol;Acc:MGI:3704367] | yes|down |
| ENSMUSG00000069805 | Fbp1 | fructose bisphosphatase 1 [Source:MGI Symbol;Acc:MGI:95492] | yes|up |
| ENSMUSG00000028268 | Gbp3 | guanylate binding protein 3 [Source:MGI Symbol;Acc:MGI:1926263] | yes|up |
| ENSMUSG00000100944 | Gm28900 | predicted gene 28900 [Source:MGI Symbol;Acc:MGI:5579606] | yes|up |
| ENSMUSG00000066443 | Gm10163 | predicted pseudogene 10163 [Source:MGI Symbol;Acc:MGI:3704341] | yes|down |
| ENSMUSG00000089960 | Ugt1a1 | UDP glucuronosyltransferase 1 family, polypeptide A1 [Source:MGI Symbol;Acc:MGI:98898] | yes|up |
| ENSMUSG00000019929 | Dcn | decorin [Source:MGI Symbol;Acc:MGI:94872] | yes|up |
| ENSMUSG00000045273 | Cenph | centromere protein H [Source:MGI Symbol;Acc:MGI:1349448] | yes|down |
| ENSMUSG00000032128 | Robo3 | roundabout guidance receptor 3 [Source:MGI Symbol;Acc:MGI:1343102] | yes|down |
| ENSMUSG00000032109 | Nlrx1 | NLR family member X1 [Source:MGI Symbol;Acc:MGI:2429611] | yes|down |
| ENSMUSG00000032452 | Clstn2 | calsyntenin 2 [Source:MGI Symbol;Acc:MGI:1929897] | yes|up |
| ENSMUSG00000001249 | Hpn | hepsin [Source:MGI Symbol;Acc:MGI:1196620] | yes|up |
| ENSMUSG00000001247 | Lsr | lipolysis stimulated lipoprotein receptor [Source:MGI Symbol;Acc:MGI:1927471] | yes|up |
| ENSMUSG00000001240 | Ramp2 | receptor (calcitonin) activity modifying protein 2 [Source:MGI Symbol;Acc:MGI:1859650] | yes|up |
| ENSMUSG00000082087 | Gm12138 | predicted gene 12138 [Source:MGI Symbol;Acc:MGI:3651749] | yes|down |
| ENSMUSG00000019256 | Ahr | aryl-hydrocarbon receptor [Source:MGI Symbol;Acc:MGI:105043] | yes|up |
| ENSMUSG00000096768 | Gm47283 | predicted gene, 47283 [Source:MGI Symbol;Acc:MGI:6096131] | yes|down |
| ENSMUSG00000117901 | Gm54798 | predicted gene, 54798 [Source:MGI Symbol;Acc:MGI:6846073] | yes|down |
| ENSMUSG00000033502 | Cdc14a | CDC14 cell division cycle 14A [Source:MGI Symbol;Acc:MGI:2442676] | yes|down |
| ENSMUSG00000043999 | Gpr75 | G protein-coupled receptor 75 [Source:MGI Symbol;Acc:MGI:2441843] | yes|down |
| ENSMUSG00000108350 | Gm44950 | predicted gene 44950 [Source:MGI Symbol;Acc:MGI:5753526] | yes|down |
| ENSMUSG00000024727 | Trpm6 | transient receptor potential cation channel, subfamily M, member 6 [Source:MGI Symbol;Acc:MGI:2675603] | yes|down |
| ENSMUSG00000062785 | Kcnc3 | potassium voltage gated channel, Shaw-related subfamily, member 3 [Source:MGI Symbol;Acc:MGI:96669] | yes|up |
| ENSMUSG00000026768 | Itga8 | integrin alpha 8 [Source:MGI Symbol;Acc:MGI:109442] | yes|up |
| ENSMUSG00000019782 | Rwdd1 | RWD domain containing 1 [Source:MGI Symbol;Acc:MGI:1913771] | yes|down |
| ENSMUSG00000024548 | Setbp1 | SET binding protein 1 [Source:MGI Symbol;Acc:MGI:1933199] | yes|up |
| ENSMUSG00000091476 | Catspere2 | cation channel sperm associated auxiliary subunit epsilon 2 [Source:MGI Symbol;Acc:MGI:5589632] | yes|up |
| ENSMUSG00000024899 | Papss2 | 3'-phosphoadenosine 5'-phosphosulfate synthase 2 [Source:MGI Symbol;Acc:MGI:1330223] | yes|up |
| ENSMUSG00000026494 | Kif26b | kinesin family member 26B [Source:MGI Symbol;Acc:MGI:2447076] | yes|up |
| ENSMUSG00000026496 | Parp1 | poly (ADP-ribose) polymerase family, member 1 [Source:MGI Symbol;Acc:MGI:1340806] | yes|down |
| ENSMUSG00000097415 | AU020206 | expressed sequence AU020206 [Source:MGI Symbol;Acc:MGI:2142134] | yes|down |
| ENSMUSG00000041372 | B4galnt3 | beta-1,4-N-acetyl-galactosaminyl transferase 3 [Source:MGI Symbol;Acc:MGI:3041155] | yes|down |
| ENSMUSG00000031903 | Pla2g15 | phospholipase A2, group XV [Source:MGI Symbol;Acc:MGI:2178076] | yes|up |
| ENSMUSG00000048489 | Depp1 | DEPP1 autophagy regulator [Source:MGI Symbol;Acc:MGI:1918730] | yes|up |
| ENSMUSG00000031906 | Smpd3 | sphingomyelin phosphodiesterase 3, neutral [Source:MGI Symbol;Acc:MGI:1927578] | yes|up |
| ENSMUSG00000053040 | Aph1c | aph1 homolog C, gamma secretase subunit [Source:MGI Symbol;Acc:MGI:1915568] | yes|down |
| ENSMUSG00000030364 | Clec2h | C-type lectin domain family 2, member h [Source:MGI Symbol;Acc:MGI:2136934] | yes|up |
| ENSMUSG00000115354 | Gm49083 | predicted gene, 49083 [Source:MGI Symbol;Acc:MGI:6118469] | yes|up |
| ENSMUSG00000121500 |  | novel transcript | yes|up |
| ENSMUSG00000121506 |  | novel transcript | yes|up |
| ENSMUSG00000027018 | Hat1 | histone aminotransferase 1 [Source:MGI Symbol;Acc:MGI:96013] | yes|down |
| ENSMUSG00000039057 | Myo16 | myosin XVI [Source:MGI Symbol;Acc:MGI:2685951] | yes|up |
| ENSMUSG00000004473 | Clec11a | C-type lectin domain family 11, member a [Source:MGI Symbol;Acc:MGI:1298219] | yes|up |
| ENSMUSG00000048087 | Ahcyl | adenosylhomocysteinase like [Source:MGI Symbol;Acc:MGI:3643647] | yes|down |
| ENSMUSG00000029546 | Uncx | UNC homeobox [Source:MGI Symbol;Acc:MGI:108013] | yes|up |
| ENSMUSG00000068874 | Selenbp1 | selenium binding protein 1 [Source:MGI Symbol;Acc:MGI:96825] | yes|up |
| ENSMUSG00000031012 | Cask | calcium/calmodulin-dependent serine protein kinase (MAGUK family) [Source:MGI Symbol;Acc:MGI:1309489] | yes|up |
| ENSMUSG00000057604 | Lmcd1 | LIM and cysteine-rich domains 1 [Source:MGI Symbol;Acc:MGI:1353635] | yes|up |
| ENSMUSG00000034192 | Lsm3 | LSM3 homolog, U6 small nuclear RNA and mRNA degradation associated [Source:MGI Symbol;Acc:MGI:1914928] | yes|down |
| ENSMUSG00000029414 | Kntc1 | kinetochore associated 1 [Source:MGI Symbol;Acc:MGI:2673709] | yes|down |
| ENSMUSG00000029413 | Naaa | N-acylethanolamine acid amidase [Source:MGI Symbol;Acc:MGI:1914361] | yes|down |
| ENSMUSG00000028010 | Gar1 | GAR1 ribonucleoprotein [Source:MGI Symbol;Acc:MGI:1930948] | yes|down |
| ENSMUSG00000035868 | Zfp983 | zinc finger protein 983 [Source:MGI Symbol;Acc:MGI:1920479] | yes|up |
| ENSMUSG00000070348 | Ccnd1 | cyclin D1 [Source:MGI Symbol;Acc:MGI:88313] | yes|down |
| ENSMUSG00000021620 | Acot12 | acyl-CoA thioesterase 12 [Source:MGI Symbol;Acc:MGI:1921406] | yes|up |
| ENSMUSG00000074024 | 4632427E13Rik | RIKEN cDNA 4632427E13 gene [Source:MGI Symbol;Acc:MGI:1915436] | yes|up |
| ENSMUSG00000046463 | 5930403N24Rik | RIKEN cDNA 5930403N24 gene [Source:MGI Symbol;Acc:MGI:2444171] | yes|up |
| ENSMUSG00000032596 | Uba7 | ubiquitin-like modifier activating enzyme 7 [Source:MGI Symbol;Acc:MGI:1349462] | yes|up |
| ENSMUSG00000032591 | Mst1 | macrophage stimulating 1 (hepatocyte growth factor-like) [Source:MGI Symbol;Acc:MGI:96080] | yes|up |
| ENSMUSG00000042549 | Map2k3os | mitogen-activated protein kinase kinase 3, opposite strand [Source:MGI Symbol;Acc:MGI:1344334] | yes|down |
| ENSMUSG00000063632 | Sox11 | SRY (sex determining region Y)-box 11 [Source:MGI Symbol;Acc:MGI:98359] | yes|up |
| ENSMUSG00000024391 | Apom | apolipoprotein M [Source:MGI Symbol;Acc:MGI:1930124] | yes|up |
| ENSMUSG00000084883 | Ccdc85c | coiled-coil domain containing 85C [Source:MGI Symbol;Acc:MGI:3644008] | yes|down |
| ENSMUSG00000024395 | Lims2 | LIM and senescent cell antigen like domains 2 [Source:MGI Symbol;Acc:MGI:2385067] | yes|up |
| ENSMUSG00000086881 | Gm13594 | predicted gene 13594 [Source:MGI Symbol;Acc:MGI:3702953] | yes|up |
| ENSMUSG00000052920 | Prkg1 | protein kinase, cGMP-dependent, type I [Source:MGI Symbol;Acc:MGI:108174] | yes|up |
| ENSMUSG00000060636 | Rpl35a | ribosomal protein L35A [Source:MGI Symbol;Acc:MGI:1928894] | yes|down |
| ENSMUSG00000032359 | Ctsh | cathepsin H [Source:MGI Symbol;Acc:MGI:107285] | yes|up |
| ENSMUSG00000002985 | Apoe | apolipoprotein E [Source:MGI Symbol;Acc:MGI:88057] | yes|up |
| ENSMUSG00000022821 | Hgd | homogentisate 1, 2-dioxygenase [Source:MGI Symbol;Acc:MGI:96078] | yes|up |
| ENSMUSG00000039814 | Xkr5 | X-linked Kx blood group related 5 [Source:MGI Symbol;Acc:MGI:2442327] | yes|down |
| ENSMUSG00000117042 | 2700054A10Rik | RIKEN cDNA 2700054A10 gene [Source:MGI Symbol;Acc:MGI:1919828] | yes|down |
| ENSMUSG00000075297 | H60b | histocompatibility 60b [Source:MGI Symbol;Acc:MGI:3649078] | yes|down |
| ENSMUSG00000020098 | Pcbd1 | pterin 4 alpha carbinolamine dehydratase/dimerization cofactor of hepatocyte nuclear factor 1 alpha (TCF1) 1 [Source:MGI Symbol;Acc:MGI:94873] | yes|up |
| ENSMUSG00000058385 | H2bc8 | H2B clustered histone 8 [Source:MGI Symbol;Acc:MGI:2448386] | yes|up |
| ENSMUSG00000119996 |  | novel transcript | yes|up |
| ENSMUSG00000119993 |  | novel transcript | yes|down |
| ENSMUSG00000058388 | Phtf1 | putative homeodomain transcription factor 1 [Source:MGI Symbol;Acc:MGI:1332671] | yes|down |
| ENSMUSG00000016918 | Sulf1 | sulfatase 1 [Source:MGI Symbol;Acc:MGI:2138563] | yes|up |
| ENSMUSG00000030621 | Me3 | malic enzyme 3, NADP(+)-dependent, mitochondrial [Source:MGI Symbol;Acc:MGI:1916679] | yes|up |
| ENSMUSG00000001763 | Tspan33 | tetraspanin 33 [Source:MGI Symbol;Acc:MGI:1919012] | yes|up |
| ENSMUSG00000027379 | Bub1 | BUB1, mitotic checkpoint serine/threonine kinase [Source:MGI Symbol;Acc:MGI:1100510] | yes|down |
| ENSMUSG00000027378 | Nphp1 | nephronophthisis 1 (juvenile) homolog (human) [Source:MGI Symbol;Acc:MGI:1858233] | yes|down |
| ENSMUSG00000020897 | Aurkb | aurora kinase B [Source:MGI Symbol;Acc:MGI:107168] | yes|down |
| ENSMUSG00000020895 | Tmem107 | transmembrane protein 107 [Source:MGI Symbol;Acc:MGI:1914160] | yes|down |
| ENSMUSG00000004113 | Cacna1b | calcium channel, voltage-dependent, N type, alpha 1B subunit [Source:MGI Symbol;Acc:MGI:88296] | yes|up |
| ENSMUSG00000020892 | Aloxe3 | arachidonate lipoxygenase 3 [Source:MGI Symbol;Acc:MGI:1345140] | yes|up |
| ENSMUSG00000051716 | Apon | apolipoprotein N [Source:MGI Symbol;Acc:MGI:88931] | yes|up |
| ENSMUSG00000039748 | Exo1 | exonuclease 1 [Source:MGI Symbol;Acc:MGI:1349427] | yes|down |
| ENSMUSG00000033717 | Adra2a | adrenergic receptor, alpha 2a [Source:MGI Symbol;Acc:MGI:87934] | yes|up |
| ENSMUSG00000058656 | Samd12 | sterile alpha motif domain containing 12 [Source:MGI Symbol;Acc:MGI:2444518] | yes|down |
| ENSMUSG00000024134 | Six2 | sine oculis-related homeobox 2 [Source:MGI Symbol;Acc:MGI:102778] | yes|down |
| ENSMUSG00000039747 | Orai2 | ORAI calcium release-activated calcium modulator 2 [Source:MGI Symbol;Acc:MGI:2443195] | yes|down |
| ENSMUSG00000046719 | Nxph3 | neurexophilin 3 [Source:MGI Symbol;Acc:MGI:1336188] | yes|down |
| ENSMUSG00000040564 | Apoc1 | apolipoprotein C-I [Source:MGI Symbol;Acc:MGI:88053] | yes|up |
| ENSMUSG00000104121 | Gm37485 | predicted gene, 37485 [Source:MGI Symbol;Acc:MGI:5610713] | yes|down |
| ENSMUSG00000040569 | Slc26a7 | solute carrier family 26, member 7 [Source:MGI Symbol;Acc:MGI:2384791] | yes|up |
| ENSMUSG00000027959 | Sass6 | SAS-6 centriolar assembly protein [Source:MGI Symbol;Acc:MGI:1920026] | yes|down |
| ENSMUSG00000073599 | Ecscr | endothelial cell surface expressed chemotaxis and apoptosis regulator [Source:MGI Symbol;Acc:MGI:1915795] | yes|up |
| ENSMUSG00000030966 | Trim21 | tripartite motif-containing 21 [Source:MGI Symbol;Acc:MGI:106657] | yes|up |
| ENSMUSG00000027951 | Adar | adenosine deaminase, RNA-specific [Source:MGI Symbol;Acc:MGI:1889575] | yes|up |
| ENSMUSG00000048763 | Hoxb3 | homeobox B3 [Source:MGI Symbol;Acc:MGI:96184] | yes|up |
| ENSMUSG00000027955 | Gask1b | golgi associated kinase 1B [Source:MGI Symbol;Acc:MGI:1915909] | yes|up |
| ENSMUSG00000003469 | Phyhip | phytanoyl-CoA hydroxylase interacting protein [Source:MGI Symbol;Acc:MGI:1860417] | yes|up |
| ENSMUSG00000023019 | Gpd1 | glycerol-3-phosphate dehydrogenase 1 (soluble) [Source:MGI Symbol;Acc:MGI:95679] | yes|up |
| ENSMUSG00000035172 | Plekhh3 | pleckstrin homology domain containing, family H (with MyTH4 domain) member 3 [Source:MGI Symbol;Acc:MGI:2384950] | yes|up |
| ENSMUSG00000034295 | Fhod3 | formin homology 2 domain containing 3 [Source:MGI Symbol;Acc:MGI:1925847] | yes|up |
| ENSMUSG00000025949 | Pikfyve | phosphoinositide kinase, FYVE type zinc finger containing [Source:MGI Symbol;Acc:MGI:1335106] | yes|up |
| ENSMUSG00000047787 | Flrt1 | fibronectin leucine rich transmembrane protein 1 [Source:MGI Symbol;Acc:MGI:3026647] | yes|down |
| ENSMUSG00000012443 | Kif11 | kinesin family member 11 [Source:MGI Symbol;Acc:MGI:1098231] | yes|down |
| ENSMUSG00000069310 | H3c3 | H3 clustered histone 3 [Source:MGI Symbol;Acc:MGI:2448320] | yes|up |
| ENSMUSG00000032372 | Plscr2 | phospholipid scramblase 2 [Source:MGI Symbol;Acc:MGI:1270860] | yes|up |
| ENSMUSG00000097944 | A130014A01Rik | RIKEN cDNA A130014A01 gene [Source:MGI Symbol;Acc:MGI:3028043] | yes|up |
| ENSMUSG00000045193 | Cirbp | cold inducible RNA binding protein [Source:MGI Symbol;Acc:MGI:893588] | yes|down |
| ENSMUSG00000044976 | Wdr72 | WD repeat domain 72 [Source:MGI Symbol;Acc:MGI:3583957] | yes|up |
| ENSMUSG00000047250 | Ptgs1 | prostaglandin-endoperoxide synthase 1 [Source:MGI Symbol;Acc:MGI:97797] | yes|down |
| ENSMUSG00000026272 | Agxt | alanine-glyoxylate aminotransferase [Source:MGI Symbol;Acc:MGI:1329033] | yes|up |
| ENSMUSG00000020701 | Tmem132e | transmembrane protein 132E [Source:MGI Symbol;Acc:MGI:2685490] | yes|up |
| ENSMUSG00000043557 | Mdga1 | MAM domain containing glycosylphosphatidylinositol anchor 1 [Source:MGI Symbol;Acc:MGI:1922012] | yes|up |
| ENSMUSG00000046070 | Igfals | insulin-like growth factor binding protein, acid labile subunit [Source:MGI Symbol;Acc:MGI:107973] | yes|up |
| ENSMUSG00000035472 | Slc25a21 | solute carrier family 25 (mitochondrial oxodicarboxylate carrier), member 21 [Source:MGI Symbol;Acc:MGI:2445059] | yes|down |
| ENSMUSG00000072875 | Gpr27 | G protein-coupled receptor 27 [Source:MGI Symbol;Acc:MGI:1202299] | yes|down |
| ENSMUSG00000008734 | Gprc5b | G protein-coupled receptor, family C, group 5, member B [Source:MGI Symbol;Acc:MGI:1927596] | yes|down |
| ENSMUSG00000064373 | Selenop | selenoprotein P [Source:MGI Symbol;Acc:MGI:894288] | yes|up |
| ENSMUSG00000068196 | Col8a1 | collagen, type VIII, alpha 1 [Source:MGI Symbol;Acc:MGI:88463] | yes|up |
| ENSMUSG00000089945 | Pakap | paralemmin A kinase anchor protein [Source:MGI Symbol;Acc:MGI:5141924] | yes|up |
| ENSMUSG00000054717 | Hmgb2 | high mobility group box 2 [Source:MGI Symbol;Acc:MGI:96157] | yes|down |
| ENSMUSG00000087639 | Gm15512 | predicted gene 15512 [Source:MGI Symbol;Acc:MGI:3782960] | yes|down |
| ENSMUSG00000045257 | Morn2 | MORN repeat containing 2 [Source:MGI Symbol;Acc:MGI:2674071] | yes|down |
| ENSMUSG00000064193 | Gm4735 | predicted gene 4735 [Source:MGI Symbol;Acc:MGI:3645521] | yes|down |
| ENSMUSG00000110981 | Gm35940 | predicted gene, 35940 [Source:MGI Symbol;Acc:MGI:5595099] | yes|down |
| ENSMUSG00000032125 | Robo4 | roundabout guidance receptor 4 [Source:MGI Symbol;Acc:MGI:1921394] | yes|up |
| ENSMUSG00000044783 | Hjurp | Holliday junction recognition protein [Source:MGI Symbol;Acc:MGI:2685821] | yes|down |
| ENSMUSG00000073834 | Mup11 | major urinary protein 11 [Source:MGI Symbol;Acc:MGI:3709617] | yes|up |
| ENSMUSG00000093753 | Gm28577 | predicted gene 28577 [Source:MGI Symbol;Acc:MGI:5579283] | yes|up |
| ENSMUSG00000079501 | Gm5138 | predicted gene 5138 [Source:MGI Symbol;Acc:MGI:3779464] | yes|up |
| ENSMUSG00000100213 | Gm28151 | predicted gene 28151 [Source:MGI Symbol;Acc:MGI:5578857] | yes|up |
| ENSMUSG00000101298 | Gm28308 | predicted gene 28308 [Source:MGI Symbol;Acc:MGI:5579014] | yes|up |
| ENSMUSG00000041577 | Prelp | proline arginine-rich end leucine-rich repeat [Source:MGI Symbol;Acc:MGI:2151110] | yes|up |
| ENSMUSG00000010461 | Eya4 | EYA transcriptional coactivator and phosphatase 4 [Source:MGI Symbol;Acc:MGI:1337104] | yes|up |
| ENSMUSG00000038605 | Samd10 | sterile alpha motif domain containing 10 [Source:MGI Symbol;Acc:MGI:2443872] | yes|down |
| ENSMUSG00000097129 | 4930507D05Rik | RIKEN cDNA 4930507D05 gene [Source:MGI Symbol;Acc:MGI:1921956] | yes|up |
| ENSMUSG00000038602 | Slc35f1 | solute carrier family 35, member F1 [Source:MGI Symbol;Acc:MGI:2139810] | yes|down |
| ENSMUSG00000038608 | Dock10 | dedicator of cytokinesis 10 [Source:MGI Symbol;Acc:MGI:2146320] | yes|up |
| ENSMUSG00000054976 | Nyap2 | neuronal tyrosine-phophorylated phosphoinositide 3-kinase adaptor 2 [Source:MGI Symbol;Acc:MGI:2443135] | yes|up |
| ENSMUSG00000031965 | Tbx20 | T-box 20 [Source:MGI Symbol;Acc:MGI:1888496] | yes|down |
| ENSMUSG00000018906 | P4ha2 | procollagen-proline, 2-oxoglutarate 4-dioxygenase (proline 4-hydroxylase), alpha II polypeptide [Source:MGI Symbol;Acc:MGI:894286] | yes|up |
| ENSMUSG00000000392 | Fap | fibroblast activation protein [Source:MGI Symbol;Acc:MGI:109608] | yes|up |
| ENSMUSG00000030382 | Slc27a5 | solute carrier family 27 (fatty acid transporter), member 5 [Source:MGI Symbol;Acc:MGI:1347100] | yes|up |
| ENSMUSG00000083899 | Gm12346 | predicted gene 12346 [Source:MGI Symbol;Acc:MGI:3649810] | yes|down |
| ENSMUSG00000022021 | Diaph3 | diaphanous related formin 3 [Source:MGI Symbol;Acc:MGI:1927222] | yes|down |
| ENSMUSG00000031388 | Naa10 | N(alpha)-acetyltransferase 10, NatA catalytic subunit [Source:MGI Symbol;Acc:MGI:1915255] | yes|down |
| ENSMUSG00000094806 | Cyp2d10 | cytochrome P450, family 2, subfamily d, polypeptide 10 [Source:MGI Symbol;Acc:MGI:88602] | yes|up |
| ENSMUSG00000040046 | Tph1 | tryptophan hydroxylase 1 [Source:MGI Symbol;Acc:MGI:98796] | yes|up |
| ENSMUSG00000011305 | Plin5 | perilipin 5 [Source:MGI Symbol;Acc:MGI:1914218] | yes|up |
| ENSMUSG00000068854 | H2bc21 | H2B clustered histone 21 [Source:MGI Symbol;Acc:MGI:2448415] | yes|down |
| ENSMUSG00000025889 | Snca | synuclein, alpha [Source:MGI Symbol;Acc:MGI:1277151] | yes|up |
| ENSMUSG00000098789 | Jmjd7 | jumonji domain containing 7 [Source:MGI Symbol;Acc:MGI:3845785] | yes|down |
| ENSMUSG00000028709 | Mob3c | MOB kinase activator 3C [Source:MGI Symbol;Acc:MGI:2140623] | yes|up |
| ENSMUSG00000028873 | Cdca8 | cell division cycle associated 8 [Source:MGI Symbol;Acc:MGI:1196274] | yes|down |
| ENSMUSG00000028702 | Rad54l | RAD54 like (S. cerevisiae) [Source:MGI Symbol;Acc:MGI:894697] | yes|down |
| ENSMUSG00000043789 | Vwce | von Willebrand factor C and EGF domains [Source:MGI Symbol;Acc:MGI:1919018] | yes|up |
| ENSMUSG00000021391 | Cenpp | centromere protein P [Source:MGI Symbol;Acc:MGI:1913586] | yes|down |
| ENSMUSG00000039167 | Adgrl4 | adhesion G protein-coupled receptor L4 [Source:MGI Symbol;Acc:MGI:2655562] | yes|up |
| ENSMUSG00000029470 | P2rx4 | purinergic receptor P2X, ligand-gated ion channel 4 [Source:MGI Symbol;Acc:MGI:1338859] | yes|up |
| ENSMUSG00000120594 |  | novel transcript | yes|up |
| ENSMUSG00000069456 | Rdh16 | retinol dehydrogenase 16 [Source:MGI Symbol;Acc:MGI:1201375] | yes|up |
| ENSMUSG00000025776 | Crispld1 | cysteine-rich secretory protein LCCL domain containing 1 [Source:MGI Symbol;Acc:MGI:1934666] | yes|up |
| ENSMUSG00000025083 | Afap1l2 | actin filament associated protein 1-like 2 [Source:MGI Symbol;Acc:MGI:2147658] | yes|up |
| ENSMUSG00000034463 | Scara3 | scavenger receptor class A, member 3 [Source:MGI Symbol;Acc:MGI:2444418] | yes|down |
| ENSMUSG00000025085 | Ablim1 | actin-binding LIM protein 1 [Source:MGI Symbol;Acc:MGI:1194500] | yes|up |
| ENSMUSG00000034460 | Six4 | sine oculis-related homeobox 4 [Source:MGI Symbol;Acc:MGI:106034] | yes|up |
| ENSMUSG00000021708 | Rasgrf2 | RAS protein-specific guanine nucleotide-releasing factor 2 [Source:MGI Symbol;Acc:MGI:109137] | yes|up |
| ENSMUSG00000046447 | Camk2n1 | calcium/calmodulin-dependent protein kinase II inhibitor 1 [Source:MGI Symbol;Acc:MGI:1913509] | yes|up |
| ENSMUSG00000046442 | Ppm1e | protein phosphatase 1E (PP2C domain containing) [Source:MGI Symbol;Acc:MGI:2444096] | yes|up |
| ENSMUSG00000021702 | Thbs4 | thrombospondin 4 [Source:MGI Symbol;Acc:MGI:1101779] | yes|up |
| ENSMUSG00000021707 | Dhfr | dihydrofolate reductase [Source:MGI Symbol;Acc:MGI:94890] | yes|down |
| ENSMUSG00000038388 | Pals2 | protein associated with LIN7 2, MAGUK family member [Source:MGI Symbol;Acc:MGI:1927340] | yes|down |
| ENSMUSG00000045062 | Pcdhb7 | protocadherin beta 7 [Source:MGI Symbol;Acc:MGI:2136741] | yes|up |
| ENSMUSG00000076846 | Trav13-2 | T cell receptor alpha variable 13-2 [Source:MGI Symbol;Acc:MGI:3651611] | yes|down |
| ENSMUSG00000086714 | 0610009E02Rik | RIKEN cDNA 0610009E02 gene [Source:MGI Symbol;Acc:MGI:3698435] | yes|up |
| ENSMUSG00000078597 | Cyp4a12b | cytochrome P450, family 4, subfamily a, polypeptide 12B [Source:MGI Symbol;Acc:MGI:3611747] | yes|up |
| ENSMUSG00000075312 | Gm13597 | predicted gene 13597 [Source:MGI Symbol;Acc:MGI:3651139] | yes|down |
| ENSMUSG00000033952 | Aspm | abnormal spindle microtubule assembly [Source:MGI Symbol;Acc:MGI:1334448] | yes|down |
| ENSMUSG00000006398 | Cdc20 | cell division cycle 20 [Source:MGI Symbol;Acc:MGI:1859866] | yes|down |
| ENSMUSG00000027111 | Itga6 | integrin alpha 6 [Source:MGI Symbol;Acc:MGI:96605] | yes|down |
| ENSMUSG00000075273 | Ttc30b | tetratricopeptide repeat domain 30B [Source:MGI Symbol;Acc:MGI:1919671] | yes|down |
| ENSMUSG00000063626 | Unc5d | unc-5 netrin receptor D [Source:MGI Symbol;Acc:MGI:2389364] | yes|up |
| ENSMUSG00000036381 | P2ry14 | purinergic receptor P2Y, G-protein coupled, 14 [Source:MGI Symbol;Acc:MGI:2155705] | yes|up |
| ENSMUSG00000043811 | Rtn4r | reticulon 4 receptor [Source:MGI Symbol;Acc:MGI:2136886] | yes|up |
| ENSMUSG00000024912 | Fosl1 | fos-like antigen 1 [Source:MGI Symbol;Acc:MGI:107179] | yes|down |
| ENSMUSG00000030978 | Rrm1 | ribonucleotide reductase M1 [Source:MGI Symbol;Acc:MGI:98180] | yes|down |
| ENSMUSG00000037653 | Kctd8 | potassium channel tetramerisation domain containing 8 [Source:MGI Symbol;Acc:MGI:2443804] | yes|up |
| ENSMUSG00000030972 | Acsm5 | acyl-CoA synthetase medium-chain family member 5 [Source:MGI Symbol;Acc:MGI:2444086] | yes|up |
| ENSMUSG00000051599 | Pcdhb2 | protocadherin beta 2 [Source:MGI Symbol;Acc:MGI:2136735] | yes|up |
| ENSMUSG00000090643 | Gm3453 | predicted gene 3453 [Source:MGI Symbol;Acc:MGI:3781629] | yes|down |
| ENSMUSG00000037474 | Dtl | denticleless E3 ubiquitin protein ligase [Source:MGI Symbol;Acc:MGI:1924093] | yes|down |
| ENSMUSG00000027939 | Nup210l | nucleoporin 210-like [Source:MGI Symbol;Acc:MGI:1924845] | yes|down |
| ENSMUSG00000074272 | Ceacam1 | carcinoembryonic antigen-related cell adhesion molecule 1 [Source:MGI Symbol;Acc:MGI:1347245] | yes|down |
| ENSMUSG00000024059 | Clip4 | CAP-GLY domain containing linker protein family, member 4 [Source:MGI Symbol;Acc:MGI:1919100] | yes|down |
| ENSMUSG00000039556 | Ppp1r3f | protein phosphatase 1, regulatory subunit 3F [Source:MGI Symbol;Acc:MGI:1859617] | yes|up |
| ENSMUSG00000026042 | Col5a2 | collagen, type V, alpha 2 [Source:MGI Symbol;Acc:MGI:88458] | yes|up |
| ENSMUSG00000026043 | Col3a1 | collagen, type III, alpha 1 [Source:MGI Symbol;Acc:MGI:88453] | yes|up |
| ENSMUSG00000023039 | Krt7 | keratin 7 [Source:MGI Symbol;Acc:MGI:96704] | yes|up |
| ENSMUSG00000025969 | Nrp2 | neuropilin 2 [Source:MGI Symbol;Acc:MGI:1100492] | yes|up |
| ENSMUSG00000056457 | Prl2c3 | prolactin family 2, subfamily c, member 3 [Source:MGI Symbol;Acc:MGI:1341833] | yes|down |
| ENSMUSG00000021091 | Serpina3n | serine (or cysteine) peptidase inhibitor, clade A, member 3N [Source:MGI Symbol;Acc:MGI:105045] | yes|up |
| ENSMUSG00000051339 | 2900026A02Rik | RIKEN cDNA 2900026A02 gene [Source:MGI Symbol;Acc:MGI:1920194] | yes|up |
| ENSMUSG00000021097 | Clmn | calmin [Source:MGI Symbol;Acc:MGI:2136957] | yes|up |
| ENSMUSG00000031730 | Dhodh | dihydroorotate dehydrogenase [Source:MGI Symbol;Acc:MGI:1928378] | yes|down |
| ENSMUSG00000069670 | Nkain2 | Na+/K+ transporting ATPase interacting 2 [Source:MGI Symbol;Acc:MGI:1923447] | yes|down |
| ENSMUSG00000030737 | Slco2b1 | solute carrier organic anion transporter family, member 2b1 [Source:MGI Symbol;Acc:MGI:1351872] | yes|up |
| ENSMUSG00000038011 | Dnah10 | dynein, axonemal, heavy chain 10 [Source:MGI Symbol;Acc:MGI:1860299] | yes|up |
| ENSMUSG00000098318 | Lockd | lncRNA downstream of Cdkn1b [Source:MGI Symbol;Acc:MGI:1915081] | yes|down |
| ENSMUSG00000029861 | Fam131b | family with sequence similarity 131, member B [Source:MGI Symbol;Acc:MGI:1923406] | yes|down |
| ENSMUSG00000022615 | Tymp | thymidine phosphorylase [Source:MGI Symbol;Acc:MGI:1920212] | yes|up |
| ENSMUSG00000029864 | Gstk1 | glutathione S-transferase kappa 1 [Source:MGI Symbol;Acc:MGI:1923513] | yes|up |
| ENSMUSG00000022346 | Myc | myelocytomatosis oncogene [Source:MGI Symbol;Acc:MGI:97250] | yes|down |
| ENSMUSG00000029869 | Ephb6 | Eph receptor B6 [Source:MGI Symbol;Acc:MGI:1096338] | yes|down |
| ENSMUSG00000029648 | Flt1 | FMS-like tyrosine kinase 1 [Source:MGI Symbol;Acc:MGI:95558] | yes|up |
| ENSMUSG00000038175 | Mylip | myosin regulatory light chain interacting protein [Source:MGI Symbol;Acc:MGI:2388271] | yes|up |
| ENSMUSG00000057278 | Snrpg | small nuclear ribonucleoprotein polypeptide G [Source:MGI Symbol;Acc:MGI:1915261] | yes|down |
| ENSMUSG00000025347 | Mettl7b | methyltransferase like 7B [Source:MGI Symbol;Acc:MGI:1918914] | yes|up |
| ENSMUSG00000028111 | Ctsk | cathepsin K [Source:MGI Symbol;Acc:MGI:107823] | yes|up |
| ENSMUSG00000025348 | Itga7 | integrin alpha 7 [Source:MGI Symbol;Acc:MGI:102700] | yes|up |
| ENSMUSG00000047907 | Tshz2 | teashirt zinc finger family member 2 [Source:MGI Symbol;Acc:MGI:2153084] | yes|up |
| ENSMUSG00000085569 | Gm12602 | predicted gene 12602 [Source:MGI Symbol;Acc:MGI:3651757] | yes|down |
| ENSMUSG00000064358 | mt-Co3 | mitochondrially encoded cytochrome c oxidase III [Source:MGI Symbol;Acc:MGI:102502] | yes|down |
| ENSMUSG00000042190 | Cmklr1 | chemokine-like receptor 1 [Source:MGI Symbol;Acc:MGI:109603] | yes|up |
| ENSMUSG00000063430 | Wscd2 | WSC domain containing 2 [Source:MGI Symbol;Acc:MGI:2445030] | yes|up |
| ENSMUSG00000014232 | Cluap1 | clusterin associated protein 1 [Source:MGI Symbol;Acc:MGI:1924029] | yes|down |
| ENSMUSG00000064354 | mt-Co2 | mitochondrially encoded cytochrome c oxidase II [Source:MGI Symbol;Acc:MGI:102503] | yes|up |
| ENSMUSG00000046056 | Sbsn | suprabasin [Source:MGI Symbol;Acc:MGI:2446326] | yes|down |
| ENSMUSG00000052229 | Gpr17 | G protein-coupled receptor 17 [Source:MGI Symbol;Acc:MGI:3584514] | yes|up |
| ENSMUSG00000087659 | Gm12606 | predicted gene 12606 [Source:MGI Symbol;Acc:MGI:3649222] | yes|down |
| ENSMUSG00000029651 | Mtus2 | microtubule associated tumor suppressor candidate 2 [Source:MGI Symbol;Acc:MGI:1915388] | yes|up |
| ENSMUSG00000114277 | Gm48583 | predicted gene, 48583 [Source:MGI Symbol;Acc:MGI:6098152] | yes|down |
| ENSMUSG00000032415 | Ube2cbp | ubiquitin-conjugating enzyme E2C binding protein [Source:MGI Symbol;Acc:MGI:1917598] | yes|down |
| ENSMUSG00000084838 | Gm10241 | predicted pseudogene 10241 [Source:MGI Symbol;Acc:MGI:3704461] | yes|up |
| ENSMUSG00000019214 | Chtf18 | CTF18, chromosome transmission fidelity factor 18 [Source:MGI Symbol;Acc:MGI:2384887] | yes|down |
| ENSMUSG00000029231 | Pdgfra | platelet derived growth factor receptor, alpha polypeptide [Source:MGI Symbol;Acc:MGI:97530] | yes|down |
| ENSMUSG00000053175 | Bcl3 | B cell leukemia/lymphoma 3 [Source:MGI Symbol;Acc:MGI:88140] | yes|up |
| ENSMUSG00000079560 | Hoxa3 | homeobox A3 [Source:MGI Symbol;Acc:MGI:96175] | yes|up |
| ENSMUSG00000079563 | Pglyrp2 | peptidoglycan recognition protein 2 [Source:MGI Symbol;Acc:MGI:1928099] | yes|up |
| ENSMUSG00000060424 | Pantr1 | POU domain, class 3, transcription factor 3 adjacent noncoding transcript 1 [Source:MGI Symbol;Acc:MGI:1913547] | yes|down |
| ENSMUSG00000060429 | Sntb1 | syntrophin, basic 1 [Source:MGI Symbol;Acc:MGI:101781] | yes|up |
| ENSMUSG00000054196 | Cthrc1 | collagen triple helix repeat containing 1 [Source:MGI Symbol;Acc:MGI:1915838] | yes|up |
| ENSMUSG00000033544 | Angptl1 | angiopoietin-like 1 [Source:MGI Symbol;Acc:MGI:1919963] | yes|up |
| ENSMUSG00000033540 | Idua | iduronidase, alpha-L [Source:MGI Symbol;Acc:MGI:96418] | yes|up |
| ENSMUSG00000000628 | Hk2 | hexokinase 2 [Source:MGI Symbol;Acc:MGI:1315197] | yes|up |
| ENSMUSG00000005470 | Asf1b | anti-silencing function 1B histone chaperone [Source:MGI Symbol;Acc:MGI:1914179] | yes|down |
| ENSMUSG00000041559 | Fmod | fibromodulin [Source:MGI Symbol;Acc:MGI:1328364] | yes|up |
| ENSMUSG00000108042 | B130021K23Rik | RIKEN cDNA B130021K23 gene [Source:MGI Symbol;Acc:MGI:2442518] | yes|up |
| ENSMUSG00000049313 | Sorl1 | sortilin-related receptor, LDLR class A repeats-containing [Source:MGI Symbol;Acc:MGI:1202296] | yes|down |
| ENSMUSG00000032607 | Amt | aminomethyltransferase [Source:MGI Symbol;Acc:MGI:3646700] | yes|up |
| ENSMUSG00000081824 | Ndufs5-ps | NADH:ubiquinone oxidoreductase core subunit S5, pseudogene [Source:MGI Symbol;Acc:MGI:3612445] | yes|down |
| ENSMUSG00000097101 | 1810034E14Rik | RIKEN cDNA 1810034E14 gene [Source:MGI Symbol;Acc:MGI:1913753] | yes|down |
| ENSMUSG00000027239 | Mdk | midkine [Source:MGI Symbol;Acc:MGI:96949] | yes|up |
| ENSMUSG00000027580 | Helz2 | helicase with zinc finger 2, transcriptional coactivator [Source:MGI Symbol;Acc:MGI:2385169] | yes|up |
| ENSMUSG00000027230 | Creb3l1 | cAMP responsive element binding protein 3-like 1 [Source:MGI Symbol;Acc:MGI:1347062] | yes|up |
| ENSMUSG00000020911 | Krt19 | keratin 19 [Source:MGI Symbol;Acc:MGI:96693] | yes|down |
| ENSMUSG00000027236 | Eif3j1 | eukaryotic translation initiation factor 3, subunit J1 [Source:MGI Symbol;Acc:MGI:1925905] | yes|down |
| ENSMUSG00000020914 | Top2a | topoisomerase (DNA) II alpha [Source:MGI Symbol;Acc:MGI:98790] | yes|down |
| ENSMUSG00000026185 | Igfbp5 | insulin-like growth factor binding protein 5 [Source:MGI Symbol;Acc:MGI:96440] | yes|up |
| ENSMUSG00000017344 | Vtn | vitronectin [Source:MGI Symbol;Acc:MGI:98940] | yes|up |
| ENSMUSG00000092167 | Gm3696 | predicted gene 3696 [Source:MGI Symbol;Acc:MGI:3781872] | yes|up |
| ENSMUSG00000056737 | Capg | capping protein (actin filament), gelsolin-like [Source:MGI Symbol;Acc:MGI:1098259] | yes|down |
| ENSMUSG00000040473 | Cfap69 | cilia and flagella associated protein 69 [Source:MGI Symbol;Acc:MGI:2443778] | yes|down |
| ENSMUSG00000066107 | Gm12666 | predicted gene 12666 [Source:MGI Symbol;Acc:MGI:3650864] | yes|up |
| ENSMUSG00000115317 | Gm32618 | predicted gene, 32618 [Source:MGI Symbol;Acc:MGI:5591777] | yes|up |
| ENSMUSG00000056596 | Trnp1 | TMF1-regulated nuclear protein 1 [Source:MGI Symbol;Acc:MGI:1916789] | yes|up |
| ENSMUSG00000021974 | Fgf9 | fibroblast growth factor 9 [Source:MGI Symbol;Acc:MGI:104723] | yes|up |
| ENSMUSG00000026981 | Il1rn | interleukin 1 receptor antagonist [Source:MGI Symbol;Acc:MGI:96547] | yes|up |
| ENSMUSG00000070960 | Gm19680 | predicted gene, 19680 [Source:MGI Symbol;Acc:MGI:5011865] | yes|down |
| ENSMUSG00000025579 | Gaa | glucosidase, alpha, acid [Source:MGI Symbol;Acc:MGI:95609] | yes|up |
| ENSMUSG00000039145 | Camk1d | calcium/calmodulin-dependent protein kinase ID [Source:MGI Symbol;Acc:MGI:2442190] | yes|down |
| ENSMUSG00000022407 | Adsl | adenylosuccinate lyase [Source:MGI Symbol;Acc:MGI:103202] | yes|down |
| ENSMUSG00000029456 | Acad10 | acyl-Coenzyme A dehydrogenase family, member 10 [Source:MGI Symbol;Acc:MGI:1919235] | yes|down |
| ENSMUSG00000042766 | Trim46 | tripartite motif-containing 46 [Source:MGI Symbol;Acc:MGI:2673000] | yes|up |
| ENSMUSG00000034488 | Edil3 | EGF-like repeats and discoidin I-like domains 3 [Source:MGI Symbol;Acc:MGI:1329025] | yes|up |
| ENSMUSG00000028587 | Orc1 | origin recognition complex, subunit 1 [Source:MGI Symbol;Acc:MGI:1328337] | yes|down |
| ENSMUSG00000028581 | Laptm5 | lysosomal-associated protein transmembrane 5 [Source:MGI Symbol;Acc:MGI:108046] | yes|up |
| ENSMUSG00000028583 | Pdpn | podoplanin [Source:MGI Symbol;Acc:MGI:103098] | yes|down |
| ENSMUSG00000022092 | Ppp3cc | protein phosphatase 3, catalytic subunit, gamma isoform [Source:MGI Symbol;Acc:MGI:107162] | yes|down |
| ENSMUSG00000034485 | Uaca | uveal autoantigen with coiled-coil domains and ankyrin repeats [Source:MGI Symbol;Acc:MGI:1919815] | yes|down |
| ENSMUSG00000025758 | Plk4 | polo like kinase 4 [Source:MGI Symbol;Acc:MGI:101783] | yes|down |
| ENSMUSG00000094777 | H2ac24 | H2A clustered histone 24 [Source:MGI Symbol;Acc:MGI:3710573] | yes|up |
| ENSMUSG00000049881 | 2810025M15Rik | RIKEN cDNA 2810025M15 gene [Source:MGI Symbol;Acc:MGI:1917203] | yes|down |
| ENSMUSG00000049882 | Vcpkmt | valosin containing protein lysine (K) methyltransferase [Source:MGI Symbol;Acc:MGI:2684917] | yes|down |
| ENSMUSG00000035829 | Ppp1r26 | protein phosphatase 1, regulatory subunit 26 [Source:MGI Symbol;Acc:MGI:2685193] | yes|up |
| ENSMUSG00000038816 | Ctnnal1 | catenin (cadherin associated protein), alpha-like 1 [Source:MGI Symbol;Acc:MGI:1859649] | yes|down |
| ENSMUSG00000027860 | Vangl1 | VANGL planar cell polarity 1 [Source:MGI Symbol;Acc:MGI:2159344] | yes|down |
| ENSMUSG00000059908 | Mug1 | murinoglobulin 1 [Source:MGI Symbol;Acc:MGI:99837] | yes|up |
| ENSMUSG00000053615 | Gm9913 | predicted gene 9913 [Source:MGI Symbol;Acc:MGI:3642395] | yes|up |
| ENSMUSG00000091228 | Gm20390 | predicted gene 20390 [Source:MGI Symbol;Acc:MGI:5141855] | yes|down |
| ENSMUSG00000028327 | Stra6l | STRA6-like [Source:MGI Symbol;Acc:MGI:1921402] | yes|up |
| ENSMUSG00000032083 | Apoa1 | apolipoprotein A-I [Source:MGI Symbol;Acc:MGI:88049] | yes|up |
| ENSMUSG00000032081 | Apoc3 | apolipoprotein C-III [Source:MGI Symbol;Acc:MGI:88055] | yes|up |
| ENSMUSG00000032080 | Apoa4 | apolipoprotein A-IV [Source:MGI Symbol;Acc:MGI:88051] | yes|up |
| ENSMUSG00000086843 | E030013I19Rik | RIKEN cDNA E030013I19 gene [Source:MGI Symbol;Acc:MGI:2443735] | yes|up |
| ENSMUSG00000020135 | Apc2 | APC regulator of WNT signaling pathway 2 [Source:MGI Symbol;Acc:MGI:1346052] | yes|down |
| ENSMUSG00000020053 | Igf1 | insulin-like growth factor 1 [Source:MGI Symbol;Acc:MGI:96432] | yes|up |
| ENSMUSG00000070372 | Capza1 | capping protein (actin filament) muscle Z-line, alpha 1 [Source:MGI Symbol;Acc:MGI:106227] | yes|down |
| ENSMUSG00000020051 | Pah | phenylalanine hydroxylase [Source:MGI Symbol;Acc:MGI:97473] | yes|up |
| ENSMUSG00000039853 | Trim14 | tripartite motif-containing 14 [Source:MGI Symbol;Acc:MGI:1921985] | yes|up |
| ENSMUSG00000107018 | Gm43694 | predicted gene 43694 [Source:MGI Symbol;Acc:MGI:5663831] | yes|down |
| ENSMUSG00000096629 | Gm3383 | predicted gene 3383 [Source:MGI Symbol;Acc:MGI:3781561] | yes|down |
| ENSMUSG00000086596 | Susd5 | sushi domain containing 5 [Source:MGI Symbol;Acc:MGI:2685972] | yes|up |
| ENSMUSG00000032889 | Gm6685 | predicted pseudogene 6685 [Source:MGI Symbol;Acc:MGI:3704119] | yes|down |
| ENSMUSG00000092283 | Gm20412 | predicted gene 20412 [Source:MGI Symbol;Acc:MGI:5141877] | yes|up |
| ENSMUSG00000040212 | Emp3 | epithelial membrane protein 3 [Source:MGI Symbol;Acc:MGI:1098729] | yes|down |
| ENSMUSG00000033022 | Cdo1 | cysteine dioxygenase 1, cytosolic [Source:MGI Symbol;Acc:MGI:105925] | yes|up |
| ENSMUSG00000033752 | Mnd1 | meiotic nuclear divisions 1 [Source:MGI Symbol;Acc:MGI:1924165] | yes|down |
| ENSMUSG00000039706 | Ldb2 | LIM domain binding 2 [Source:MGI Symbol;Acc:MGI:894670] | yes|up |
| ENSMUSG00000039701 | Usp53 | ubiquitin specific peptidase 53 [Source:MGI Symbol;Acc:MGI:2139607] | yes|up |
| ENSMUSG00000024247 | Pkdcc | protein kinase domain containing, cytoplasmic [Source:MGI Symbol;Acc:MGI:2147077] | yes|up |
| ENSMUSG00000116358 | Gm49450 | predicted gene, 49450 [Source:MGI Symbol;Acc:MGI:6155101] | yes|up |
| ENSMUSG00000024245 | Tmem178 | transmembrane protein 178 [Source:MGI Symbol;Acc:MGI:1915277] | yes|up |
| ENSMUSG00000027500 | Stmn2 | stathmin-like 2 [Source:MGI Symbol;Acc:MGI:98241] | yes|down |
| ENSMUSG00000023236 | Scg5 | secretogranin V [Source:MGI Symbol;Acc:MGI:98289] | yes|down |
| ENSMUSG00000027913 | Crct1 | cysteine-rich C-terminal 1 [Source:MGI Symbol;Acc:MGI:1921425] | yes|up |
| ENSMUSG00000073557 | Ppp1r12b | protein phosphatase 1, regulatory subunit 12B [Source:MGI Symbol;Acc:MGI:1916417] | yes|down |
| ENSMUSG00000027919 | Lce1g | late cornified envelope 1G [Source:MGI Symbol;Acc:MGI:1913445] | yes|up |
| ENSMUSG00000073555 | Gm4951 | predicted gene 4951 [Source:MGI Symbol;Acc:MGI:3644953] | yes|up |
| ENSMUSG00000050445 | Cyp8b1 | cytochrome P450, family 8, subfamily b, polypeptide 1 [Source:MGI Symbol;Acc:MGI:1338044] | yes|up |
| ENSMUSG00000050440 | Hamp | hepcidin antimicrobial peptide [Source:MGI Symbol;Acc:MGI:1933533] | yes|up |
| ENSMUSG00000031538 | Plat | plasminogen activator, tissue [Source:MGI Symbol;Acc:MGI:97610] | yes|up |
| ENSMUSG00000026068 | Il18rap | interleukin 18 receptor accessory protein [Source:MGI Symbol;Acc:MGI:1338888] | yes|down |
| ENSMUSG00000026069 | Il1rl1 | interleukin 1 receptor-like 1 [Source:MGI Symbol;Acc:MGI:98427] | yes|down |
| ENSMUSG00000030220 | Arhgdib | Rho, GDP dissociation inhibitor (GDI) beta [Source:MGI Symbol;Acc:MGI:101940] | yes|down |
| ENSMUSG00000030222 | Rerg | RAS-like, estrogen-regulated, growth-inhibitor [Source:MGI Symbol;Acc:MGI:2665139] | yes|up |
| ENSMUSG00000069601 | Ank3 | ankyrin 3, epithelial [Source:MGI Symbol;Acc:MGI:88026] | yes|down |
| ENSMUSG00000097715 | Gpr137b-ps | G protein-coupled receptor 137B, pseudogene [Source:MGI Symbol;Acc:MGI:3710533] | yes|down |
| ENSMUSG00000041859 | Mcm3 | minichromosome maintenance complex component 3 [Source:MGI Symbol;Acc:MGI:101845] | yes|down |
| ENSMUSG00000062168 | Ppef1 | protein phosphatase with EF hand calcium-binding domain 1 [Source:MGI Symbol;Acc:MGI:1097157] | yes|up |
| ENSMUSG00000056476 | Med12l | mediator complex subunit 12-like [Source:MGI Symbol;Acc:MGI:2139916] | yes|up |
| ENSMUSG00000022180 | Slc7a8 | solute carrier family 7 (cationic amino acid transporter, y+ system), member 8 [Source:MGI Symbol;Acc:MGI:1355323] | yes|down |
| ENSMUSG00000049502 | Dtx3l | deltex 3-like, E3 ubiquitin ligase [Source:MGI Symbol;Acc:MGI:2656973] | yes|up |
| ENSMUSG00000038155 | Gstp2 | glutathione S-transferase, pi 2 [Source:MGI Symbol;Acc:MGI:95864] | yes|down |
| ENSMUSG00000103928 | Gm37893 | predicted gene, 37893 [Source:MGI Symbol;Acc:MGI:5611121] | yes|up |
| ENSMUSG00000097906 | Gm9625 | predicted gene 9625 [Source:MGI Symbol;Acc:MGI:3780033] | yes|down |
| ENSMUSG00000063383 | Zfp947 | zinc finger protein 947 [Source:MGI Symbol;Acc:MGI:3646759] | yes|down |
| ENSMUSG00000029661 | Col1a2 | collagen, type I, alpha 2 [Source:MGI Symbol;Acc:MGI:88468] | yes|up |
| ENSMUSG00000015980 | Lrrc27 | leucine rich repeat containing 27 [Source:MGI Symbol;Acc:MGI:1923862] | yes|up |
| ENSMUSG00000028134 | Ptbp2 | polypyrimidine tract binding protein 2 [Source:MGI Symbol;Acc:MGI:1860489] | yes|down |
| ENSMUSG00000039470 | Zdhhc2 | zinc finger, DHHC domain containing 2 [Source:MGI Symbol;Acc:MGI:1923452] | yes|up |
| ENSMUSG00000037519 | Ppfia1 | protein tyrosine phosphatase, receptor type, f polypeptide (PTPRF), interacting protein (liprin), alpha 1 [Source:MGI Symbol;Acc:MGI:1924750] | yes|up |
| ENSMUSG00000102307 | Gm38194 | predicted gene, 38194 [Source:MGI Symbol;Acc:MGI:5611422] | yes|up |
| ENSMUSG00000089901 | Gm8113 | predicted gene 8113 [Source:MGI Symbol;Acc:MGI:3648791] | yes|up |
| ENSMUSG00000078143 | Gm17344 | predicted gene, 17344 [Source:MGI Symbol;Acc:MGI:4936978] | yes|down |
| ENSMUSG00000034342 | Cbl | Casitas B-lineage lymphoma [Source:MGI Symbol;Acc:MGI:88279] | yes|down |
| ENSMUSG00000034839 | Larp6 | La ribonucleoprotein domain family, member 6 [Source:MGI Symbol;Acc:MGI:1914807] | yes|up |
| ENSMUSG00000111481 | Gm47652 | predicted gene, 47652 [Source:MGI Symbol;Acc:MGI:6096735] | yes|up |
| ENSMUSG00000034349 | Smc4 | structural maintenance of chromosomes 4 [Source:MGI Symbol;Acc:MGI:1917349] | yes|down |
| ENSMUSG00000018830 | Myh11 | myosin, heavy polypeptide 11, smooth muscle [Source:MGI Symbol;Acc:MGI:102643] | yes|up |
| ENSMUSG00000039286 | Fndc3b | fibronectin type III domain containing 3B [Source:MGI Symbol;Acc:MGI:1919257] | yes|up |
| ENSMUSG00000054753 | AU018091 | expressed sequence AU018091 [Source:MGI Symbol;Acc:MGI:2142124] | yes|up |
| ENSMUSG00000120367 |  | novel transcript | yes|down |
| ENSMUSG00000120364 |  | novel transcript | yes|up |
| ENSMUSG00000042249 | Grk3 | G protein-coupled receptor kinase 3 [Source:MGI Symbol;Acc:MGI:87941] | yes|down |
| ENSMUSG00000045294 | Insig1 | insulin induced gene 1 [Source:MGI Symbol;Acc:MGI:1916289] | yes|up |
| ENSMUSG00000103076 | Gm37902 | predicted gene, 37902 [Source:MGI Symbol;Acc:MGI:5611130] | yes|up |
| ENSMUSG00000103071 | Gm38110 | predicted gene, 38110 [Source:MGI Symbol;Acc:MGI:5611338] | yes|up |
| ENSMUSG00000114210 | A330084C13Rik | RIKEN cDNA A330084C13 gene [Source:MGI Symbol;Acc:MGI:2685802] | yes|up |
| ENSMUSG00000017057 | Il13ra1 | interleukin 13 receptor, alpha 1 [Source:MGI Symbol;Acc:MGI:105052] | yes|up |
| ENSMUSG00000062762 | Ei24 | etoposide induced 2.4 mRNA [Source:MGI Symbol;Acc:MGI:108090] | yes|down |
| ENSMUSG00000015568 | Lpl | lipoprotein lipase [Source:MGI Symbol;Acc:MGI:96820] | yes|up |
| ENSMUSG00000019894 | Slc6a15 | solute carrier family 6 (neurotransmitter transporter), member 15 [Source:MGI Symbol;Acc:MGI:2143484] | yes|down |
| ENSMUSG00000036083 | Slc17a3 | solute carrier family 17 (sodium phosphate), member 3 [Source:MGI Symbol;Acc:MGI:2389216] | yes|up |
| ENSMUSG00000053158 | Fes | feline sarcoma oncogene [Source:MGI Symbol;Acc:MGI:95514] | yes|down |
| ENSMUSG00000024747 | Aldh1a7 | aldehyde dehydrogenase family 1, subfamily A7 [Source:MGI Symbol;Acc:MGI:1347050] | yes|down |
| ENSMUSG00000074865 | Zfp934 | zinc finger protein 934 [Source:MGI Symbol;Acc:MGI:1924367] | yes|down |
| ENSMUSG00000024743 | Syt7 | synaptotagmin VII [Source:MGI Symbol;Acc:MGI:1859545] | yes|down |
| ENSMUSG00000029227 | Fip1l1 | FIP1 like 1 (S. cerevisiae) [Source:MGI Symbol;Acc:MGI:1914149] | yes|down |
| ENSMUSG00000044165 | Bcl2l15 | BCLl2-like 15 [Source:MGI Symbol;Acc:MGI:2685412] | yes|down |
| ENSMUSG00000060407 | Cyp2a12 | cytochrome P450, family 2, subfamily a, polypeptide 12 [Source:MGI Symbol;Acc:MGI:105055] | yes|up |
| ENSMUSG00000001802 | Lrp3 | low density lipoprotein receptor-related protein 3 [Source:MGI Symbol;Acc:MGI:3584516] | yes|up |
| ENSMUSG00000081593 | Gm11841 | predicted gene 11841 [Source:MGI Symbol;Acc:MGI:3651164] | yes|down |
| ENSMUSG00000037025 | Foxa2 | forkhead box A2 [Source:MGI Symbol;Acc:MGI:1347476] | yes|up |
| ENSMUSG00000070526 | Peg12 | paternally expressed 12 [Source:MGI Symbol;Acc:MGI:1351637] | yes|down |
| ENSMUSG00000070522 | Gm6505 | predicted pseudogene 6505 [Source:MGI Symbol;Acc:MGI:3648080] | yes|up |
| ENSMUSG00000063011 | Msln | mesothelin [Source:MGI Symbol;Acc:MGI:1888992] | yes|down |
| ENSMUSG00000040663 | Clcf1 | cardiotrophin-like cytokine factor 1 [Source:MGI Symbol;Acc:MGI:1930088] | yes|down |
| ENSMUSG00000027070 | Lrp2 | low density lipoprotein receptor-related protein 2 [Source:MGI Symbol;Acc:MGI:95794] | yes|up |
| ENSMUSG00000063172 | Hspb11 | heat shock protein family B (small), member 11 [Source:MGI Symbol;Acc:MGI:1920188] | yes|down |
| ENSMUSG00000090124 | Ugt1a7c | UDP glucuronosyltransferase 1 family, polypeptide A7C [Source:MGI Symbol;Acc:MGI:3032636] | yes|down |
| ENSMUSG00000079654 | Prrt4 | proline-rich transmembrane protein 4 [Source:MGI Symbol;Acc:MGI:2141677] | yes|down |
| ENSMUSG00000000861 | Bcl11a | B cell CLL/lymphoma 11A (zinc finger protein) [Source:MGI Symbol;Acc:MGI:106190] | yes|up |
| ENSMUSG00000085037 | 4933421O10Rik | RIKEN cDNA 4933421O10 gene [Source:MGI Symbol;Acc:MGI:1918323] | yes|up |
| ENSMUSG00000032741 | Tpcn1 | two pore channel 1 [Source:MGI Symbol;Acc:MGI:2182472] | yes|up |
| ENSMUSG00000048065 | Cyb5r2 | cytochrome b5 reductase 2 [Source:MGI Symbol;Acc:MGI:2444415] | yes|up |
| ENSMUSG00000039062 | Anpep | alanyl (membrane) aminopeptidase [Source:MGI Symbol;Acc:MGI:5000466] | yes|up |
| ENSMUSG00000043290 | Zfp784 | zinc finger protein 784 [Source:MGI Symbol;Acc:MGI:3606042] | yes|down |
| ENSMUSG00000060147 | Serpinb6a | serine (or cysteine) peptidase inhibitor, clade B, member 6a [Source:MGI Symbol;Acc:MGI:103123] | yes|down |
| ENSMUSG00000031070 | Mrgprf | MAS-related GPR, member F [Source:MGI Symbol;Acc:MGI:2384823] | yes|up |
| ENSMUSG00000057406 | Nsd2 | nuclear receptor binding SET domain protein 2 [Source:MGI Symbol;Acc:MGI:1276574] | yes|down |
| ENSMUSG00000057400 | Ces1c | carboxylesterase 1C [Source:MGI Symbol;Acc:MGI:95420] | yes|up |
| ENSMUSG00000026308 | Klhl30 | kelch-like 30 [Source:MGI Symbol;Acc:MGI:1918038] | yes|up |
| ENSMUSG00000028834 | Trim63 | tripartite motif-containing 63 [Source:MGI Symbol;Acc:MGI:2447992] | yes|up |
| ENSMUSG00000028838 | Extl1 | exostosin-like glycosyltransferase 1 [Source:MGI Symbol;Acc:MGI:1888742] | yes|up |
| ENSMUSG00000034640 | Tiparp | TCDD-inducible poly(ADP-ribose) polymerase [Source:MGI Symbol;Acc:MGI:2159210] | yes|down |
| ENSMUSG00000098292 | Gm27194 | predicted gene 27194 [Source:MGI Symbol;Acc:MGI:5521037] | yes|down |
| ENSMUSG00000029307 | Dmp1 | dentin matrix protein 1 [Source:MGI Symbol;Acc:MGI:94910] | yes|down |
| ENSMUSG00000022422 | Dscc1 | DNA replication and sister chromatid cohesion 1 [Source:MGI Symbol;Acc:MGI:1919357] | yes|down |
| ENSMUSG00000046159 | Chrm3 | cholinergic receptor, muscarinic 3, cardiac [Source:MGI Symbol;Acc:MGI:88398] | yes|up |
| ENSMUSG00000060397 | Zfp128 | zinc finger protein 128 [Source:MGI Symbol;Acc:MGI:2389445] | yes|up |
| ENSMUSG00000120883 |  | novel transcript | yes|up |
| ENSMUSG00000021377 | Dek | DEK proto-oncogene (DNA binding) [Source:MGI Symbol;Acc:MGI:1926209] | yes|down |
| ENSMUSG00000035513 | Ntng2 | netrin G2 [Source:MGI Symbol;Acc:MGI:2159341] | yes|up |
| ENSMUSG00000033910 | Gucy1a1 | guanylate cyclase 1, soluble, alpha 1 [Source:MGI Symbol;Acc:MGI:1926562] | yes|up |
| ENSMUSG00000028307 | Aldob | aldolase B, fructose-bisphosphate [Source:MGI Symbol;Acc:MGI:87995] | yes|up |
| ENSMUSG00000021379 | Id4 | inhibitor of DNA binding 4 [Source:MGI Symbol;Acc:MGI:99414] | yes|down |
| ENSMUSG00000078087 | Rps12l1 | ribosomal protein S12-like 1 [Source:MGI Symbol;Acc:MGI:3783241] | yes|down |
| ENSMUSG00000120081 |  | novel transcript | yes|up |
| ENSMUSG00000094410 | Zbed6 | zinc finger, BED type containing 6 [Source:MGI Symbol;Acc:MGI:3828086] | yes|up |
| ENSMUSG00000020155 | Kcnmb1 | potassium large conductance calcium-activated channel, subfamily M, beta member 1 [Source:MGI Symbol;Acc:MGI:1334203] | yes|up |
| ENSMUSG00000022881 | Rfc4 | replication factor C (activator 1) 4 [Source:MGI Symbol;Acc:MGI:2146571] | yes|down |
| ENSMUSG00000052415 | Tchh | trichohyalin [Source:MGI Symbol;Acc:MGI:2177944] | yes|up |
| ENSMUSG00000078886 | Gm2026 | predicted gene 2026 [Source:MGI Symbol;Acc:MGI:3780195] | yes|down |
| ENSMUSG00000078881 | Gm14434 | predicted gene 14434 [Source:MGI Symbol;Acc:MGI:3702417] | yes|down |
| ENSMUSG00000001120 | Pcbp3 | poly(rC) binding protein 3 [Source:MGI Symbol;Acc:MGI:1890470] | yes|down |
| ENSMUSG00000078889 | Gm14288 | predicted gene 14288 [Source:MGI Symbol;Acc:MGI:3706570] | yes|down |
| ENSMUSG00000001128 | Cfp | complement factor properdin [Source:MGI Symbol;Acc:MGI:97545] | yes|up |
| ENSMUSG00000002835 | Chaf1a | chromatin assembly factor 1, subunit A (p150) [Source:MGI Symbol;Acc:MGI:1351331] | yes|down |
| ENSMUSG00000024481 | Lvrn | laeverin [Source:MGI Symbol;Acc:MGI:1921824] | yes|up |
| ENSMUSG00000032207 | Lipc | lipase, hepatic [Source:MGI Symbol;Acc:MGI:96216] | yes|up |
| ENSMUSG00000030935 | Acsm3 | acyl-CoA synthetase medium-chain family member 3 [Source:MGI Symbol;Acc:MGI:99538] | yes|up |
| ENSMUSG00000037617 | Spag1 | sperm associated antigen 1 [Source:MGI Symbol;Acc:MGI:1349387] | yes|down |
| ENSMUSG00000036523 | Greb1 | gene regulated by estrogen in breast cancer protein [Source:MGI Symbol;Acc:MGI:2149712] | yes|up |
| ENSMUSG00000036256 | Igfbp7 | insulin-like growth factor binding protein 7 [Source:MGI Symbol;Acc:MGI:1352480] | yes|up |
| ENSMUSG00000080875 | Gm7332 | predicted gene 7332 [Source:MGI Symbol;Acc:MGI:3643344] | yes|up |
| ENSMUSG00000000296 | Tpd52l1 | tumor protein D52-like 1 [Source:MGI Symbol;Acc:MGI:1298386] | yes|up |
| ENSMUSG00000000295 | Hddc2 | HD domain containing 2 [Source:MGI Symbol;Acc:MGI:1916942] | yes|down |
| ENSMUSG00000024269 | Tpgs2 | tubulin polyglutamylase complex subunit 2 [Source:MGI Symbol;Acc:MGI:1913898] | yes|down |
| ENSMUSG00000067017 | Capza1-ps1 | capping protein (actin filament) muscle Z-line, alpha 1, pseudogene 1 [Source:MGI Symbol;Acc:MGI:106236] | yes|up |
| ENSMUSG00000059325 | Hopx | HOP homeobox [Source:MGI Symbol;Acc:MGI:1916782] | yes|down |
| ENSMUSG00000059326 | Csf2ra | colony stimulating factor 2 receptor, alpha, low-affinity (granulocyte-macrophage) [Source:MGI Symbol;Acc:MGI:1339754] | yes|up |
| ENSMUSG00000061947 | Serpina10 | serine (or cysteine) peptidase inhibitor, clade A (alpha-1 antiproteinase, antitrypsin), member 10 [Source:MGI Symbol;Acc:MGI:2667725] | yes|up |
| ENSMUSG00000056673 | Kdm5d | lysine (K)-specific demethylase 5D [Source:MGI Symbol;Acc:MGI:99780] | yes|down |
| ENSMUSG00000031519 | Asb5 | ankyrin repeat and SOCs box-containing 5 [Source:MGI Symbol;Acc:MGI:1923544] | yes|up |
| ENSMUSG00000050310 | Rictor | RPTOR independent companion of MTOR, complex 2 [Source:MGI Symbol;Acc:MGI:1926007] | yes|up |
| ENSMUSG00000055493 | Epm2a | epilepsy, progressive myoclonic epilepsy, type 2 gene alpha [Source:MGI Symbol;Acc:MGI:1341085] | yes|up |
| ENSMUSG00000050315 | Synpo2 | synaptopodin 2 [Source:MGI Symbol;Acc:MGI:2153070] | yes|up |
| ENSMUSG00000030208 | Emp1 | epithelial membrane protein 1 [Source:MGI Symbol;Acc:MGI:107941] | yes|down |
| ENSMUSG00000012428 | Steap4 | STEAP family member 4 [Source:MGI Symbol;Acc:MGI:1923560] | yes|up |
| ENSMUSG00000059146 | Ntrk3 | neurotrophic tyrosine kinase, receptor, type 3 [Source:MGI Symbol;Acc:MGI:97385] | yes|up |
| ENSMUSG00000015340 | Cybb | cytochrome b-245, beta polypeptide [Source:MGI Symbol;Acc:MGI:88574] | yes|up |
| ENSMUSG00000051378 | Kif18b | kinesin family member 18B [Source:MGI Symbol;Acc:MGI:2446979] | yes|down |
| ENSMUSG00000091957 | Rps2-ps10 | ribosomal protein S2, pseudogene 10 [Source:MGI Symbol;Acc:MGI:3645604] | yes|down |
| ENSMUSG00000112478 | Gm47761 | predicted gene, 47761 [Source:MGI Symbol;Acc:MGI:6096912] | yes|down |
| ENSMUSG00000022304 | Dpys | dihydropyrimidinase [Source:MGI Symbol;Acc:MGI:1928679] | yes|up |
| ENSMUSG00000029821 | Gsdme | gasdermin E [Source:MGI Symbol;Acc:MGI:1889850] | yes|down |
| ENSMUSG00000029822 | Osbpl3 | oxysterol binding protein-like 3 [Source:MGI Symbol;Acc:MGI:1918970] | yes|down |
| ENSMUSG00000035187 | Nkx6-1 | NK6 homeobox 1 [Source:MGI Symbol;Acc:MGI:1206039] | yes|up |
| ENSMUSG00000035184 | Fam124a | family with sequence similarity 124, member A [Source:MGI Symbol;Acc:MGI:3645930] | yes|up |
| ENSMUSG00000025650 | Col7a1 | collagen, type VII, alpha 1 [Source:MGI Symbol;Acc:MGI:88462] | yes|up |
| ENSMUSG00000028152 | Tspan5 | tetraspanin 5 [Source:MGI Symbol;Acc:MGI:1928096] | yes|down |
| ENSMUSG00000114184 | Gm7143 | predicted gene 7143 [Source:MGI Symbol;Acc:MGI:3646528] | yes|up |
| ENSMUSG00000052776 | Oas1a | 2'-5' oligoadenylate synthetase 1A [Source:MGI Symbol;Acc:MGI:2180860] | yes|up |
| ENSMUSG00000074749 | Kiz | kizuna centrosomal protein [Source:MGI Symbol;Acc:MGI:2684960] | yes|down |
| ENSMUSG00000080316 | Spaca6 | sperm acrosome associated 6 [Source:MGI Symbol;Acc:MGI:1922452] | yes|up |
| ENSMUSG00000071862 | Lrrtm2 | leucine rich repeat transmembrane neuronal 2 [Source:MGI Symbol;Acc:MGI:2389174] | yes|down |
| ENSMUSG00000068289 | Cma2 | chymase 2, mast cell [Source:MGI Symbol;Acc:MGI:88426] | yes|up |
| ENSMUSG00000039356 | Exosc2 | exosome component 2 [Source:MGI Symbol;Acc:MGI:2385133] | yes|down |
| ENSMUSG00000050953 | Gja1 | gap junction protein, alpha 1 [Source:MGI Symbol;Acc:MGI:95713] | yes|down |
| ENSMUSG00000021536 | Adcy2 | adenylate cyclase 2 [Source:MGI Symbol;Acc:MGI:99676] | yes|up |
| ENSMUSG00000061175 | Fnip2 | folliculin interacting protein 2 [Source:MGI Symbol;Acc:MGI:2683054] | yes|up |
| ENSMUSG00000091383 | H2af-ps2 | H2A histone family, pseudogene 2 [Source:MGI Symbol;Acc:MGI:3646032] | yes|up |
| ENSMUSG00000063171 | Rps4l | ribosomal protein S4-like [Source:MGI Symbol;Acc:MGI:1913434] | yes|down |
| ENSMUSG00000064284 | Cdpf1 | cysteine rich, DPF motif domain containing 1 [Source:MGI Symbol;Acc:MGI:1919605] | yes|down |
| ENSMUSG00000022945 | Chaf1b | chromatin assembly factor 1, subunit B (p60) [Source:MGI Symbol;Acc:MGI:1314881] | yes|down |
| ENSMUSG00000041046 | Ramp3 | receptor (calcitonin) activity modifying protein 3 [Source:MGI Symbol;Acc:MGI:1860292] | yes|down |
| ENSMUSG00000001823 | Hoxd12 | homeobox D12 [Source:MGI Symbol;Acc:MGI:96204] | yes|down |
| ENSMUSG00000027276 | Jag1 | jagged 1 [Source:MGI Symbol;Acc:MGI:1095416] | yes|up |
| ENSMUSG00000020950 | Foxg1 | forkhead box G1 [Source:MGI Symbol;Acc:MGI:1347464] | yes|down |
| ENSMUSG00000014633 | Cmc2 | COX assembly mitochondrial protein 2 [Source:MGI Symbol;Acc:MGI:1913781] | yes|down |
| ENSMUSG00000058152 | Chsy3 | chondroitin sulfate synthase 3 [Source:MGI Symbol;Acc:MGI:1926173] | yes|up |
| ENSMUSG00000050761 | Gp1bb | glycoprotein Ib, beta polypeptide [Source:MGI Symbol;Acc:MGI:107852] | yes|up |
| ENSMUSG00000030055 | Rab43 | RAB43, member RAS oncogene family [Source:MGI Symbol;Acc:MGI:1917084] | yes|up |
| ENSMUSG00000037005 | Xpnpep2 | X-prolyl aminopeptidase (aminopeptidase P) 2, membrane-bound [Source:MGI Symbol;Acc:MGI:2180001] | yes|down |
| ENSMUSG00000059791 | Nrm | nurim (nuclear envelope membrane protein) [Source:MGI Symbol;Acc:MGI:2146855] | yes|down |
| ENSMUSG00000040640 | Erc2 | ELKS/RAB6-interacting/CAST family member 2 [Source:MGI Symbol;Acc:MGI:1098749] | yes|down |
| ENSMUSG00000104554 | Gm4610 | predicted gene 4610 [Source:MGI Symbol;Acc:MGI:3782793] | yes|down |
| ENSMUSG00000048007 | Timm8a1 | translocase of inner mitochondrial membrane 8A1 [Source:MGI Symbol;Acc:MGI:1353433] | yes|down |
| ENSMUSG00000090145 | Ugt1a6b | UDP glucuronosyltransferase 1 family, polypeptide A6B [Source:MGI Symbol;Acc:MGI:3580629] | yes|down |
| ENSMUSG00000000884 | Gnb1l | guanine nucleotide binding protein (G protein), beta polypeptide 1-like [Source:MGI Symbol;Acc:MGI:1338057] | yes|down |
| ENSMUSG00000018899 | Irf1 | interferon regulatory factor 1 [Source:MGI Symbol;Acc:MGI:96590] | yes|up |
| ENSMUSG00000063727 | Tnfrsf11b | tumor necrosis factor receptor superfamily, member 11b (osteoprotegerin) [Source:MGI Symbol;Acc:MGI:109587] | yes|up |
| ENSMUSG00000021118 | Plek2 | pleckstrin 2 [Source:MGI Symbol;Acc:MGI:1351466] | yes|down |
| ENSMUSG00000034110 | Kctd7 | potassium channel tetramerisation domain containing 7 [Source:MGI Symbol;Acc:MGI:2442265] | yes|up |
| ENSMUSG00000021336 | Slc17a4 | solute carrier family 17 (sodium phosphate), member 4 [Source:MGI Symbol;Acc:MGI:2442850] | yes|up |
| ENSMUSG00000031098 | Syt8 | synaptotagmin VIII [Source:MGI Symbol;Acc:MGI:1859867] | yes|down |
| ENSMUSG00000015083 | C8g | complement component 8, gamma polypeptide [Source:MGI Symbol;Acc:MGI:88237] | yes|up |
| ENSMUSG00000007050 | Lsm2 | LSM2 homolog, U6 small nuclear RNA and mRNA degradation associated [Source:MGI Symbol;Acc:MGI:90676] | yes|down |
| ENSMUSG00000023830 | Igf2r | insulin-like growth factor 2 receptor [Source:MGI Symbol;Acc:MGI:96435] | yes|up |
| ENSMUSG00000017817 | Jph2 | junctophilin 2 [Source:MGI Symbol;Acc:MGI:1891496] | yes|up |
| ENSMUSG00000061411 | Nol4l | nucleolar protein 4-like [Source:MGI Symbol;Acc:MGI:1918765] | yes|up |
| ENSMUSG00000029368 | Alb | albumin [Source:MGI Symbol;Acc:MGI:87991] | yes|up |
| ENSMUSG00000029369 | Afm | afamin [Source:MGI Symbol;Acc:MGI:2429409] | yes|up |
| ENSMUSG00000022445 | Cyp2d26 | cytochrome P450, family 2, subfamily d, polypeptide 26 [Source:MGI Symbol;Acc:MGI:1923529] | yes|up |
| ENSMUSG00000029363 | Rfc5 | replication factor C (activator 1) 5 [Source:MGI Symbol;Acc:MGI:1919401] | yes|down |
| ENSMUSG00000049719 | Prss46 | protease, serine 46 [Source:MGI Symbol;Acc:MGI:1921556] | yes|up |
| ENSMUSG00000042895 | Abra | actin-binding Rho activating protein [Source:MGI Symbol;Acc:MGI:2444891] | yes|up |
| ENSMUSG00000015880 | Ncapg | non-SMC condensin I complex, subunit G [Source:MGI Symbol;Acc:MGI:1930197] | yes|down |
| ENSMUSG00000023034 | Nr4a1 | nuclear receptor subfamily 4, group A, member 1 [Source:MGI Symbol;Acc:MGI:1352454] | yes|down |
| ENSMUSG00000002104 | Rapsn | receptor-associated protein of the synapse [Source:MGI Symbol;Acc:MGI:99422] | yes|up |
| ENSMUSG00000002107 | Celf2 | CUGBP, Elav-like family member 2 [Source:MGI Symbol;Acc:MGI:1338822] | yes|down |
| ENSMUSG00000002108 | Nr1h3 | nuclear receptor subfamily 1, group H, member 3 [Source:MGI Symbol;Acc:MGI:1352462] | yes|up |
| ENSMUSG00000046179 | E2f8 | E2F transcription factor 8 [Source:MGI Symbol;Acc:MGI:1922038] | yes|down |
| ENSMUSG00000042099 | Kank3 | KN motif and ankyrin repeat domains 3 [Source:MGI Symbol;Acc:MGI:1098615] | yes|down |
| ENSMUSG00000042097 | Zfp239 | zinc finger protein 239 [Source:MGI Symbol;Acc:MGI:1306812] | yes|down |
| ENSMUSG00000028369 | Svep1 | sushi, von Willebrand factor type A, EGF and pentraxin domain containing 1 [Source:MGI Symbol;Acc:MGI:1928849] | yes|up |
| ENSMUSG00000028364 | Tnc | tenascin C [Source:MGI Symbol;Acc:MGI:101922] | yes|up |
| ENSMUSG00000028367 | Txn1 | thioredoxin 1 [Source:MGI Symbol;Acc:MGI:98874] | yes|down |
| ENSMUSG00000008307 | 1700109H08Rik | RIKEN cDNA 1700109H08 gene [Source:MGI Symbol;Acc:MGI:1924286] | yes|up |
| ENSMUSG00000001227 | Sema6b | sema domain, transmembrane domain (TM), and cytoplasmic domain, (semaphorin) 6B [Source:MGI Symbol;Acc:MGI:1202889] | yes|down |
| ENSMUSG00000107768 | Gm36582 | predicted gene, 36582 [Source:MGI Symbol;Acc:MGI:5595741] | yes|down |
| ENSMUSG00000095041 |  |  | yes|up |
| ENSMUSG00000024302 | Dtna | dystrobrevin alpha [Source:MGI Symbol;Acc:MGI:106039] | yes|down |
| ENSMUSG00000020017 | Hal | histidine ammonia lyase [Source:MGI Symbol;Acc:MGI:96010] | yes|up |
| ENSMUSG00000052430 | Bmpr1b | bone morphogenetic protein receptor, type 1B [Source:MGI Symbol;Acc:MGI:107191] | yes|up |
| ENSMUSG00000035686 | Thrsp | thyroid hormone responsive [Source:MGI Symbol;Acc:MGI:109126] | yes|up |
| ENSMUSG00000021492 | F12 | coagulation factor XII (Hageman factor) [Source:MGI Symbol;Acc:MGI:1891012] | yes|up |
| ENSMUSG00000021943 | Gdf10 | growth differentiation factor 10 [Source:MGI Symbol;Acc:MGI:95684] | yes|up |
| ENSMUSG00000032221 | Mns1 | meiosis-specific nuclear structural protein 1 [Source:MGI Symbol;Acc:MGI:107933] | yes|down |
| ENSMUSG00000037638 | Zbtb42 | zinc finger and BTB domain containing 42 [Source:MGI Symbol;Acc:MGI:3644133] | yes|up |
| ENSMUSG00000062510 | Nsl1 | NSL1, MIS12 kinetochore complex component [Source:MGI Symbol;Acc:MGI:2685830] | yes|down |
| ENSMUSG00000067038 | Rps12-ps3 | ribosomal protein S12, pseudogene 3 [Source:MGI Symbol;Acc:MGI:3704503] | yes|down |
| ENSMUSG00000040253 | Gbp7 | guanylate binding protein 7 [Source:MGI Symbol;Acc:MGI:2444421] | yes|up |
| ENSMUSG00000111343 | Gm47175 | predicted gene, 47175 [Source:MGI Symbol;Acc:MGI:6095958] | yes|down |
| ENSMUSG00000101356 | Gm28876 | predicted gene 28876 [Source:MGI Symbol;Acc:MGI:5579582] | yes|up |
| ENSMUSG00000000278 | Scpep1 | serine carboxypeptidase 1 [Source:MGI Symbol;Acc:MGI:1921867] | yes|up |
| ENSMUSG00000005357 | Slc1a6 | solute carrier family 1 (high affinity aspartate/glutamate transporter), member 6 [Source:MGI Symbol;Acc:MGI:1096331] | yes|up |
| ENSMUSG00000097530 | Kansl2-ps | KAT8 regulatory NSL complex subunit 2, pseudogene [Source:MGI Symbol;Acc:MGI:3648525] | yes|up |
| ENSMUSG00000097536 | 2610037D02Rik | RIKEN cDNA 2610037D02 gene [Source:MGI Symbol;Acc:MGI:1917290] | yes|up |
| ENSMUSG00000097537 | 2610020C07Rik | RIKEN cDNA 2610020C07 gene [Source:MGI Symbol;Acc:MGI:1917168] | yes|down |
| ENSMUSG00000056656 | Apol8 | apolipoprotein L 8 [Source:MGI Symbol;Acc:MGI:2444921] | yes|down |
| ENSMUSG00000091625 | Lsm5 | LSM5 homolog, U6 small nuclear RNA and mRNA degradation associated [Source:MGI Symbol;Acc:MGI:1913623] | yes|down |
| ENSMUSG00000090622 | A930033H14Rik | RIKEN cDNA A930033H14 gene [Source:MGI Symbol;Acc:MGI:2444562] | yes|up |
| ENSMUSG00000005681 | Apoa2 | apolipoprotein A-II [Source:MGI Symbol;Acc:MGI:88050] | yes|up |
| ENSMUSG00000005686 | Ampd3 | adenosine monophosphate deaminase 3 [Source:MGI Symbol;Acc:MGI:1096344] | yes|down |
| ENSMUSG00000060613 | Cyp2c70 | cytochrome P450, family 2, subfamily c, polypeptide 70 [Source:MGI Symbol;Acc:MGI:2385878] | yes|up |
| ENSMUSG00000001918 | Slc1a5 | solute carrier family 1 (neutral amino acid transporter), member 5 [Source:MGI Symbol;Acc:MGI:105305] | yes|down |
| ENSMUSG00000110439 | Mup22 | major urinary protein 22 [Source:MGI Symbol;Acc:MGI:5434675] | yes|up |
| ENSMUSG00000038071 | Npy6r | neuropeptide Y receptor Y6 [Source:MGI Symbol;Acc:MGI:1098590] | yes|up |
| ENSMUSG00000038074 | Fkbp14 | FK506 binding protein 14 [Source:MGI Symbol;Acc:MGI:2387639] | yes|up |
| ENSMUSG00000097754 | Ptgs2os2 | prostaglandin-endoperoxide synthase 2, opposite strand 2 [Source:MGI Symbol;Acc:MGI:5477181] | yes|down |
| ENSMUSG00000118642 | AY036118 | cDNA sequence AY036118 [Source:MGI Symbol;Acc:MGI:2158419] | yes|up |
| ENSMUSG00000073514 | Dok6 | docking protein 6 [Source:MGI Symbol;Acc:MGI:3639495] | yes|up |
| ENSMUSG00000112496 | Gm48764 | predicted gene, 48764 [Source:MGI Symbol;Acc:MGI:6098449] | yes|up |
| ENSMUSG00000022146 | Osmr | oncostatin M receptor [Source:MGI Symbol;Acc:MGI:1330819] | yes|up |
| ENSMUSG00000022149 | C9 | complement component 9 [Source:MGI Symbol;Acc:MGI:1098282] | yes|up |
| ENSMUSG00000022148 | Fyb | FYN binding protein [Source:MGI Symbol;Acc:MGI:1346327] | yes|up |
| ENSMUSG00000115149 | 9330188P03Rik | RIKEN cDNA 9330188P03 gene [Source:MGI Symbol;Acc:MGI:2686435] | yes|down |
| ENSMUSG00000051351 | Zfp46 | zinc finger protein 46 [Source:MGI Symbol;Acc:MGI:99192] | yes|up |
| ENSMUSG00000004558 | Ndrg2 | N-myc downstream regulated gene 2 [Source:MGI Symbol;Acc:MGI:1352498] | yes|up |
| ENSMUSG00000051359 | Ncald | neurocalcin delta [Source:MGI Symbol;Acc:MGI:1196326] | yes|up |
| ENSMUSG00000029804 | Herc3 | hect domain and RLD 3 [Source:MGI Symbol;Acc:MGI:1921248] | yes|up |
| ENSMUSG00000022322 | Shcbp1 | Shc SH2-domain binding protein 1 [Source:MGI Symbol;Acc:MGI:1338802] | yes|down |
| ENSMUSG00000022321 | Cdh10 | cadherin 10 [Source:MGI Symbol;Acc:MGI:107436] | yes|up |
| ENSMUSG00000066755 | Tnfsf18 | tumor necrosis factor (ligand) superfamily, member 18 [Source:MGI Symbol;Acc:MGI:2673064] | yes|up |
| ENSMUSG00000031137 | Fgf13 | fibroblast growth factor 13 [Source:MGI Symbol;Acc:MGI:109178] | yes|up |
| ENSMUSG00000095677 | Dynlt1f | dynein light chain Tctex-type 1F [Source:MGI Symbol;Acc:MGI:3780996] | yes|down |
| ENSMUSG00000075588 | Hoxb2 | homeobox B2 [Source:MGI Symbol;Acc:MGI:96183] | yes|up |
| ENSMUSG00000101698 | Gm29562 | predicted gene 29562 [Source:MGI Symbol;Acc:MGI:5580268] | yes|up |
| ENSMUSG00000025498 | Irf7 | interferon regulatory factor 7 [Source:MGI Symbol;Acc:MGI:1859212] | yes|up |
| ENSMUSG00000048636 | A730049H05Rik | RIKEN cDNA A730049H05 gene [Source:MGI Symbol;Acc:MGI:1921766] | yes|up |
| ENSMUSG00000058952 | Cfi | complement component factor i [Source:MGI Symbol;Acc:MGI:105937] | yes|up |
| ENSMUSG00000029771 | Irf5 | interferon regulatory factor 5 [Source:MGI Symbol;Acc:MGI:1350924] | yes|down |
| ENSMUSG00000047963 | Stbd1 | starch binding domain 1 [Source:MGI Symbol;Acc:MGI:1261768] | yes|up |
| ENSMUSG00000028179 | Cth | cystathionase (cystathionine gamma-lyase) [Source:MGI Symbol;Acc:MGI:1339968] | yes|up |
| ENSMUSG00000028173 | Wls | wntless WNT ligand secretion mediator [Source:MGI Symbol;Acc:MGI:1915401] | yes|up |
| ENSMUSG00000028175 | Depdc1a | DEP domain containing 1a [Source:MGI Symbol;Acc:MGI:1923381] | yes|down |
| ENSMUSG00000028176 | Lrrc7 | leucine rich repeat containing 7 [Source:MGI Symbol;Acc:MGI:2676665] | yes|up |
| ENSMUSG00000114433 | Gm48488 | predicted gene, 48488 [Source:MGI Symbol;Acc:MGI:6098010] | yes|up |
| ENSMUSG00000074768 | Bhmt | betaine-homocysteine methyltransferase [Source:MGI Symbol;Acc:MGI:1339972] | yes|up |
| ENSMUSG00000016494 | Cd34 | CD34 antigen [Source:MGI Symbol;Acc:MGI:88329] | yes|down |
| ENSMUSG00000020648 | Dus4l | dihydrouridine synthase 4-like (S. cerevisiae) [Source:MGI Symbol;Acc:MGI:1919166] | yes|down |
| ENSMUSG00000069208 | Zfp825 | zinc finger protein 825 [Source:MGI Symbol;Acc:MGI:2385315] | yes|down |
| ENSMUSG00000112640 | Gm32687 | predicted gene, 32687 [Source:MGI Symbol;Acc:MGI:5591846] | yes|down |
| ENSMUSG00000046610 | Oacyl | O-acyltransferase like [Source:MGI Symbol;Acc:MGI:2442915] | yes|up |
| ENSMUSG00000079277 | Hoxd3 | homeobox D3 [Source:MGI Symbol;Acc:MGI:96207] | yes|up |
| ENSMUSG00000021552 | Gkap1 | G kinase anchoring protein 1 [Source:MGI Symbol;Acc:MGI:1891694] | yes|down |
| ENSMUSG00000062727 | H2bc12 | H2B clustered histone 12 [Source:MGI Symbol;Acc:MGI:2448399] | yes|up |
| ENSMUSG00000082860 | Gm13204 | predicted gene 13204 [Source:MGI Symbol;Acc:MGI:3651444] | yes|down |
| ENSMUSG00000042286 | Stab1 | stabilin 1 [Source:MGI Symbol;Acc:MGI:2178742] | yes|up |
| ENSMUSG00000042284 | Itga1 | integrin alpha 1 [Source:MGI Symbol;Acc:MGI:96599] | yes|up |
| ENSMUSG00000117165 | Gm49832 | predicted gene, 49832 [Source:MGI Symbol;Acc:MGI:6270499] | yes|down |
| ENSMUSG00000041064 | Pif1 | PIF1 5'-to-3' DNA helicase [Source:MGI Symbol;Acc:MGI:2143057] | yes|down |
| ENSMUSG00000018189 | Uchl5 | ubiquitin carboxyl-terminal esterase L5 [Source:MGI Symbol;Acc:MGI:1914848] | yes|down |
| ENSMUSG00000039934 | Gsap | gamma-secretase activating protein [Source:MGI Symbol;Acc:MGI:2442259] | yes|down |
| ENSMUSG00000036223 | Ska1 | spindle and kinetochore associated complex subunit 1 [Source:MGI Symbol;Acc:MGI:1913718] | yes|down |
| ENSMUSG00000075012 | Fjx1 | four jointed box 1 [Source:MGI Symbol;Acc:MGI:1341907] | yes|up |
| ENSMUSG00000039158 | Akna | AT-hook transcription factor [Source:MGI Symbol;Acc:MGI:2140340] | yes|up |
| ENSMUSG00000041390 | Mdfic | MyoD family inhibitor domain containing [Source:MGI Symbol;Acc:MGI:104611] | yes|up |
| ENSMUSG00000018986 | Slfn3 | schlafen 3 [Source:MGI Symbol;Acc:MGI:1329005] | yes|down |
| ENSMUSG00000058799 | Nap1l1 | nucleosome assembly protein 1-like 1 [Source:MGI Symbol;Acc:MGI:1855693] | yes|down |
| ENSMUSG00000024691 | Fam111a | family with sequence similarity 111, member A [Source:MGI Symbol;Acc:MGI:1915508] | yes|down |
| ENSMUSG00000038685 | Rtel1 | regulator of telomere elongation helicase 1 [Source:MGI Symbol;Acc:MGI:2139369] | yes|down |
| ENSMUSG00000001661 | Hoxc6 | homeobox C6 [Source:MGI Symbol;Acc:MGI:96197] | yes|up |
| ENSMUSG00000001663 | Gstt1 | glutathione S-transferase, theta 1 [Source:MGI Symbol;Acc:MGI:107379] | yes|up |
| ENSMUSG00000037797 | Adh4 | alcohol dehydrogenase 4 (class II), pi polypeptide [Source:MGI Symbol;Acc:MGI:1349472] | yes|up |
| ENSMUSG00000037798 | Mat1a | methionine adenosyltransferase I, alpha [Source:MGI Symbol;Acc:MGI:88017] | yes|up |
| ENSMUSG00000027520 | Zdbf2 | zinc finger, DBF-type containing 2 [Source:MGI Symbol;Acc:MGI:1921134] | yes|up |
| ENSMUSG00000020974 | Pole2 | polymerase (DNA directed), epsilon 2 (p59 subunit) [Source:MGI Symbol;Acc:MGI:1197514] | yes|down |
| ENSMUSG00000020205 | Phlda1 | pleckstrin homology like domain, family A, member 1 [Source:MGI Symbol;Acc:MGI:1096880] | yes|down |
| ENSMUSG00000109378 | Gm49396 | predicted gene, 49396 [Source:MGI Symbol;Acc:MGI:6121629] | yes|up |
| ENSMUSG00000037572 | Wdhd1 | WD repeat and HMG-box DNA binding protein 1 [Source:MGI Symbol;Acc:MGI:2443514] | yes|down |
| ENSMUSG00000030074 | Gxylt2 | glucoside xylosyltransferase 2 [Source:MGI Symbol;Acc:MGI:2682940] | yes|up |
| ENSMUSG00000030077 | Chl1 | cell adhesion molecule L1-like [Source:MGI Symbol;Acc:MGI:1098266] | yes|up |
| ENSMUSG00000040624 | Plekhg1 | pleckstrin homology domain containing, family G (with RhoGef domain) member 1 [Source:MGI Symbol;Acc:MGI:2676551] | yes|up |
| ENSMUSG00000111133 | Gm5831 | predicted gene 5831 [Source:MGI Symbol;Acc:MGI:3779529] | yes|down |
| ENSMUSG00000041936 | Agrn | agrin [Source:MGI Symbol;Acc:MGI:87961] | yes|up |
| ENSMUSG00000026121 | Sema4c | sema domain, immunoglobulin domain (Ig), transmembrane domain (TM) and short cytoplasmic domain, (semaphorin) 4C [Source:MGI Symbol;Acc:MGI:109252] | yes|up |
| ENSMUSG00000071656 | Lrrn4cl | LRRN4 C-terminal like [Source:MGI Symbol;Acc:MGI:1916102] | yes|up |
| ENSMUSG00000034171 | Faah | fatty acid amide hydrolase [Source:MGI Symbol;Acc:MGI:109609] | yes|up |
| ENSMUSG00000032261 | Sh3bgrl2 | SH3 domain binding glutamic acid-rich protein like 2 [Source:MGI Symbol;Acc:MGI:1915350] | yes|down |
| ENSMUSG00000034173 | Chchd2l | coiled-coil-helix-coiled-coil-helix domain containing 2-like [Source:MGI Symbol;Acc:MGI:1919220] | yes|up |
| ENSMUSG00000021314 | Amph | amphiphysin [Source:MGI Symbol;Acc:MGI:103574] | yes|down |
| ENSMUSG00000021319 | Sfrp4 | secreted frizzled-related protein 4 [Source:MGI Symbol;Acc:MGI:892010] | yes|up |
| ENSMUSG00000029163 | Emilin1 | elastin microfibril interfacer 1 [Source:MGI Symbol;Acc:MGI:1926189] | yes|up |
| ENSMUSG00000060807 | Serpina6 | serine (or cysteine) peptidase inhibitor, clade A, member 6 [Source:MGI Symbol;Acc:MGI:88278] | yes|up |
| ENSMUSG00000023057 | Fabp2 | fatty acid binding protein 2, intestinal [Source:MGI Symbol;Acc:MGI:95478] | yes|up |
| ENSMUSG00000007038 | Neu1 | neuraminidase 1 [Source:MGI Symbol;Acc:MGI:97305] | yes|up |
| ENSMUSG00000007035 | Msh5 | mutS homolog 5 [Source:MGI Symbol;Acc:MGI:1329021] | yes|down |
| ENSMUSG00000027513 | Pck1 | phosphoenolpyruvate carboxykinase 1, cytosolic [Source:MGI Symbol;Acc:MGI:97501] | yes|up |
| ENSMUSG00000036825 | Ssx2ip | synovial sarcoma, X 2 interacting protein [Source:MGI Symbol;Acc:MGI:2139150] | yes|down |
| ENSMUSG00000042873 | Lhfpl4 | lipoma HMGIC fusion partner-like protein 4 [Source:MGI Symbol;Acc:MGI:3057108] | yes|down |
| ENSMUSG00000110669 | Gm10060 | predicted gene 10060 [Source:MGI Symbol;Acc:MGI:3710638] | yes|up |
| ENSMUSG00000028693 | Nasp | nuclear autoantigenic sperm protein (histone-binding) [Source:MGI Symbol;Acc:MGI:1355328] | yes|down |
| ENSMUSG00000034799 | Unc13a | unc-13 homolog A [Source:MGI Symbol;Acc:MGI:3051532] | yes|up |
| ENSMUSG00000022464 | Slc38a4 | solute carrier family 38, member 4 [Source:MGI Symbol;Acc:MGI:1916604] | yes|down |
| ENSMUSG00000021136 | Smoc1 | SPARC related modular calcium binding 1 [Source:MGI Symbol;Acc:MGI:1929878] | yes|up |
| ENSMUSG00000110256 | Gm45412 | predicted gene 45412 [Source:MGI Symbol;Acc:MGI:5791248] | yes|up |
| ENSMUSG00000075391 | Glo1-ps | glyoxalase 1, pseudogene [Source:MGI Symbol;Acc:MGI:3649615] | yes|down |
| ENSMUSG00000075394 | Hoxc4 | homeobox C4 [Source:MGI Symbol;Acc:MGI:96195] | yes|up |
| ENSMUSG00000028435 | Aqp3 | aquaporin 3 [Source:MGI Symbol;Acc:MGI:1333777] | yes|up |
| ENSMUSG00000052180 | Serpinb6c | serine (or cysteine) peptidase inhibitor, clade B, member 6c [Source:MGI Symbol;Acc:MGI:2145481] | yes|up |
| ENSMUSG00000052632 | Asap2 | ArfGAP with SH3 domain, ankyrin repeat and PH domain 2 [Source:MGI Symbol;Acc:MGI:2685438] | yes|down |
| ENSMUSG00000051984 | Sec31b | Sec31 homolog B (S. cerevisiae) [Source:MGI Symbol;Acc:MGI:2685187] | yes|up |
| ENSMUSG00000032028 | Nxpe2 | neurexophilin and PC-esterase domain family, member 2 [Source:MGI Symbol;Acc:MGI:1925502] | yes|up |
| ENSMUSG00000032020 | Ubash3b | ubiquitin associated and SH3 domain containing, B [Source:MGI Symbol;Acc:MGI:1920078] | yes|down |
| ENSMUSG00000032796 | Lama1 | laminin, alpha 1 [Source:MGI Symbol;Acc:MGI:99892] | yes|up |
| ENSMUSG00000054675 | Tmem119 | transmembrane protein 119 [Source:MGI Symbol;Acc:MGI:2385228] | yes|up |
| ENSMUSG00000087370 | Tmem170b | transmembrane protein 170B [Source:MGI Symbol;Acc:MGI:3647046] | yes|up |
| ENSMUSG00000054589 | Gm9949 | predicted gene 9949 [Source:MGI Symbol;Acc:MGI:3647947] | yes|down |
| ENSMUSG00000087484 | 2900089D17Rik | RIKEN cDNA 2900089D17 gene [Source:MGI Symbol;Acc:MGI:1914527] | yes|up |
| ENSMUSG00000002870 | Mcm2 | minichromosome maintenance complex component 2 [Source:MGI Symbol;Acc:MGI:105380] | yes|down |
| ENSMUSG00000021965 | Ska3 | spindle and kinetochore associated complex subunit 3 [Source:MGI Symbol;Acc:MGI:3041235] | yes|down |
| ENSMUSG00000060240 | Cend1 | cell cycle exit and neuronal differentiation 1 [Source:MGI Symbol;Acc:MGI:1929898] | yes|down |
| ENSMUSG00000113476 | Gm48309 | predicted gene, 48309 [Source:MGI Symbol;Acc:MGI:6097758] | yes|up |
| ENSMUSG00000049134 | Nrap | nebulin-related anchoring protein [Source:MGI Symbol;Acc:MGI:1098765] | yes|up |
| ENSMUSG00000024990 | Rbp4 | retinol binding protein 4, plasma [Source:MGI Symbol;Acc:MGI:97879] | yes|up |
| ENSMUSG00000086427 | Hoxa11os | homeobox A11, opposite strand [Source:MGI Symbol;Acc:MGI:107208] | yes|down |
| ENSMUSG00000030793 | Pycard | PYD and CARD domain containing [Source:MGI Symbol;Acc:MGI:1931465] | yes|down |
| ENSMUSG00000059363 | Fxn | frataxin [Source:MGI Symbol;Acc:MGI:1096879] | yes|down |
| ENSMUSG00000041729 | Coro2b | coronin, actin binding protein, 2B [Source:MGI Symbol;Acc:MGI:2444283] | yes|up |
| ENSMUSG00000041237 | Pklr | pyruvate kinase liver and red blood cell [Source:MGI Symbol;Acc:MGI:97604] | yes|up |
| ENSMUSG00000041235 | Chd7 | chromodomain helicase DNA binding protein 7 [Source:MGI Symbol;Acc:MGI:2444748] | yes|up |
| ENSMUSG00000050359 | Sprr1a | small proline-rich protein 1A [Source:MGI Symbol;Acc:MGI:106660] | yes|down |
| ENSMUSG00000037211 | Spry1 | sprouty RTK signaling antagonist 1 [Source:MGI Symbol;Acc:MGI:1345139] | yes|up |
| ENSMUSG00000030532 | Hddc3 | HD domain containing 3 [Source:MGI Symbol;Acc:MGI:1915945] | yes|down |
| ENSMUSG00000118661 | Muc6 | mucin 6, gastric [Source:MGI Symbol;Acc:MGI:2663233] | yes|down |
| ENSMUSG00000030249 | Abcc9 | ATP-binding cassette, sub-family C (CFTR/MRP), member 9 [Source:MGI Symbol;Acc:MGI:1352630] | yes|up |
| ENSMUSG00000027750 | Postn | periostin, osteoblast specific factor [Source:MGI Symbol;Acc:MGI:1926321] | yes|up |
| ENSMUSG00000070713 | Hmgn2-ps | high mobility group nucleosomal binding domain 2, pseudogene [Source:MGI Symbol;Acc:MGI:3704312] | yes|down |
| ENSMUSG00000027752 | Exosc8 | exosome component 8 [Source:MGI Symbol;Acc:MGI:1916889] | yes|down |
| ENSMUSG00000073530 | Pappa2 | pappalysin 2 [Source:MGI Symbol;Acc:MGI:3051647] | yes|up |
| ENSMUSG00000022123 | Scel | sciellin [Source:MGI Symbol;Acc:MGI:1891228] | yes|up |
| ENSMUSG00000022122 | Ednrb | endothelin receptor type B [Source:MGI Symbol;Acc:MGI:102720] | yes|up |
| ENSMUSG00000066363 | Serpina3f | serine (or cysteine) peptidase inhibitor, clade A, member 3F [Source:MGI Symbol;Acc:MGI:2182838] | yes|up |
| ENSMUSG00000037185 | Krt80 | keratin 80 [Source:MGI Symbol;Acc:MGI:1921377] | yes|up |
| ENSMUSG00000066366 | Serpina1a | serine (or cysteine) peptidase inhibitor, clade A, member 1A [Source:MGI Symbol;Acc:MGI:891971] | yes|up |
| ENSMUSG00000055723 | Rras2 | related RAS viral (r-ras) oncogene 2 [Source:MGI Symbol;Acc:MGI:1914172] | yes|down |
| ENSMUSG00000072620 | Slfn2 | schlafen 2 [Source:MGI Symbol;Acc:MGI:1313258] | yes|up |
| ENSMUSG00000056185 | Snx32 | sorting nexin 32 [Source:MGI Symbol;Acc:MGI:2444704] | yes|up |
| ENSMUSG00000050730 | Arhgap42 | Rho GTPase activating protein 42 [Source:MGI Symbol;Acc:MGI:1918794] | yes|up |
| ENSMUSG00000031112 | Stk26 | serine/threonine kinase 26 [Source:MGI Symbol;Acc:MGI:1917665] | yes|up |
| ENSMUSG00000031119 | Gpc4 | glypican 4 [Source:MGI Symbol;Acc:MGI:104902] | yes|up |
| ENSMUSG00000025479 | Cyp2e1 | cytochrome P450, family 2, subfamily e, polypeptide 1 [Source:MGI Symbol;Acc:MGI:88607] | yes|up |
| ENSMUSG00000025473 | Adam8 | a disintegrin and metallopeptidase domain 8 [Source:MGI Symbol;Acc:MGI:107825] | yes|down |
| ENSMUSG00000025477 | Inpp5a | inositol polyphosphate-5-phosphatase A [Source:MGI Symbol;Acc:MGI:2686961] | yes|up |
| ENSMUSG00000073394 | Runx2os1 | runt related transcription factor 2, opposite strand 1 [Source:MGI Symbol;Acc:MGI:3641707] | yes|down |
| ENSMUSG00000039328 | Rnf122 | ring finger protein 122 [Source:MGI Symbol;Acc:MGI:1916117] | yes|up |
| ENSMUSG00000109005 | Gm45221 | predicted gene 45221 [Source:MGI Symbol;Acc:MGI:5753797] | yes|down |
| ENSMUSG00000029755 | Dlx5 | distal-less homeobox 5 [Source:MGI Symbol;Acc:MGI:101926] | yes|up |
| ENSMUSG00000121464 |  | novel transcript | yes|down |
| ENSMUSG00000047986 | Palm3 | paralemmin 3 [Source:MGI Symbol;Acc:MGI:1921587] | yes|up |
| ENSMUSG00000062093 | Gm10110 | predicted gene 10110 [Source:MGI Symbol;Acc:MGI:3641718] | yes|down |
| ENSMUSG00000025610 | Map3k7cl | Map3k7 C-terminal like [Source:MGI Symbol;Acc:MGI:2446584] | yes|up |
| ENSMUSG00000021109 | Hif1a | hypoxia inducible factor 1, alpha subunit [Source:MGI Symbol;Acc:MGI:106918] | yes|up |
| ENSMUSG00000034329 | Brip1 | BRCA1 interacting protein C-terminal helicase 1 [Source:MGI Symbol;Acc:MGI:2442836] | yes|down |
| ENSMUSG00000032498 | Mlh1 | mutL homolog 1 [Source:MGI Symbol;Acc:MGI:101938] | yes|down |
| ENSMUSG00000032492 | Pth1r | parathyroid hormone 1 receptor [Source:MGI Symbol;Acc:MGI:97801] | yes|up |
| ENSMUSG00000068246 | Apol9b | apolipoprotein L 9b [Source:MGI Symbol;Acc:MGI:1919148] | yes|up |
| ENSMUSG00000068245 | Phf11d | PHD finger protein 11D [Source:MGI Symbol;Acc:MGI:1277133] | yes|up |
| ENSMUSG00000042118 | Bhmt2 | betaine-homocysteine methyltransferase 2 [Source:MGI Symbol;Acc:MGI:1891379] | yes|up |
| ENSMUSG00000103898 | Gm30238 | predicted gene, 30238 [Source:MGI Symbol;Acc:MGI:5589397] | yes|up |
| ENSMUSG00000103897 | Pcdhga8 | protocadherin gamma subfamily A, 8 [Source:MGI Symbol;Acc:MGI:1935221] | yes|up |
| ENSMUSG00000042115 | Klhdc8a | kelch domain containing 8A [Source:MGI Symbol;Acc:MGI:2442630] | yes|down |
| ENSMUSG00000050910 | Cdr2l | cerebellar degeneration-related protein 2-like [Source:MGI Symbol;Acc:MGI:2684867] | yes|down |
| ENSMUSG00000024056 | Ndc80 | NDC80 kinetochore complex component [Source:MGI Symbol;Acc:MGI:1914302] | yes|down |
| ENSMUSG00000021575 | Ahrr | aryl-hydrocarbon receptor repressor [Source:MGI Symbol;Acc:MGI:1333776] | yes|down |
| ENSMUSG00000024053 | Emilin2 | elastin microfibril interfacer 2 [Source:MGI Symbol;Acc:MGI:2389136] | yes|down |
| ENSMUSG00000068794 | Col28a1 | collagen, type XXVIII, alpha 1 [Source:MGI Symbol;Acc:MGI:2685312] | yes|up |
| ENSMUSG00000045589 | Frrs1l | ferric-chelate reductase 1 like [Source:MGI Symbol;Acc:MGI:2442704] | yes|down |
| ENSMUSG00000053469 | Tg | thyroglobulin [Source:MGI Symbol;Acc:MGI:98733] | yes|up |
| ENSMUSG00000022906 | Parp9 | poly (ADP-ribose) polymerase family, member 9 [Source:MGI Symbol;Acc:MGI:1933117] | yes|up |
| ENSMUSG00000039910 | Cited2 | Cbp/p300-interacting transactivator, with Glu/Asp-rich carboxy-terminal domain, 2 [Source:MGI Symbol;Acc:MGI:1306784] | yes|down |
| ENSMUSG00000039911 | Spsb1 | splA/ryanodine receptor domain and SOCS box containing 1 [Source:MGI Symbol;Acc:MGI:1921896] | yes|up |
| ENSMUSG00000044674 | Fzd1 | frizzled class receptor 1 [Source:MGI Symbol;Acc:MGI:1196625] | yes|up |
| ENSMUSG00000078700 | D030028A08Rik | RIKEN cDNA D030028A08 gene [Source:MGI Symbol;Acc:MGI:2441931] | yes|up |
| ENSMUSG00000078706 | Gm53 | predicted gene 53 [Source:MGI Symbol;Acc:MGI:2684899] | yes|up |
| ENSMUSG00000027508 | Pag1 | phosphoprotein associated with glycosphingolipid microdomains 1 [Source:MGI Symbol;Acc:MGI:2443160] | yes|up |
| ENSMUSG00000028121 | Bcar3 | breast cancer anti-estrogen resistance 3 [Source:MGI Symbol;Acc:MGI:1352501] | yes|down |
| ENSMUSG00000033453 | Adamts15 | a disintegrin-like and metallopeptidase (reprolysin type) with thrombospondin type 1 motif, 15 [Source:MGI Symbol;Acc:MGI:2449569] | yes|up |
| ENSMUSG00000033450 | Tagap | T cell activation Rho GTPase activating protein [Source:MGI Symbol;Acc:MGI:3615484] | yes|down |
| ENSMUSG00000113640 | Adat3 | adenosine deaminase, tRNA-specific 3 [Source:MGI Symbol;Acc:MGI:1924344] | yes|down |
| ENSMUSG00000004043 | Stat5a | signal transducer and activator of transcription 5A [Source:MGI Symbol;Acc:MGI:103036] | yes|down |
| ENSMUSG00000040605 | Bace2 | beta-site APP-cleaving enzyme 2 [Source:MGI Symbol;Acc:MGI:1860440] | yes|up |
| ENSMUSG00000040606 | Kazn | kazrin, periplakin interacting protein [Source:MGI Symbol;Acc:MGI:1918779] | yes|up |
| ENSMUSG00000041912 | Tdrkh | tudor and KH domain containing protein [Source:MGI Symbol;Acc:MGI:1919884] | yes|down |
| ENSMUSG00000041911 | Dlx1 | distal-less homeobox 1 [Source:MGI Symbol;Acc:MGI:94901] | yes|down |
| ENSMUSG00000071633 | Gm4952 | predicted gene 4952 [Source:MGI Symbol;Acc:MGI:3643569] | yes|up |
| ENSMUSG00000071632 | 2510002D24Rik | RIKEN cDNA 2510002D24 gene [Source:MGI Symbol;Acc:MGI:1919557] | yes|down |
| ENSMUSG00000062300 | Nectin2 | nectin cell adhesion molecule 2 [Source:MGI Symbol;Acc:MGI:97822] | yes|up |
| ENSMUSG00000026100 | Mstn | myostatin [Source:MGI Symbol;Acc:MGI:95691] | yes|up |
| ENSMUSG00000026104 | Stat1 | signal transducer and activator of transcription 1 [Source:MGI Symbol;Acc:MGI:103063] | yes|up |
| ENSMUSG00000026109 | Tmeff2 | transmembrane protein with EGF-like and two follistatin-like domains 2 [Source:MGI Symbol;Acc:MGI:1861735] | yes|down |
| ENSMUSG00000107000 | Gm43481 | predicted gene 43481 [Source:MGI Symbol;Acc:MGI:5663618] | yes|up |
| ENSMUSG00000094066 | Fam205a2 | family with sequence similarity 205, member A2 [Source:MGI Symbol;Acc:MGI:3701946] | yes|up |
| ENSMUSG00000074577 | Ripor3 | RIPOR family member 3 [Source:MGI Symbol;Acc:MGI:1916803] | yes|down |
| ENSMUSG00000021373 | Cap2 | CAP, adenylate cyclase-associated protein, 2 (yeast) [Source:MGI Symbol;Acc:MGI:1914502] | yes|up |
| ENSMUSG00000026365 | Cfh | complement component factor h [Source:MGI Symbol;Acc:MGI:88385] | yes|up |
| ENSMUSG00000062248 | Cks2 | CDC28 protein kinase regulatory subunit 2 [Source:MGI Symbol;Acc:MGI:1913447] | yes|down |
| ENSMUSG00000061132 | Blnk | B cell linker [Source:MGI Symbol;Acc:MGI:96878] | yes|down |
| ENSMUSG00000067367 | Lyar | Ly1 antibody reactive clone [Source:MGI Symbol;Acc:MGI:107470] | yes|down |
| ENSMUSG00000026368 | F13b | coagulation factor XIII, beta subunit [Source:MGI Symbol;Acc:MGI:88379] | yes|up |
| ENSMUSG00000085183 | Wincr1 | WNT induced non-coding RNA 1 [Source:MGI Symbol;Acc:MGI:3649757] | yes|down |
| ENSMUSG00000085189 | Gm11963 | predicted gene 11963 [Source:MGI Symbol;Acc:MGI:3652248] | yes|down |
| ENSMUSG00000046352 | Gjb2 | gap junction protein, beta 2 [Source:MGI Symbol;Acc:MGI:95720] | yes|up |
| ENSMUSG00000029102 | Hgfac | hepatocyte growth factor activator [Source:MGI Symbol;Acc:MGI:1859281] | yes|up |
| ENSMUSG00000020429 | Igfbp1 | insulin-like growth factor binding protein 1 [Source:MGI Symbol;Acc:MGI:96436] | yes|up |
| ENSMUSG00000057396 | Zfp759 | zinc finger protein 759 [Source:MGI Symbol;Acc:MGI:2446280] | yes|up |
| ENSMUSG00000020427 | Igfbp3 | insulin-like growth factor binding protein 3 [Source:MGI Symbol;Acc:MGI:96438] | yes|up |
| ENSMUSG00000083672 | Kpna2-ps | Kpna2 retrotransposed pseudogene [Source:MGI Symbol;Acc:MGI:3647335] | yes|up |
| ENSMUSG00000022489 | Pde1b | phosphodiesterase 1B, Ca2+-calmodulin dependent [Source:MGI Symbol;Acc:MGI:97523] | yes|up |
| ENSMUSG00000022485 | Hoxc5 | homeobox C5 [Source:MGI Symbol;Acc:MGI:96196] | yes|up |
| ENSMUSG00000035208 | Slfn8 | schlafen 8 [Source:MGI Symbol;Acc:MGI:2672859] | yes|up |
| ENSMUSG00000120826 |  | novel transcript, antisense to Hexim2 | yes|up |
| ENSMUSG00000120796 |  | novel transcript, antisense to Uqcrc1 | yes|up |
| ENSMUSG00000062937 | Mtap | methylthioadenosine phosphorylase [Source:MGI Symbol;Acc:MGI:1914152] | yes|down |
| ENSMUSG00000064032 | Gm10143 | predicted gene 10143 [Source:MGI Symbol;Acc:MGI:3704492] | yes|down |
| ENSMUSG00000025134 | Alyref | Aly/REF export factor [Source:MGI Symbol;Acc:MGI:1341044] | yes|down |
| ENSMUSG00000004040 | Stat3 | signal transducer and activator of transcription 3 [Source:MGI Symbol;Acc:MGI:103038] | yes|up |
| ENSMUSG00000102555 | 6430511E19Rik | RIKEN cDNA 6430511E19 gene [Source:MGI Symbol;Acc:MGI:2443259] | yes|up |
| ENSMUSG00000107724 | Gm16042 | predicted gene 16042 [Source:MGI Symbol;Acc:MGI:3801935] | yes|down |
| ENSMUSG00000016024 | Lbp | lipopolysaccharide binding protein [Source:MGI Symbol;Acc:MGI:1098776] | yes|up |
| ENSMUSG00000106415 | Gm42893 | predicted gene 42893 [Source:MGI Symbol;Acc:MGI:5663030] | yes|down |
| ENSMUSG00000063683 | Glyat | glycine-N-acyltransferase [Source:MGI Symbol;Acc:MGI:2147502] | yes|down |
| ENSMUSG00000023990 | Tfeb | transcription factor EB [Source:MGI Symbol;Acc:MGI:103270] | yes|up |
| ENSMUSG00000107092 | Gm7993 | predicted gene 7993 [Source:MGI Symbol;Acc:MGI:3647399] | yes|down |
| ENSMUSG00000084911 | Gm16185 | predicted gene 16185 [Source:MGI Symbol;Acc:MGI:3802169] | yes|up |
| ENSMUSG00000061013 | Mkx | mohawk homeobox [Source:MGI Symbol;Acc:MGI:2687286] | yes|up |
| ENSMUSG00000057967 | Fgf18 | fibroblast growth factor 18 [Source:MGI Symbol;Acc:MGI:1277980] | yes|up |
| ENSMUSG00000116606 | Gm10479 | predicted gene 10479 [Source:MGI Symbol;Acc:MGI:3704385] | yes|up |
| ENSMUSG00000021908 | Ncoa4-ps | nuclear receptor coactivator 4, pseudogene [Source:MGI Symbol;Acc:MGI:3648259] | yes|up |
| ENSMUSG00000078931 | Pdf | peptide deformylase (mitochondrial) [Source:MGI Symbol;Acc:MGI:1915273] | yes|down |
| ENSMUSG00000010358 | Ifi35 | interferon-induced protein 35 [Source:MGI Symbol;Acc:MGI:1917360] | yes|up |
| ENSMUSG00000033207 | Mamdc2 | MAM domain containing 2 [Source:MGI Symbol;Acc:MGI:1918988] | yes|up |
| ENSMUSG00000053702 | Nebl | nebulette [Source:MGI Symbol;Acc:MGI:1921353] | yes|down |
| ENSMUSG00000033209 | Ttc28 | tetratricopeptide repeat domain 28 [Source:MGI Symbol;Acc:MGI:2140873] | yes|up |
| ENSMUSG00000097327 | E030030I06Rik | RIKEN cDNA E030030I06 gene [Source:MGI Symbol;Acc:MGI:2442914] | yes|up |
| ENSMUSG00000049115 | Agtr1a | angiotensin II receptor, type 1a [Source:MGI Symbol;Acc:MGI:87964] | yes|up |
| ENSMUSG00000032911 | Cspg4 | chondroitin sulfate proteoglycan 4 [Source:MGI Symbol;Acc:MGI:2153093] | yes|up |
| ENSMUSG00000049233 | Apoo-ps | apolipoprotein O, pseudogene [Source:MGI Symbol;Acc:MGI:3649039] | yes|down |
| ENSMUSG00000038522 | Mfsd4b1 | major facilitator superfamily domain containing 4B1 [Source:MGI Symbol;Acc:MGI:2143575] | yes|up |
| ENSMUSG00000038521 | C1s1 | complement component 1, s subcomponent 1 [Source:MGI Symbol;Acc:MGI:1355312] | yes|up |
| ENSMUSG00000038526 | Car14 | carbonic anhydrase 14 [Source:MGI Symbol;Acc:MGI:1344341] | yes|up |
| ENSMUSG00000097572 | Gm26797 | predicted gene, 26797 [Source:MGI Symbol;Acc:MGI:5477291] | yes|up |
| ENSMUSG00000059430 | Actg2 | actin, gamma 2, smooth muscle, enteric [Source:MGI Symbol;Acc:MGI:104589] | yes|up |
| ENSMUSG00000059434 | Gckr | glucokinase regulatory protein [Source:MGI Symbol;Acc:MGI:1096345] | yes|up |
| ENSMUSG00000027199 | Gatm | glycine amidinotransferase (L-arginine:glycine amidinotransferase) [Source:MGI Symbol;Acc:MGI:1914342] | yes|down |
| ENSMUSG00000036585 | Fgf1 | fibroblast growth factor 1 [Source:MGI Symbol;Acc:MGI:95515] | yes|up |
| ENSMUSG00000018821 | Avpi1 | arginine vasopressin-induced 1 [Source:MGI Symbol;Acc:MGI:1916784] | yes|down |
| ENSMUSG00000041741 | Pde3a | phosphodiesterase 3A, cGMP inhibited [Source:MGI Symbol;Acc:MGI:1860764] | yes|up |
| ENSMUSG00000041219 | Arhgap11a | Rho GTPase activating protein 11A [Source:MGI Symbol;Acc:MGI:2444300] | yes|down |
| ENSMUSG00000105258 | Gm40038 | predicted gene, 40038 [Source:MGI Symbol;Acc:MGI:5622923] | yes|up |
| ENSMUSG00000047747 | Rnf150 | ring finger protein 150 [Source:MGI Symbol;Acc:MGI:2443860] | yes|up |
| ENSMUSG00000030515 | Tarsl2 | threonyl-tRNA synthetase-like 2 [Source:MGI Symbol;Acc:MGI:2444486] | yes|down |
| ENSMUSG00000027994 | Mcub | mitochondrial calcium uniporter dominant negative beta subunit [Source:MGI Symbol;Acc:MGI:1914065] | yes|down |
| ENSMUSG00000022103 | Gfra2 | glial cell line derived neurotrophic factor family receptor alpha 2 [Source:MGI Symbol;Acc:MGI:1195462] | yes|down |
| ENSMUSG00000004267 | Eno2 | enolase 2, gamma neuronal [Source:MGI Symbol;Acc:MGI:95394] | yes|up |
| ENSMUSG00000058914 | C1qtnf3 | C1q and tumor necrosis factor related protein 3 [Source:MGI Symbol;Acc:MGI:1932136] | yes|up |
| ENSMUSG00000112808 | Gm4739 | predicted gene 4739 [Source:MGI Symbol;Acc:MGI:3642984] | yes|down |
| ENSMUSG00000031173 | Otc | ornithine transcarbamylase [Source:MGI Symbol;Acc:MGI:97448] | yes|up |
| ENSMUSG00000057706 | Mex3b | mex3 RNA binding family member B [Source:MGI Symbol;Acc:MGI:1918252] | yes|up |
| ENSMUSG00000070867 | Trabd2b | TraB domain containing 2B [Source:MGI Symbol;Acc:MGI:3650152] | yes|down |
| ENSMUSG00000039349 | C130074G19Rik | RIKEN cDNA C130074G19 gene [Source:MGI Symbol;Acc:MGI:2444831] | yes|up |
| ENSMUSG00000034520 | Gjc1 | gap junction protein, gamma 1 [Source:MGI Symbol;Acc:MGI:95718] | yes|down |
| ENSMUSG00000021294 | Kif26a | kinesin family member 26A [Source:MGI Symbol;Acc:MGI:2447072] | yes|down |
| ENSMUSG00000034528 | Hsd17b13 | hydroxysteroid (17-beta) dehydrogenase 13 [Source:MGI Symbol;Acc:MGI:2140804] | yes|up |
| ENSMUSG00000029735 | Tpk1 | thiamine pyrophosphokinase [Source:MGI Symbol;Acc:MGI:1352500] | yes|up |
| ENSMUSG00000029730 | Mcm7 | minichromosome maintenance complex component 7 [Source:MGI Symbol;Acc:MGI:1298398] | yes|down |
| ENSMUSG00000050936 | Gm42743 | predicted gene 42743 [Source:MGI Symbol;Acc:MGI:5662880] | yes|up |
| ENSMUSG00000050931 | Sgms2 | sphingomyelin synthase 2 [Source:MGI Symbol;Acc:MGI:1921692] | yes|down |
| ENSMUSG00000020918 | Kat2a | K(lysine) acetyltransferase 2A [Source:MGI Symbol;Acc:MGI:1343101] | yes|down |
| ENSMUSG00000009281 | Rarres2 | retinoic acid receptor responder (tazarotene induced) 2 [Source:MGI Symbol;Acc:MGI:1918910] | yes|up |
| ENSMUSG00000035692 | Isg15 | ISG15 ubiquitin-like modifier [Source:MGI Symbol;Acc:MGI:1855694] | yes|up |
| ENSMUSG00000035929 | H2-Q4 | histocompatibility 2, Q region locus 4 [Source:MGI Symbol;Acc:MGI:95933] | yes|up |
| ENSMUSG00000028063 | Lmna | lamin A [Source:MGI Symbol;Acc:MGI:96794] | yes|down |
| ENSMUSG00000028066 | Pmf1 | polyamine-modulated factor 1 [Source:MGI Symbol;Acc:MGI:1914287] | yes|down |
| ENSMUSG00000028064 | Sema4a | sema domain, immunoglobulin domain (Ig), transmembrane domain (TM) and short cytoplasmic domain, (semaphorin) 4A [Source:MGI Symbol;Acc:MGI:107560] | yes|up |
| ENSMUSG00000028068 | Iqgap3 | IQ motif containing GTPase activating protein 3 [Source:MGI Symbol;Acc:MGI:3028642] | yes|down |
| ENSMUSG00000028069 | Gpatch4 | G patch domain containing 4 [Source:MGI Symbol;Acc:MGI:1913864] | yes|down |
| ENSMUSG00000117123 | Gm49890 | predicted gene, 49890 [Source:MGI Symbol;Acc:MGI:6270578] | yes|down |
| ENSMUSG00000036006 | Ripor2 | RHO family interacting cell polarization regulator 2 [Source:MGI Symbol;Acc:MGI:2444879] | yes|down |
| ENSMUSG00000078234 | Klhdc7a | kelch domain containing 7A [Source:MGI Symbol;Acc:MGI:2444612] | yes|up |
| ENSMUSG00000004864 | Mapk13 | mitogen-activated protein kinase 13 [Source:MGI Symbol;Acc:MGI:1346864] | yes|down |
| ENSMUSG00000121075 |  | novel transcript, antisense to Maff | yes|down |
| ENSMUSG00000048960 | Prex2 | phosphatidylinositol-3,4,5-trisphosphate-dependent Rac exchange factor 2 [Source:MGI Symbol;Acc:MGI:1923385] | yes|up |
| ENSMUSG00000020241 | Col6a2 | collagen, type VI, alpha 2 [Source:MGI Symbol;Acc:MGI:88460] | yes|up |
| ENSMUSG00000054422 | Fabp1 | fatty acid binding protein 1, liver [Source:MGI Symbol;Acc:MGI:95479] | yes|up |
| ENSMUSG00000054136 | Adm2 | adrenomedullin 2 [Source:MGI Symbol;Acc:MGI:2675256] | yes|up |
| ENSMUSG00000032532 | Cck | cholecystokinin [Source:MGI Symbol;Acc:MGI:88297] | yes|down |
| ENSMUSG00000020303 | Stc2 | stanniocalcin 2 [Source:MGI Symbol;Acc:MGI:1316731] | yes|up |
| ENSMUSG00000040084 | Bub1b | BUB1B, mitotic checkpoint serine/threonine kinase [Source:MGI Symbol;Acc:MGI:1333889] | yes|down |
| ENSMUSG00000027387 | Zc3h8 | zinc finger CCCH type containing 8 [Source:MGI Symbol;Acc:MGI:1930128] | yes|down |
| ENSMUSG00000020865 | Abcc3 | ATP-binding cassette, sub-family C (CFTR/MRP), member 3 [Source:MGI Symbol;Acc:MGI:1923658] | yes|up |
| ENSMUSG00000026166 | Ccl20 | chemokine (C-C motif) ligand 20 [Source:MGI Symbol;Acc:MGI:1329031] | yes|up |
| ENSMUSG00000030162 | Olr1 | oxidized low density lipoprotein (lectin-like) receptor 1 [Source:MGI Symbol;Acc:MGI:1261434] | yes|up |
| ENSMUSG00000030160 | Tmem52b | transmembrane protein 52B [Source:MGI Symbol;Acc:MGI:2442838] | yes|up |
| ENSMUSG00000021359 | Tfap2a | transcription factor AP-2, alpha [Source:MGI Symbol;Acc:MGI:104671] | yes|down |
| ENSMUSG00000073274 | Gm14636 | predicted gene 14636 [Source:MGI Symbol;Acc:MGI:3641976] | yes|down |
| ENSMUSG00000031343 | Gabra3 | gamma-aminobutyric acid (GABA) A receptor, subunit alpha 3 [Source:MGI Symbol;Acc:MGI:95615] | yes|up |
| ENSMUSG00000031342 | Gpm6b | glycoprotein m6b [Source:MGI Symbol;Acc:MGI:107672] | yes|up |
| ENSMUSG00000047686 | Rtl3 | retrotransposon Gag like 3 [Source:MGI Symbol;Acc:MGI:2685221] | yes|up |
| ENSMUSG00000038754 | Elovl3 | elongation of very long chain fatty acids (FEN1/Elo2, SUR4/Elo3, yeast)-like 3 [Source:MGI Symbol;Acc:MGI:1195976] | yes|up |
| ENSMUSG00000110598 | Gm45854 | predicted gene 45854 [Source:MGI Symbol;Acc:MGI:5804969] | yes|up |
| ENSMUSG00000026383 | Epb41l5 | erythrocyte membrane protein band 4.1 like 5 [Source:MGI Symbol;Acc:MGI:103006] | yes|up |
| ENSMUSG00000042834 | Nrep | neuronal regeneration related protein [Source:MGI Symbol;Acc:MGI:99444] | yes|up |
| ENSMUSG00000056394 | Lig1 | ligase I, DNA, ATP-dependent [Source:MGI Symbol;Acc:MGI:101789] | yes|down |
| ENSMUSG00000047534 | Mis18bp1 | MIS18 binding protein 1 [Source:MGI Symbol;Acc:MGI:2145099] | yes|down |
| ENSMUSG00000020407 | Upp1 | uridine phosphorylase 1 [Source:MGI Symbol;Acc:MGI:1097668] | yes|down |
| ENSMUSG00000026837 | Col5a1 | collagen, type V, alpha 1 [Source:MGI Symbol;Acc:MGI:88457] | yes|up |
| ENSMUSG00000075602 | Ly6a | lymphocyte antigen 6 complex, locus A [Source:MGI Symbol;Acc:MGI:107527] | yes|down |
| ENSMUSG00000038349 | Plcl1 | phospholipase C-like 1 [Source:MGI Symbol;Acc:MGI:3036262] | yes|up |
| ENSMUSG00000020808 | Pimreg | PICALM interacting mitotic regulator [Source:MGI Symbol;Acc:MGI:1924434] | yes|down |
| ENSMUSG00000029380 | Cxcl1 | chemokine (C-X-C motif) ligand 1 [Source:MGI Symbol;Acc:MGI:108068] | yes|down |
| ENSMUSG00000025154 | Arhgap19 | Rho GTPase activating protein 19 [Source:MGI Symbol;Acc:MGI:1918335] | yes|down |
| ENSMUSG00000028476 | Reck | reversion-inducing-cysteine-rich protein with kazal motifs [Source:MGI Symbol;Acc:MGI:1855698] | yes|up |
| ENSMUSG00000025150 | Cbr2 | carbonyl reductase 2 [Source:MGI Symbol;Acc:MGI:107200] | yes|up |
| ENSMUSG00000048424 | Ranbp3l | RAN binding protein 3-like [Source:MGI Symbol;Acc:MGI:2444654] | yes|down |
| ENSMUSG00000052673 | Gm9887 | predicted gene 9887 [Source:MGI Symbol;Acc:MGI:3642578] | yes|down |
| ENSMUSG00000107877 | 4933427D14Rik | RIKEN cDNA 4933427D14 gene [Source:MGI Symbol;Acc:MGI:1921727] | yes|down |
| ENSMUSG00000054630 | Ugt2b5 | UDP glucuronosyltransferase 2 family, polypeptide B5 [Source:MGI Symbol;Acc:MGI:98900] | yes|up |
| ENSMUSG00000006356 | Crip2 | cysteine rich protein 2 [Source:MGI Symbol;Acc:MGI:1915587] | yes|down |
| ENSMUSG00000018339 | Gpx3 | glutathione peroxidase 3 [Source:MGI Symbol;Acc:MGI:105102] | yes|up |
| ENSMUSG00000032064 | Dixdc1 | DIX domain containing 1 [Source:MGI Symbol;Acc:MGI:2679721] | yes|down |
| ENSMUSG00000032062 | 2310030G06Rik | RIKEN cDNA 2310030G06 gene [Source:MGI Symbol;Acc:MGI:1914202] | yes|down |
| ENSMUSG00000068457 | Uty | ubiquitously transcribed tetratricopeptide repeat containing, Y-linked [Source:MGI Symbol;Acc:MGI:894810] | yes|down |
| ENSMUSG00000019312 | Grb7 | growth factor receptor bound protein 7 [Source:MGI Symbol;Acc:MGI:102683] | yes|up |
| ENSMUSG00000007594 | Hapln4 | hyaluronan and proteoglycan link protein 4 [Source:MGI Symbol;Acc:MGI:2679531] | yes|up |
| ENSMUSG00000032281 | Acsbg1 | acyl-CoA synthetase bubblegum family member 1 [Source:MGI Symbol;Acc:MGI:2385656] | yes|down |
| ENSMUSG00000021922 | Itih4 | inter alpha-trypsin inhibitor, heavy chain 4 [Source:MGI Symbol;Acc:MGI:109536] | yes|up |
| ENSMUSG00000024151 | Msh2 | mutS homolog 2 [Source:MGI Symbol;Acc:MGI:101816] | yes|down |
| ENSMUSG00000079355 | Ackr4 | atypical chemokine receptor 4 [Source:MGI Symbol;Acc:MGI:2181676] | yes|up |
| ENSMUSG00000031722 | Hp | haptoglobin [Source:MGI Symbol;Acc:MGI:96211] | yes|up |
| ENSMUSG00000033880 | Lgals3bp | lectin, galactoside-binding, soluble, 3 binding protein [Source:MGI Symbol;Acc:MGI:99554] | yes|up |
| ENSMUSG00000005338 | Cadm3 | cell adhesion molecule 3 [Source:MGI Symbol;Acc:MGI:2137858] | yes|up |
| ENSMUSG00000050395 | Tnfsf15 | tumor necrosis factor (ligand) superfamily, member 15 [Source:MGI Symbol;Acc:MGI:2180140] | yes|up |
| ENSMUSG00000060678 | H4c3 | H4 clustered histone 3 [Source:MGI Symbol;Acc:MGI:2448421] | yes|up |
| ENSMUSG00000086390 | 1810019D21Rik | RIKEN cDNA 1810019D21 gene [Source:MGI Symbol;Acc:MGI:1917021] | yes|up |
| ENSMUSG00000097558 | Gm26902 | predicted gene, 26902 [Source:MGI Symbol;Acc:MGI:5477396] | yes|up |
| ENSMUSG00000030750 | Nsmce1 | NSE1 homolog, SMC5-SMC6 complex component [Source:MGI Symbol;Acc:MGI:1914961] | yes|down |
| ENSMUSG00000020388 | Pdlim4 | PDZ and LIM domain 4 [Source:MGI Symbol;Acc:MGI:1353470] | yes|up |
| ENSMUSG00000023345 | Poc1a | POC1 centriolar protein A [Source:MGI Symbol;Acc:MGI:1917485] | yes|down |
| ENSMUSG00000020387 | Jade2 | jade family PHD finger 2 [Source:MGI Symbol;Acc:MGI:1924151] | yes|down |
| ENSMUSG00000033083 | Tbc1d4 | TBC1 domain family, member 4 [Source:MGI Symbol;Acc:MGI:2429660] | yes|down |
| ENSMUSG00000108852 | Gm44911 | predicted gene 44911 [Source:MGI Symbol;Acc:MGI:5753487] | yes|down |
| ENSMUSG00000018800 | Abca5 | ATP-binding cassette, sub-family A (ABC1), member 5 [Source:MGI Symbol;Acc:MGI:2386607] | yes|up |
| ENSMUSG00000027715 | Ccna2 | cyclin A2 [Source:MGI Symbol;Acc:MGI:108069] | yes|down |
| ENSMUSG00000022383 | Ppara | peroxisome proliferator activated receptor alpha [Source:MGI Symbol;Acc:MGI:104740] | yes|up |
| ENSMUSG00000031486 | Adgra2 | adhesion G protein-coupled receptor A2 [Source:MGI Symbol;Acc:MGI:1925810] | yes|down |
| ENSMUSG00000055761 | Nkain3 | Na+/K+ transporting ATPase interacting 3 [Source:MGI Symbol;Acc:MGI:2444830] | yes|up |
| ENSMUSG00000055760 | Gemin6 | gem nuclear organelle associated protein 6 [Source:MGI Symbol;Acc:MGI:1914492] | yes|down |
| ENSMUSG00000031480 | Thsd1 | thrombospondin, type I, domain 1 [Source:MGI Symbol;Acc:MGI:1929096] | yes|down |
| ENSMUSG00000006517 | Mvd | mevalonate (diphospho) decarboxylase [Source:MGI Symbol;Acc:MGI:2179327] | yes|up |
| ENSMUSG00000072664 | Ugt3a1 | UDP glycosyltransferases 3 family, polypeptide A1 [Source:MGI Symbol;Acc:MGI:2146055] | yes|up |
| ENSMUSG00000104728 | Gm42462 | predicted gene 42462 [Source:MGI Symbol;Acc:MGI:5662599] | yes|down |
| ENSMUSG00000030284 | Creld1 | cysteine-rich with EGF-like domains 1 [Source:MGI Symbol;Acc:MGI:2152539] | yes|up |
| ENSMUSG00000040147 | Maob | monoamine oxidase B [Source:MGI Symbol;Acc:MGI:96916] | yes|up |
| ENSMUSG00000051444 | Bbs12 | Bardet-Biedl syndrome 12 (human) [Source:MGI Symbol;Acc:MGI:2686651] | yes|down |
| ENSMUSG00000118364 | Gm54420 | predicted gene, 54420 [Source:MGI Symbol;Acc:MGI:6845320] | yes|up |
| ENSMUSG00000046811 | Gltpd2 | glycolipid transfer protein domain containing 2 [Source:MGI Symbol;Acc:MGI:2444527] | yes|up |
| ENSMUSG00000031628 | Casp3 | caspase 3 [Source:MGI Symbol;Acc:MGI:107739] | yes|down |
| ENSMUSG00000031629 | Cenpu | centromere protein U [Source:MGI Symbol;Acc:MGI:1919126] | yes|down |
| ENSMUSG00000070806 | Zmynd12 | zinc finger, MYND domain containing 12 [Source:MGI Symbol;Acc:MGI:2140259] | yes|up |
| ENSMUSG00000017950 | Hnf4a | hepatic nuclear factor 4, alpha [Source:MGI Symbol;Acc:MGI:109128] | yes|up |
| ENSMUSG00000054690 | Emcn | endomucin [Source:MGI Symbol;Acc:MGI:1891716] | yes|up |
| ENSMUSG00000029716 | Tfr2 | transferrin receptor 2 [Source:MGI Symbol;Acc:MGI:1354956] | yes|up |
| ENSMUSG00000029247 | Paics | phosphoribosylaminoimidazole carboxylase, phosphoribosylaminoribosylaminoimidazole, succinocarboxamide synthetase [Source:MGI Symbol;Acc:MGI:1914304] | yes|down |
| ENSMUSG00000029246 | Ppat | phosphoribosyl pyrophosphate amidotransferase [Source:MGI Symbol;Acc:MGI:2387203] | yes|down |
| ENSMUSG00000089706 | B230216N24Rik | RIKEN cDNA B230216N24 gene [Source:MGI Symbol;Acc:MGI:1925853] | yes|down |
| ENSMUSG00000057722 | Lepr | leptin receptor [Source:MGI Symbol;Acc:MGI:104993] | yes|down |
| ENSMUSG00000025384 | Faap100 | Fanconi anemia core complex associated protein 100 [Source:MGI Symbol;Acc:MGI:1919135] | yes|down |
| ENSMUSG00000045875 | Adra1a | adrenergic receptor, alpha 1a [Source:MGI Symbol;Acc:MGI:104773] | yes|up |
| ENSMUSG00000072774 | Zfp951 | zinc finger protein 951 [Source:MGI Symbol;Acc:MGI:2441896] | yes|up |
| ENSMUSG00000035900 | Gramd4 | GRAM domain containing 4 [Source:MGI Symbol;Acc:MGI:2676308] | yes|down |
| ENSMUSG00000069267 | H3c2 | H3 clustered histone 2 [Source:MGI Symbol;Acc:MGI:2448319] | yes|up |
| ENSMUSG00000042155 | Klhl23 | kelch-like 23 [Source:MGI Symbol;Acc:MGI:2683536] | yes|down |
| ENSMUSG00000028044 | Cks1b | CDC28 protein kinase 1b [Source:MGI Symbol;Acc:MGI:1889208] | yes|down |
| ENSMUSG00000029675 | Eln | elastin [Source:MGI Symbol;Acc:MGI:95317] | yes|up |
| ENSMUSG00000102982 | Gm38319 | predicted gene, 38319 [Source:MGI Symbol;Acc:MGI:5611547] | yes|up |
| ENSMUSG00000020672 | Sntg2 | syntrophin, gamma 2 [Source:MGI Symbol;Acc:MGI:1919541] | yes|up |
| ENSMUSG00000020674 | Pxdn | peroxidasin [Source:MGI Symbol;Acc:MGI:1916925] | yes|up |
| ENSMUSG00000069910 | Spdl1 | spindle apparatus coiled-coil protein 1 [Source:MGI Symbol;Acc:MGI:1917635] | yes|down |
| ENSMUSG00000069917 | Hba-a2 | hemoglobin alpha, adult chain 2 [Source:MGI Symbol;Acc:MGI:96016] | yes|up |
| ENSMUSG00000045095 | Magi1 | membrane associated guanylate kinase, WW and PDZ domain containing 1 [Source:MGI Symbol;Acc:MGI:1203522] | yes|up |
| ENSMUSG00000069919 | Hba-a1 | hemoglobin alpha, adult chain 1 [Source:MGI Symbol;Acc:MGI:96015] | yes|up |
| ENSMUSG00000025574 | Tk1 | thymidine kinase 1 [Source:MGI Symbol;Acc:MGI:98763] | yes|down |
| ENSMUSG00000019874 | Fabp7 | fatty acid binding protein 7, brain [Source:MGI Symbol;Acc:MGI:101916] | yes|up |
| ENSMUSG00000019873 | Reep3 | receptor accessory protein 3 [Source:MGI Symbol;Acc:MGI:88930] | yes|up |
| ENSMUSG00000019872 | Smpdl3a | sphingomyelin phosphodiesterase, acid-like 3A [Source:MGI Symbol;Acc:MGI:1931437] | yes|up |
| ENSMUSG00000010830 | Kdelr3 | KDEL (Lys-Asp-Glu-Leu) endoplasmic reticulum protein retention receptor 3 [Source:MGI Symbol;Acc:MGI:2145953] | yes|up |
| ENSMUSG00000027313 | Chac1 | ChaC, cation transport regulator 1 [Source:MGI Symbol;Acc:MGI:1916315] | yes|up |
| ENSMUSG00000027316 | Gfra4 | glial cell line derived neurotrophic factor family receptor alpha 4 [Source:MGI Symbol;Acc:MGI:1341873] | yes|down |
| ENSMUSG00000039952 | Dag1 | dystroglycan 1 [Source:MGI Symbol;Acc:MGI:101864] | yes|up |
| ENSMUSG00000032548 | Slco2a1 | solute carrier organic anion transporter family, member 2a1 [Source:MGI Symbol;Acc:MGI:1346021] | yes|down |
| ENSMUSG00000054409 | Tmem74 | transmembrane protein 74 [Source:MGI Symbol;Acc:MGI:2443417] | yes|down |
| ENSMUSG00000040310 | Alx4 | aristaless-like homeobox 4 [Source:MGI Symbol;Acc:MGI:108359] | yes|down |
| ENSMUSG00000099519 | Gm29253 | predicted gene 29253 [Source:MGI Symbol;Acc:MGI:5579959] | yes|down |
| ENSMUSG00000024365 | Cyp21a1 | cytochrome P450, family 21, subfamily a, polypeptide 1 [Source:MGI Symbol;Acc:MGI:88591] | yes|up |
| ENSMUSG00000037086 | Prr32 | proline rich 32 [Source:MGI Symbol;Acc:MGI:1916050] | yes|up |
| ENSMUSG00000020846 | Rflnb | refilin B [Source:MGI Symbol;Acc:MGI:1923816] | yes|up |
| ENSMUSG00000019813 | Cep57l1 | centrosomal protein 57-like 1 [Source:MGI Symbol;Acc:MGI:1915511] | yes|down |
| ENSMUSG00000050271 | Prag1 | PEAK1 related kinase activating pseudokinase 1 [Source:MGI Symbol;Acc:MGI:1196223] | yes|up |
| ENSMUSG00000101188 | Eif4a-ps4 | eukaryotic translation initiation factor 4A, pseudogene 4 [Source:MGI Symbol;Acc:MGI:1195958] | yes|down |
| ENSMUSG00000026639 | Lamb3 | laminin, beta 3 [Source:MGI Symbol;Acc:MGI:99915] | yes|down |
| ENSMUSG00000062345 | Serpinb2 | serine (or cysteine) peptidase inhibitor, clade B, member 2 [Source:MGI Symbol;Acc:MGI:97609] | yes|down |
| ENSMUSG00000062209 | Erbb4 | erb-b2 receptor tyrosine kinase 4 [Source:MGI Symbol;Acc:MGI:104771] | yes|up |
| ENSMUSG00000097055 | Gm4419 | predicted gene 4419 [Source:MGI Symbol;Acc:MGI:3782604] | yes|down |
| ENSMUSG00000049493 | Pls1 | plastin 1 (I-isoform) [Source:MGI Symbol;Acc:MGI:104809] | yes|down |
| ENSMUSG00000059602 | Syn3 | synapsin III [Source:MGI Symbol;Acc:MGI:1351334] | yes|up |
| ENSMUSG00000062981 | Mrpl42 | mitochondrial ribosomal protein L42 [Source:MGI Symbol;Acc:MGI:1333774] | yes|down |
| ENSMUSG00000056004 | Elapor2 | endosome-lysosome associated apoptosis and autophagy regulator family member 2 [Source:MGI Symbol;Acc:MGI:2443264] | yes|up |
| ENSMUSG00000004655 | Aqp1 | aquaporin 1 [Source:MGI Symbol;Acc:MGI:103201] | yes|down |
| ENSMUSG00000041406 | BC055324 | cDNA sequence BC055324 [Source:MGI Symbol;Acc:MGI:3590554] | yes|down |
| ENSMUSG00000041959 | S100a10 | S100 calcium binding protein A10 (calpactin) [Source:MGI Symbol;Acc:MGI:1339468] | yes|down |
| ENSMUSG00000047330 | Kcne4 | potassium voltage-gated channel, Isk-related subfamily, gene 4 [Source:MGI Symbol;Acc:MGI:1891125] | yes|up |
| ENSMUSG00000105353 | Gm42428 | predicted gene 42428 [Source:MGI Symbol;Acc:MGI:5662565] | yes|up |
| ENSMUSG00000038775 | Vill | villin-like [Source:MGI Symbol;Acc:MGI:1201781] | yes|up |
| ENSMUSG00000042812 | Foxf1 | forkhead box F1 [Source:MGI Symbol;Acc:MGI:1347470] | yes|up |
| ENSMUSG00000046318 | Ccbe1 | collagen and calcium binding EGF domains 1 [Source:MGI Symbol;Acc:MGI:2445053] | yes|down |
| ENSMUSG00000058897 | Col25a1 | collagen, type XXV, alpha 1 [Source:MGI Symbol;Acc:MGI:1924268] | yes|up |
| ENSMUSG00000016200 | Syt14 | synaptotagmin XIV [Source:MGI Symbol;Acc:MGI:2444490] | yes|up |
| ENSMUSG00000034265 | Zdhhc14 | zinc finger, DHHC domain containing 14 [Source:MGI Symbol;Acc:MGI:2653229] | yes|up |
| ENSMUSG00000043366 | Olfr78 | olfactory receptor 78 [Source:MGI Symbol;Acc:MGI:2157548] | yes|up |
| ENSMUSG00000028678 | Kif2c | kinesin family member 2C [Source:MGI Symbol;Acc:MGI:1921054] | yes|down |
| ENSMUSG00000087247 | Alkal1 | ALK and LTK ligand 1 [Source:MGI Symbol;Acc:MGI:3645495] | yes|up |
| ENSMUSG00000052125 | F730043M19Rik | RIKEN cDNA F730043M19 gene [Source:MGI Symbol;Acc:MGI:2443237] | yes|up |
| ENSMUSG00000120284 |  | novel transcript, sense intronic to Cpq | yes|down |
| ENSMUSG00000038943 | Prc1 | protein regulator of cytokinesis 1 [Source:MGI Symbol;Acc:MGI:1858961] | yes|down |
| ENSMUSG00000042367 | Gjb3 | gap junction protein, beta 3 [Source:MGI Symbol;Acc:MGI:95721] | yes|down |
| ENSMUSG00000042363 | Lgalsl | lectin, galactoside binding-like [Source:MGI Symbol;Acc:MGI:1916114] | yes|up |
| ENSMUSG00000025175 | Fn3k | fructosamine 3 kinase [Source:MGI Symbol;Acc:MGI:1926834] | yes|up |
| ENSMUSG00000028459 | Cd72 | CD72 antigen [Source:MGI Symbol;Acc:MGI:88345] | yes|up |
| ENSMUSG00000078688 | Mup2 | major urinary protein 2 [Source:MGI Symbol;Acc:MGI:97234] | yes|up |
| ENSMUSG00000078680 | Mup10 | major urinary protein 10 [Source:MGI Symbol;Acc:MGI:1924164] | yes|up |
| ENSMUSG00000019992 | Mtfr2 | mitochondrial fission regulator 2 [Source:MGI Symbol;Acc:MGI:1919054] | yes|down |
| ENSMUSG00000114469 | C730002L08Rik | RIKEN cDNA C730002L08 gene [Source:MGI Symbol;Acc:MGI:2443493] | yes|down |
| ENSMUSG00000062593 | Gm49339 | predicted gene, 49339 [Source:MGI Symbol;Acc:MGI:6121530] | yes|up |
| ENSMUSG00000036639 | Nudt1 | nudix (nucleoside diphosphate linked moiety X)-type motif 1 [Source:MGI Symbol;Acc:MGI:109280] | yes|down |
| ENSMUSG00000014158 | Trpv4 | transient receptor potential cation channel, subfamily V, member 4 [Source:MGI Symbol;Acc:MGI:1926945] | yes|down |
| ENSMUSG00000051726 | Kcnf1 | potassium voltage-gated channel, subfamily F, member 1 [Source:MGI Symbol;Acc:MGI:2687399] | yes|down |
| ENSMUSG00000058070 | Eml1 | echinoderm microtubule associated protein like 1 [Source:MGI Symbol;Acc:MGI:1915769] | yes|down |
| ENSMUSG00000101249 | Gm29216 | predicted gene 29216 [Source:MGI Symbol;Acc:MGI:5579922] | yes|down |
| ENSMUSG00000104988 | Gm43622 | predicted gene 43622 [Source:MGI Symbol;Acc:MGI:5663759] | yes|up |
| ENSMUSG00000030772 | Dkk3 | dickkopf WNT signaling pathway inhibitor 3 [Source:MGI Symbol;Acc:MGI:1354952] | yes|up |
| ENSMUSG00000043969 | Emx2 | empty spiracles homeobox 2 [Source:MGI Symbol;Acc:MGI:95388] | yes|up |
| ENSMUSG00000014542 | Clec4f | C-type lectin domain family 4, member f [Source:MGI Symbol;Acc:MGI:1859834] | yes|up |
| ENSMUSG00000000942 | Hoxa4 | homeobox A4 [Source:MGI Symbol;Acc:MGI:96176] | yes|up |
| ENSMUSG00000005718 | Tfap4 | transcription factor AP4 [Source:MGI Symbol;Acc:MGI:103239] | yes|down |
| ENSMUSG00000000948 | Gm38393 | predicted gene, 38393 [Source:MGI Symbol;Acc:MGI:5613898] | yes|up |
| ENSMUSG00000030551 | Nr2f2 | nuclear receptor subfamily 2, group F, member 2 [Source:MGI Symbol;Acc:MGI:1352452] | yes|down |
| ENSMUSG00000040127 | Sdr9c7 | 4short chain dehydrogenase/reductase family 9C, member 7 [Source:MGI Symbol;Acc:MGI:1917311] | yes|up |
| ENSMUSG00000021259 | Cyp46a1 | cytochrome P450, family 46, subfamily a, polypeptide 1 [Source:MGI Symbol;Acc:MGI:1341877] | yes|down |
| ENSMUSG00000021253 | Tgfb3 | transforming growth factor, beta 3 [Source:MGI Symbol;Acc:MGI:98727] | yes|up |
| ENSMUSG00000039385 | Cdh6 | cadherin 6 [Source:MGI Symbol;Acc:MGI:107435] | yes|up |
| ENSMUSG00000029082 | Bst1 | bone marrow stromal cell antigen 1 [Source:MGI Symbol;Acc:MGI:105370] | yes|down |
| ENSMUSG00000026463 | Atp2b4 | ATPase, Ca++ transporting, plasma membrane 4 [Source:MGI Symbol;Acc:MGI:88111] | yes|up |
| ENSMUSG00000061615 | H2ac4 | H2A clustered histone 4 [Source:MGI Symbol;Acc:MGI:2448306] | yes|up |
| ENSMUSG00000049630 | C1ql3 | C1q-like 3 [Source:MGI Symbol;Acc:MGI:2387350] | yes|up |
| ENSMUSG00000015943 | Bola1 | bolA-like 1 (E. coli) [Source:MGI Symbol;Acc:MGI:1916418] | yes|down |
| ENSMUSG00000089726 | Mir17hg | Mir17 host gene (non-protein coding) [Source:MGI Symbol;Acc:MGI:1923207] | yes|down |
| ENSMUSG00000097855 | A930007I19Rik | RIKEN cDNA A930007I19 gene [Source:MGI Symbol;Acc:MGI:1925029] | yes|up |
| ENSMUSG00000053199 | Arhgap20 | Rho GTPase activating protein 20 [Source:MGI Symbol;Acc:MGI:2445175] | yes|up |
| ENSMUSG00000028024 | Enpep | glutamyl aminopeptidase [Source:MGI Symbol;Acc:MGI:106645] | yes|up |
| ENSMUSG00000028020 | Glrb | glycine receptor, beta subunit [Source:MGI Symbol;Acc:MGI:95751] | yes|up |
| ENSMUSG00000035031 | C8a | complement component 8, alpha polypeptide [Source:MGI Symbol;Acc:MGI:2668347] | yes|up |
| ENSMUSG00000095186 | Gm10718 | predicted gene 10718 [Source:MGI Symbol;Acc:MGI:3642028] | yes|up |
| ENSMUSG00000020614 | Fam20a | FAM20A, golgi associated secretory pathway pseudokinase [Source:MGI Symbol;Acc:MGI:2388266] | yes|up |
| ENSMUSG00000008668 | Rps18 | ribosomal protein S18 [Source:MGI Symbol;Acc:MGI:98146] | yes|down |
| ENSMUSG00000064225 | Paqr9 | progestin and adipoQ receptor family member IX [Source:MGI Symbol;Acc:MGI:1922802] | yes|up |
| ENSMUSG00000063506 | Arhgap22 | Rho GTPase activating protein 22 [Source:MGI Symbol;Acc:MGI:2443418] | yes|down |
| ENSMUSG00000064220 | H2ac18 | H2A clustered histone 18 [Source:MGI Symbol;Acc:MGI:96097] | yes|up |
| ENSMUSG00000018451 | 6330403K07Rik | RIKEN cDNA 6330403K07 gene [Source:MGI Symbol;Acc:MGI:1918001] | yes|up |
| ENSMUSG00000018102 | H2bc4 | H2B clustered histone 4 [Source:MGI Symbol;Acc:MGI:1915274] | yes|up |
| ENSMUSG00000052353 | Cemip | cell migration inducing protein, hyaluronan binding [Source:MGI Symbol;Acc:MGI:2443629] | yes|up |
| ENSMUSG00000068086 | Cyp2d9 | cytochrome P450, family 2, subfamily d, polypeptide 9 [Source:MGI Symbol;Acc:MGI:88606] | yes|up |
| ENSMUSG00000044927 | H1f10 | H1.10 linker histone [Source:MGI Symbol;Acc:MGI:2685307] | yes|down |
| ENSMUSG00000068083 | Cyp2d40 | cytochrome P450, family 2, subfamily d, polypeptide 40 [Source:MGI Symbol;Acc:MGI:1919004] | yes|up |
| ENSMUSG00000020866 | Cacna1g | calcium channel, voltage-dependent, T type, alpha 1G subunit [Source:MGI Symbol;Acc:MGI:1201678] | yes|up |
| ENSMUSG00000078762 | Haus5 | HAUS augmin-like complex, subunit 5 [Source:MGI Symbol;Acc:MGI:1919159] | yes|down |
| ENSMUSG00000019852 | Arfgef3 | ARFGEF family member 3 [Source:MGI Symbol;Acc:MGI:106387] | yes|up |
| ENSMUSG00000019850 | Tnfaip3 | tumor necrosis factor, alpha-induced protein 3 [Source:MGI Symbol;Acc:MGI:1196377] | yes|down |
| ENSMUSG00000103653 | Gstp-ps | glutathione S-transferase, pi, pseudogene [Source:MGI Symbol;Acc:MGI:3782108] | yes|down |
| ENSMUSG00000048922 | Cdca2 | cell division cycle associated 2 [Source:MGI Symbol;Acc:MGI:1919787] | yes|down |
| ENSMUSG00000111205 | Gm39307 | predicted gene, 39307 [Source:MGI Symbol;Acc:MGI:5622192] | yes|down |
| ENSMUSG00000111202 | Gm48275 | predicted gene, 48275 [Source:MGI Symbol;Acc:MGI:6097702] | yes|down |
| ENSMUSG00000079415 | Cntf | ciliary neurotrophic factor [Source:MGI Symbol;Acc:MGI:88439] | yes|up |
| ENSMUSG00000113152 | Gm48422 | predicted gene, 48422 [Source:MGI Symbol;Acc:MGI:6097918] | yes|up |
| ENSMUSG00000100309 | Gm6644 | predicted gene 6644 [Source:MGI Symbol;Acc:MGI:3644951] | yes|down |
| ENSMUSG00000000028 | Cdc45 | cell division cycle 45 [Source:MGI Symbol;Acc:MGI:1338073] | yes|down |
| ENSMUSG00000093587 | Gm20554 | predicted gene, 20554 [Source:MGI Symbol;Acc:MGI:5295661] | yes|up |
| ENSMUSG00000097078 | Gm26566 | predicted gene, 26566 [Source:MGI Symbol;Acc:MGI:5477060] | yes|up |
| ENSMUSG00000097073 | 9430037G07Rik | RIKEN cDNA 9430037G07 gene [Source:MGI Symbol;Acc:MGI:2444544] | yes|up |
| ENSMUSG00000030123 | Plxnd1 | plexin D1 [Source:MGI Symbol;Acc:MGI:2154244] | yes|up |
| ENSMUSG00000040841 | Six5 | sine oculis-related homeobox 5 [Source:MGI Symbol;Acc:MGI:106220] | yes|up |
| ENSMUSG00000060882 | Kcnd2 | potassium voltage-gated channel, Shal-related family, member 2 [Source:MGI Symbol;Acc:MGI:102663] | yes|up |
| ENSMUSG00000054342 | Kcnn4 | potassium intermediate/small conductance calcium-activated channel, subfamily N, member 4 [Source:MGI Symbol;Acc:MGI:1277957] | yes|down |
| ENSMUSG00000056025 | Clca3a1 | chloride channel accessory 3A1 [Source:MGI Symbol;Acc:MGI:1316732] | yes|up |
| ENSMUSG00000063286 | Gvin-ps7 | GTPase, very large interferon inducible, pseudogene 7 [Source:MGI Symbol;Acc:MGI:3644223] | yes|up |
| ENSMUSG00000022758 | P2rx6 | purinergic receptor P2X, ligand-gated ion channel, 6 [Source:MGI Symbol;Acc:MGI:1337113] | yes|up |
| ENSMUSG00000066072 | Cyp4a10 | cytochrome P450, family 4, subfamily a, polypeptide 10 [Source:MGI Symbol;Acc:MGI:88611] | yes|up |
| ENSMUSG00000066071 | Cyp4a12a | cytochrome P450, family 4, subfamily a, polypeptide 12a [Source:MGI Symbol;Acc:MGI:88612] | yes|up |
| ENSMUSG00000105881 | 4932422M17Rik | RIKEN cDNA 4932422M17 gene [Source:MGI Symbol;Acc:MGI:1921616] | yes|up |
| ENSMUSG00000003363 | Pld3 | phospholipase D family, member 3 [Source:MGI Symbol;Acc:MGI:1333782] | yes|up |
| ENSMUSG00000026875 | Traf1 | TNF receptor-associated factor 1 [Source:MGI Symbol;Acc:MGI:101836] | yes|down |
| ENSMUSG00000026874 | Hc | hemolytic complement [Source:MGI Symbol;Acc:MGI:96031] | yes|up |
| ENSMUSG00000082585 | Gm15387 | predicted gene 15387 [Source:MGI Symbol;Acc:MGI:3705374] | yes|down |
| ENSMUSG00000074874 | Ctla2b | cytotoxic T lymphocyte-associated protein 2 beta [Source:MGI Symbol;Acc:MGI:88555] | yes|up |
| ENSMUSG00000089809 | Rasgef1b | RasGEF domain family, member 1B [Source:MGI Symbol;Acc:MGI:2443755] | yes|up |
| ENSMUSG00000029695 | Aass | aminoadipate-semialdehyde synthase [Source:MGI Symbol;Acc:MGI:1353573] | yes|up |
| ENSMUSG00000103983 | Gm20045 | predicted gene, 20045 [Source:MGI Symbol;Acc:MGI:5012230] | yes|up |
| ENSMUSG00000042694 | Stn1 | STN1, CST complex subunit [Source:MGI Symbol;Acc:MGI:1915581] | yes|down |
| ENSMUSG00000025991 | Cps1 | carbamoyl-phosphate synthetase 1 [Source:MGI Symbol;Acc:MGI:891996] | yes|up |
| ENSMUSG00000028614 | Ndc1 | NDC1 transmembrane nucleoporin [Source:MGI Symbol;Acc:MGI:1920037] | yes|down |
| ENSMUSG00000025993 | Slc40a1 | solute carrier family 40 (iron-regulated transporter), member 1 [Source:MGI Symbol;Acc:MGI:1315204] | yes|up |
| ENSMUSG00000034248 | Slc25a37 | solute carrier family 25, member 37 [Source:MGI Symbol;Acc:MGI:1914962] | yes|down |
| ENSMUSG00000028619 | Tceanc2 | transcription elongation factor A (SII) N-terminal and central domain containing 2 [Source:MGI Symbol;Acc:MGI:1913776] | yes|up |
| ENSMUSG00000021062 | Rab15 | RAB15, member RAS oncogene family [Source:MGI Symbol;Acc:MGI:1916865] | yes|up |
| ENSMUSG00000032715 | Trib3 | tribbles pseudokinase 3 [Source:MGI Symbol;Acc:MGI:1345675] | yes|up |
| ENSMUSG00000032718 | Mansc1 | MANSC domain containing 1 [Source:MGI Symbol;Acc:MGI:1914979] | yes|up |
| ENSMUSG00000029528 | Pxn | paxillin [Source:MGI Symbol;Acc:MGI:108295] | yes|down |
| ENSMUSG00000040187 | Arntl2 | aryl hydrocarbon receptor nuclear translocator-like 2 [Source:MGI Symbol;Acc:MGI:2684845] | yes|down |
| ENSMUSG00000025194 | Abcc2 | ATP-binding cassette, sub-family C (CFTR/MRP), member 2 [Source:MGI Symbol;Acc:MGI:1352447] | yes|up |
| ENSMUSG00000025196 | Cpn1 | carboxypeptidase N, polypeptide 1 [Source:MGI Symbol;Acc:MGI:2135874] | yes|up |
| ENSMUSG00000074277 | Phldb3 | pleckstrin homology like domain, family B, member 3 [Source:MGI Symbol;Acc:MGI:3642959] | yes|up |
| ENSMUSG00000026388 | 3110009E18Rik | RIKEN cDNA 3110009E18 gene [Source:MGI Symbol;Acc:MGI:1920353] | yes|down |
| ENSMUSG00000018593 | Sparc | secreted acidic cysteine rich glycoprotein [Source:MGI Symbol;Acc:MGI:98373] | yes|up |
| ENSMUSG00000074896 | Ifit3 | interferon-induced protein with tetratricopeptide repeats 3 [Source:MGI Symbol;Acc:MGI:1101055] | yes|up |
| ENSMUSG00000091400 | Gm6356 | predicted gene 6356 [Source:MGI Symbol;Acc:MGI:3646390] | yes|up |
| ENSMUSG00000121404 |  | novel transcript | yes|down |
| ENSMUSG00000033594 | Spata2l | spermatogenesis associated 2-like [Source:MGI Symbol;Acc:MGI:1926029] | yes|up |
| ENSMUSG00000024805 | Pcgf5 | polycomb group ring finger 5 [Source:MGI Symbol;Acc:MGI:1923505] | yes|down |
| ENSMUSG00000024806 | Mlana | melan-A [Source:MGI Symbol;Acc:MGI:108454] | yes|up |
| ENSMUSG00000030717 | Nupr1 | nuclear protein transcription regulator 1 [Source:MGI Symbol;Acc:MGI:1891834] | yes|up |
| ENSMUSG00000030711 | Sult1a1 | sulfotransferase family 1A, phenol-preferring, member 1 [Source:MGI Symbol;Acc:MGI:102896] | yes|up |
| ENSMUSG00000059456 | Ptk2b | PTK2 protein tyrosine kinase 2 beta [Source:MGI Symbol;Acc:MGI:104908] | yes|down |
| ENSMUSG00000001039 | B9d1 | B9 protein domain 1 [Source:MGI Symbol;Acc:MGI:1351471] | yes|down |
| ENSMUSG00000043903 | Zfp469 | zinc finger protein 469 [Source:MGI Symbol;Acc:MGI:2684868] | yes|up |
| ENSMUSG00000051627 | H1f4 | H1.4 linker histone, cluster member [Source:MGI Symbol;Acc:MGI:1931527] | yes|up |
| ENSMUSG00000048120 | Entpd1 | ectonucleoside triphosphate diphosphohydrolase 1 [Source:MGI Symbol;Acc:MGI:102805] | yes|down |
| ENSMUSG00000039633 | Lonrf1 | LON peptidase N-terminal domain and ring finger 1 [Source:MGI Symbol;Acc:MGI:3609241] | yes|up |
| ENSMUSG00000031444 | F10 | coagulation factor X [Source:MGI Symbol;Acc:MGI:103107] | yes|up |
| ENSMUSG00000023341 | Mx2 | MX dynamin-like GTPase 2 [Source:MGI Symbol;Acc:MGI:97244] | yes|up |
| ENSMUSG00000074476 | Spc24 | SPC24, NDC80 kinetochore complex component, homolog (S. cerevisiae) [Source:MGI Symbol;Acc:MGI:1914879] | yes|down |
| ENSMUSG00000029068 | Ccnl2 | cyclin L2 [Source:MGI Symbol;Acc:MGI:1927119] | yes|up |
| ENSMUSG00000029061 | Mmp23 | matrix metallopeptidase 23 [Source:MGI Symbol;Acc:MGI:1347361] | yes|up |
| ENSMUSG00000031661 | Nkd1 | naked cuticle 1 [Source:MGI Symbol;Acc:MGI:2135954] | yes|up |
| ENSMUSG00000023169 | Slc38a1 | solute carrier family 38, member 1 [Source:MGI Symbol;Acc:MGI:2145895] | yes|up |
| ENSMUSG00000026405 | C4bp | complement component 4 binding protein [Source:MGI Symbol;Acc:MGI:88229] | yes|up |
| ENSMUSG00000025104 | Hdgfl3 | HDGF like 3 [Source:MGI Symbol;Acc:MGI:1352760] | yes|down |
| ENSMUSG00000056270 | Prr9 | proline rich 9 [Source:MGI Symbol;Acc:MGI:1925680] | yes|up |
| ENSMUSG00000021186 | Fbln5 | fibulin 5 [Source:MGI Symbol;Acc:MGI:1346091] | yes|up |
| ENSMUSG00000074785 | Plxnc1 | plexin C1 [Source:MGI Symbol;Acc:MGI:1890127] | yes|up |
| ENSMUSG00000074782 | 4833422C13Rik | RIKEN cDNA 4833422C13 gene [Source:MGI Symbol;Acc:MGI:3603826] | yes|up |
| ENSMUSG00000021180 | Rps6ka5 | ribosomal protein S6 kinase, polypeptide 5 [Source:MGI Symbol;Acc:MGI:1920336] | yes|down |
| ENSMUSG00000029201 | Ugdh | UDP-glucose dehydrogenase [Source:MGI Symbol;Acc:MGI:1306785] | yes|down |
| ENSMUSG00000003779 | Kif20a | kinesin family member 20A [Source:MGI Symbol;Acc:MGI:1201682] | yes|down |
| ENSMUSG00000047104 | Pbp2 | phosphatidylethanolamine binding protein 2 [Source:MGI Symbol;Acc:MGI:1923650] | yes|down |
| ENSMUSG00000069049 | Eif2s3y | eukaryotic translation initiation factor 2, subunit 3, structural gene Y-linked [Source:MGI Symbol;Acc:MGI:1349430] | yes|down |
| ENSMUSG00000015968 | Cacna1d | calcium channel, voltage-dependent, L type, alpha 1D subunit [Source:MGI Symbol;Acc:MGI:88293] | yes|down |
| ENSMUSG00000069045 | Ddx3y | DEAD box helicase 3, Y-linked [Source:MGI Symbol;Acc:MGI:1349406] | yes|down |
| ENSMUSG00000007646 | Rad51c | RAD51 paralog C [Source:MGI Symbol;Acc:MGI:2150020] | yes|down |
| ENSMUSG00000015217 | Hmgb3 | high mobility group box 3 [Source:MGI Symbol;Acc:MGI:1098219] | yes|down |
| ENSMUSG00000024935 | Slc1a1 | solute carrier family 1 (neuronal/epithelial high affinity glutamate transporter, system Xag), member 1 [Source:MGI Symbol;Acc:MGI:105083] | yes|up |
| ENSMUSG00000020901 | Pik3r5 | phosphoinositide-3-kinase regulatory subunit 5 [Source:MGI Symbol;Acc:MGI:2443588] | yes|down |
| ENSMUSG00000020900 | Myh10 | myosin, heavy polypeptide 10, non-muscle [Source:MGI Symbol;Acc:MGI:1930780] | yes|up |
| ENSMUSG00000020583 | Matn3 | matrilin 3 [Source:MGI Symbol;Acc:MGI:1328350] | yes|up |
| ENSMUSG00000114117 | Gm48795 | predicted gene, 48795 [Source:MGI Symbol;Acc:MGI:6098498] | yes|down |
| ENSMUSG00000020638 | Cmpk2 | cytidine monophosphate (UMP-CMP) kinase 2, mitochondrial [Source:MGI Symbol;Acc:MGI:99830] | yes|up |
| ENSMUSG00000028001 | Fga | fibrinogen alpha chain [Source:MGI Symbol;Acc:MGI:1316726] | yes|up |
| ENSMUSG00000034906 | Ncaph | non-SMC condensin I complex, subunit H [Source:MGI Symbol;Acc:MGI:2444777] | yes|down |
| ENSMUSG00000028005 | Gucy1b1 | guanylate cyclase 1, soluble, beta 1 [Source:MGI Symbol;Acc:MGI:1860604] | yes|up |
| ENSMUSG00000028558 | Calr4 | calreticulin 4 [Source:MGI Symbol;Acc:MGI:2140435] | yes|down |
| ENSMUSG00000052595 | A1cf | APOBEC1 complementation factor [Source:MGI Symbol;Acc:MGI:1917115] | yes|up |
| ENSMUSG00000072940 | Gm10443 | predicted pseudogene 10443 [Source:MGI Symbol;Acc:MGI:3704272] | yes|down |
| ENSMUSG00000072944 | Nup62cl | nucleoporin 62 C-terminal like [Source:MGI Symbol;Acc:MGI:2685565] | yes|up |
| ENSMUSG00000100980 | Gm29100 | predicted gene 29100 [Source:MGI Symbol;Acc:MGI:5579806] | yes|down |
| ENSMUSG00000063564 | Col23a1 | collagen, type XXIII, alpha 1 [Source:MGI Symbol;Acc:MGI:2653243] | yes|up |
| ENSMUSG00000032368 | Zic1 | zinc finger protein of the cerebellum 1 [Source:MGI Symbol;Acc:MGI:106683] | yes|up |
| ENSMUSG00000039191 | Rbpj | recombination signal binding protein for immunoglobulin kappa J region [Source:MGI Symbol;Acc:MGI:96522] | yes|down |
| ENSMUSG00000093769 | H3c14 | H3 clustered histone 14 [Source:MGI Symbol;Acc:MGI:2448355] | yes|up |
| ENSMUSG00000092572 | Serpinb10 | serine (or cysteine) peptidase inhibitor, clade B (ovalbumin), member 10 [Source:MGI Symbol;Acc:MGI:2138648] | yes|down |
| ENSMUSG00000045362 | Tnfrsf26 | tumor necrosis factor receptor superfamily, member 26 [Source:MGI Symbol;Acc:MGI:2651928] | yes|up |
| ENSMUSG00000018166 | Erbb3 | erb-b2 receptor tyrosine kinase 3 [Source:MGI Symbol;Acc:MGI:95411] | yes|up |
| ENSMUSG00000033361 | Prrg3 | proline rich Gla (G-carboxyglutamic acid) 3 (transmembrane) [Source:MGI Symbol;Acc:MGI:2685214] | yes|down |
| ENSMUSG00000002910 | Arrdc2 | arrestin domain containing 2 [Source:MGI Symbol;Acc:MGI:1918057] | yes|up |
| ENSMUSG00000039994 | Timeless | timeless circadian clock 1 [Source:MGI Symbol;Acc:MGI:1321393] | yes|down |
| ENSMUSG00000032502 | Stac | src homology three (SH3) and cysteine rich domain [Source:MGI Symbol;Acc:MGI:1201400] | yes|down |
| ENSMUSG00000032501 | Trib1 | tribbles pseudokinase 1 [Source:MGI Symbol;Acc:MGI:2443397] | yes|down |
| ENSMUSG00000037845 | Fdxacb1 | ferredoxin-fold anticodon binding domain containing 1 [Source:MGI Symbol;Acc:MGI:3584513] | yes|down |
| ENSMUSG00000037846 | Rtkn2 | rhotekin 2 [Source:MGI Symbol;Acc:MGI:2158417] | yes|down |
| ENSMUSG00000019966 | Kitl | kit ligand [Source:MGI Symbol;Acc:MGI:96974] | yes|down |
| ENSMUSG00000037594 | Clba1 | clathrin binding box of aftiphilin containing 1 [Source:MGI Symbol;Acc:MGI:2443738] | yes|down |
| ENSMUSG00000111229 | Gm39323 | predicted gene, 39323 [Source:MGI Symbol;Acc:MGI:5622208] | yes|up |
| ENSMUSG00000033105 | Lss | lanosterol synthase [Source:MGI Symbol;Acc:MGI:1336155] | yes|up |
| ENSMUSG00000050052 | Tdrp | testis development related protein [Source:MGI Symbol;Acc:MGI:1919398] | yes|up |
| ENSMUSG00000026675 | Hsd17b7 | hydroxysteroid (17-beta) dehydrogenase 7 [Source:MGI Symbol;Acc:MGI:1330808] | yes|up |
| ENSMUSG00000026676 | Ccdc3 | coiled-coil domain containing 3 [Source:MGI Symbol;Acc:MGI:1921436] | yes|up |
| ENSMUSG00000001930 | Vwf | Von Willebrand factor [Source:MGI Symbol;Acc:MGI:98941] | yes|up |
| ENSMUSG00000059022 | Kcp | kielin/chordin-like protein [Source:MGI Symbol;Acc:MGI:2141640] | yes|up |
| ENSMUSG00000049422 | Chchd10 | coiled-coil-helix-coiled-coil-helix domain containing 10 [Source:MGI Symbol;Acc:MGI:2143558] | yes|up |
| ENSMUSG00000116908 | Gm49599 | predicted gene, 49599 [Source:MGI Symbol;Acc:MGI:6215007] | yes|up |
| ENSMUSG00000038732 | Mboat1 | membrane bound O-acyltransferase domain containing 1 [Source:MGI Symbol;Acc:MGI:2387184] | yes|down |
| ENSMUSG00000030103 | Bhlhe40 | basic helix-loop-helix family, member e40 [Source:MGI Symbol;Acc:MGI:1097714] | yes|up |
| ENSMUSG00000037337 | Map4k1 | mitogen-activated protein kinase kinase kinase kinase 1 [Source:MGI Symbol;Acc:MGI:1346882] | yes|down |
| ENSMUSG00000030107 | Usp18 | ubiquitin specific peptidase 18 [Source:MGI Symbol;Acc:MGI:1344364] | yes|up |
| ENSMUSG00000027852 | Nras | neuroblastoma ras oncogene [Source:MGI Symbol;Acc:MGI:97376] | yes|down |
| ENSMUSG00000020805 | Slc13a5 | solute carrier family 13 (sodium-dependent citrate transporter), member 5 [Source:MGI Symbol;Acc:MGI:3037150] | yes|up |
| ENSMUSG00000041444 | Arhgap32 | Rho GTPase activating protein 32 [Source:MGI Symbol;Acc:MGI:2450166] | yes|down |
| ENSMUSG00000016984 | Etaa1 | Ewing tumor-associated antigen 1 [Source:MGI Symbol;Acc:MGI:1915395] | yes|down |
| ENSMUSG00000078877 | Gm14295 | predicted gene 14295 [Source:MGI Symbol;Acc:MGI:3709624] | yes|down |
| ENSMUSG00000076498 | Trbc2 | T cell receptor beta, constant 2 [Source:MGI Symbol;Acc:MGI:4835227] | yes|down |
| ENSMUSG00000023571 | C1qtnf12 | C1q and tumor necrosis factor related 12 [Source:MGI Symbol;Acc:MGI:1914639] | yes|down |
| ENSMUSG00000036887 | C1qa | complement component 1, q subcomponent, alpha polypeptide [Source:MGI Symbol;Acc:MGI:88223] | yes|up |
| ENSMUSG00000036885 | Arhgef26 | Rho guanine nucleotide exchange factor (GEF) 26 [Source:MGI Symbol;Acc:MGI:1918053] | yes|up |
| ENSMUSG00000073787 | Gm10575 | predicted gene 10575 [Source:MGI Symbol;Acc:MGI:3708765] | yes|down |
| ENSMUSG00000031271 | Serpina7 | serine (or cysteine) peptidase inhibitor, clade A (alpha-1 antiproteinase, antitrypsin), member 7 [Source:MGI Symbol;Acc:MGI:3041197] | yes|up |
| ENSMUSG00000002265 | Peg3 | paternally expressed 3 [Source:MGI Symbol;Acc:MGI:104748] | yes|up |
| ENSMUSG00000002266 | Zim1 | zinc finger, imprinted 1 [Source:MGI Symbol;Acc:MGI:1341879] | yes|up |
| ENSMUSG00000026890 | Lhx6 | LIM homeobox protein 6 [Source:MGI Symbol;Acc:MGI:1306803] | yes|down |
| ENSMUSG00000026896 | Ifih1 | interferon induced with helicase C domain 1 [Source:MGI Symbol;Acc:MGI:1918836] | yes|up |
| ENSMUSG00000040717 | Il17rd | interleukin 17 receptor D [Source:MGI Symbol;Acc:MGI:2159727] | yes|up |
| ENSMUSG00000074457 | S100a16 | S100 calcium binding protein A16 [Source:MGI Symbol;Acc:MGI:1915110] | yes|down |
| ENSMUSG00000025203 | Scd2 | stearoyl-Coenzyme A desaturase 2 [Source:MGI Symbol;Acc:MGI:98240] | yes|up |
| ENSMUSG00000028634 | Hivep3 | human immunodeficiency virus type I enhancer binding protein 3 [Source:MGI Symbol;Acc:MGI:106589] | yes|down |
| ENSMUSG00000025207 | Sema4g | sema domain, immunoglobulin domain (Ig), transmembrane domain (TM) and short cytoplasmic domain, (semaphorin) 4G [Source:MGI Symbol;Acc:MGI:1347047] | yes|up |
| ENSMUSG00000034731 | Dgkh | diacylglycerol kinase, eta [Source:MGI Symbol;Acc:MGI:2444188] | yes|up |
| ENSMUSG00000039055 | Eme1 | essential meiotic structure-specific endonuclease 1 [Source:MGI Symbol;Acc:MGI:3576783] | yes|down |
| ENSMUSG00000029544 | Cabp1 | calcium binding protein 1 [Source:MGI Symbol;Acc:MGI:1352750] | yes|up |
| ENSMUSG00000024678 | Ms4a4d | membrane-spanning 4-domains, subfamily A, member 4D [Source:MGI Symbol;Acc:MGI:1913857] | yes|up |
| ENSMUSG00000023919 | Cenpq | centromere protein Q [Source:MGI Symbol;Acc:MGI:1933744] | yes|down |
| ENSMUSG00000108621 | Gm33989 | predicted gene, 33989 [Source:MGI Symbol;Acc:MGI:5593148] | yes|up |
| ENSMUSG00000051316 | Taf7 | TATA-box binding protein associated factor 7 [Source:MGI Symbol;Acc:MGI:1346348] | yes|down |
| ENSMUSG00000061540 | Orm2 | orosomucoid 2 [Source:MGI Symbol;Acc:MGI:97444] | yes|up |
| ENSMUSG00000049999 | Ppp1r3d | protein phosphatase 1, regulatory subunit 3D [Source:MGI Symbol;Acc:MGI:1917664] | yes|up |
| ENSMUSG00000046711 | Hmga1 | high mobility group AT-hook 1 [Source:MGI Symbol;Acc:MGI:96160] | yes|down |
| ENSMUSG00000046718 | Bst2 | bone marrow stromal cell antigen 2 [Source:MGI Symbol;Acc:MGI:1916800] | yes|up |
| ENSMUSG00000024136 | Dnase1l2 | deoxyribonuclease 1-like 2 [Source:MGI Symbol;Acc:MGI:1913955] | yes|up |
| ENSMUSG00000021457 | Syk | spleen tyrosine kinase [Source:MGI Symbol;Acc:MGI:99515] | yes|down |
| ENSMUSG00000044576 | Garem2 | GRB2 associated regulator of MAPK1 subtype 2 [Source:MGI Symbol;Acc:MGI:2685290] | yes|up |
| ENSMUSG00000038903 | Ccdc68 | coiled-coil domain containing 68 [Source:MGI Symbol;Acc:MGI:3612676] | yes|up |
| ENSMUSG00000042417 | Ccno | cyclin O [Source:MGI Symbol;Acc:MGI:2145534] | yes|down |
| ENSMUSG00000053560 | Ier2 | immediate early response 2 [Source:MGI Symbol;Acc:MGI:104815] | yes|down |
| ENSMUSG00000059810 | Rgs3 | regulator of G-protein signaling 3 [Source:MGI Symbol;Acc:MGI:1354734] | yes|up |
| ENSMUSG00000002769 | Gnmt | glycine N-methyltransferase [Source:MGI Symbol;Acc:MGI:1202304] | yes|up |
| ENSMUSG00000055926 | Gm14137 | predicted gene 14137 [Source:MGI Symbol;Acc:MGI:3651144] | yes|down |
| ENSMUSG00000117628 | Gm50012 | predicted gene, 50012 [Source:MGI Symbol;Acc:MGI:6275301] | yes|up |
| ENSMUSG00000002588 | Pon1 | paraoxonase 1 [Source:MGI Symbol;Acc:MGI:103295] | yes|up |
| ENSMUSG00000024795 | Kif20b | kinesin family member 20B [Source:MGI Symbol;Acc:MGI:2444576] | yes|down |
| ENSMUSG00000024791 | Cdca5 | cell division cycle associated 5 [Source:MGI Symbol;Acc:MGI:1915099] | yes|down |
| ENSMUSG00000079592 | C1qtnf5 | C1q and tumor necrosis factor related protein 5 [Source:MGI Symbol;Acc:MGI:2385958] | yes|up |
| ENSMUSG00000010607 | Pigyl | phosphatidylinositol glycan anchor biosynthesis, class Y-like [Source:MGI Symbol;Acc:MGI:1913518] | yes|up |
| ENSMUSG00000010601 | Apol7a | apolipoprotein L 7a [Source:MGI Symbol;Acc:MGI:1923011] | yes|up |
| ENSMUSG00000054034 | Tceal5 | transcription elongation factor A (SII)-like 5 [Source:MGI Symbol;Acc:MGI:3036236] | yes|up |
| ENSMUSG00000027115 | Kif18a | kinesin family member 18A [Source:MGI Symbol;Acc:MGI:2446977] | yes|down |
| ENSMUSG00000020325 | Fstl3 | follistatin-like 3 [Source:MGI Symbol;Acc:MGI:1890391] | yes|up |
| ENSMUSG00000121283 |  | novel transcript | yes|up |
| ENSMUSG00000054252 | Fgfr3 | fibroblast growth factor receptor 3 [Source:MGI Symbol;Acc:MGI:95524] | yes|up |
| ENSMUSG00000030731 | Syt3 | synaptotagmin III [Source:MGI Symbol;Acc:MGI:99665] | yes|up |
| ENSMUSG00000082016 | Pgam1-ps2 | phosphoglycerate mutase 1, pseudogene 2 [Source:MGI Symbol;Acc:MGI:3645709] | yes|up |
| ENSMUSG00000039617 | Gm7488 | predicted gene 7488 [Source:MGI Symbol;Acc:MGI:3647006] | yes|down |
| ENSMUSG00000050550 | Gm11868 | predicted gene 11868 [Source:MGI Symbol;Acc:MGI:3650507] | yes|down |
| ENSMUSG00000026249 | Serpine2 | serine (or cysteine) peptidase inhibitor, clade E, member 2 [Source:MGI Symbol;Acc:MGI:101780] | yes|up |
| ENSMUSG00000030844 | Rgs10 | regulator of G-protein signalling 10 [Source:MGI Symbol;Acc:MGI:1915115] | yes|down |
| ENSMUSG00000019775 | Rgs17 | regulator of G-protein signaling 17 [Source:MGI Symbol;Acc:MGI:1927469] | yes|down |
| ENSMUSG00000019772 | Vip | vasoactive intestinal polypeptide [Source:MGI Symbol;Acc:MGI:98933] | yes|up |
| ENSMUSG00000019773 | Fbxo5 | F-box protein 5 [Source:MGI Symbol;Acc:MGI:1914391] | yes|down |
| ENSMUSG00000115276 | 9930017N22Rik | RIKEN cDNA 9930017N22 gene [Source:MGI Symbol;Acc:MGI:2443961] | yes|down |
| ENSMUSG00000021214 | Akr1c18 | aldo-keto reductase family 1, member C18 [Source:MGI Symbol;Acc:MGI:2145420] | yes|down |
| ENSMUSG00000021210 | Akr1c6 | aldo-keto reductase family 1, member C6 [Source:MGI Symbol;Acc:MGI:1933427] | yes|up |
| ENSMUSG00000022090 | Pdlim2 | PDZ and LIM domain 2 [Source:MGI Symbol;Acc:MGI:2384850] | yes|down |
| ENSMUSG00000021219 | Rgs6 | regulator of G-protein signaling 6 [Source:MGI Symbol;Acc:MGI:1354730] | yes|down |
| ENSMUSG00000055782 | Abcd2 | ATP-binding cassette, sub-family D (ALD), member 2 [Source:MGI Symbol;Acc:MGI:1349467] | yes|up |
| ENSMUSG00000060961 | Slc4a4 | solute carrier family 4 (anion exchanger), member 4 [Source:MGI Symbol;Acc:MGI:1927555] | yes|up |
| ENSMUSG00000106961 | Gm43128 | predicted gene 43128 [Source:MGI Symbol;Acc:MGI:5663265] | yes|up |
| ENSMUSG00000026429 | Ube2t | ubiquitin-conjugating enzyme E2T [Source:MGI Symbol;Acc:MGI:1914446] | yes|down |
| ENSMUSG00000017176 | Nt5c3b | 5'-nucleotidase, cytosolic IIIB [Source:MGI Symbol;Acc:MGI:1915356] | yes|down |
| ENSMUSG00000012519 | Mlkl | mixed lineage kinase domain-like [Source:MGI Symbol;Acc:MGI:1921818] | yes|down |
| ENSMUSG00000005803 | Sqor | sulfide quinone oxidoreductase [Source:MGI Symbol;Acc:MGI:1929899] | yes|down |
| ENSMUSG00000005802 | Slc30a4 | solute carrier family 30 (zinc transporter), member 4 [Source:MGI Symbol;Acc:MGI:1345282] | yes|down |
| ENSMUSG00000118246 | Gm6052 | predicted gene 6052 [Source:MGI Symbol;Acc:MGI:3645585] | yes|down |
| ENSMUSG00000097819 | Gm26813 | predicted gene, 26813 [Source:MGI Symbol;Acc:MGI:5477307] | yes|down |
| ENSMUSG00000022231 | Sema5a | sema domain, seven thrombospondin repeats (type 1 and type 1-like), transmembrane domain (TM) and short cytoplasmic domain, (semaphorin) 5A [Source:MGI Symbol;Acc:MGI:107556] | yes|up |
| ENSMUSG00000022235 | Cmbl | carboxymethylenebutenolidase-like (Pseudomonas) [Source:MGI Symbol;Acc:MGI:1916824] | yes|up |
| ENSMUSG00000022548 | Apod | apolipoprotein D [Source:MGI Symbol;Acc:MGI:88056] | yes|up |
| ENSMUSG00000035615 | Frmpd1 | FERM and PDZ domain containing 1 [Source:MGI Symbol;Acc:MGI:2446274] | yes|up |
| ENSMUSG00000038224 | Serpinf2 | serine (or cysteine) peptidase inhibitor, clade F, member 2 [Source:MGI Symbol;Acc:MGI:107173] | yes|up |
| ENSMUSG00000046523 | Kctd4 | potassium channel tetramerisation domain containing 4 [Source:MGI Symbol;Acc:MGI:1914766] | yes|down |
| ENSMUSG00000072968 | Gm17728 | predicted gene, 17728 [Source:MGI Symbol;Acc:MGI:4937362] | yes|down |
| ENSMUSG00000072964 | Bhlhb9 | basic helix-loop-helix domain containing, class B9 [Source:MGI Symbol;Acc:MGI:1917487] | yes|down |
| ENSMUSG00000016458 | Wt1 | WT1 transcription factor [Source:MGI Symbol;Acc:MGI:98968] | yes|up |
| ENSMUSG00000069972 | Rps13-ps2 | ribosomal protein S13, pseudogene 2 [Source:MGI Symbol;Acc:MGI:3704295] | yes|down |
| ENSMUSG00000032348 | Gsta4 | glutathione S-transferase, alpha 4 [Source:MGI Symbol;Acc:MGI:1309515] | yes|down |
| ENSMUSG00000032344 | Cgas | cyclic GMP-AMP synthase [Source:MGI Symbol;Acc:MGI:2442261] | yes|down |
| ENSMUSG00000052316 | Lrrc15 | leucine rich repeat containing 15 [Source:MGI Symbol;Acc:MGI:1921738] | yes|up |
| ENSMUSG00000032690 | Oas2 | 2'-5' oligoadenylate synthetase 2 [Source:MGI Symbol;Acc:MGI:2180852] | yes|up |
| ENSMUSG00000037921 | Ddx60 | DExD/H box helicase 60 [Source:MGI Symbol;Acc:MGI:2384570] | yes|up |
| ENSMUSG00000027327 | 1700037H04Rik | RIKEN cDNA 1700037H04 gene [Source:MGI Symbol;Acc:MGI:1914576] | yes|down |
| ENSMUSG00000037820 | Tgm2 | transglutaminase 2, C polypeptide [Source:MGI Symbol;Acc:MGI:98731] | yes|up |
| ENSMUSG00000021846 | Peli2 | pellino 2 [Source:MGI Symbol;Acc:MGI:1891445] | yes|down |
| ENSMUSG00000078853 | Igtp | interferon gamma induced GTPase [Source:MGI Symbol;Acc:MGI:107729] | yes|up |
| ENSMUSG00000017499 | Cdc6 | cell division cycle 6 [Source:MGI Symbol;Acc:MGI:1345150] | yes|down |
| ENSMUSG00000058207 | Serpina3k | serine (or cysteine) peptidase inhibitor, clade A, member 3K [Source:MGI Symbol;Acc:MGI:98377] | yes|up |
| ENSMUSG00000017493 | Igfbp4 | insulin-like growth factor binding protein 4 [Source:MGI Symbol;Acc:MGI:96439] | yes|down |
| ENSMUSG00000024301 | Kifc5b | kinesin family member C5B [Source:MGI Symbol;Acc:MGI:2137414] | yes|down |
| ENSMUSG00000024659 | Anxa1 | annexin A1 [Source:MGI Symbol;Acc:MGI:96819] | yes|down |
| ENSMUSG00000024308 | Tapbp | TAP binding protein [Source:MGI Symbol;Acc:MGI:1201689] | yes|up |
| ENSMUSG00000044033 | Ccdc141 | coiled-coil domain containing 141 [Source:MGI Symbol;Acc:MGI:1919735] | yes|up |
| ENSMUSG00000026655 | Fam107b | family with sequence similarity 107, member B [Source:MGI Symbol;Acc:MGI:1913790] | yes|down |
| ENSMUSG00000097651 | 4930461G14Rik | RIKEN cDNA 4930461G14 gene [Source:MGI Symbol;Acc:MGI:1922129] | yes|down |
| ENSMUSG00000030693 | Klk10 | kallikrein related-peptidase 10 [Source:MGI Symbol;Acc:MGI:1916790] | yes|down |
| ENSMUSG00000059555 | Tor4a | torsin family 4, member A [Source:MGI Symbol;Acc:MGI:2442720] | yes|down |
| ENSMUSG00000036446 | Lum | lumican [Source:MGI Symbol;Acc:MGI:109347] | yes|up |
| ENSMUSG00000096385 | Gm11168 | predicted gene 11168 [Source:MGI Symbol;Acc:MGI:3779420] | yes|up |
| ENSMUSG00000036334 | Igsf10 | immunoglobulin superfamily, member 10 [Source:MGI Symbol;Acc:MGI:1923481] | yes|up |
| ENSMUSG00000047604 | Frat2 | frequently rearranged in advanced T cell lymphomas 2 [Source:MGI Symbol;Acc:MGI:2673967] | yes|down |
| ENSMUSG00000037313 | Tacc3 | transforming, acidic coiled-coil containing protein 3 [Source:MGI Symbol;Acc:MGI:1341163] | yes|down |
| ENSMUSG00000027656 | Ccn5 | cellular communication network factor 5 [Source:MGI Symbol;Acc:MGI:1328326] | yes|up |
| ENSMUSG00000027306 | Nusap1 | nucleolar and spindle associated protein 1 [Source:MGI Symbol;Acc:MGI:2675669] | yes|down |
| ENSMUSG00000027875 | Hmgcs2 | 3-hydroxy-3-methylglutaryl-Coenzyme A synthase 2 [Source:MGI Symbol;Acc:MGI:101939] | yes|up |
| ENSMUSG00000121345 | 2610005L07Rik | cadherin 11 pseudogene [Source:NCBI gene (formerly Entrezgene);Acc:381598] | yes|up |
| ENSMUSG00000031877 | Ces2g | carboxylesterase 2G [Source:MGI Symbol;Acc:MGI:1919611] | yes|down |
| ENSMUSG00000031870 | Pgr | progesterone receptor [Source:MGI Symbol;Acc:MGI:97567] | yes|up |
| ENSMUSG00000090381 | Gm6158 | predicted gene 6158 [Source:MGI Symbol;Acc:MGI:3779562] | yes|down |
| ENSMUSG00000021222 | Dcaf4 | DDB1 and CUL4 associated factor 4 [Source:MGI Symbol;Acc:MGI:1921078] | yes|down |
| ENSMUSG00000096929 | A330023F24Rik | RIKEN cDNA A330023F24 gene [Source:MGI Symbol;Acc:MGI:2443958] | yes|up |
| ENSMUSG00000031253 | Srpx2 | sushi-repeat-containing protein, X-linked 2 [Source:MGI Symbol;Acc:MGI:1916042] | yes|up |
| ENSMUSG00000031250 | Tnmd | tenomodulin [Source:MGI Symbol;Acc:MGI:1929885] | yes|up |
| ENSMUSG00000095865 | Gm13237 | predicted gene 13237 [Source:MGI Symbol;Acc:MGI:3649924] | yes|down |
| ENSMUSG00000040734 | Ppp1r13l | protein phosphatase 1, regulatory subunit 13 like [Source:MGI Symbol;Acc:MGI:3525053] | yes|up |
| ENSMUSG00000040732 | Erg | ETS transcription factor [Source:MGI Symbol;Acc:MGI:95415] | yes|up |
| ENSMUSG00000046959 | Slc26a1 | solute carrier family 26 (sulfate transporter), member 1 [Source:MGI Symbol;Acc:MGI:2385894] | yes|up |
| ENSMUSG00000043122 | A530016L24Rik | RIKEN cDNA A530016L24 gene [Source:MGI Symbol;Acc:MGI:2443020] | yes|down |
| ENSMUSG00000039405 | Prss23 | protease, serine 23 [Source:MGI Symbol;Acc:MGI:1923703] | yes|up |
| ENSMUSG00000040289 | Hey1 | hairy/enhancer-of-split related with YRPW motif 1 [Source:MGI Symbol;Acc:MGI:1341800] | yes|up |
| ENSMUSG00000034755 | Pcdh11x | protocadherin 11 X-linked [Source:MGI Symbol;Acc:MGI:2442849] | yes|up |
| ENSMUSG00000034206 | Polq | polymerase (DNA directed), theta [Source:MGI Symbol;Acc:MGI:2155399] | yes|down |
| ENSMUSG00000034205 | Loxl2 | lysyl oxidase-like 2 [Source:MGI Symbol;Acc:MGI:2137913] | yes|up |
| ENSMUSG00000034758 | Tle6 | transducin-like enhancer of split 6 [Source:MGI Symbol;Acc:MGI:2149593] | yes|down |
| ENSMUSG00000074622 | Mafb | v-maf musculoaponeurotic fibrosarcoma oncogene family, protein B (avian) [Source:MGI Symbol;Acc:MGI:104555] | yes|up |
| ENSMUSG00000029561 | Oasl2 | 2'-5' oligoadenylate synthetase-like 2 [Source:MGI Symbol;Acc:MGI:1344390] | yes|up |
| ENSMUSG00000029563 | Foxp2 | forkhead box P2 [Source:MGI Symbol;Acc:MGI:2148705] | yes|up |
| ENSMUSG00000042659 | Arrdc4 | arrestin domain containing 4 [Source:MGI Symbol;Acc:MGI:1913662] | yes|up |
| ENSMUSG00000028967 | Errfi1 | ERBB receptor feedback inhibitor 1 [Source:MGI Symbol;Acc:MGI:1921405] | yes|down |
| ENSMUSG00000085781 | Gm15640 | predicted gene 15640 [Source:MGI Symbol;Acc:MGI:3783084] | yes|up |
| ENSMUSG00000085786 | Gm15987 | predicted gene 15987 [Source:MGI Symbol;Acc:MGI:3801849] | yes|down |
| ENSMUSG00000085787 | Gm13092 | predicted gene 13092 [Source:MGI Symbol;Acc:MGI:3649661] | yes|down |
| ENSMUSG00000017390 | Aldoc | aldolase C, fructose-bisphosphate [Source:MGI Symbol;Acc:MGI:101863] | yes|up |
| ENSMUSG00000068101 | Cenpm | centromere protein M [Source:MGI Symbol;Acc:MGI:1913820] | yes|down |
| ENSMUSG00000120992 |  | novel transcript | yes|down |
| ENSMUSG00000120994 |  | novel transcript, sense intronic to Rbm25 | yes|down |
| ENSMUSG00000042476 | Abcb4 | ATP-binding cassette, sub-family B (MDR/TAP), member 4 [Source:MGI Symbol;Acc:MGI:97569] | yes|up |
| ENSMUSG00000035158 | Mitf | melanogenesis associated transcription factor [Source:MGI Symbol;Acc:MGI:104554] | yes|down |
| ENSMUSG00000020774 | Aspa | aspartoacylase [Source:MGI Symbol;Acc:MGI:87914] | yes|down |
| ENSMUSG00000042389 | Tsen2 | tRNA splicing endonuclease subunit 2 [Source:MGI Symbol;Acc:MGI:2141599] | yes|down |
| ENSMUSG00000028212 | Ccne2 | cyclin E2 [Source:MGI Symbol;Acc:MGI:1329034] | yes|down |
| ENSMUSG00000100642 | Gm28230 | predicted gene 28230 [Source:MGI Symbol;Acc:MGI:5578936] | yes|up |
| ENSMUSG00000078190 | Dnm3os | dynamin 3, opposite strand [Source:MGI Symbol;Acc:MGI:3052332] | yes|up |
| ENSMUSG00000019970 | Sgk1 | serum/glucocorticoid regulated kinase 1 [Source:MGI Symbol;Acc:MGI:1340062] | yes|down |
| ENSMUSG00000067818 | Myl9 | myosin, light polypeptide 9, regulatory [Source:MGI Symbol;Acc:MGI:2138915] | yes|up |
| ENSMUSG00000114470 | Gm49395 | predicted gene, 49395 [Source:MGI Symbol;Acc:MGI:6121627] | yes|up |
| ENSMUSG00000070407 | Hs3st3b1 | heparan sulfate (glucosamine) 3-O-sulfotransferase 3B1 [Source:MGI Symbol;Acc:MGI:1333853] | yes|up |
| ENSMUSG00000027469 | Tpx2 | TPX2, microtubule-associated [Source:MGI Symbol;Acc:MGI:1919369] | yes|down |
| ENSMUSG00000101599 | Gm20342 | predicted gene, 20342 [Source:MGI Symbol;Acc:MGI:5012527] | yes|up |
| ENSMUSG00000044734 | Serpinb1a | serine (or cysteine) peptidase inhibitor, clade B, member 1a [Source:MGI Symbol;Acc:MGI:1913472] | yes|up |
| ENSMUSG00000078994 | Zfp429 | zinc finger protein 429 [Source:MGI Symbol;Acc:MGI:1920057] | yes|down |
| ENSMUSG00000027796 | Smad9 | SMAD family member 9 [Source:MGI Symbol;Acc:MGI:1859993] | yes|down |
| ENSMUSG00000029012 | Orc5 | origin recognition complex, subunit 5 [Source:MGI Symbol;Acc:MGI:1347044] | yes|down |
| ENSMUSG00000039671 | Zmynd8 | zinc finger, MYND-type containing 8 [Source:MGI Symbol;Acc:MGI:1918025] | yes|down |
| ENSMUSG00000024512 | Dynap | dynactin associated protein [Source:MGI Symbol;Acc:MGI:1922827] | yes|down |
| ENSMUSG00000024846 | Cst6 | cystatin E/M [Source:MGI Symbol;Acc:MGI:1920970] | yes|down |
| ENSMUSG00000030867 | Plk1 | polo like kinase 1 [Source:MGI Symbol;Acc:MGI:97621] | yes|down |
| ENSMUSG00000026227 | 2810459M11Rik | RIKEN cDNA 2810459M11 gene [Source:MGI Symbol;Acc:MGI:1920042] | yes|up |
| ENSMUSG00000030862 | Cpxm2 | carboxypeptidase X 2 (M14 family) [Source:MGI Symbol;Acc:MGI:1926006] | yes|up |
| ENSMUSG00000024371 | C2 | complement component 2 (within H-2S) [Source:MGI Symbol;Acc:MGI:88226] | yes|up |
| ENSMUSG00000073421 | H2-Ab1 | histocompatibility 2, class II antigen A, beta 1 [Source:MGI Symbol;Acc:MGI:103070] | yes|up |
| ENSMUSG00000000301 | Pemt | phosphatidylethanolamine N-methyltransferase [Source:MGI Symbol;Acc:MGI:104535] | yes|down |
| ENSMUSG00000000305 | Cdh4 | cadherin 4 [Source:MGI Symbol;Acc:MGI:99218] | yes|up |
| ENSMUSG00000031403 | Dkc1 | dyskeratosis congenita 1, dyskerin [Source:MGI Symbol;Acc:MGI:1861727] | yes|down |
| ENSMUSG00000049382 | Krt8 | keratin 8 [Source:MGI Symbol;Acc:MGI:96705] | yes|down |
| ENSMUSG00000050578 | Mmp13 | matrix metallopeptidase 13 [Source:MGI Symbol;Acc:MGI:1340026] | yes|up |
| ENSMUSG00000042248 | Cyp2c37 | cytochrome P450, family 2. subfamily c, polypeptide 37 [Source:MGI Symbol;Acc:MGI:1306806] | yes|up |
| ENSMUSG00000042246 | Tmc7 | transmembrane channel-like gene family 7 [Source:MGI Symbol;Acc:MGI:2443317] | yes|up |
| ENSMUSG00000051220 | Ercc6l | excision repair cross-complementing rodent repair deficiency complementation group 6 like [Source:MGI Symbol;Acc:MGI:2654144] | yes|down |
| ENSMUSG00000029022 | Miip | migration and invasion inhibitory protein [Source:MGI Symbol;Acc:MGI:106506] | yes|down |
| ENSMUSG00000047415 | Gpr68 | G protein-coupled receptor 68 [Source:MGI Symbol;Acc:MGI:2441763] | yes|down |
| ENSMUSG00000047419 | Cmya5 | cardiomyopathy associated 5 [Source:MGI Symbol;Acc:MGI:1923719] | yes|up |
| ENSMUSG00000038203 | Hoxa13 | homeobox A13 [Source:MGI Symbol;Acc:MGI:96173] | yes|down |
| ENSMUSG00000035365 | Parpbp | PARP1 binding protein [Source:MGI Symbol;Acc:MGI:1922567] | yes|down |
| ENSMUSG00000028773 | Fabp3 | fatty acid binding protein 3, muscle and heart [Source:MGI Symbol;Acc:MGI:95476] | yes|up |
| ENSMUSG00000022219 | Cideb | cell death-inducing DNA fragmentation factor, alpha subunit-like effector B [Source:MGI Symbol;Acc:MGI:1270844] | yes|up |
| ENSMUSG00000025075 | Habp2 | hyaluronic acid binding protein 2 [Source:MGI Symbol;Acc:MGI:1196378] | yes|up |
| ENSMUSG00000048521 | Cxcr6 | chemokine (C-X-C motif) receptor 6 [Source:MGI Symbol;Acc:MGI:1934582] | yes|down |
| ENSMUSG00000043243 | Niban3 | niban apoptosis regulator 3 [Source:MGI Symbol;Acc:MGI:3686743] | yes|up |
| ENSMUSG00000052085 | Dock8 | dedicator of cytokinesis 8 [Source:MGI Symbol;Acc:MGI:1921396] | yes|down |
| ENSMUSG00000013155 | Enkd1 | enkurin domain containing 1 [Source:MGI Symbol;Acc:MGI:2142593] | yes|down |
| ENSMUSG00000103509 | Gm38372 | predicted gene, 38372 [Source:MGI Symbol;Acc:MGI:5611600] | yes|up |
| ENSMUSG00000042041 | 2010003K11Rik | RIKEN cDNA 2010003K11 gene [Source:MGI Symbol;Acc:MGI:1917111] | yes|up |
| ENSMUSG00000117251 | Gm49891 | predicted gene, 49891 [Source:MGI Symbol;Acc:MGI:6270580] | yes|down |
| ENSMUSG00000034413 | Neurl1b | neuralized E3 ubiquitin protein ligase 1B [Source:MGI Symbol;Acc:MGI:3643092] | yes|up |
| ENSMUSG00000052331 | Ankrd44 | ankyrin repeat domain 44 [Source:MGI Symbol;Acc:MGI:3045243] | yes|up |
| ENSMUSG00000020875 | Hoxb9 | homeobox B9 [Source:MGI Symbol;Acc:MGI:96190] | yes|up |
| ENSMUSG00000037940 | Inpp4b | inositol polyphosphate-4-phosphatase, type II [Source:MGI Symbol;Acc:MGI:2158925] | yes|down |
| ENSMUSG00000037942 | Crp | C-reactive protein, pentraxin-related [Source:MGI Symbol;Acc:MGI:88512] | yes|up |
| ENSMUSG00000059991 | Nptx2 | neuronal pentraxin 2 [Source:MGI Symbol;Acc:MGI:1858209] | yes|up |
| ENSMUSG00000061353 | Cxcl12 | chemokine (C-X-C motif) ligand 12 [Source:MGI Symbol;Acc:MGI:103556] | yes|up |
| ENSMUSG00000021822 | Plau | plasminogen activator, urokinase [Source:MGI Symbol;Acc:MGI:97611] | yes|down |
| ENSMUSG00000034777 | Vax2 | ventral anterior homeobox 2 [Source:MGI Symbol;Acc:MGI:1346018] | yes|up |
| ENSMUSG00000062611 | Rps3a2 | ribosomal protein S3A2 [Source:MGI Symbol;Acc:MGI:3642853] | yes|down |
| ENSMUSG00000050714 | Zbtb26 | zinc finger and BTB domain containing 26 [Source:MGI Symbol;Acc:MGI:2444402] | yes|up |
| ENSMUSG00000019564 | Arid3a | AT rich interactive domain 3A (BRIGHT-like) [Source:MGI Symbol;Acc:MGI:1328360] | yes|up |
| ENSMUSG00000078878 | Gm14305 | predicted gene 14305 [Source:MGI Symbol;Acc:MGI:3709632] | yes|down |
| ENSMUSG00000078879 | Zfp973 | zinc finger protein 973 [Source:MGI Symbol;Acc:MGI:3615331] | yes|down |
| ENSMUSG00000024379 | Tslp | thymic stromal lymphopoietin [Source:MGI Symbol;Acc:MGI:1855696] | yes|down |
| ENSMUSG00000033327 | Tnxb | tenascin XB [Source:MGI Symbol;Acc:MGI:1932137] | yes|down |
| ENSMUSG00000032815 | Fanca | Fanconi anemia, complementation group A [Source:MGI Symbol;Acc:MGI:1341823] | yes|down |
| ENSMUSG00000030671 | Pde3b | phosphodiesterase 3B, cGMP-inhibited [Source:MGI Symbol;Acc:MGI:1333863] | yes|down |
| ENSMUSG00000030677 | Kif22 | kinesin family member 22 [Source:MGI Symbol;Acc:MGI:109233] | yes|down |
| ENSMUSG00000030674 | Qprt | quinolinate phosphoribosyltransferase [Source:MGI Symbol;Acc:MGI:1914625] | yes|up |
| ENSMUSG00000022853 | Ehhadh | enoyl-Coenzyme A, hydratase/3-hydroxyacyl Coenzyme A dehydrogenase [Source:MGI Symbol;Acc:MGI:1277964] | yes|up |
| ENSMUSG00000037379 | Spon2 | spondin 2, extracellular matrix protein [Source:MGI Symbol;Acc:MGI:1923724] | yes|down |
| ENSMUSG00000027326 | Knl1 | kinetochore scaffold 1 [Source:MGI Symbol;Acc:MGI:1923714] | yes|down |
| ENSMUSG00000027323 | Rad51 | RAD51 recombinase [Source:MGI Symbol;Acc:MGI:97890] | yes|down |
| ENSMUSG00000041481 | Serpina3g | serine (or cysteine) peptidase inhibitor, clade A, member 3G [Source:MGI Symbol;Acc:MGI:105046] | yes|up |
| ENSMUSG00000041482 | Piezo2 | piezo-type mechanosensitive ion channel component 2 [Source:MGI Symbol;Acc:MGI:1918781] | yes|up |
| ENSMUSG00000040280 | Ndufa4l2 | Ndufa4, mitochondrial complex associated like 2 [Source:MGI Symbol;Acc:MGI:3039567] | yes|up |
| ENSMUSG00000040282 | Cdin1 | CDAN1 interacting nuclease 1 [Source:MGI Symbol;Acc:MGI:3026886] | yes|down |
| ENSMUSG00000039795 | Zfand1 | zinc finger, AN1-type domain 1 [Source:MGI Symbol;Acc:MGI:1913611] | yes|down |
| ENSMUSG00000031239 | Itm2a | integral membrane protein 2A [Source:MGI Symbol;Acc:MGI:107706] | yes|up |
| ENSMUSG00000027347 | Rasgrp1 | RAS guanyl releasing protein 1 [Source:MGI Symbol;Acc:MGI:1314635] | yes|up |
| ENSMUSG00000024742 | Fen1 | flap structure specific endonuclease 1 [Source:MGI Symbol;Acc:MGI:102779] | yes|down |
| ENSMUSG00000009654 | Oit3 | oncoprotein induced transcript 3 [Source:MGI Symbol;Acc:MGI:1201782] | yes|up |
| ENSMUSG00000022667 | Cd200r1 | CD200 receptor 1 [Source:MGI Symbol;Acc:MGI:1889024] | yes|up |
| ENSMUSG00000022665 | Ccdc80 | coiled-coil domain containing 80 [Source:MGI Symbol;Acc:MGI:1915146] | yes|up |
| ENSMUSG00000057359 | Gm17494 | predicted gene, 17494 [Source:MGI Symbol;Acc:MGI:4937128] | yes|down |
| ENSMUSG00000097993 | Ptprv | protein tyrosine phosphatase, receptor type, V [Source:MGI Symbol;Acc:MGI:108027] | yes|up |
| ENSMUSG00000034773 | Hrob | homologous recombination factor with OB-fold [Source:MGI Symbol;Acc:MGI:2387601] | yes|down |
| ENSMUSG00000023951 | Vegfa | vascular endothelial growth factor A [Source:MGI Symbol;Acc:MGI:103178] | yes|up |
| ENSMUSG00000095597 | Rps7-ps3 | ribosomal protein S7, pseudogene 3 [Source:MGI Symbol;Acc:MGI:3648396] | yes|down |
| ENSMUSG00000047881 | Rell1 | RELT-like 1 [Source:MGI Symbol;Acc:MGI:2140767] | yes|up |
| ENSMUSG00000028989 | Angptl7 | angiopoietin-like 7 [Source:MGI Symbol;Acc:MGI:3605801] | yes|down |
| ENSMUSG00000028988 | Ctnnbip1 | catenin beta interacting protein 1 [Source:MGI Symbol;Acc:MGI:1915756] | yes|down |
| ENSMUSG00000045954 | Cavin2 | caveolae associated 2 [Source:MGI Symbol;Acc:MGI:99513] | yes|down |

**Supplementary Table 1-2. The list of altered genes as the result of G: profiler analysis in *Kras^G12D^* cells compared with selected *Cbx4^-/-^, Kras^G12D^* cells.**

| Gene id | Gene name | Gene description | Kras^G12D^_vs_Selected |
| --- | --- | --- | --- |
| ENSMUSG00000104235 | Gm37589 | predicted gene, 37589 [Source:MGI Symbol;Acc:MGI:5610817] | yes|up |
| ENSMUSG00000053214 | Gm9899 | predicted gene 9899 [Source:MGI Symbol;Acc:MGI:3708711] | yes|down |
| ENSMUSG00000028188 | Spata1 | spermatogenesis associated 1 [Source:MGI Symbol;Acc:MGI:1918201] | yes|down |
| ENSMUSG00000053219 | Raet1e | retinoic acid early transcript 1E [Source:MGI Symbol;Acc:MGI:2675273] | yes|down |
| ENSMUSG00000000365 | Rnf17 | ring finger protein 17 [Source:MGI Symbol;Acc:MGI:1353419] | yes|up |
| ENSMUSG00000075394 | Hoxc4 | homeobox C4 [Source:MGI Symbol;Acc:MGI:96195] | yes|down |
| ENSMUSG00000038893 | Fam117a | family with sequence similarity 117, member A [Source:MGI Symbol;Acc:MGI:2144564] | yes|down |
| ENSMUSG00000004891 | Nes | nestin [Source:MGI Symbol;Acc:MGI:101784] | yes|down |
| ENSMUSG00000046027 | Stard5 | StAR-related lipid transfer (START) domain containing 5 [Source:MGI Symbol;Acc:MGI:2156765] | yes|up |
| ENSMUSG00000038894 | Irs2 | insulin receptor substrate 2 [Source:MGI Symbol;Acc:MGI:109334] | yes|down |
| ENSMUSG00000028436 | Dcaf12 | DDB1 and CUL4 associated factor 12 [Source:MGI Symbol;Acc:MGI:1916220] | yes|up |
| ENSMUSG00000041261 | Car8 | carbonic anhydrase 8 [Source:MGI Symbol;Acc:MGI:88253] | yes|up |
| ENSMUSG00000018372 | Cep95 | centrosomal protein 95 [Source:MGI Symbol;Acc:MGI:2443502] | yes|down |
| ENSMUSG00000120330 |  | novel transcript | yes|up |
| ENSMUSG00000047907 | Tshz2 | teashirt zinc finger family member 2 [Source:MGI Symbol;Acc:MGI:2153084] | yes|up |
| ENSMUSG00000034957 | Cebpa | CCAAT/enhancer binding protein (C/EBP), alpha [Source:MGI Symbol;Acc:MGI:99480] | yes|up |
| ENSMUSG00000049866 | Arl4c | ADP-ribosylation factor-like 4C [Source:MGI Symbol;Acc:MGI:2445172] | yes|down |
| ENSMUSG00000056888 | Glipr1 | GLI pathogenesis-related 1 (glioma) [Source:MGI Symbol;Acc:MGI:1920940] | yes|up |
| ENSMUSG00000052632 | Asap2 | ArfGAP with SH3 domain, ankyrin repeat and PH domain 2 [Source:MGI Symbol;Acc:MGI:2685438] | yes|down |
| ENSMUSG00000042453 | Reln | reelin [Source:MGI Symbol;Acc:MGI:103022] | yes|up |
| ENSMUSG00000032332 | Col12a1 | collagen, type XII, alpha 1 [Source:MGI Symbol;Acc:MGI:88448] | yes|up |
| ENSMUSG00000023885 | Thbs2 | thrombospondin 2 [Source:MGI Symbol;Acc:MGI:98738] | yes|up |
| ENSMUSG00000023886 | Smoc2 | SPARC related modular calcium binding 2 [Source:MGI Symbol;Acc:MGI:1929881] | yes|down |
| ENSMUSG00000020758 | Itgb4 | integrin beta 4 [Source:MGI Symbol;Acc:MGI:96613] | yes|down |
| ENSMUSG00000046598 | Bdh1 | 3-hydroxybutyrate dehydrogenase, type 1 [Source:MGI Symbol;Acc:MGI:1919161] | yes|up |
| ENSMUSG00000021569 | Trip13 | thyroid hormone receptor interactor 13 [Source:MGI Symbol;Acc:MGI:1916966] | yes|down |
| ENSMUSG00000033863 | Klf9 | Kruppel-like factor 9 [Source:MGI Symbol;Acc:MGI:1333856] | yes|up |
| ENSMUSG00000028232 | Tmem68 | transmembrane protein 68 [Source:MGI Symbol;Acc:MGI:1919348] | yes|up |
| ENSMUSG00000045136 | Tubb2b | tubulin, beta 2B class IIB [Source:MGI Symbol;Acc:MGI:1920960] | yes|up |
| ENSMUSG00000074364 | Ehd2 | EH-domain containing 2 [Source:MGI Symbol;Acc:MGI:2154274] | yes|up |
| ENSMUSG00000021567 | Nkd2 | naked cuticle 2 [Source:MGI Symbol;Acc:MGI:1919543] | yes|up |
| ENSMUSG00000052852 | Reep1 | receptor accessory protein 1 [Source:MGI Symbol;Acc:MGI:1098827] | yes|down |
| ENSMUSG00000052854 | Nrk | Nik related kinase [Source:MGI Symbol;Acc:MGI:1351326] | yes|up |
| ENSMUSG00000044447 | Dock5 | dedicator of cytokinesis 5 [Source:MGI Symbol;Acc:MGI:2652871] | yes|up |
| ENSMUSG00000068015 | Lrch1 | leucine-rich repeats and calponin homology (CH) domain containing 1 [Source:MGI Symbol;Acc:MGI:2443390] | yes|up |
| ENSMUSG00000035042 | Ccl5 | chemokine (C-C motif) ligand 5 [Source:MGI Symbol;Acc:MGI:98262] | yes|down |
| ENSMUSG00000008690 | Ncaph2 | non-SMC condensin II complex, subunit H2 [Source:MGI Symbol;Acc:MGI:1289164] | yes|down |
| ENSMUSG00000103088 | Pcdhgb6 | protocadherin gamma subfamily B, 6 [Source:MGI Symbol;Acc:MGI:1935197] | yes|up |
| ENSMUSG00000044533 | Rps2 | ribosomal protein S2 [Source:MGI Symbol;Acc:MGI:105110] | yes|down |
| ENSMUSG00000017734 | Dbndd2 | dysbindin (dystrobrevin binding protein 1) domain containing 2 [Source:MGI Symbol;Acc:MGI:106562] | yes|up |
| ENSMUSG00000045822 | Zswim3 | zinc finger SWIM-type containing 3 [Source:MGI Symbol;Acc:MGI:1914788] | yes|down |
| ENSMUSG00000032023 | Jhy | junctional cadherin complex regulator [Source:MGI Symbol;Acc:MGI:1918239] | yes|down |
| ENSMUSG00000017737 | Mmp9 | matrix metallopeptidase 9 [Source:MGI Symbol;Acc:MGI:97011] | yes|down |
| ENSMUSG00000110386 | Gm42031 | predicted gene, 42031 [Source:MGI Symbol;Acc:MGI:5624916] | yes|down |
| ENSMUSG00000019917 | Septin10 | septin 10 [Source:MGI Symbol;Acc:MGI:1918110] | yes|down |
| ENSMUSG00000078606 | Gvin2 | GTPase, very large interferon inducible, family member 2 [Source:MGI Symbol;Acc:MGI:3782245] | yes|up |
| ENSMUSG00000036030 | Prtg | protogenin [Source:MGI Symbol;Acc:MGI:2444710] | yes|down |
| ENSMUSG00000054675 | Tmem119 | transmembrane protein 119 [Source:MGI Symbol;Acc:MGI:2385228] | yes|up |
| ENSMUSG00000054676 | 1600014C10Rik | RIKEN cDNA 1600014C10 gene [Source:MGI Symbol;Acc:MGI:1919494] | yes|up |
| ENSMUSG00000071478 | H2ac7 | H2A clustered histone 7 [Source:MGI Symbol;Acc:MGI:2448289] | yes|down |
| ENSMUSG00000074166 | AW146154 | expressed sequence AW146154 [Source:MGI Symbol;Acc:MGI:2142212] | yes|up |
| ENSMUSG00000117748 | Derpc | DERPC proline and glycine rich nuclear protein [Source:MGI Symbol;Acc:MGI:6303050] | yes|down |
| ENSMUSG00000093985 | Gm10406 | predicted gene 10406 [Source:MGI Symbol;Acc:MGI:3711272] | yes|up |
| ENSMUSG00000021765 | Fst | follistatin [Source:MGI Symbol;Acc:MGI:95586] | yes|up |
| ENSMUSG00000028437 | Ubap1 | ubiquitin-associated protein 1 [Source:MGI Symbol;Acc:MGI:2149543] | yes|up |
| ENSMUSG00000054580 | Pla2r1 | phospholipase A2 receptor 1 [Source:MGI Symbol;Acc:MGI:102468] | yes|up |
| ENSMUSG00000020362 | Cnot6 | CCR4-NOT transcription complex, subunit 6 [Source:MGI Symbol;Acc:MGI:2144529] | yes|down |
| ENSMUSG00000087377 | AV099323 | expressed sequence AV099323 [Source:MGI Symbol;Acc:MGI:2139252] | yes|down |
| ENSMUSG00000038895 | Zfp653 | zinc finger protein 653 [Source:MGI Symbol;Acc:MGI:2442362] | yes|down |
| ENSMUSG00000062044 | Lmtk3 | lemur tyrosine kinase 3 [Source:MGI Symbol;Acc:MGI:3039582] | yes|up |
| ENSMUSG00000038685 | Rtel1 | regulator of telomere elongation helicase 1 [Source:MGI Symbol;Acc:MGI:2139369] | yes|down |
| ENSMUSG00000121137 |  | novel transcript, antisense to Slc7a15 | yes|up |
| ENSMUSG00000031391 | L1cam | L1 cell adhesion molecule [Source:MGI Symbol;Acc:MGI:96721] | yes|up |
| ENSMUSG00000001630 | Stk38l | serine/threonine kinase 38 like [Source:MGI Symbol;Acc:MGI:1922250] | yes|up |
| ENSMUSG00000113388 | Gm48111 | predicted gene, 48111 [Source:MGI Symbol;Acc:MGI:6097464] | yes|up |
| ENSMUSG00000078862 | Gm14326 | predicted gene 14326 [Source:MGI Symbol;Acc:MGI:3709298] | yes|up |
| ENSMUSG00000020305 | Asb3 | ankyrin repeat and SOCS box-containing 3 [Source:MGI Symbol;Acc:MGI:1929749] | yes|down |
| ENSMUSG00000078867 | Gm14418 | predicted gene 14418 [Source:MGI Symbol;Acc:MGI:3702408] | yes|up |
| ENSMUSG00000002870 | Mcm2 | minichromosome maintenance complex component 2 [Source:MGI Symbol;Acc:MGI:105380] | yes|down |
| ENSMUSG00000015468 | Notch4 | notch 4 [Source:MGI Symbol;Acc:MGI:107471] | yes|down |
| ENSMUSG00000015243 | Abca1 | ATP-binding cassette, sub-family A (ABC1), member 1 [Source:MGI Symbol;Acc:MGI:99607] | yes|up |
| ENSMUSG00000032249 | Anp32a | acidic (leucine-rich) nuclear phosphoprotein 32 family, member A [Source:MGI Symbol;Acc:MGI:108447] | yes|down |
| ENSMUSG00000021969 | Zdhhc20 | zinc finger, DHHC domain containing 20 [Source:MGI Symbol;Acc:MGI:1923215] | yes|up |
| ENSMUSG00000024190 | Dusp1 | dual specificity phosphatase 1 [Source:MGI Symbol;Acc:MGI:105120] | yes|up |
| ENSMUSG00000021963 | Sap18 | Sin3-associated polypeptide 18 [Source:MGI Symbol;Acc:MGI:1277978] | yes|up |
| ENSMUSG00000060240 | Cend1 | cell cycle exit and neuronal differentiation 1 [Source:MGI Symbol;Acc:MGI:1929898] | yes|up |
| ENSMUSG00000037605 | Adgrl3 | adhesion G protein-coupled receptor L3 [Source:MGI Symbol;Acc:MGI:2441950] | yes|up |
| ENSMUSG00000001270 | Ckb | creatine kinase, brain [Source:MGI Symbol;Acc:MGI:88407] | yes|up |
| ENSMUSG00000049001 | Ndnf | neuron-derived neurotrophic factor [Source:MGI Symbol;Acc:MGI:1915419] | yes|up |
| ENSMUSG00000031398 | Plxna3 | plexin A3 [Source:MGI Symbol;Acc:MGI:107683] | yes|down |
| ENSMUSG00000006958 | Chrd | chordin [Source:MGI Symbol;Acc:MGI:1313268] | yes|up |
| ENSMUSG00000063594 | Gng8 | guanine nucleotide binding protein (G protein), gamma 8 [Source:MGI Symbol;Acc:MGI:109163] | yes|up |
| ENSMUSG00000117959 | D330050I16Rik | RIKEN cDNA D330050I16 gene [Source:MGI Symbol;Acc:MGI:3041222] | yes|down |
| ENSMUSG00000031250 | Tnmd | tenomodulin [Source:MGI Symbol;Acc:MGI:1929885] | yes|up |
| ENSMUSG00000096225 | Lhx8 | LIM homeobox protein 8 [Source:MGI Symbol;Acc:MGI:1096343] | yes|up |
| ENSMUSG00000030921 | Trim30a | tripartite motif-containing 30A [Source:MGI Symbol;Acc:MGI:98178] | yes|up |
| ENSMUSG00000074945 | Olfr1314 | olfactory receptor 1314 [Source:MGI Symbol;Acc:MGI:3031148] | yes|up |
| ENSMUSG00000113491 | Gm19221 | predicted gene, 19221 [Source:MGI Symbol;Acc:MGI:5011406] | yes|down |
| ENSMUSG00000036766 | Dner | delta/notch-like EGF repeat containing [Source:MGI Symbol;Acc:MGI:2152889] | yes|up |
| ENSMUSG00000044066 | Cep68 | centrosomal protein 68 [Source:MGI Symbol;Acc:MGI:2667663] | yes|down |
| ENSMUSG00000113495 | Gm19792 | predicted gene, 19792 [Source:MGI Symbol;Acc:MGI:5011977] | yes|down |
| ENSMUSG00000030660 | Pik3c2a | phosphatidylinositol-4-phosphate 3-kinase catalytic subunit type 2 alpha [Source:MGI Symbol;Acc:MGI:1203729] | yes|up |
| ENSMUSG00000036768 | Kif15 | kinesin family member 15 [Source:MGI Symbol;Acc:MGI:1098258] | yes|down |
| ENSMUSG00000056121 | Fez2 | fasciculation and elongation protein zeta 2 (zygin II) [Source:MGI Symbol;Acc:MGI:2675856] | yes|up |
| ENSMUSG00000037787 | Coa8 | cytochrome c oxidase assembly factor 8 [Source:MGI Symbol;Acc:MGI:1915270] | yes|down |
| ENSMUSG00000001655 | Hoxc13 | homeobox C13 [Source:MGI Symbol;Acc:MGI:99560] | yes|down |
| ENSMUSG00000001656 | Hoxc11 | homeobox C11 [Source:MGI Symbol;Acc:MGI:96193] | yes|down |
| ENSMUSG00000001657 | Hoxc8 | homeobox C8 [Source:MGI Symbol;Acc:MGI:96198] | yes|down |
| ENSMUSG00000036368 | Rmdn2 | regulator of microtubule dynamics 2 [Source:MGI Symbol;Acc:MGI:2147043] | yes|up |
| ENSMUSG00000113019 | Gm47467 | predicted gene, 47467 [Source:MGI Symbol;Acc:MGI:6096433] | yes|down |
| ENSMUSG00000055980 | Irs1 | insulin receptor substrate 1 [Source:MGI Symbol;Acc:MGI:99454] | yes|up |
| ENSMUSG00000000782 | Tcf7 | transcription factor 7, T cell specific [Source:MGI Symbol;Acc:MGI:98507] | yes|down |
| ENSMUSG00000024220 | Zfp523 | zinc finger protein 523 [Source:MGI Symbol;Acc:MGI:2687278] | yes|down |
| ENSMUSG00000024222 | Fkbp5 | FK506 binding protein 5 [Source:MGI Symbol;Acc:MGI:104670] | yes|down |
| ENSMUSG00000005370 | Msh6 | mutS homolog 6 [Source:MGI Symbol;Acc:MGI:1343961] | yes|down |
| ENSMUSG00000024990 | Rbp4 | retinol binding protein 4, plasma [Source:MGI Symbol;Acc:MGI:97879] | yes|down |
| ENSMUSG00000027533 | Fabp5 | fatty acid binding protein 5, epidermal [Source:MGI Symbol;Acc:MGI:101790] | yes|down |
| ENSMUSG00000020212 | Mdm1 | transformed mouse 3T3 cell double minute 1 [Source:MGI Symbol;Acc:MGI:96951] | yes|down |
| ENSMUSG00000041126 | H2az2 | H2A.Z histone variant 2 [Source:MGI Symbol;Acc:MGI:1924855] | yes|down |
| ENSMUSG00000027242 | Wdr76 | WD repeat domain 76 [Source:MGI Symbol;Acc:MGI:1926186] | yes|down |
| ENSMUSG00000020961 | Ston2 | stonin 2 [Source:MGI Symbol;Acc:MGI:1918272] | yes|up |
| ENSMUSG00000041548 | Hspb8 | heat shock protein 8 [Source:MGI Symbol;Acc:MGI:2135756] | yes|up |
| ENSMUSG00000097519 | 4930558J18Rik | RIKEN cDNA 4930558J18 gene [Source:MGI Symbol;Acc:MGI:1922573] | yes|down |
| ENSMUSG00000050555 | Hyls1 | HYLS1, centriolar and ciliogenesis associated [Source:MGI Symbol;Acc:MGI:1924082] | yes|down |
| ENSMUSG00000030796 | Tead2 | TEA domain family member 2 [Source:MGI Symbol;Acc:MGI:104904] | yes|down |
| ENSMUSG00000030790 | Adm | adrenomedullin [Source:MGI Symbol;Acc:MGI:108058] | yes|up |
| ENSMUSG00000030793 | Pycard | PYD and CARD domain containing [Source:MGI Symbol;Acc:MGI:1931465] | yes|up |
| ENSMUSG00000054850 | Smim10l2a | small integral membrane protein 10 like 2A [Source:MGI Symbol;Acc:MGI:2443645] | yes|down |
| ENSMUSG00000030409 | Dmpk | dystrophia myotonica-protein kinase [Source:MGI Symbol;Acc:MGI:94906] | yes|up |
| ENSMUSG00000059363 | Fxn | frataxin [Source:MGI Symbol;Acc:MGI:1096879] | yes|down |
| ENSMUSG00000025887 | Casp12 | caspase 12 [Source:MGI Symbol;Acc:MGI:1312922] | yes|down |
| ENSMUSG00000030407 | Qpctl | glutaminyl-peptide cyclotransferase-like [Source:MGI Symbol;Acc:MGI:1914619] | yes|up |
| ENSMUSG00000037366 | Pafah2 | platelet-activating factor acetylhydrolase 2 [Source:MGI Symbol;Acc:MGI:2140321] | yes|up |
| ENSMUSG00000039652 | Cpeb3 | cytoplasmic polyadenylation element binding protein 3 [Source:MGI Symbol;Acc:MGI:2443075] | yes|up |
| ENSMUSG00000033685 | Ucp2 | uncoupling protein 2 (mitochondrial, proton carrier) [Source:MGI Symbol;Acc:MGI:109354] | yes|down |
| ENSMUSG00000037568 | Vash2 | vasohibin 2 [Source:MGI Symbol;Acc:MGI:2444826] | yes|down |
| ENSMUSG00000036564 | Ndrg4 | N-myc downstream regulated gene 4 [Source:MGI Symbol;Acc:MGI:2384590] | yes|up |
| ENSMUSG00000036565 | Ttyh3 | tweety family member 3 [Source:MGI Symbol;Acc:MGI:1925589] | yes|down |
| ENSMUSG00000095180 | Rhox5 | reproductive homeobox 5 [Source:MGI Symbol;Acc:MGI:97538] | yes|down |
| ENSMUSG00000113128 | Gm47813 | predicted gene, 47813 [Source:MGI Symbol;Acc:MGI:6096997] | yes|down |
| ENSMUSG00000030064 | Frmd4b | FERM domain containing 4B [Source:MGI Symbol;Acc:MGI:2141794] | yes|up |
| ENSMUSG00000097194 | 9330175E14Rik | RIKEN cDNA 9330175E14 gene [Source:MGI Symbol;Acc:MGI:2443913] | yes|down |
| ENSMUSG00000097195 | Snhg5 | small nucleolar RNA host gene 5 [Source:MGI Symbol;Acc:MGI:1919905] | yes|down |
| ENSMUSG00000030060 | Hmces | 5-hydroxymethylcytosine (hmC) binding, ES cell specific [Source:MGI Symbol;Acc:MGI:1914053] | yes|down |
| ENSMUSG00000027333 | Smox | spermine oxidase [Source:MGI Symbol;Acc:MGI:2445356] | yes|down |
| ENSMUSG00000040187 | Arntl2 | aryl hydrocarbon receptor nuclear translocator-like 2 [Source:MGI Symbol;Acc:MGI:2684845] | yes|up |
| ENSMUSG00000027331 | Knstrn | kinetochore-localized astrin/SPAG5 binding [Source:MGI Symbol;Acc:MGI:1289298] | yes|down |
| ENSMUSG00000027330 | Cdc25b | cell division cycle 25B [Source:MGI Symbol;Acc:MGI:99701] | yes|down |
| ENSMUSG00000040631 | Dok4 | docking protein 4 [Source:MGI Symbol;Acc:MGI:2148865] | yes|up |
| ENSMUSG00000048355 | Arxes1 | adipocyte-related X-chromosome expressed sequence 1 [Source:MGI Symbol;Acc:MGI:1923469] | yes|down |
| ENSMUSG00000121318 |  | novel transcript | yes|down |
| ENSMUSG00000121319 |  | novel transcript | yes|down |
| ENSMUSG00000026202 | Tuba4a | tubulin, alpha 4A [Source:MGI Symbol;Acc:MGI:1095410] | yes|up |
| ENSMUSG00000030882 | Dnhd1 | dynein heavy chain domain 1 [Source:MGI Symbol;Acc:MGI:1924755] | yes|up |
| ENSMUSG00000026204 | Ptprn | protein tyrosine phosphatase, receptor type, N [Source:MGI Symbol;Acc:MGI:102765] | yes|down |
| ENSMUSG00000050359 | Sprr1a | small proline-rich protein 1A [Source:MGI Symbol;Acc:MGI:106660] | yes|up |
| ENSMUSG00000002006 | Pdzd4 | PDZ domain containing 4 [Source:MGI Symbol;Acc:MGI:2443483] | yes|down |
| ENSMUSG00000030086 | Chchd6 | coiled-coil-helix-coiled-coil-helix domain containing 6 [Source:MGI Symbol;Acc:MGI:1913348] | yes|down |
| ENSMUSG00000055435 | Maf | avian musculoaponeurotic fibrosarcoma oncogene homolog [Source:MGI Symbol;Acc:MGI:96909] | yes|up |
| ENSMUSG00000030088 | Aldh1l1 | aldehyde dehydrogenase 1 family, member L1 [Source:MGI Symbol;Acc:MGI:1340024] | yes|up |
| ENSMUSG00000097178 | 2310002F09Rik | RIKEN cDNA 2310002F09 gene [Source:MGI Symbol;Acc:MGI:1916779] | yes|up |
| ENSMUSG00000055430 | Nap1l5 | nucleosome assembly protein 1-like 5 [Source:MGI Symbol;Acc:MGI:1923555] | yes|up |
| ENSMUSG00000037211 | Spry1 | sprouty RTK signaling antagonist 1 [Source:MGI Symbol;Acc:MGI:1345139] | yes|up |
| ENSMUSG00000030246 | Ldhb | lactate dehydrogenase B [Source:MGI Symbol;Acc:MGI:96763] | yes|down |
| ENSMUSG00000097772 | 5430416N02Rik | RIKEN cDNA 5430416N02 gene [Source:MGI Symbol;Acc:MGI:1918676] | yes|down |
| ENSMUSG00000056749 | Nfil3 | nuclear factor, interleukin 3, regulated [Source:MGI Symbol;Acc:MGI:109495] | yes|up |
| ENSMUSG00000030536 | Iqgap1 | IQ motif containing GTPase activating protein 1 [Source:MGI Symbol;Acc:MGI:1352757] | yes|up |
| ENSMUSG00000030538 | Cib1 | calcium and integrin binding 1 (calmyrin) [Source:MGI Symbol;Acc:MGI:1344418] | yes|up |
| ENSMUSG00000059187 | Tafa1 | TAFA chemokine like family member 1 [Source:MGI Symbol;Acc:MGI:2443695] | yes|down |
| ENSMUSG00000031428 | Zcchc18 | zinc finger, CCHC domain containing 18 [Source:MGI Symbol;Acc:MGI:1914245] | yes|down |
| ENSMUSG00000027750 | Postn | periostin, osteoblast specific factor [Source:MGI Symbol;Acc:MGI:1926321] | yes|up |
| ENSMUSG00000070713 | Hmgn2-ps | high mobility group nucleosomal binding domain 2, pseudogene [Source:MGI Symbol;Acc:MGI:3704312] | yes|down |
| ENSMUSG00000027752 | Exosc8 | exosome component 8 [Source:MGI Symbol;Acc:MGI:1916889] | yes|down |
| ENSMUSG00000031373 | Car5b | carbonic anhydrase 5b, mitochondrial [Source:MGI Symbol;Acc:MGI:1926249] | yes|up |
| ENSMUSG00000059027 | 9630013D21Rik | RIKEN cDNA 9630013D21 gene [Source:MGI Symbol;Acc:MGI:2442649] | yes|down |
| ENSMUSG00000040521 | Tsfm | Ts translation elongation factor, mitochondrial [Source:MGI Symbol;Acc:MGI:1913649] | yes|down |
| ENSMUSG00000031930 | Wwp2 | WW domain containing E3 ubiquitin protein ligase 2 [Source:MGI Symbol;Acc:MGI:1914144] | yes|down |
| ENSMUSG00000026110 | Mgat4a | mannoside acetylglucosaminyltransferase 4, isoenzyme A [Source:MGI Symbol;Acc:MGI:2662992] | yes|down |
| ENSMUSG00000031425 | Plp1 | proteolipid protein (myelin) 1 [Source:MGI Symbol;Acc:MGI:97623] | yes|down |
| ENSMUSG00000033032 | Afap1l1 | actin filament associated protein 1-like 1 [Source:MGI Symbol;Acc:MGI:2147199] | yes|down |
| ENSMUSG00000060923 | Acyp2 | acylphosphatase 2, muscle type [Source:MGI Symbol;Acc:MGI:1922822] | yes|up |
| ENSMUSG00000051537 | Gm5124 | predicted pseudogene 5124 [Source:MGI Symbol;Acc:MGI:3643622] | yes|down |
| ENSMUSG00000066363 | Serpina3f | serine (or cysteine) peptidase inhibitor, clade A, member 3F [Source:MGI Symbol;Acc:MGI:2182838] | yes|down |
| ENSMUSG00000019647 | Sema6a | sema domain, transmembrane domain (TM), and cytoplasmic domain, (semaphorin) 6A [Source:MGI Symbol;Acc:MGI:1203727] | yes|up |
| ENSMUSG00000039787 | Cercam | cerebral endothelial cell adhesion molecule [Source:MGI Symbol;Acc:MGI:2139134] | yes|up |
| ENSMUSG00000003534 | Ddr1 | discoidin domain receptor family, member 1 [Source:MGI Symbol;Acc:MGI:99216] | yes|up |
| ENSMUSG00000039781 | Cep131 | centrosomal protein 131 [Source:MGI Symbol;Acc:MGI:107440] | yes|down |
| ENSMUSG00000003531 | Dgcr6 | DiGeorge syndrome critical region gene 6 [Source:MGI Symbol;Acc:MGI:1202877] | yes|down |
| ENSMUSG00000039782 | Cpeb2 | cytoplasmic polyadenylation element binding protein 2 [Source:MGI Symbol;Acc:MGI:2442640] | yes|up |
| ENSMUSG00000031503 | Col4a2 | collagen, type IV, alpha 2 [Source:MGI Symbol;Acc:MGI:88455] | yes|up |
| ENSMUSG00000055723 | Rras2 | related RAS viral (r-ras) oncogene 2 [Source:MGI Symbol;Acc:MGI:1914172] | yes|up |
| ENSMUSG00000025875 | Tspan17 | tetraspanin 17 [Source:MGI Symbol;Acc:MGI:1921507] | yes|up |
| ENSMUSG00000085088 | 4931413K12Rik | RIKEN cDNA 4931413K12 gene [Source:MGI Symbol;Acc:MGI:1918237] | yes|up |
| ENSMUSG00000062077 | Trim54 | tripartite motif-containing 54 [Source:MGI Symbol;Acc:MGI:1889623] | yes|down |
| ENSMUSG00000062075 | Lmnb2 | lamin B2 [Source:MGI Symbol;Acc:MGI:96796] | yes|down |
| ENSMUSG00000072620 | Slfn2 | schlafen 2 [Source:MGI Symbol;Acc:MGI:1313258] | yes|down |
| ENSMUSG00000030337 | Vamp1 | vesicle-associated membrane protein 1 [Source:MGI Symbol;Acc:MGI:1313276] | yes|up |
| ENSMUSG00000072844 | G530011O06Rik | RIKEN cDNA G530011O06 gene [Source:MGI Symbol;Acc:MGI:3603513] | yes|down |
| ENSMUSG00000039438 | Ttc36 | tetratricopeptide repeat domain 36 [Source:MGI Symbol;Acc:MGI:2384760] | yes|up |
| ENSMUSG00000021367 | Edn1 | endothelin 1 [Source:MGI Symbol;Acc:MGI:95283] | yes|up |
| ENSMUSG00000031750 | Il34 | interleukin 34 [Source:MGI Symbol;Acc:MGI:1923777] | yes|up |
| ENSMUSG00000021365 | Nedd9 | neural precursor cell expressed, developmentally down-regulated gene 9 [Source:MGI Symbol;Acc:MGI:97302] | yes|up |
| ENSMUSG00000031756 | Cenpn | centromere protein N [Source:MGI Symbol;Acc:MGI:1919405] | yes|down |
| ENSMUSG00000020953 | Coch | cochlin [Source:MGI Symbol;Acc:MGI:1278313] | yes|up |
| ENSMUSG00000031209 | Heph | hephaestin [Source:MGI Symbol;Acc:MGI:1332240] | yes|up |
| ENSMUSG00000028341 | Nr4a3 | nuclear receptor subfamily 4, group A, member 3 [Source:MGI Symbol;Acc:MGI:1352457] | yes|up |
| ENSMUSG00000098557 | Kctd12 | potassium channel tetramerisation domain containing 12 [Source:MGI Symbol;Acc:MGI:2145823] | yes|up |
| ENSMUSG00000071532 | Gm10335 | predicted gene 10335 [Source:MGI Symbol;Acc:MGI:3641693] | yes|down |
| ENSMUSG00000029810 | Tmem176b | transmembrane protein 176B [Source:MGI Symbol;Acc:MGI:1916348] | yes|down |
| ENSMUSG00000026576 | Atp1b1 | ATPase, Na+/K+ transporting, beta 1 polypeptide [Source:MGI Symbol;Acc:MGI:88108] | yes|up |
| ENSMUSG00000023505 | Cdca3 | cell division cycle associated 3 [Source:MGI Symbol;Acc:MGI:1315198] | yes|down |
| ENSMUSG00000029009 | Mthfr | methylenetetrahydrofolate reductase [Source:MGI Symbol;Acc:MGI:106639] | yes|up |
| ENSMUSG00000040747 | Cd53 | CD53 antigen [Source:MGI Symbol;Acc:MGI:88341] | yes|down |
| ENSMUSG00000026579 | F5 | coagulation factor V [Source:MGI Symbol;Acc:MGI:88382] | yes|up |
| ENSMUSG00000022054 | Nefm | neurofilament, medium polypeptide [Source:MGI Symbol;Acc:MGI:97314] | yes|up |
| ENSMUSG00000029001 | Fbxo44 | F-box protein 44 [Source:MGI Symbol;Acc:MGI:1354744] | yes|up |
| ENSMUSG00000039735 | Fnbp1l | formin binding protein 1-like [Source:MGI Symbol;Acc:MGI:1925642] | yes|down |
| ENSMUSG00000042909 | Olfr648 | olfactory receptor 648 [Source:MGI Symbol;Acc:MGI:3030482] | yes|down |
| ENSMUSG00000003228 | Grk5 | G protein-coupled receptor kinase 5 [Source:MGI Symbol;Acc:MGI:109161] | yes|up |
| ENSMUSG00000035305 | Ror1 | receptor tyrosine kinase-like orphan receptor 1 [Source:MGI Symbol;Acc:MGI:1347520] | yes|up |
| ENSMUSG00000030643 | Rab30 | RAB30, member RAS oncogene family [Source:MGI Symbol;Acc:MGI:1923235] | yes|up |
| ENSMUSG00000036813 | Entpd8 | ectonucleoside triphosphate diphosphohydrolase 8 [Source:MGI Symbol;Acc:MGI:1919340] | yes|up |
| ENSMUSG00000026979 | Psd4 | pleckstrin and Sec7 domain containing 4 [Source:MGI Symbol;Acc:MGI:2674093] | yes|up |
| ENSMUSG00000022673 | Mcm4 | minichromosome maintenance complex component 4 [Source:MGI Symbol;Acc:MGI:103199] | yes|down |
| ENSMUSG00000047181 | Samd14 | sterile alpha motif domain containing 14 [Source:MGI Symbol;Acc:MGI:2384945] | yes|down |
| ENSMUSG00000085882 | 2610507I01Rik | RIKEN cDNA 2610507I01 gene [Source:MGI Symbol;Acc:MGI:1919453] | yes|down |
| ENSMUSG00000025586 | Cpeb1 | cytoplasmic polyadenylation element binding protein 1 [Source:MGI Symbol;Acc:MGI:108442] | yes|up |
| ENSMUSG00000025584 | Pde8a | phosphodiesterase 8A [Source:MGI Symbol;Acc:MGI:1277116] | yes|up |
| ENSMUSG00000025582 | Nptx1 | neuronal pentraxin 1 [Source:MGI Symbol;Acc:MGI:107811] | yes|down |
| ENSMUSG00000043122 | A530016L24Rik | RIKEN cDNA A530016L24 gene [Source:MGI Symbol;Acc:MGI:2443020] | yes|up |
| ENSMUSG00000025479 | Cyp2e1 | cytochrome P450, family 2, subfamily e, polypeptide 1 [Source:MGI Symbol;Acc:MGI:88607] | yes|up |
| ENSMUSG00000029177 | Cenpa | centromere protein A [Source:MGI Symbol;Acc:MGI:88375] | yes|down |
| ENSMUSG00000048402 | Gli2 | GLI-Kruppel family member GLI2 [Source:MGI Symbol;Acc:MGI:95728] | yes|down |
| ENSMUSG00000029910 | Mad2l1 | MAD2 mitotic arrest deficient-like 1 [Source:MGI Symbol;Acc:MGI:1860374] | yes|down |
| ENSMUSG00000012483 | Rpa3 | replication protein A3 [Source:MGI Symbol;Acc:MGI:1915490] | yes|down |
| ENSMUSG00000025475 | Adgra1 | adhesion G protein-coupled receptor A1 [Source:MGI Symbol;Acc:MGI:1277167] | yes|down |
| ENSMUSG00000025278 | Flnb | filamin, beta [Source:MGI Symbol;Acc:MGI:2446089] | yes|up |
| ENSMUSG00000022582 | Ly6g | lymphocyte antigen 6 complex, locus G [Source:MGI Symbol;Acc:MGI:109440] | yes|down |
| ENSMUSG00000115920 | Gm18283 | predicted gene, 18283 [Source:MGI Symbol;Acc:MGI:5010468] | yes|down |
| ENSMUSG00000021678 | F2rl1 | coagulation factor II (thrombin) receptor-like 1 [Source:MGI Symbol;Acc:MGI:101910] | yes|up |
| ENSMUSG00000039323 | Igfbp2 | insulin-like growth factor binding protein 2 [Source:MGI Symbol;Acc:MGI:96437] | yes|up |
| ENSMUSG00000042846 | Lrrtm3 | leucine rich repeat transmembrane neuronal 3 [Source:MGI Symbol;Acc:MGI:2389177] | yes|up |
| ENSMUSG00000042842 | Serpinb6b | serine (or cysteine) peptidase inhibitor, clade B, member 6b [Source:MGI Symbol;Acc:MGI:894688] | yes|up |
| ENSMUSG00000109005 | Gm45221 | predicted gene 45221 [Source:MGI Symbol;Acc:MGI:5753797] | yes|up |
| ENSMUSG00000071424 | Grid2 | glutamate receptor, ionotropic, delta 2 [Source:MGI Symbol;Acc:MGI:95813] | yes|up |
| ENSMUSG00000098374 | Gm28043 | predicted gene, 28043 [Source:MGI Symbol;Acc:MGI:5547779] | yes|down |
| ENSMUSG00000046295 | Ankle1 | ankyrin repeat and LEM domain containing 1 [Source:MGI Symbol;Acc:MGI:1918775] | yes|down |
| ENSMUSG00000061518 | Cox5b | cytochrome c oxidase subunit 5B [Source:MGI Symbol;Acc:MGI:88475] | yes|down |
| ENSMUSG00000029755 | Dlx5 | distal-less homeobox 5 [Source:MGI Symbol;Acc:MGI:101926] | yes|up |
| ENSMUSG00000025790 | Slco3a1 | solute carrier organic anion transporter family, member 3a1 [Source:MGI Symbol;Acc:MGI:1351867] | yes|up |
| ENSMUSG00000020691 | Mettl2 | methyltransferase like 2 [Source:MGI Symbol;Acc:MGI:1289171] | yes|down |
| ENSMUSG00000027199 | Gatm | glycine amidinotransferase (L-arginine:glycine amidinotransferase) [Source:MGI Symbol;Acc:MGI:1914342] | yes|up |
| ENSMUSG00000030688 | Stard10 | START domain containing 10 [Source:MGI Symbol;Acc:MGI:1860093] | yes|up |
| ENSMUSG00000029591 | Ung | uracil DNA glycosylase [Source:MGI Symbol;Acc:MGI:109352] | yes|down |
| ENSMUSG00000002835 | Chaf1a | chromatin assembly factor 1, subunit A (p150) [Source:MGI Symbol;Acc:MGI:1351331] | yes|down |
| ENSMUSG00000029596 | Sdsl | serine dehydratase-like [Source:MGI Symbol;Acc:MGI:2182607] | yes|down |
| ENSMUSG00000105703 | Gm43305 | predicted gene 43305 [Source:MGI Symbol;Acc:MGI:5663442] | yes|down |
| ENSMUSG00000067575 | Rpl35a-ps3 | ribosomal protein L35A, pseudogene 3 [Source:MGI Symbol;Acc:MGI:3704473] | yes|down |
| ENSMUSG00000056234 | Ncoa4 | nuclear receptor coactivator 4 [Source:MGI Symbol;Acc:MGI:1350932] | yes|up |
| ENSMUSG00000048503 | Tlcd5 | TLC domain containing 5 [Source:MGI Symbol;Acc:MGI:2685030] | yes|up |
| ENSMUSG00000016256 | Ctsz | cathepsin Z [Source:MGI Symbol;Acc:MGI:1891190] | yes|up |
| ENSMUSG00000073599 | Ecscr | endothelial cell surface expressed chemotaxis and apoptosis regulator [Source:MGI Symbol;Acc:MGI:1915795] | yes|up |
| ENSMUSG00000046561 | Arsj | arylsulfatase J [Source:MGI Symbol;Acc:MGI:2443513] | yes|down |
| ENSMUSG00000038319 | Kcnh2 | potassium voltage-gated channel, subfamily H (eag-related), member 2 [Source:MGI Symbol;Acc:MGI:1341722] | yes|down |
| ENSMUSG00000062980 | Cped1 | cadherin-like and PC-esterase domain containing 1 [Source:MGI Symbol;Acc:MGI:2444814] | yes|up |
| ENSMUSG00000042604 | Kcna4 | potassium voltage-gated channel, shaker-related subfamily, member 4 [Source:MGI Symbol;Acc:MGI:96661] | yes|down |
| ENSMUSG00000027985 | Lef1 | lymphoid enhancer binding factor 1 [Source:MGI Symbol;Acc:MGI:96770] | yes|down |
| ENSMUSG00000034327 | Kctd9 | potassium channel tetramerisation domain containing 9 [Source:MGI Symbol;Acc:MGI:2145579] | yes|up |
| ENSMUSG00000034329 | Brip1 | BRCA1 interacting protein C-terminal helicase 1 [Source:MGI Symbol;Acc:MGI:2442836] | yes|down |
| ENSMUSG00000087659 | Gm12606 | predicted gene 12606 [Source:MGI Symbol;Acc:MGI:3649222] | yes|up |
| ENSMUSG00000042608 | Stk40 | serine/threonine kinase 40 [Source:MGI Symbol;Acc:MGI:1921428] | yes|up |
| ENSMUSG00000035540 | Gc | vitamin D binding protein [Source:MGI Symbol;Acc:MGI:95669] | yes|up |
| ENSMUSG00000020181 | Nav3 | neuron navigator 3 [Source:MGI Symbol;Acc:MGI:2183703] | yes|down |
| ENSMUSG00000006014 | Prg4 | proteoglycan 4 (megakaryocyte stimulating factor, articular superficial zone protein) [Source:MGI Symbol;Acc:MGI:1891344] | yes|up |
| ENSMUSG00000020185 | E2f7 | E2F transcription factor 7 [Source:MGI Symbol;Acc:MGI:1289147] | yes|down |
| ENSMUSG00000117545 | Gm30794 | predicted gene, 30794 [Source:MGI Symbol;Acc:MGI:5589953] | yes|up |
| ENSMUSG00000056917 | Sipa1 | signal-induced proliferation associated gene 1 [Source:MGI Symbol;Acc:MGI:107576] | yes|down |
| ENSMUSG00000064023 | Klk8 | kallikrein related-peptidase 8 [Source:MGI Symbol;Acc:MGI:1343327] | yes|up |
| ENSMUSG00000028402 | Mpdz | multiple PDZ domain crumbs cell polarity complex component [Source:MGI Symbol;Acc:MGI:1343489] | yes|up |
| ENSMUSG00000042029 | Ncapg2 | non-SMC condensin II complex, subunit G2 [Source:MGI Symbol;Acc:MGI:1923294] | yes|down |
| ENSMUSG00000076846 | Trav13-2 | T cell receptor alpha variable 13-2 [Source:MGI Symbol;Acc:MGI:3651611] | yes|up |
| ENSMUSG00000018340 | Anxa6 | annexin A6 [Source:MGI Symbol;Acc:MGI:88255] | yes|up |
| ENSMUSG00000028530 | Jak1 | Janus kinase 1 [Source:MGI Symbol;Acc:MGI:96628] | yes|up |
| ENSMUSG00000003032 | Klf4 | Kruppel-like factor 4 (gut) [Source:MGI Symbol;Acc:MGI:1342287] | yes|up |
| ENSMUSG00000103898 | Gm30238 | predicted gene, 30238 [Source:MGI Symbol;Acc:MGI:5589397] | yes|down |
| ENSMUSG00000035125 | Gcfc2 | GC-rich sequence DNA binding factor 2 [Source:MGI Symbol;Acc:MGI:2141656] | yes|down |
| ENSMUSG00000032387 | Rbpms2 | RNA binding protein with multiple splicing 2 [Source:MGI Symbol;Acc:MGI:1919223] | yes|down |
| ENSMUSG00000103897 | Pcdhga8 | protocadherin gamma subfamily A, 8 [Source:MGI Symbol;Acc:MGI:1935221] | yes|up |
| ENSMUSG00000027358 | Bmp2 | bone morphogenetic protein 2 [Source:MGI Symbol;Acc:MGI:88177] | yes|down |
| ENSMUSG00000120789 |  | novel transcript | yes|up |
| ENSMUSG00000021614 | Vcan | versican [Source:MGI Symbol;Acc:MGI:102889] | yes|up |
| ENSMUSG00000032300 | 1700017B05Rik | RIKEN cDNA 1700017B05 gene [Source:MGI Symbol;Acc:MGI:1921461] | yes|down |
| ENSMUSG00000050914 | Ankrd37 | ankyrin repeat domain 37 [Source:MGI Symbol;Acc:MGI:3603344] | yes|up |
| ENSMUSG00000020747 | Tmem94 | transmembrane protein 94 [Source:MGI Symbol;Acc:MGI:1919197] | yes|down |
| ENSMUSG00000113569 | Gm48035 | predicted gene, 48035 [Source:MGI Symbol;Acc:MGI:6097350] | yes|down |
| ENSMUSG00000032555 | Topbp1 | topoisomerase (DNA) II binding protein 1 [Source:MGI Symbol;Acc:MGI:1920018] | yes|down |
| ENSMUSG00000006301 | Tmbim1 | transmembrane BAX inhibitor motif containing 1 [Source:MGI Symbol;Acc:MGI:1916910] | yes|up |
| ENSMUSG00000042429 | Adora1 | adenosine A1 receptor [Source:MGI Symbol;Acc:MGI:99401] | yes|down |
| ENSMUSG00000033871 | Ppargc1b | peroxisome proliferative activated receptor, gamma, coactivator 1 beta [Source:MGI Symbol;Acc:MGI:2444934] | yes|down |
| ENSMUSG00000028249 | Sdcbp | syndecan binding protein [Source:MGI Symbol;Acc:MGI:1337026] | yes|up |
| ENSMUSG00000024056 | Ndc80 | NDC80 kinetochore complex component [Source:MGI Symbol;Acc:MGI:1914302] | yes|down |
| ENSMUSG00000063450 | Syne2 | spectrin repeat containing, nuclear envelope 2 [Source:MGI Symbol;Acc:MGI:2449316] | yes|down |
| ENSMUSG00000024053 | Emilin2 | elastin microfibril interfacer 2 [Source:MGI Symbol;Acc:MGI:2389136] | yes|up |
| ENSMUSG00000097156 | Gm3764 | predicted gene 3764 [Source:MGI Symbol;Acc:MGI:3781938] | yes|down |
| ENSMUSG00000063455 | D630045J12Rik | RIKEN cDNA D630045J12 gene [Source:MGI Symbol;Acc:MGI:2669829] | yes|down |
| ENSMUSG00000063698 | Sfxn4 | sideroflexin 4 [Source:MGI Symbol;Acc:MGI:2137680] | yes|down |
| ENSMUSG00000045103 | Dmd | dystrophin, muscular dystrophy [Source:MGI Symbol;Acc:MGI:94909] | yes|up |
| ENSMUSG00000045104 | Ldhb-ps | lactate dehydrogenase B, pseudogene [Source:MGI Symbol;Acc:MGI:3645435] | yes|down |
| ENSMUSG00000103144 | Pcdhga1 | protocadherin gamma subfamily A, 1 [Source:MGI Symbol;Acc:MGI:1935212] | yes|up |
| ENSMUSG00000045107 | Saysd1 | SAYSVFN motif domain containing 1 [Source:MGI Symbol;Acc:MGI:1914759] | yes|up |
| ENSMUSG00000068794 | Col28a1 | collagen, type XXVIII, alpha 1 [Source:MGI Symbol;Acc:MGI:2685312] | yes|up |
| ENSMUSG00000036875 | Dna2 | DNA replication helicase/nuclease 2 [Source:MGI Symbol;Acc:MGI:2443732] | yes|down |
| ENSMUSG00000063696 | Gm8730 | predicted pseudogene 8730 [Source:MGI Symbol;Acc:MGI:3644565] | yes|up |
| ENSMUSG00000044456 | Rin3 | Ras and Rab interactor 3 [Source:MGI Symbol;Acc:MGI:2385708] | yes|up |
| ENSMUSG00000075593 | Gal3st4 | galactose-3-O-sulfotransferase 4 [Source:MGI Symbol;Acc:MGI:1916254] | yes|down |
| ENSMUSG00000045589 | Frrs1l | ferric-chelate reductase 1 like [Source:MGI Symbol;Acc:MGI:2442704] | yes|up |
| ENSMUSG00000021819 | Zswim8 | zinc finger SWIM-type containing 8 [Source:MGI Symbol;Acc:MGI:1919156] | yes|up |
| ENSMUSG00000120308 |  | novel transcript, antisense to Stub1 | yes|down |
| ENSMUSG00000045854 | Lyrm2 | LYR motif containing 2 [Source:MGI Symbol;Acc:MGI:1917573] | yes|up |
| ENSMUSG00000052446 | Zfp961 | zinc finger protein 961 [Source:MGI Symbol;Acc:MGI:3583954] | yes|down |
| ENSMUSG00000032010 | Usp2 | ubiquitin specific peptidase 2 [Source:MGI Symbol;Acc:MGI:1858178] | yes|up |
| ENSMUSG00000032011 | Thy1 | thymus cell antigen 1, theta [Source:MGI Symbol;Acc:MGI:98747] | yes|up |
| ENSMUSG00000017723 | Wfdc2 | WAP four-disulfide core domain 2 [Source:MGI Symbol;Acc:MGI:1914951] | yes|up |
| ENSMUSG00000078674 | Mup18 | major urinary protein 18 [Source:MGI Symbol;Acc:MGI:3705220] | yes|up |
| ENSMUSG00000078672 | Mup20 | major urinary protein 20 [Source:MGI Symbol;Acc:MGI:3651981] | yes|up |
| ENSMUSG00000078673 | Mup19 | major urinary protein 19 [Source:MGI Symbol;Acc:MGI:3705235] | yes|up |
| ENSMUSG00000078670 | Fam174b | family with sequence similarity 174, member B [Source:MGI Symbol;Acc:MGI:3698178] | yes|up |
| ENSMUSG00000017724 | Etv4 | ets variant 4 [Source:MGI Symbol;Acc:MGI:99423] | yes|down |
| ENSMUSG00000021806 | Nid2 | nidogen 2 [Source:MGI Symbol;Acc:MGI:1298229] | yes|up |
| ENSMUSG00000112980 | D430020J02Rik | RIKEN cDNA D430020J02 gene [Source:MGI Symbol;Acc:MGI:2442237] | yes|down |
| ENSMUSG00000021803 | Cdhr1 | cadherin-related family member 1 [Source:MGI Symbol;Acc:MGI:2157782] | yes|up |
| ENSMUSG00000054679 | Srsf12 | serine and arginine-rich splicing factor 12 [Source:MGI Symbol;Acc:MGI:2661424] | yes|up |
| ENSMUSG00000045215 | Asxl3 | ASXL transcriptional regulator 3 [Source:MGI Symbol;Acc:MGI:2685175] | yes|up |
| ENSMUSG00000047044 | D030056L22Rik | RIKEN cDNA D030056L22 gene [Source:MGI Symbol;Acc:MGI:3583960] | yes|down |
| ENSMUSG00000049036 | Tmem121 | transmembrane protein 121 [Source:MGI Symbol;Acc:MGI:1916445] | yes|down |
| ENSMUSG00000090622 | A930033H14Rik | RIKEN cDNA A930033H14 gene [Source:MGI Symbol;Acc:MGI:2444562] | yes|down |
| ENSMUSG00000020354 | Sgcd | sarcoglycan, delta (dystrophin-associated glycoprotein) [Source:MGI Symbol;Acc:MGI:1346525] | yes|up |
| ENSMUSG00000027435 | Cd93 | CD93 antigen [Source:MGI Symbol;Acc:MGI:106664] | yes|down |
| ENSMUSG00000053483 | Usp21 | ubiquitin specific peptidase 21 [Source:MGI Symbol;Acc:MGI:1353665] | yes|down |
| ENSMUSG00000117819 | Gm50253 | predicted gene, 50253 [Source:MGI Symbol;Acc:MGI:6303071] | yes|down |
| ENSMUSG00000021763 | Cspg4b | chondroitin sulfate proteoglycan 4B [Source:MGI Symbol;Acc:MGI:3040697] | yes|up |
| ENSMUSG00000078816 | Prkcg | protein kinase C, gamma [Source:MGI Symbol;Acc:MGI:97597] | yes|up |
| ENSMUSG00000038738 | Shank1 | SH3 and multiple ankyrin repeat domains 1 [Source:MGI Symbol;Acc:MGI:3613677] | yes|down |
| ENSMUSG00000113393 | Gm48415 | predicted gene, 48415 [Source:MGI Symbol;Acc:MGI:6097908] | yes|up |
| ENSMUSG00000117815 | Gm50166 | predicted gene, 50166 [Source:MGI Symbol;Acc:MGI:6302931] | yes|down |
| ENSMUSG00000019838 | Slc16a10 | solute carrier family 16 (monocarboxylic acid transporters), member 10 [Source:MGI Symbol;Acc:MGI:1919722] | yes|down |
| ENSMUSG00000079389 | Gm3149 | predicted gene 3149 [Source:MGI Symbol;Acc:MGI:3781328] | yes|up |
| ENSMUSG00000034591 | Slc41a2 | solute carrier family 41, member 2 [Source:MGI Symbol;Acc:MGI:2442940] | yes|up |
| ENSMUSG00000019831 | Wasf1 | WASP family, member 1 [Source:MGI Symbol;Acc:MGI:1890563] | yes|down |
| ENSMUSG00000053007 | Creb5 | cAMP responsive element binding protein 5 [Source:MGI Symbol;Acc:MGI:2443973] | yes|down |
| ENSMUSG00000032478 | Nme6 | NME/NM23 nucleoside diphosphate kinase 6 [Source:MGI Symbol;Acc:MGI:1861676] | yes|down |
| ENSMUSG00000113262 | Gm48551 | predicted gene, 48551 [Source:MGI Symbol;Acc:MGI:6098103] | yes|down |
| ENSMUSG00000078706 | Gm53 | predicted gene 53 [Source:MGI Symbol;Acc:MGI:2684899] | yes|down |
| ENSMUSG00000024180 | Pgap6 | post-glycosylphosphatidylinositol attachment to proteins 6 [Source:MGI Symbol;Acc:MGI:1926283] | yes|down |
| ENSMUSG00000028010 | Gar1 | GAR1 ribonucleoprotein [Source:MGI Symbol;Acc:MGI:1930948] | yes|down |
| ENSMUSG00000054065 | Pkp3 | plakophilin 3 [Source:MGI Symbol;Acc:MGI:1891830] | yes|down |
| ENSMUSG00000024451 | Arap3 | ArfGAP with RhoGAP domain, ankyrin repeat and PH domain 3 [Source:MGI Symbol;Acc:MGI:2147274] | yes|down |
| ENSMUSG00000041842 | Fhdc1 | FH2 domain containing 1 [Source:MGI Symbol;Acc:MGI:2684972] | yes|up |
| ENSMUSG00000079654 | Prrt4 | proline-rich transmembrane protein 4 [Source:MGI Symbol;Acc:MGI:2141677] | yes|up |
| ENSMUSG00000078490 | Cfap74 | cilia and flagella associated protein 74 [Source:MGI Symbol;Acc:MGI:1917130] | yes|down |
| ENSMUSG00000116632 | Magef1 | MAGE family member F1 [Source:MGI Symbol;Acc:MGI:1923472] | yes|down |
| ENSMUSG00000078496 | Zfp982 | zinc finger protein 982 [Source:MGI Symbol;Acc:MGI:3701121] | yes|up |
| ENSMUSG00000036790 | Slitrk2 | SLIT and NTRK-like family, member 2 [Source:MGI Symbol;Acc:MGI:2679449] | yes|up |
| ENSMUSG00000050295 | Foxc1 | forkhead box C1 [Source:MGI Symbol;Acc:MGI:1347466] | yes|down |
| ENSMUSG00000032878 | Ccdc85a | coiled-coil domain containing 85A [Source:MGI Symbol;Acc:MGI:2445069] | yes|up |
| ENSMUSG00000063077 | Kif1b | kinesin family member 1B [Source:MGI Symbol;Acc:MGI:108426] | yes|up |
| ENSMUSG00000117922 | Gm50397 | predicted gene, 50397 [Source:MGI Symbol;Acc:MGI:6303305] | yes|up |
| ENSMUSG00000096210 | H1f0 | H1.0 linker histone [Source:MGI Symbol;Acc:MGI:95893] | yes|down |
| ENSMUSG00000099241 | Gvin-ps2 | GTPase, very large interferon inducible, pseudogene 2 [Source:MGI Symbol;Acc:MGI:5011037] | yes|up |
| ENSMUSG00000030659 | Nucb2 | nucleobindin 2 [Source:MGI Symbol;Acc:MGI:1858179] | yes|up |
| ENSMUSG00000044071 | Tafa2 | TAFA chemokine like family member 2 [Source:MGI Symbol;Acc:MGI:2143691] | yes|up |
| ENSMUSG00000097616 | 1110019D14Rik | RIKEN cDNA 1110019D14 gene [Source:MGI Symbol;Acc:MGI:1923561] | yes|down |
| ENSMUSG00000097343 | 9030407P20Rik | RIKEN cDNA 9030407P20 gene [Source:MGI Symbol;Acc:MGI:1918818] | yes|up |
| ENSMUSG00000027954 | Efna1 | ephrin A1 [Source:MGI Symbol;Acc:MGI:103236] | yes|down |
| ENSMUSG00000058571 | Gpc6 | glypican 6 [Source:MGI Symbol;Acc:MGI:1346322] | yes|up |
| ENSMUSG00000037339 | Fam53a | family with sequence similarity 53, member A [Source:MGI Symbol;Acc:MGI:1919225] | yes|down |
| ENSMUSG00000020363 | Gfpt2 | glutamine fructose-6-phosphate transaminase 2 [Source:MGI Symbol;Acc:MGI:1338883] | yes|up |
[truncated: 1,067,140 more chars]
